# Discovery of Zoanthamine Alkaloids from *Zoanthus vietnamensis* with Antioxidant and Neuroprotective Activities

**DOI:** 10.1021/acs.joc.5c00280

**Published:** 2025-04-02

**Authors:** Shu-Rong Chen, Yang-Chen Chang, Yi Chen, Yih-Fung Chen, Yu-Chi Lin, Cheng-chau Chiu, Yuan-Bin Cheng

**Affiliations:** † Department of Marine Biotechnology and Resources, National Sun Yat-sen University, Kaohsiung 80424, Taiwan; ‡ Graduate Institute of Natural Products, College of Pharmacy, Kaohsiung Medical University, Kaohsiung 80708, Taiwan; § School of Pharmacy, College of Pharmacy, Kaohsiung Medical University, Kaohsiung 80708, Taiwan; ∥ National Research Institute of Chinese Medicine, Ministry of Health and Welfare, Taipei 11221, Taiwan; ⊥ Department of Chemistry, National Sun Yat-sen University, Kaohsiung 80424, Taiwan; # Center for Theoretical and Computational Physics, National Sun Yat-sen University, Kaohsiung 80424, Taiwan

## Abstract

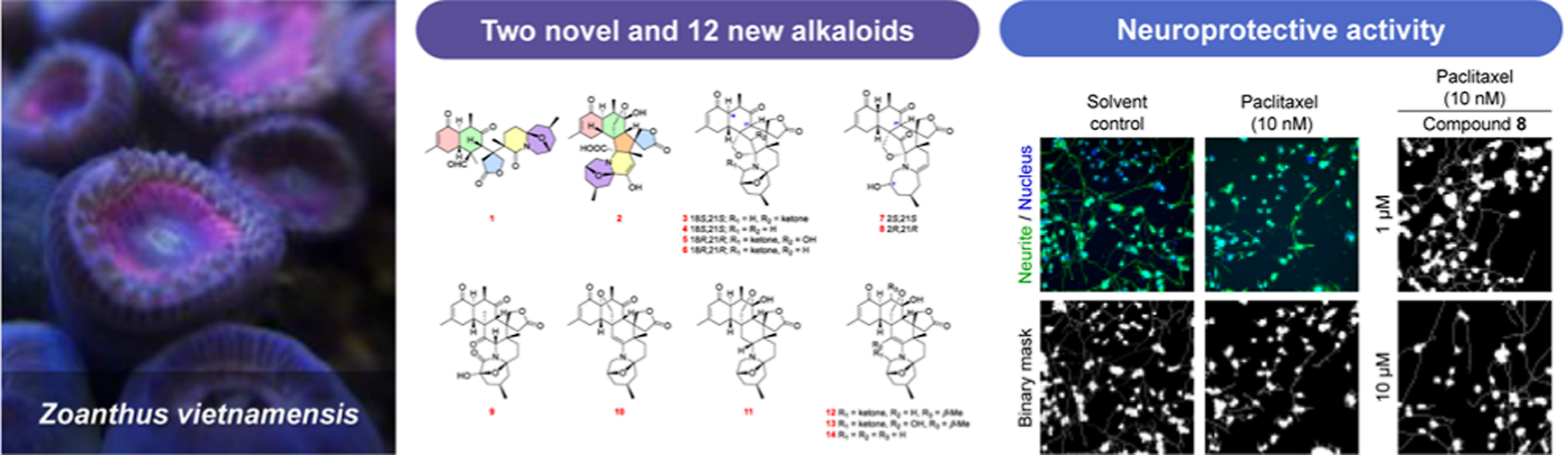

Two novel alkaloids, zoanides A and B (**1** and **2**), 12 new zoanthamine-type alkaloids (**3**–**14**), together with two known compounds (**15** and **16**) were obtained from the zoantharian *Zoanthus vietnamensis*. Their structures and absolute configurations were established by extensive spectroscopic data, DP4+ probability calculation, and X-ray crystallographic analyses. Structurally, compound **1** represents an unusual functionalized skeleton caused by oxidative cleavage; compound **2** possesses an unprecedented 6/6/5/6/7-fused pentacyclic carbon skeleton. Additionally, the plausible biosynthetic pathways of **1** and **2** were proposed. All isolates were evaluated in vitro for neuroprotection. Among them, compounds **2**, **3**, **8**, **9**, and **15** exhibited neuroprotection against paclitaxel-induced neurite damage without interfering with the anticancer effects of paclitaxel. Moreover, compounds **2** and **9** demonstrated moderate protective effects against oxaliplatin-induced oxidative stress overload without interfering with the anticancer effects of oxaliplatin.

The zoantharian genus *Zoanthus* (Zoanthidae), containing about 43 species, is distributed in tropical and subtropical regions. Previous chemical investigations on this marine invertebrate have led to the isolation of a series of secondary metabolites,^1^ including sterols,^2,3^ ecdysteroids,^4,5^ 2-aminoimidazole alkaloids,^6^ and zoanthamine alkaloids,^7−9^ some of which exhibit antiviral,^5^ antiosteoporotic,^10^ and neuroinflammatory^8^ activities. Zoanthamine alkaloids, characterized by a stereochemically dense heptacyclic skeleton, are primarily produced by zoantharians and can be recognized as biomarkers for these organisms. These complex and unusual alkaloids have also garnered significant attention from many synthetic groups for their potential total synthesis strategies.^11−15^

The taxane compound paclitaxel and the platinum-based agent oxaliplatin are commonly used chemotherapeutic drugs which lead to chemotherapy-induced peripheral neuropathy (CIPN). CIPN is one of the dose-limiting side effects and frequently results in the reduced dosage of paclitaxel and oxaliplatin or even the termination of anticancer therapy.^16^ Approximately 70% of cancer patients receiving paclitaxel- or oxaliplatin-based chemotherapy suffer from significant neuropathic symptoms,^17^ such as hypersensitivity to heat or cold, mechanical allodynia, and numbness.^18^ The accumulation of paclitaxel and oxaliplatin in the dorsal root ganglia (DRG) neurons is one of the major causes of neurotoxicity and further leads to the dysregulation of signal transmission. Despite extensive research efforts, even the most promising investigational agents typically achieve only partial prevention of chemotherapy-induced nerve damage. Till now, there is still no Food and Drug Administration (FDA)-approved therapy for preventing or treating CIPN.

In our research on bioactive marine natural products from Taiwanese indigenous zoantharians, the *Zoanthus vietnamensis* from the northern coastal area of Taiwan was collected. Using an acid–base extraction and repeated column chromatography isolation, our study on *Z. vietnamensis* resulted in the discovery of two novel (**1** and **2**), 12 new (**3**–**14**), and two known (**15** and **16**) zoanthamine alkaloids (Figure 1). Herein, we present the isolation, structure elucidation, and neuroprotective activity of all isolated compounds.

**Figure 1:**
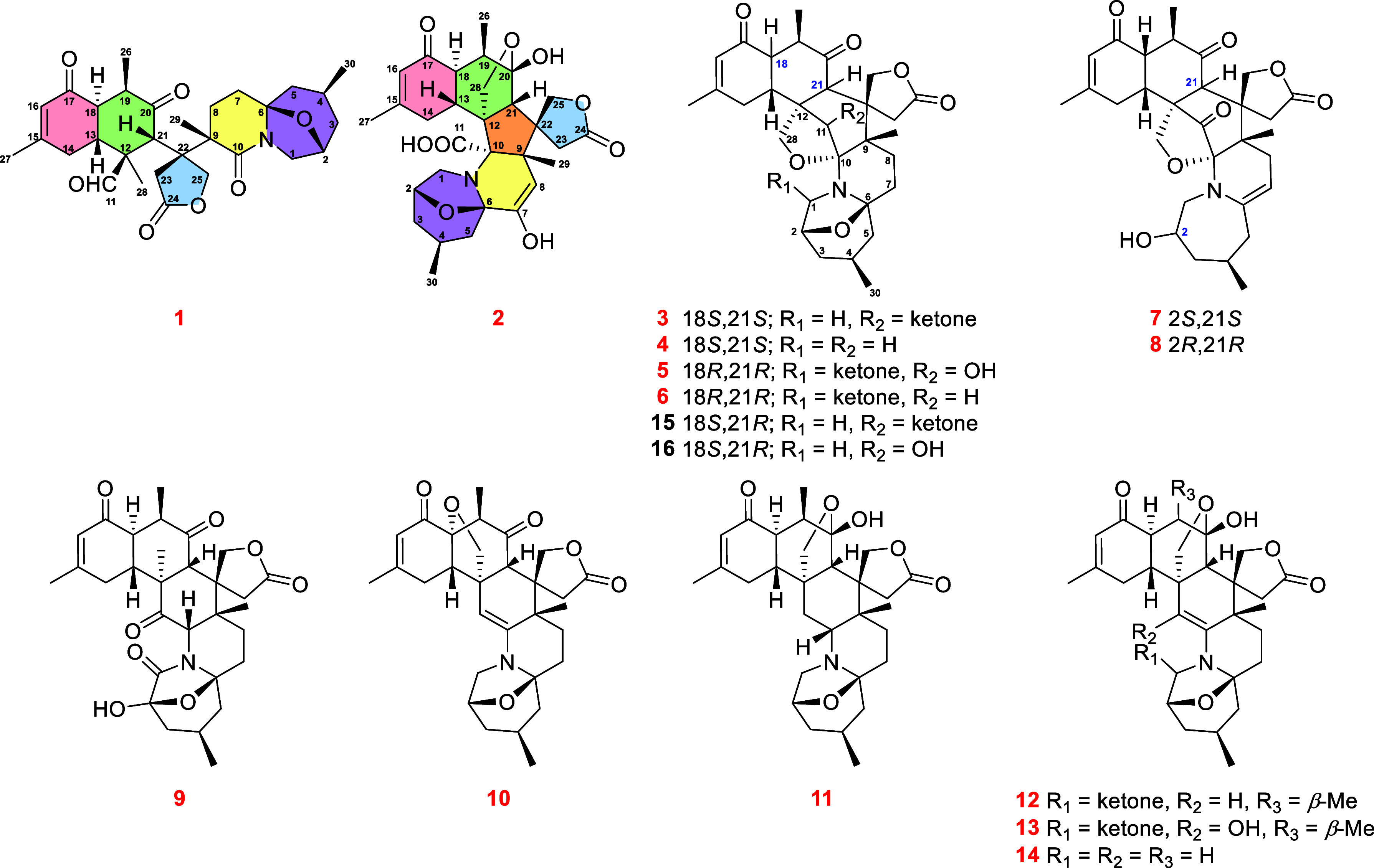
Structures of compounds **1**–**16**.

## Results and Discussion

Ethanol extract of *Z. vietnamensis* was solvent-partitioned by acid–base extraction to give four portions (hexanes, 75% MeOH_(aq)_, CH_2_Cl_2_, and aqueous layers). The CH_2_Cl_2_ layer was subjected to repetitive chromatography, including silica/C_18_ open column and NP/RP-HPLC to afford 14 new compounds; zoanides A and B (**1** and **2**), (18*S*,21*S*)-kuroshine E (**3**), (18*S*,21*S*)-11-dehydroxykuroshine A (**4**), 1-keto-kuroshine A (**5**), 1-keto-11-dehydroxykuroshine A (**6**), kuroshines L–P (**7**–**11**), 1-keto-zoanthenamine (**12**), 1-keto-11-hydroxyzoanthenamine (**13**), and 26-norzoanthenamine (**14**), along with two known compounds; 18-*epi*-kuroshine E (**15**),^19^ and 18-*epi*-kuroshine A (**16**).^19^

Zoanide A (**1**) was purified as colorless needle crystals. The molecular formula was determined as C_30_H_39_NO_7_, obtained by HRESIMS and NMR data, requiring 12 indices of hydrogen deficiency. The ^1^H NMR data of **1** (Table 1) exhibited several signals characteristic of 28-deoxyzoanthenamine,^20^ including five methyls [δ_H_ 0.79 (d, *J* = 6.5 Hz, Me-30), δ_H_ 1.25 (s, Me-28), δ_H_ 1.25 (s, Me-29), δ_H_ 1.46 (d, *J* = 7.2 Hz, Me-26), and δ_H_ 1.76 (s, Me-27)], two deshielded methylenes [δ_H_ 2.43 and 2.94 (d, *J* = 17.6 Hz, H_2_-23); δ_H_ 3.19 (d, *J* = 11.1 Hz, H-1a) and δ_H_ 3.77 (dd, *J* = 11.1, 6.5 Hz, H-1b)], one oxymethylene [δ_H_ 4.98 and 5.21 (d, *J* = 12.0 Hz, H_2_-25)], five deshielded methines [δ_H_ 2.79 (dd, *J* = 13.7, 5.1 Hz, H-18), δ_H_ 2.93 (m, H-13), δ_H_ 3.48 (dd, *J* = 7.2, 5.1 Hz, H-19), δ_H_ 4.49 (m, H-2), and δ_H_ 6.01 (s, H-21)], as well as one typical olefinic methine [δ_H_ 6.02 (s, H-16)]. The ^13^C{^1^H} and DEPT spectra showed a total of 30 carbon signals (Table 1) that were classified into seven sp^2^ carbon atoms (two ketones at δ_C_ 196.5 and 213.4; one aldehyde at δ_C_ 204.3; one amide at δ_C_ 173.3; one ester at δ_C_ 175.8; one double-bond at δ_C_ 126.4 and 161.8) and 23 sp^3^ carbon atoms (one hemiaminal quaternary carbon at δ_C_ 92.6; one oxymethine at δ_C_ 73.9; one oxymethylene at δ_C_ 74.5; three aliphatic quaternary carbons at δ_C_ 50.4, 51.8, and 59.4; five methines at δ_C_ 23.7, 36.1, 46.9, 48.0, and 51.1; seven methylenes at δ_C_ 26.8, 30.4, 30.5, 37.1, 37.6, 42.8, and 49.2; five methyls at δ_C_ 11.2, 12.9, 21.5, 21.7, and 24.1). The above functionalities accounted for six out of 12 degrees of unsaturation, requiring six additional rings in **1**.

**Table 1:** ^1^H and ^13^C{^1^H} NMR Data for **1–5** in C_5_D_5_N^a^

	**1**^b^		**2**^b^		**3**^b^		**4**^c^		**5**^c^	
no.	δ_H_	δ_c_, type	δ_H_	δ_c_, type	δ_H_	δ_c_, type	δ_H_	δ_c_, type	δ_H_	δ_c_, type
1	3.77, dd (11.1, 6.5)	49.2, CH_2_	3.82, d (13.9)	45.0, CH_2_	3.72,t (7.3)	47.0, CH_2_	3.17,t (6.4)	48.3, CH_2_		174.5, C
	3.19, d (11.1)		3.55, dd (13.9, 7.5)		2.74,d (8,4)		3.10, d (6.4)			
2	4.49, m	73.9, CH	4.53, m	68.5, CH	4.42, m	74.0, CH	4.48, m	73.8, CH	4.48, m	77.0, CH
3	1.41, dd (13.4, 5.3)	37.6, CH_2_	1.60, m	38.6, CH_2_	1.39, m	39.0, CH_2_	1.46, m	39.4, CH_2_	1.90, dd (13.2, 4.6)	33.3, CH_2_
	1.31, m		1.45, m				1.40, m		1.41, m	
4	1.69, m	23.7, CH	2.21, m	26.4, CH	2.19, m	23.3, CH	2.23, m	23.4, CH	2.25, m	24.3, CH
5	2.03, dd (13.2, 5.0)	42.8, CH_2_	2.60, dd (12.6, 3.5)	48.6, CH_2_	2.25, dd (12.4, 5.5)	44.2, CH_2_	2.27, m	44.7, CH_2_	2.32, m	40.2, CH_2_
	1.05, dd (13.2, 12.5)		1.77, t (12.6)		1.04, t (12.4)		1.02,t (12.0)		1.11, dd (12.9, 11.2)	
6		92.6, C		93.6, C		90.0, C		90.0, C		93.9, C
7	1.89, m	30.4, CH_2_		138.4, C	1.88, td (12.7, 3.9)	30.1, CH_2_	1.89, m	30.3, CH_2_	2.05, m	30.0, CH_2_
	1.73, m				1.61, dt (12.7, 3.7)		1.58, m		1.27, m	
8	1.93, m	26.8, CH_2_	5.81, s	107.3, CH	2.33, td (13.4, 3.9)	28.5, CH_2_	2.22, m	28.2, CH_2_	2.29, m	26.9, CH_2_
	1.70, m				1.68, dt (13.4, 3.9)		1.56, m		1.22, m	
9		50.4, C		54.7, C		49.9, C		44.5, C		47.5, C
10		173.3, C		83.4, C		96.1, C		101.4, C		93.7, C
11	9.98, s	204.3, CH		184.7, C		207.6, C	1.94,d (12.4)	38.5, CH_2_	6.65, s	69.6, CH
							1.67, d (12.4)			
12		59.4, C		61.6, C		55.2, C		52.0, C		52.0, C
13	2.93, m	36.1, CH	2.92, m	41.8, CH	2.78, m	35.8, CH	2.29, m	42.2, CH	3.06, m	32.8, CH
14	2.13, m	30.5, CH_2_	3.59, m	34.0, CH_2_	2.90, m	28.8, CH_2_	2.94, m	29.7, CH_2_	2.38, m	29.6, CH_2_
	1.84, m		2.77, m		2.14, m		2.16, m		1.75, m	
15		161.8, C		162.5, C		159.8, C		160.5, C		162.0, C
16	6.02, s	126.4, CH	5.97, s	126.2, CH	6.00, s	125.3, CH	5.99, s	125.0, CH	5.94, s	126.8, CH
17		196.5, C		199.4, C		196.5, C		197.4, C		197.2, C
18	2.79, dd (13.7, 5.1)	48.0, CH	2.91, m	50.3, CH	3.38, dd (13.3, 4.4)	51.0, CH	2.60, dd (13.1, 4.1)	53.0, CH	2.59, dd (14.1, 4.5)	47.9, CH
19	3.48, dd (7.2, 5.1)	46.9, CH	2.96, m	43.2, CH	2.85, m	41.9, CH	2.78, m	42.1, CH	3.51, dq (7.2, 4.5)	46.2, CH
20		213.4, C		111.0, C		207.8, C		209.3, C		214.1, C
21	6.01, s	51.1, CH	2.70, s	50.8, CH	4.16, s	66.9, CH	3.53, s	63.2, CH	3.83, s	48.4, CH
22		51.8, C		55.0, C		49.2, C		49.1, C		43.9, C
23	2.94, d (17.6)	37.1, CH_2_	4.39, d (17.0)	36.6, CH_2_	4.28, d (17.8)	43.1, CH_2_	3.87, d (17.6)	43.3, CH_2_	3.68, d (16.5)	35.5, CH_2_
	2.43, d (17.6)		2.96, d (17.0)		3.18, d (17.8)		2.78, d (17.6)		3.55, d (16.5)	
24		175.8, C		177.8, C		177.2, C		177.9, C		178.7, C
25	5.21, d (12.0)	74.5, CH_2_	4.97, d (9.5)	73.7, CH_2_	5.25, d (11.2)	74.8, CH_2_	5.28, d (10.9)	76.0, CH_2_	4.60, d (9.8)	74.3, CH_2_
	4.98, d (12.0)		4.83, d (9.5)		4.34, d (11.2)		4.23, d (10.9)		4.44, d (9.8)	
26	1.46, d (7.2)	12.9, CH_3_	1.45, d (6.7)	13.3, CH_3_	1.09, d (6.4)	12.3, CH_3_	1.12, d (6.3)	12.3, CH_3_	1.24, d (7.2)	13.3, CH_3_
27	1.76, s	24.1, CH_3_	1.71, s	24.2, CH_3_	1.85, s	23.8, CH_3_	1.87, s	23.9, CH_3_	1.31, s	23.7, CH_3_
28	1.25, s	11.2, CH_3_	4.50, d (10.2)	71.6, CH_2_	4.41, d (9.0)	70.2, CH_2_	3.92, d (8.4)	74.1, CH_2_	4.31, d (9.3)	66.6, CH_2_
			4.14, d (10.2)		4.14, d (9.0)		3.68, d (8.4)		4.09, d (9.3)	
29	1.25, s	21.7, CH_3_	1.56, s	22.1, CH_3_	1.37, s	20.1, CH_3_	1.43, s	23.0, CH_3_	1.48, s	22.9, CH_3_
30	0.79,d (6.5)	21.5, CH_3_	1.05, d (6.4)	22.1, CH_3_	0.83, d (6.5)	22.2, CH_3_	0.83, d (6.1)	22.2, CH_3_	0.87, d (6.5)	21.4, CH_3_

a Chemical shifts are in ppm. *J* values in Hz are in parentheses.

b ^1^H and ^13^C{^1^H} NMR were recorded at 600 and 150 MHz, respectively.

c ^1^H and ^13^C{^1^H} NMR were recorded at 400 and 100 MHz, respectively.

**Figure 2:**
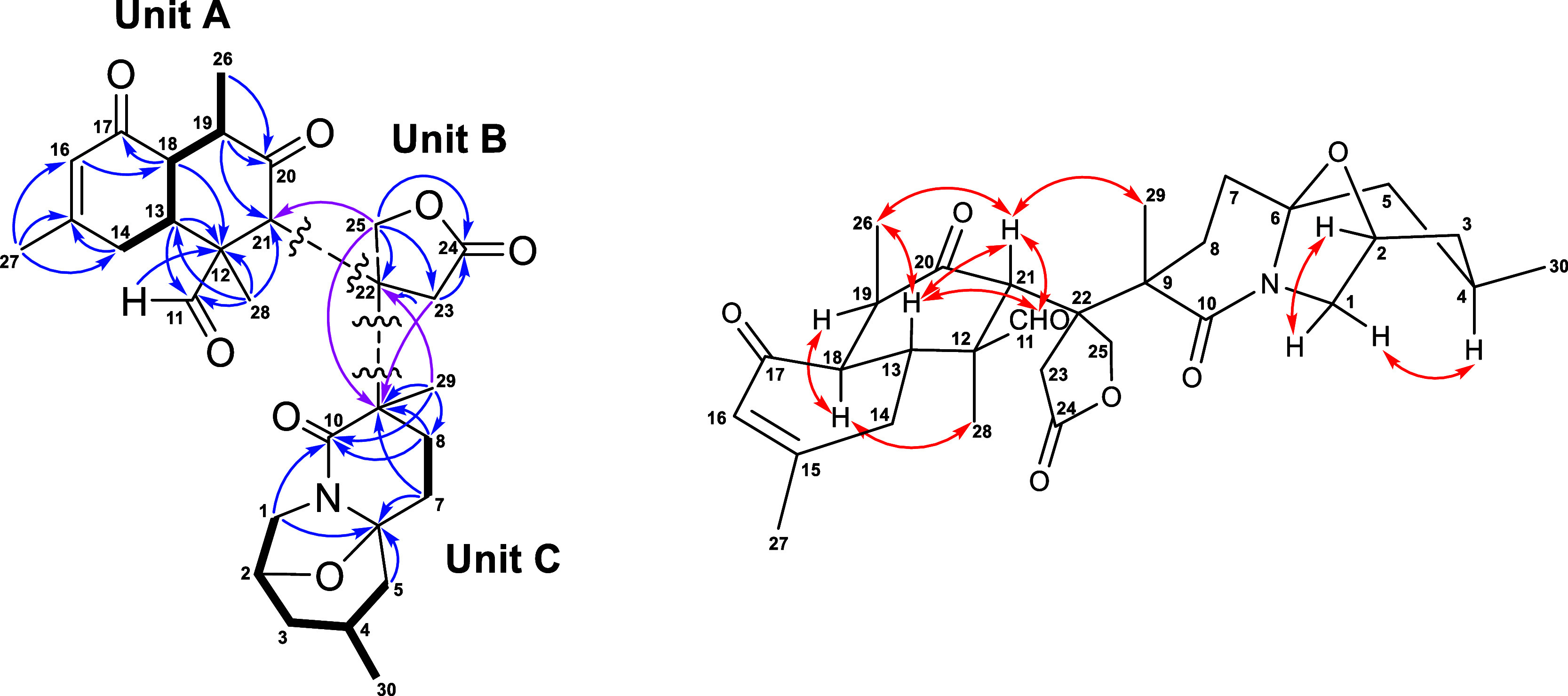
Key COSY (bold), HMBC (arrow), and NOESY (double arrow) correlations of **1**.

Analysis of 2D NMR spectra, including COSY, HSQC, and HMBC, elucidated the planar structure of **1**. Unit A (Figure 2), which possessed a 15-en-17-one moiety, an aldehyde at C-12, and three methyl groups attached at C-12, C-15, and C-19, was established by the COSY correlations of H_2_-14 (δ_H_ 1.84 and 2.13)/H-13/H-18/H-19/Me-26, along with the corresponding HMBC correlations from Me-28 to C-11 (δ_C_ 204.3)/C-12 (δ_C_ 59.4)/C-13 (δ_C_ 36.1)/C-21 (δ_C_ 51.1), from H-11 (δ_H_ 9.98)/H-13/H-18 to C-12, from H-19 to C-21, from Me-27 to C-14 (δ_C_ 30.5)/C-15 (δ_C_ 161.8)/C-16 (δ_C_ 126.4), from H-18 to C-17 (δ_C_ 196.5), and from H-16 to C-18 (δ_C_ 48.0). The presence of a γ-lactone moiety (unit B) was supported by the HMBC correlations from H_2_-23 to C-22 (δ_C_ 51.8)/C-24 (δ_C_ 175.8) and from H_2_-25 to C-22/C-23 (δ_C_ 37.1)/C-24. Moreover, the HMBC correlations from H_2_-1/H_2_-5 (δ_H_ 1.05 and 2.03)/H_2_-7 (δ_H_ 1.73 and 1.89) to C-6 (δ_C_ 92.6), from H_2_-1/H_2_-8 (δ_H_ 1.70 and 1.93) to C-10 (δ_C_ 173.3), from H_2_-7/H_2_-8 to C-9 (δ_C_ 50.4), and from Me-29 to C-8 (δ_C_ 26.8)/C-9/C-10, as well as the COSY cross-peaks of H_2_-1/H-2/H_2_-3 (δ_H_ 1.31 and 1.41)/H-4 (δ_H_ 1.69)/H_2_-5 and H-4/Me-30, constructed the unit C (Figure 2). These three partial structures (units A, B, and C) were connected based on key HMBC correlations from H_2_-23/H_2_-25 to C-9, Me-29 to C-22, and H_2_-25 to C-21. Finally, the novel skeleton of **1** was identified as shown.

**Figure 3:**
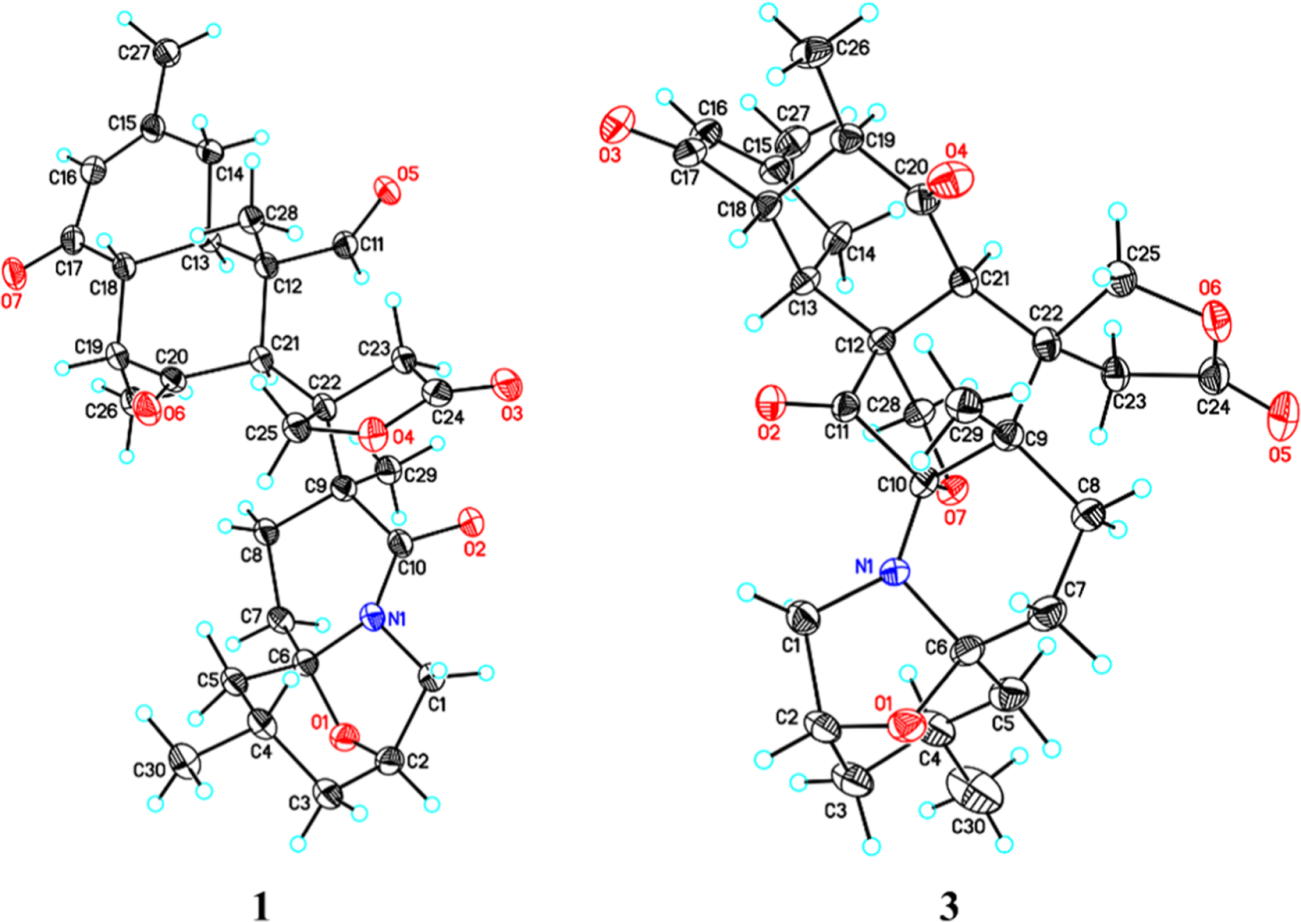
X-ray ORTEP drawings of **1** and **3** (displacement ellipsoids are drawn at the 50% probability level).

In the NOESY spectrum of **1**, the cross-peaks of H-1α (δ_H_ 3.19)/H-4 and H-19/H-18/Me-28 indicated their cofacial arrangement and tentatively assigned as α-orientation (Figure 2). On the other hand, the correlations of H-1β (δ_H_ 3.77)/H-2 and Me-29/H-21/H-11/H-13/Me-26 suggested that these protons possessed β-orientation (Figure 2). Fortunately, single-crystal X-ray diffraction analysis using Cu Kα radiation of **1** was successfully conducted to confirm the planar structure and the stereochemistry. Consequently, the absolute configuration of **1** was assigned as 2*R*,4*S*,6*S*,9*S*,12*S*,13*R*,18*R*,19*R*,21*R*,22*R* with an absolute structure parameter of −0.12(11) [Figure 3, CCDC 2055085].

Zoanide B (**2**) was isolated as a white amorphous powder. The molecular formula was deduced as C_30_H_37_NO_9_ from the HRESIMS ion peak at *m*/*z* 578.2352 ([M + Na]^+^, calcd 578.2361), indicating 13 degrees of unsaturation. Numerous signals that are typical of zoanthenamine^20^ were present in the ^1^H NMR data of **2** (Table 1), including four methyls [δ_H_ 1.45 (d, *J* = 6.7 Hz, Me-26), δ_H_ 1.71 (s, Me-27), δ_H_ 1.56 (s, Me-29), and δ_H_ 1.05 (d, *J* = 6.4 Hz, Me-30)], two deshielded methylenes [δ_H_ 3.55 (dd, *J* = 13.9, 7.5 Hz, H-1a) and δ_H_ 3.82 (d, *J* = 13.9 Hz, H-1b); δ_H_ 2.96 and 4.39 (d, *J* = 17.0 Hz, H_2_-23)], two oxymethylenes [δ_H_ 4.83 and 4.97 (d, *J* = 9.5 Hz, H_2_-25); δ_H_ 4.14 and 4.50 (d, *J* = 10.2 Hz, H_2_-28)], one oxymethine [δ_H_ 4.53 (m, H-2)], four deshielded methines [δ_H_ 2.92 (m, H-13), δ_H_ 2.91 (m, H-18), δ_H_ 2.96 (m, H-19), and δ_H_ 2.70 (s, H-21)], and one distinctive olefinic methine [δ_H_ 5.97 (s, H-16)]. With the help of HSQC spectrum and ^13^C{^1^H} NMR data (Table 1), the 30 carbon were assigned as four methyls (δ_C_ 13.3, 22.1, 22.1, and 24.2), seven methylenes (two oxygenated at δ_C_ 71.6 and 73.7), eight methines (two olefinic carbons at δ_C_ 107.3 and 126.2; one oxygenated at δ_C_ 68.5), and 11 quaternary carbons (three carbonyls at δ_C_ 177.8, 184.7, and 199.4; two olefinic at δ_C_ 138.4 and 162.5; one hemiketal at δ_C_ 111.0; and one hemiaminal at 93.6). Three carbonyl groups and two double bonds accounted for five degrees of unsaturation, and the remaining eight degrees of unsaturation required compound **2** to have another octacyclic carbon skeleton.

**Figure 4:**
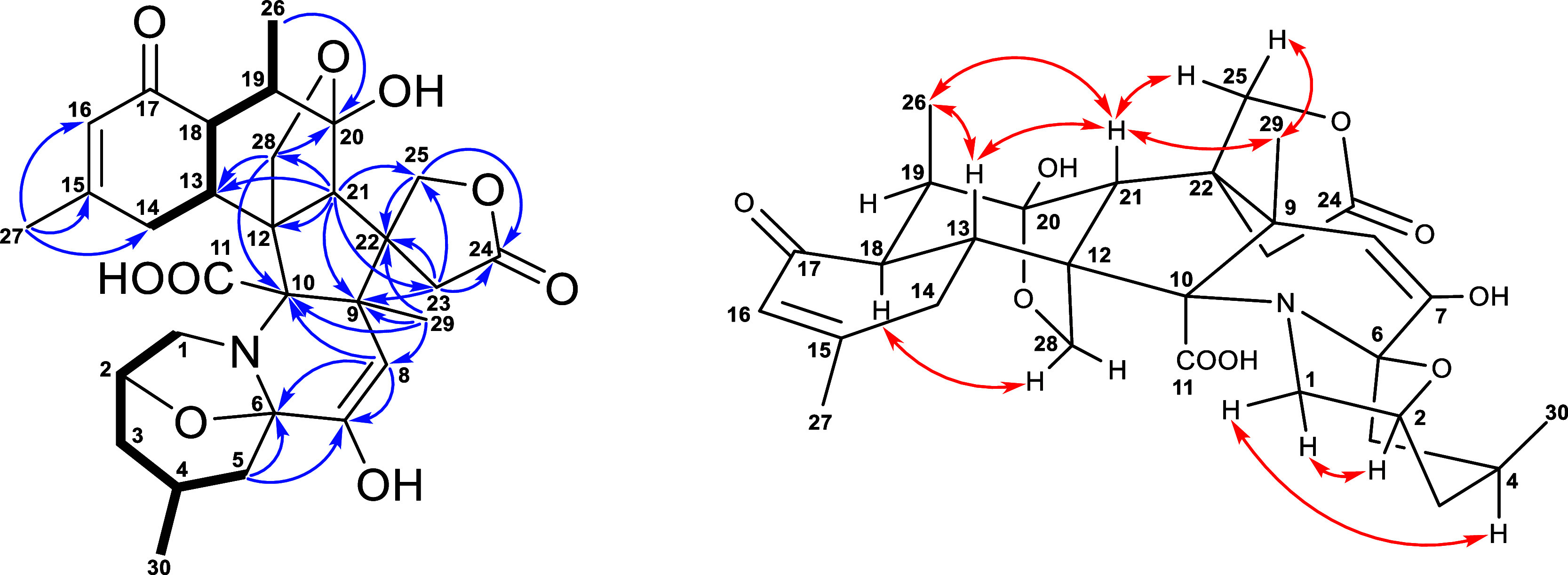
Key COSY (bold), HMBC (arrow), and NOESY (double arrow) correlations of **2**.

The planar structure of **2** was elucidated by a detailed analysis of the COSY and HMBC correlations (Figure 4). The relative configurations of **2** were determined by the NOESY spectrum (Figure 4). The NOESY correlations of H-1β (δ_H_ 3.55)/H-2 (δ_H_ 4.53) and Me-29 (δ_H_ 1.56)/H_2_-25 (δ_H_ 4.83 and 4.97)/H-21 (δ_H_ 2.70)/H-13 (δ_H_ 2.92)/Me-26 (δ_H_ 1.45) indicated that these protons were coplanar and assigned as β-oriented. On the contrary, the NOESY correlations of H-1α (δ_H_ 3.82)/H-4 (δ_H_ 2.21) and H-18 (δ_H_ 2.91)/H-28 (δ_H_ 4.50) revealed that they were α-oriented. The above highly similar NOESY correlations exhibited that the relative configuration of **2** was consistent with that of zoanthenamine (the absolute configuration was established by X-ray crystallographic analysis, CCDC 2046949),^21^ suggesting that **2** and zoanthenamine share the same absolute configuration.

**Figure 5:**
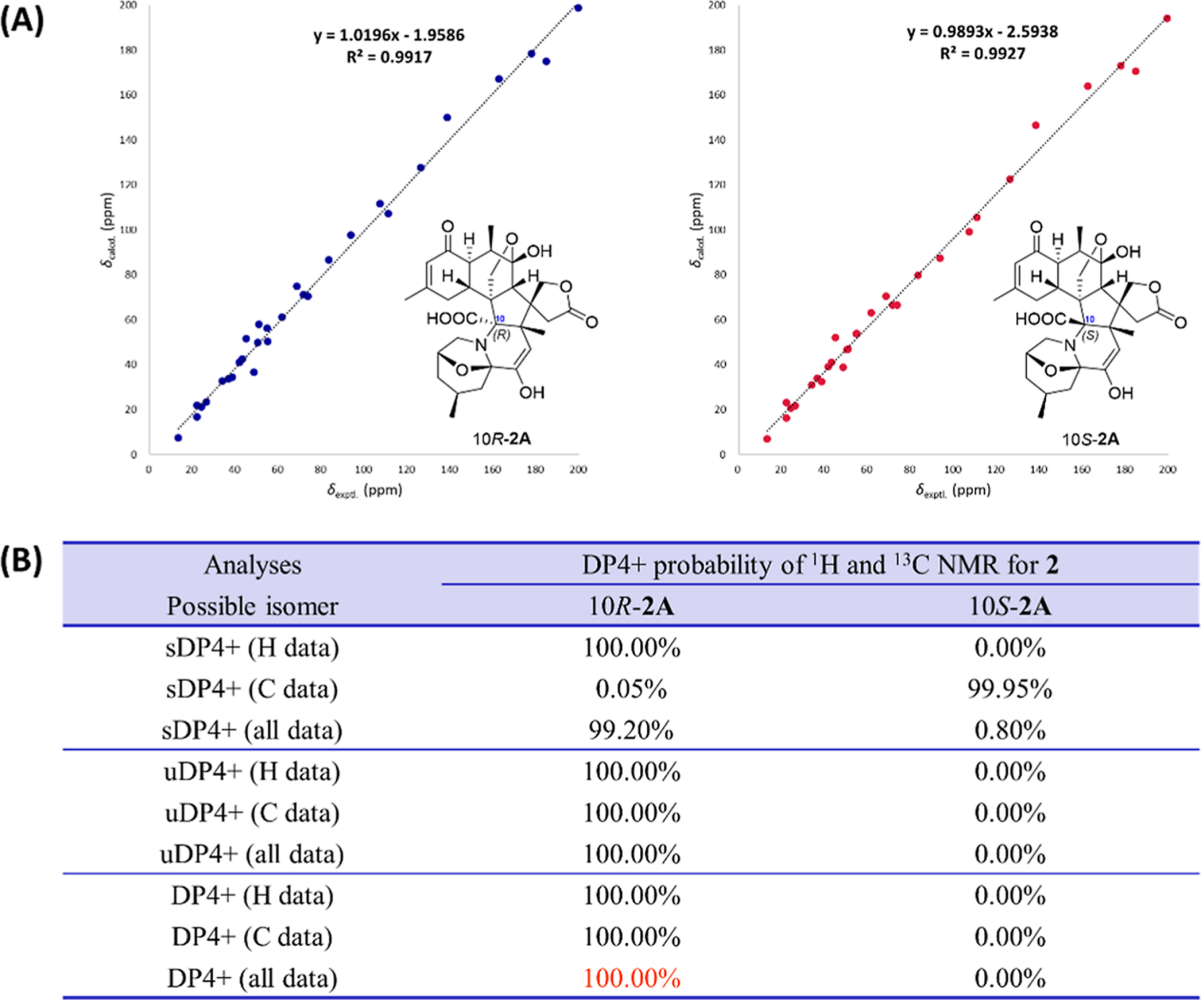
NMR calculations with the DP4+ probability analysis of **2**. (A) Linear correlations between the experimental and calculated ^13^C{^1^H} NMR chemical shifts for 10*R*-**2** and 10*S*-**2**. (B) DP4+ probability analyses of 10*R*-**2** and 10*S*-**2**.

**Figure 6:**
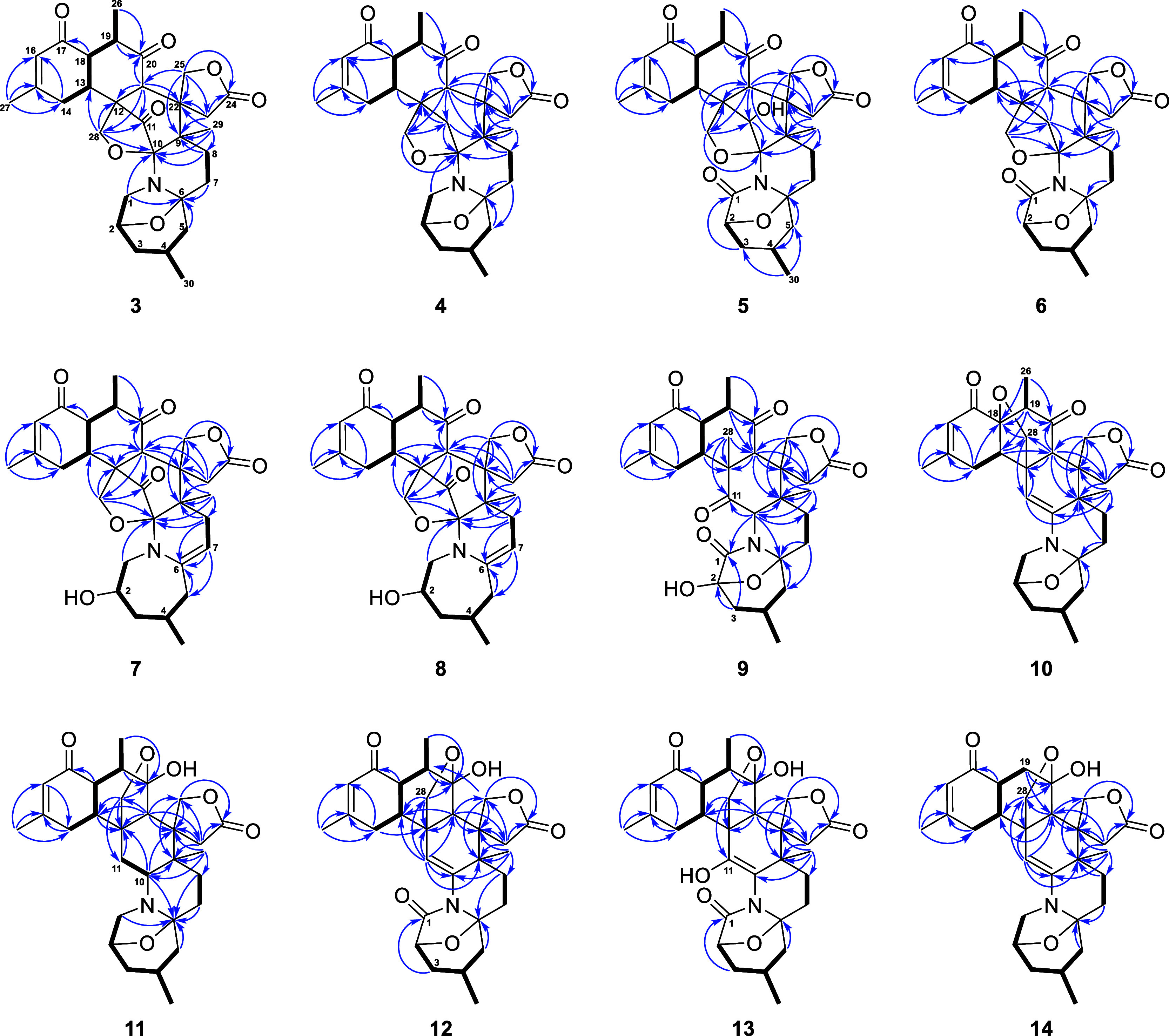
Key COSY (bold) and HMBC (arrow) correlations of **3**–**14**.

**Figure 7:**
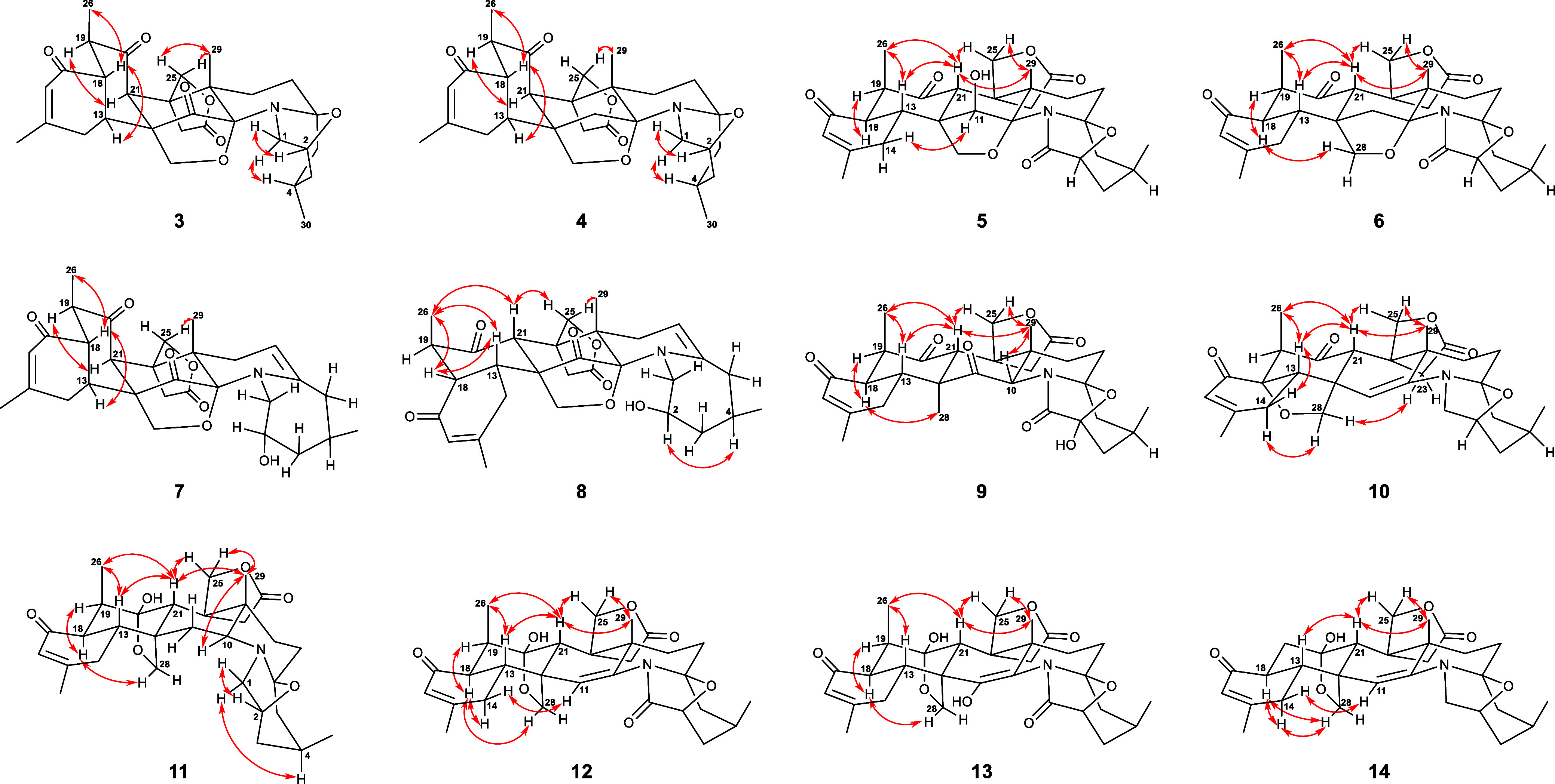
Key NOESY (double arrow) correlations of **3**–**14**.

Subsequently, the structure of **2** was confirmed using the quantum chemical prediction (QCP)^22,23^ of 1D NMR chemical shifts with DP4+ probability analyses,^24^ a potent and widely applicable approach for determining the proper structures of natural products with complicated frameworks. However, the stereochemistry of C-10 in **2** cannot be determined by the NOESY analysis. Thus, the ^1^H and ^13^C{^1^H} NMR calculations for 10*R*-**2** and 10*S*-**2** were carried out using the gauge-independent atomic orbital (GIAO) method at the mPW1PW91/6–311G(d,p) level in pyridine. The result displayed that the calculated ^13^C{^1^H} NMR chemical shifts of 10*R*-**2** and 10*S*-**2** both agreed well with the experimental NMR data with good linear correlation coefficients (*R*^2^ = 0.9917 and 0.9927, respectively) (Figure 5A). With a 100% probability, 10*R*-**2** was the most credible structure according to further DP4+ calculations based on both ^1^H and ^13^C{^1^H} NMR data (Figure 5B). Eventually, the absolute configuration of **2** was identified as 2*R*,4*S*,6*R*,9*S*,10*R*,12*R*,13*R*,18*R*,19*R*,20*S*,21*R*,22*R*.

(18*S*,21*S*)-Kuroshine E (**3**) was obtained as a colorless needle crystal, and its molecular formula was established as C_30_H_37_NO_7_ by HRESIMS ([M + Na]^+^*m*/*z* 546.2465). The planar structure of **3** (Figure 6) was identical to those of kuroshine E^25^ (CCDC 2212110) by comprehensively elucidating 1D and 2D NMR spectra (Table 1). The C-18 and C-21 chemical shifts between **3** (δ_H_ 3.38/δ_C_ 51.0; δ_H_ 4.16/δ_C_ 66.9) and kuroshine E (δ_H_ 2.26/δ_C_ 46.9; δ_H_ 3.63/δ_C_ 57.9)^25^ were dissimilar, which indicated that they are epimers at C-18 and C-21. The relative configurations of **3** were examined using the NOESY correlations (Figure 7). The key NOESY resonances of H-1β (δ_H_ 3.72)/H-2 (δ_H_ 4.42), H_2_-25 (δ_H_ 4.34 and 5.25)/Me-29 (δ_H_ 1.37), and H-13 (δ_H_ 2.78)/H-18 (δ_H_ 3.38)/Me-26 (δ_H_ 1.09) were observed, resulting in placing these protons on the same face of the molecule, while H-1α (δ_H_ 2.74)/H-4 (δ_H_ 2.19) and H-19 (δ_H_ 2.85)/H-21 (δ_H_ 4.16) were revealed to be on the opposite face. Finally, the absolute configuration of **3** was determined as 2*R*,4*S*,6*S*,9*S*,10*S*,12*S*,13*R*,18*S*,19*R*,21*S*,22*R* by a single-crystal X-ray crystallographic analysis [Figure 3, absolute structure parameter: 0.017(5), CCDC 2206603].

(18*S*,21*S*)-11-Dehydroxykuroshine A (**4**) was isolated as an isomer of 11-dehydroxy-18-*epi*-kuroshine A^21^ with a molecular formula of C_30_H_39_NO_6_ by its HRESIMS data. A detailed analysis of 1D and 2D NMR spectra, including COSY, HSQC, and HMBC, led to the planar structure of **4** (Figure 6). The NOESY correlations of **4** (Figure 7) were identical to that of **3**, for which the absolute configuration was defined by X-ray analysis. Hence, the absolute configuration of **4** was assigned based on shared biogenesis.

The molecular formula of 1-keto-kuroshine A (**5**) was deduced as C_30_H_37_NO_8_ based on the HRESIMS for the sodiated adduct [M + Na]^+^ at *m*/*z* 562.2410. The ^1^H and ^13^C{^1^H} data of **5** (Table 1) were almost the same as those of kuroshine A,^26^ except for one additional carbonyl signal (δ_C_ 174.5) and the missing methylene signal of H_2_-1, which implied the H_2_-1 was replaced by a carbonyl group in **5**. This assignment was supported by the HMBC correlation (Figure 6) from H-3 (δ_H_ 1.41) to C-1 (δ_C_ 174.5). The NOESY cross-peaks observed for H-13 (δ_H_ 3.06)/H-21 (δ_H_ 3.83), H-13/Me-26 (δ_H_ 1.24), H-21/H-25 (δ_H_ 4.60), H-21/Me-26, H-21/Me-29 (δ_H_ 1.48), and H-25 (δ_H_ 4.44)/Me-29 indicated that these protons and methyl groups wer β-oriented. In contrast, the correlations of H-11 (δ_H_ 6.65)/H-14 (δ_H_ 2.38) and H-18 (δ_H_ 2.59)/H-19 (δ_H_ 3.51) suggested H-11, H-18, and H-19 were α-oriented. The similarity NOESY correlations (Figure 7) demonstrated that **5** had the same configuration as (2*R*,4*S*,6*S*,9*S*,10*S*,12*S*,13*R*,18*R*,19*R*,21*R*,22*R*)-kuroshine A,^26^ in which the absolute configuration was established by X-ray crystallographic analysis; accordingly, the structure of **5** was depicted as shown.

1-Keto-11-dehydroxykuroshine A (**6**) gave a sodiated molecular ion peak by HRESIMS spectrum at *m*/*z* 546.2464 [M + Na]^+^ for a molecular formula of C_30_H_37_NO_7_Na^+^ (calcd 546.2462). The structure of **6** was characterized as an analogue of **5** based on the similarity of the 1D and 2D NMR data (Table 2). The main difference between **5** and **6** was that the oxymethine at C-11 in **5** was reduced to methylene (δ_H_ 4.81 and 2.15/δ_C_ 36.0) in **6**, which was supported by the HMBC correlations (Figure 6) from H_2_-11 (δ_H_ 4.81 and 2.15) to C-9 (δ_C_ 44.8)/C-10 (δ_C_ 97.0)/C-12 (δ_C_ 50.2)/C-13 (δ_C_ 35.3)/C-21 (δ_C_ 55.3) and from H_2_-28 (δ_H_ 4.28 and 4.02) to C-11 (δ_C_ 36.0). The relative configuration of **6** was determined to be the same as those of **5** based on their close similarity NOESY correlations (Figure 7). Thus, compound **6** was elucidated as shown.

**Table 2:** ^1^H and ^13^C{^1^H} NMR Data for **6–10** in C_5_D_5_N^a^

	**6**^b^		**7**^b^		**8**^c^		**9**^b^		**10**^b^	
no.	δ_H_	δ_c_, type	δ_H_	δ_c_, type	δ_H_	δ_c_, type	δ_H_	δ_c_, type	δ_H_	δ_c_, type
1		175.5, C	3.74, dd (15.1, 9.0)	57.6, CH_2_	3.88, m	58.0, CH_2_		173.1, C	3.11, m	50.8, CH_2_
			3.23, d (15.1)		2.13, d (14.5)					
2	4.48, m	76.9, CH	4.15, m	71.7, CH	4.09, m	71.8, CH		101.7, C	4.60, m	73.9, CH
3	1.89, dd (12.7, 4.4)	33.4, CH_2_	2.26, m	48.0, CH_2_	2.26, m	48.1, CH_2_	2.30, dd (12.7, 5.8)	38.7, CH_2_	1.46, m	38.4, CH_2_
	1.39, dd (3.7, 1.4)		1.37, m		2.23, m		1.73, m		1.39, m	
4	2.23, m	24.3, CH	1.73, m	34.1, CH	1.74, m	34.0, CH	3.02, m	24.9, CH	1.66, m	23.4, CH
5	2.30, dd (13.6, 3.4)	40.0, CH_2_	2.05, m	40.9, CH_2_	2.12, m	40.8, CH_2_	2.16, dd (13.3, 5.6)	39.1, CH_2_	1.80, m	44.5, CH_2_
	1.10, m						1.09, m		1.02, t (13.0)	
6		93.6, C		141.8, C		142.1, C		88.3, C		89.9, C
7	1.95, m	29.8, CH_2_	4.26, dd (6.8, 1.7)	91.6, CH	4.32, d (7.0)	90.4, CH	1.92, dt (13.4, 3.2)	29.9, CH_2_	2.06, m	29.9, CH_2_
	1.76, m						1.76, m		1.81, m	
8	2.20, m	25.2, CH_2_	2.90, m	29.5, CH_2_	2.76, m	26.4, CH_2_	1.99, m	26.7, CH_2_	1.79, m	25.4, CH_2_
	1.35, m		1.94, m		1.64, m		1.64, m		1.54, m	
9		44.8, C		49.0, C		49.0, C		43.4, C		40.8, C
10		97.0, C		97.7, C		96.7, C	5.10, s	62.8, CH		146.7, C
11	4.81, d (12.7)	36.0, CH_2_		210.6, C		208.5, C		208.1, C	4.11, s	89.8, CH
	2.15, d (12.7)									
12		50.2, C		55.5, C		55.7, C		54.2, C		51.3, C
13	2.93, ddd (16.8, 11.9, 4.9)	35.3, CH	2.37, m	35.7, CH	3.43, m	31.9, CH	3.28, m	44.1, CH	3.11, m	50.8, CH
14	2.64, dd (17.9, 5.0)	29.8, CH_2_	2.84, m	28.9, CH_2_	2.36, dd (19.4, 5.8)	31.3, CH_2_	2.35, m	31.5, CH_2_	2.66, dd (18.3, 9.4)	29.3, CH_2_
	2.53, m		1.89, m		2.19, m				2.53, dd (18.3, 6.5)	
15		161.8, C		160.0, C		160.6, C		160.4, C		162.1, C
16	6.00, s	126.8, CH	6.02, s	125.4, CH	5.90, s	126.1, CH	5.83, s	126.6, CH	6.13, s	125.5, CH
17		196.9, C		196.5, C		196.6, C		196.3, C		191.8, C
18	2.54, dd (13.9, 4.7)	47.4, CH	3.47, dd (13.4, 4.7)	50.8, CH	2.96, t (5.3)	49.7, CH	2.88, dd (13.5, 5.6)	48.3, CH		86.4, C
19	3.47, dq (7.2, 3.1)	46.0, CH	2.86, m	41.9, CH	3.26, m	43.6, CH	3.26, m	46.3, CH	3.74, q (7.4)	53.8, CH
20		212.5, C		207.6, C		210.3, C		210.1, C		211.7, C
21	3.32, s	55.3, CH	4.23, s	67.0, CH	3.54, s	56.2, CH	3.99, s	50.8, CH	3.66, s	52.4, CH
22		47.6, C		49.1, C		47.0, C		48.3, C		45.0, C
23	3.57, d (19.3)	34.9, CH_2_	4.35, d (17.7)	43.8, CH_2_	3.83, d (18.7)	34.3, CH_2_	3.42, d (17.1)	35.2, CH_2_	3.50, d (18.1)	34.5, CH_2_
	3.51, d (19.3)		3.18, d (17.7)		3.73, d (18.7)		3.16, d (17.1)		2.57, d (18.1)	
24		178.5, C		177.0, C		177.9, C		176.2, C		177.1, C
25	4.47, d (9.8)	74.4, CH_2_	5.28, d (11.4)	74.6, CH_2_	4.45, d (10.0)	73.6, CH_2_	4.90, d (9.7)	70.9, CH_2_	4.65, d (9.6)	72.0, CH_2_
	4.34,d (9.8)		4.29, d (11.4)		4.18, d (10.0)		4.67, d (9.7)		4.40, d (9.6)	
26	1.22, d (7.4)	13.7, CH_3_	1.10, d (6.4)	12.3, CH_3_	1.25, d (6.4)	16.7, CH_3_	1.26, d (6.9)	13.5, CH_3_	1.17, d (7.0)	15.0, CH_3_
27	1.75, s	24.2, CH_3_	1.91, s	23.9, CH_3_	1.66, s	23.6, CH_3_	1.71, s	24.0, CH_3_	1.95, s	24.4, CH_3_
28	4.28, d (9.0)	69.5, CH_2_	4.48,d (9.4)	70.0, CH_2_	4.25, d (10.2)	66.5, CH_2_	1.23, s	14.6, CH_3_	4.14, d (9.8)	75.3, CH_2_
	4.02, d (9.0)		4.10,d (9.4)		4.03, d (10.2)				3.87, d (9.8)	
29	1.09, s	23.5, CH_3_	1.32, s	19.0, CH_3_	0.96, s	18.9, CH_3_	1.25, s	24.2, CH_3_	1.36, s	25.0, CH_3_
30	0.85, d (6.5)	21.4, CH_3_	0.91,d (6.7)	23.8, CH_3_	0.93, d (6.6)	23.8, CH_3_	0.95, d (6.7)	21.3, CH_3_	0.73, d (6.5)	21.6, CH_3_

a Chemical shifts are in ppm. *J* values in Hz are in parentheses.

b ^1^H and ^13^C{^1^H} NMR were recorded at 600 and 150 MHz, respectively.

c ^1^H and ^13^C{^1^H} NMR were recorded at 400 and 100 MHz, respectively.

Kuroshine L (**7**) was isolated as a white amorphous powder, and its molecular formula was determined by HRESIMS as C_30_H_37_NO_7_ based on observing the sodiated adduct [M + Na]^+^ at 546.24603. The 1D and 2D NMR data (Table 2) of **7** resembled those of **3**, implying that **7** belonged to the same framework. The most detectable difference was that the hemiaminal carbon at C-6 and methylene at C-7 in **3** were altered to a C=C double bond in **7** (C-6: δ_C_ 141.8, C; C-7: δ_H_ 4.26, δ_C_ 91.6, CH), which caused the ether linkage between C-2 and C-6 to be absent. The above assumption was verified by the COSY cross-peaks (Figure 6) between H-7 and H_2_-8 (δ_H_ 1.94 and 2.90), the critical HMBC correlations (Figure 6) from H_2_-5 (δ_H_ 2.05)/H-7/H_2_-8 to C-6, as well as the MS data of **7**. Thus, the planar structure of **7** was confirmed and illustrated as shown. The relative configuration of **7** was established by the correlations observed in the NOESY spectrum. The NOESY correlations of H-13 (δ_H_ 2.37)/H-18 (δ_H_ 3.47)/Me-26 (δ_H_ 1.10) and H-25 (δ_H_ 5.28)/Me-29 (δ_H_ 1.32) revealed that these protons and methyls were β-oriented, whereas the cross-peaks of H-19 (δ_H_ 2.86)/H-21 (δ_H_ 4.23) indicated that they were α-oriented (Figure 7). In determining the orientation of the hydroxy group at C-2, the NOESY data were ineffective. Accordingly, the NMR calculations and DP4+ analysis for 2*R*-**7** and 2*S*-**7** were conducted (Tables S12–S14). The analysis resulted in an 89.65% probability for the experimental data of **7** to match 2*S*-**7**, suggesting that the absolute configuration of **7** should be 2*S*. Therefore, the structure of **7** was identified.

Kuroshine M (**8**) was found to have an identical molecular formula to **7**. A comparison of the ^1^H and ^13^C{^1^H} NMR spectra between **7** and **8** (Table 2) demonstrated that the chemical shifts of H-21 (δ_H_ 4.23 in **7**, δ_H_ 3.54 in **8**) and C-21 (δ_C_ 67.0 in **7**, δ_C_ 56.2 in **8**) were different. Furthermore, the planar structure of **8** elucidated by comprehensive 2D NMR analysis (Figure 6) was the same as that of **7**, which disclosed that **8** is a C-21 epimer of **7**. The NOESY spectrum of **8** exhibited correlations of H-13 (δ_H_ 3.43)/H-18 (δ_H_ 2.96)/Me-26 (δ_H_ 1.25)/H-21/H_2_-25 (δ_H_ 4.18 and 4.45)/Me-29 (δ_H_ 0.96), indicating the β-orientation of these protons (Figure 7). The presence of a NOESY cross-peak between H-2 (δ_H_ 4.09) and H-4 (δ_H_ 1.74) revealed that H-2 and H-4 were α-orientated (Figure 7), which was supported by the NMR calculation with DP4+ probability for 2*R*-**8** (99.99% for all NMR data, Tables S19–S21). Hence, the structure of **8** was ascertained and illustrated as shown.

Kuroshine N (**9**) was deduced to process a molecular formula of C_30_H_37_NO_8_ from HRESIMS of the sodiated adduct [M + Na]^+^ at *m*/*z* 562.2407. The UV and NMR data (Table 2) were similar to those of 28-deoxyzoanthenamine,^20^ inferring that they shared the same framework. In comparison with this known compound, three variations of **9** were observed. One was that the double bond Δ^10,11^ in 28-deoxyzoanthenamine was replaced by a N-substituted methine (C-10: δ_H_ 5.10/δ_C_ 62.8) and a ketone group (C-11: δ_C_ 208.1) in **9**, which was supported by the HMBC correlations (Figure 6) from H-10 to C-1 (δ_C_ 173.1)/C-6 (δ_C_ 88.3)/C-8 (δ_C_ 26.7)/C-9 (δ_C_ 43.4)/C-11, from Me-28 (δ_H_ 1.23) to C-11, and from Me-29 (δ_H_ 1.25) to C-10. Another was that methylene at C-1 in 28-deoxyzoanthenamine was oxidized to the ketone (C-1: δ_C_ 173.1) in **9**, according to the HMBC correlations (Figure 6) from H_2_-3 (δ_H_ 1.73 and 2.30)/H-10 to C-1. The other was that an additional hydroxy group was found to be attached at C-2 in **9**, which was corroborated by the chemical shift at C-2 (δ_C_ 101.7) and the key HMBC resonances (Figure 6) from H_2_-3 to C-2. The NOESY spectrum showed correlations similar to those of 28-deoxyzoanthenamine (CCDC 2212109). In addition, the orientation of H-10 was assigned as β-face based on the NOESY cross-peaks (Figure 7) between H-10 and Me-29. Consequently, the structure of **9** was defined as shown.

The molecular formula of **10** was determined to be C_30_H_37_NO_6_ by HRESIMS ([M + Na]^+^*m*/*z* 530.2516, calcd 530.2513) and ^13^C{^1^H} NMR data (Table 2), indicating 13 degrees of unsaturation. A comparison of the 1D NMR data of 10 (Table 2) with 28-deoxyzoanthenamine^20^ revealed that they shared the same carbon skeleton. Moreover, missing the signals for H-18 and Me-28 suggested the presence of an ether linkage between C-18 (δ_C_ 86.4) and C-28 (δ_H_ 4.14 and 3.87/δ_C_ 75.3) in **10**. This assumption was validated by the HMBC correlation (Figure 6) from H_2_-28 to C-18 and one more degree of unsaturation than 28-deoxyzoanthenamine (12 degrees of unsaturation).^20^ The NOESY correlations (Figure 7) of Me-29 (δ_H_ 1.36)/H_2_-25 (δ_H_ 4.65 and 4.40)/H-21 (δ_H_ 3.66)/Me-26 (δ_H_ 1.17)/H-13 (δ_H_ 3.11)/H-14β (δ_H_ 2.53) indicated that these protons have the same β orientation. Contrarily, the NOESY correlations of H-14α (δ_H_ 2.66)/H_2_-28 (δ_H_ 4.14 and 3.87)/H-23 (δ_H_ 3.50) displayed they were on the other face of this molecule. The similar NOESY correlations between **10** and 28-deoxyzoanthenamine (CCDC 2212109) demonstrated the same stereochemistry. Consequently, the structure of **10** was established and named kuroshine O.

Compound **11**, which has two more hydrogens than zoanthenamine, was assigned the molecular formula C_30_H_41_NO_6_. The ^1^H and ^13^C{^1^H} NMR data (Table 3) of **11** highly resembled that of zoanthenamine. The major difference was noted in the chemical shifts of C-10 (δ_H_ 2.70/δ_C_ 61.3 in **11**, δ_C_ 144.6 in zoanthenamine) and C-11 (δ_H_ 1.69 and 1.59/δ_C_ 26.1 in **11**, δ_H_ 4.08/δ_C_ 90.7 in zoanthenamine) in the 1D NMR spectrum. This result indicated that the double bond C=CH between C-10 and C-11 was replaced by the CH–CH_2_, supported by the COSY cross-peaks between H-10 and H_2_-11 (Figure 6). Comparable NOESY correlations (Figure 7) required that **11** and zoanthenamine (CCDC 2046949)^20^ share a common relative configuration. In addition, the NOESY correlations between H-10 and Me-29 (δ_H_ 1.05) indicated that H-10 and Me-29 should be β-oriented. Accordingly, the structure of **11** was identified as shown and was named kuroshine P.

**Table 3:** ^1^H and ^13^C{^1^H} NMR Data for **11–14** in C_5_D_5_N^a^

	**11**^b^		**12**^b^		**13**^b^		**14**^c^	
no.	δ_H_	δ_c_, type	δ_H_	δ_c_, type	δ_H_	δ_c_, type	δ_H_	δ_c_, type
1	2.64, d (8.8)	56.2, CH_2_		172.7, C		183.2, C	3.08, m	50.9, CH_2_
	2.59, dd (8.8, 6.7)							
2	4.38, m	73.7, CH	4.56, m	76.9, CH	4.70, m	77.0, CH	4.59, m	73.8, CH
3	1.43, m	38.3, CH_2_	1.91, m	33.6, CH_2_	1.92, m	32.5, CH_2_	1.48, m	38.4, CH_2_
	1.30, m		1.41, m		1.37, m		1.39, m	
4	1.94, m	23.8, CH	1.88, m	24.4, CH	1.86, m	24.7, CH	1.71, m	23.5, CH
5	2.22, m	44.1, CH_2_	2.04, dd (14.0, 5.3)	38.2, CH_2_	2.05, dd (13.6, 5.5)	37.1, CH_2_	1.83, m	44.5, CH_2_
	0.98, m		1.07, m		1.01, m		1.03, m	
6		95.5, C		92.6, C		94.9, C		90.0, C
7	2.01, m	28.4, CH_2_	2.16, m	30.1, CH_2_	2.18, m	30.1, CH_2_	2.06, m	30.1, CH_2_
	1.69, m		1.86, m		1.91, m		1.82, m	
8	1.70, m	27.1, CH_2_	1.92, m	25.5, CH_2_	1.95, m	25.6, CH_2_	1.84, m	25.3, CH_2_
	1.47, m		1.68, m		1.70, m		1.58, m	
9		41.1, C		41.6, C		41.9, C		41.6, C
10	2.70, d (5.9)	61.3, CH		135.1, C		114.8, C		144.4, C
11	1.69, m	26.1, CH	6.72, s	113.1, CH		172.4, C	4.08, s	90.3, CH
	1.59, dd (16.1, 6.5)							
12		45.0, C		48.7, C		51.9, C		48.9, C
13	2.02, m	45.5, CH	2.36, m	44.7, CH	2.58, m	46.2, CH	2.18, dt (11.2, 4.1)	49.7, CH
14	2.25, m	31.5, CH_2_	2.67, m	32.4, CH_2_	2.74, d (8.0)	35.0, CH_2_	2.47, d (3.9)	33.5, CH_2_
			2.35, m				2.41, m	
15		160.1, C		159.9, C		161.2, C		160.3, C
16	6.02, s	127.0, CH	5.96, s	127.0, CH	5.96, s	126.4, CH	6.04, s	126.2, CH
17		199.3, C		198.8, C		198.6, C		200.1, C
18	2.78, dd (13.6, 5.5)	49.0, CH	2.82, dd (13.1, 5.3)	48.8, CH	2.88, dd (13.2, 5.6)	48.8, CH	2.80, m	46.4, CH
19	2.96, dq (6.8, 5.5)	43.5, CH	2.96, qd (6.8, 5.3)	44.2, CH	2.99, dq (6.8, 5.6)	44.1, CH	2.74, m	42.6, CH_2_
							2.48, m	
20		111.8, C		112.2, C		111.8, C		109.1, C
21	2.11, s	46.4, CH	2.49, s	43.7, CH	2.61, s	45.6, CH	2.30, s	50.9, CH
22		43.8, C		45.3, C		45.6, C		45.5, C
23	4.49, d (19.4)	35.2, CH_2_	4.21, d (18.1)	34.9, CH_2_	4.13, d (18.4)	34.6, CH_2_	4.24, d (18.5)	34.8, CH_2_
	3.90, d (19.4)		2.99, d (18.1)		3.15, d (18.4)		2.80, d (18.5)	
24		180.4, C		178.2, C		178.3, C		178.7, C
25	4.81, d (8.9)	76.0, CH_2_	5.55, d (9.3)	72.3, CH_2_	5.54, d (9.2)	71.9, CH_2_	5.59, d (9.1)	72.8, CH_2_
	4.54, d (8.9)		4.56, d (9.3)		4.53, d (9.2)		4.47, d (9.1)	
26	1.37, d (6.8)	12.9, CH_3_	1.41, d (6.8)	13.2, CH_3_	1.41, d (6.8)	13.5, CH_3_		
27	1.89, s	24.2, CH_3_	1.75, s	24.0, CH_3_	1.73, s	24.2, CH_3_	1.88, s	23.9, CH_3_
28	4.26, d (8.6)	68.9, CH_2_	4.16, d (8.9)	71.4, CH_2_	4.43, d (9.1)	70.6, CH_2_	4.27, d (8.8)	74.4, CH_2_
	4.00, d (8.6)		3.70, d (8.9)		4.21, d (9.1)		3.73, d (8.8)	
29	1.05, s	23.6, CH_3_	1.39, s	23.2, CH_3_	1.38, s	22.2, CH_3_	1.29, s	25.4, CH_3_
30	0.86, d (6.7)	21.8, CH_3_	0.78, d (6.4)	21.2, CH_3_	0.69, d (6.5)	20.9, CH_3_	0.75, d (6.4)	21.7, CH_3_

a Chemical shifts are in ppm. *J* values in Hz are in parentheses.

b ^1^H and ^13^C{^1^H} NMR were recorded at 600 and 150 MHz, respectively.

c ^1^H and ^13^C{^1^H} NMR were recorded at 400 and 100 MHz, respectively.

1-Keto-zoanthenamine (**12**) was shown to have the molecular formula C_30_H_37_NO_7_ by HRESIMS ([M + Na]^+^*m*/*z* 546.2462, calcd 546.2462) and ^13^C{^1^H} NMR data (Table 3). Its 1D NMR data (Table 3) indicated that its structure was similar to those of zoanthenamine, but the methylene at C-1 in zoanthenamine was oxidized to form a keto group (δ_C_ 172.7) in **12**. This finding was proven by the HMBC correlation (Figure 6) from H_2_-3 (δ_H_ 1.91 and 1.41) to C-1 (δ_C_ 172.7). Furthermore, the NOESY correlations (Figure 7) of H-21 (δ_H_ 2.49) with H-13 (δ_H_ 2.36), H_2_-25 (δ_H_ 5.55 and 4.56), Me-26 (δ_H_ 1.41), and Me-29 (δ_H_ 1.39) demonstrated the β-orientation of these protons. On the other hand, the NOESY correlations (Figure 7) of H-18 (δ_H_ 2.82) with H-19 (δ_H_ 2.96) and H_2_-28 (δ_H_ 4.16 and 3.70) revealed these protons should be α-orientated. The results of the NOESY experiment were similar to that of zoanthenamine, in which the absolute configuration was confirmed by single-crystal X-ray analysis,^21^ suggesting that the configurations of **12** and zoanthenamine were the same. Therefore, the structure of 1-keto-zoanthenamine (**12**) was determined.

A molecular formula of C_30_H_37_NO_8_ was determined for compound **13** from HRESIMS ([M + Na]^+^*m*/*z* 562.2418, calcd 562.2411), indicating that it was 16 mass units more than that of **12**. Analysis of the NMR data (Table 3) of **13** showed a close similarity to **12**. The only noticeable difference between **12** and **13** was that a hydroxy group at C-11 in **13** was supported by the HMBC correlations (Figure 6) from H_2_-28 (δ_H_ 4.43 and 4.21) to C-11 (δ_C_ 172.4). This assumption also agreed with the mass data of **13**. The configurations of **13** were determined to be the same as those of **12** based on their key NOESY correlations (Figure 7). Thus, the structure of **13** was assigned as 1-keto-11-hydroxyzoanthenamine.

The HRESIMS data ([M + H]^+^*m*/*z* 496.2694, calcd 496.2694) of **14** exhibited a protonated molecule consistent with a molecular formula of C_29_H_27_NO_6_. The ^1^H and ^13^C{^1^H} NMR data of **14** (Table 3) closely resembled those of zoanthenamine, except that the absence of a characteristic methyl doublet signal (C-26) and a methine signal (C-19) was altered to methylene (δ_H_ 2.74 and 2.48, δ_C_ 42.6). The assignment above was corroborated by the COSY cross-peak (Figure 6) between H-18 (δ_H_ 2.80)/H_2_-19 and the HMBC correlations (Figure 6) from H_2_-19 to C-20 (δ_C_ 109.1) and C-21 (δ_C_ 50.9). The key NOESY correlations (Figure 7) between **14** and zoanthenamine suggested they share the same relative configurations at the ten stereocenters. Accordingly, compound **14** was elucidated as 26-norzoanthenamine.

**Scheme 1:**
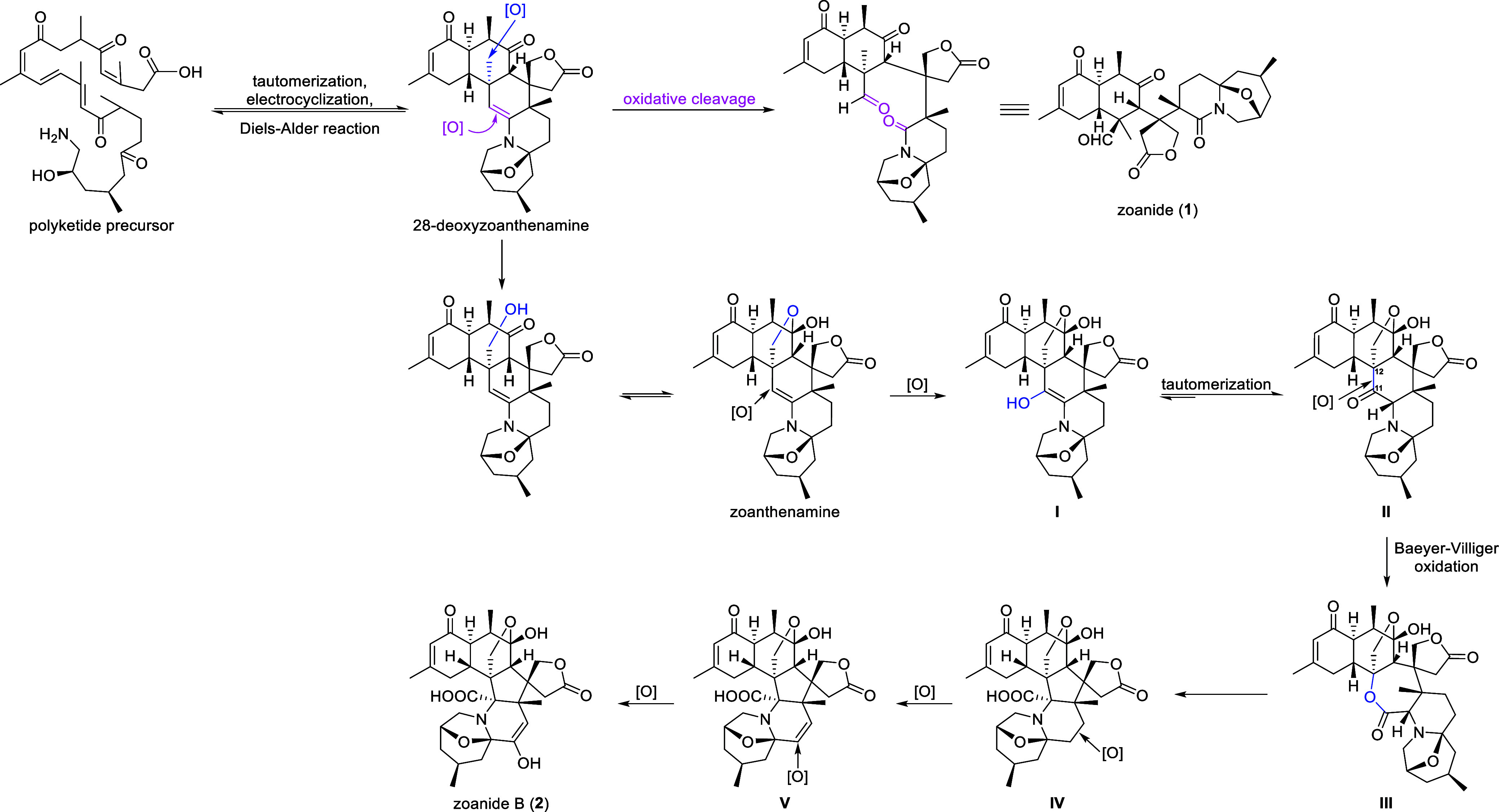
Proposed Biosynthetic Pathways for **1** and **2**

Structurally, compounds **1** and **2** have piqued our interest due to their unprecedented ring systems. The hypothetical biosynthetic pathways for **1** and **2** were proposed based on 28-deoxyzoanthenamine^20^ and zoanthenamine^20^ isolated from the same animal material, respectively. The oxidative cleavage of 28-deoxyzoanthenamine resulted in compound **1** (Scheme 1). As shown in Scheme 1, intermediate **II** is generated from zoanthenamine by oxidation and tautomerization. The critical intermediate **III** could be formed by the Baeyer–Villiger oxidation between C-11 and C-12. Then, intermediate **IV** might be produced via the ring contraction from **III**. Finally, zoanide (**2**) is produced through oxidation reactions of intermediate **V**.

These secondary metabolites have unique structures that may confer special bioactivity. Four major alkaloids (zoanthamine, norzoanthamine, zoanthenamine, and 28-deoxyzoanthenamine) isolated from *Z. vietnamensis* have been shown to exhibit neuroprotective effects against paclitaxel-induced peripheral neuropathy (PIPN).^20^ Therefore, a series of zoanthamine derivatives with similar core moieties were investigated for the potential to develop into neuroprotective agents.

Here, compounds **1**–**16** were evaluated for the neuroprotective potential of ameliorating paclitaxel-induced neurotoxicity. Results from cell viability assays on ND7/23 DRG neurons demonstrated that these compounds were non-neurotoxic below 10 μM, with cell viabilities remaining higher than 85% after treatment for 72 h (Table S22). One of the necessities of developing the neuroprotective agent for ameliorating PIPN is avoiding the interference of the anticancer efficacy of paclitaxel. Accordingly, to evaluate the interference of compounds **1**–**16** to the cytotoxicity of paclitaxel on cancer cells, we measured the viability of human cervical cancer SiHa cells with cotreatment of compounds **1**–**16** and paclitaxel. The viability of SiHa cells was significantly reduced to approximately 40% with the treatment of paclitaxel at 10 nM, whereas the cotreatment of paclitaxel with most testing compounds (1 and 10 μM) did not alter the anticancer efficacy (Table S23).

**Figure 8:**
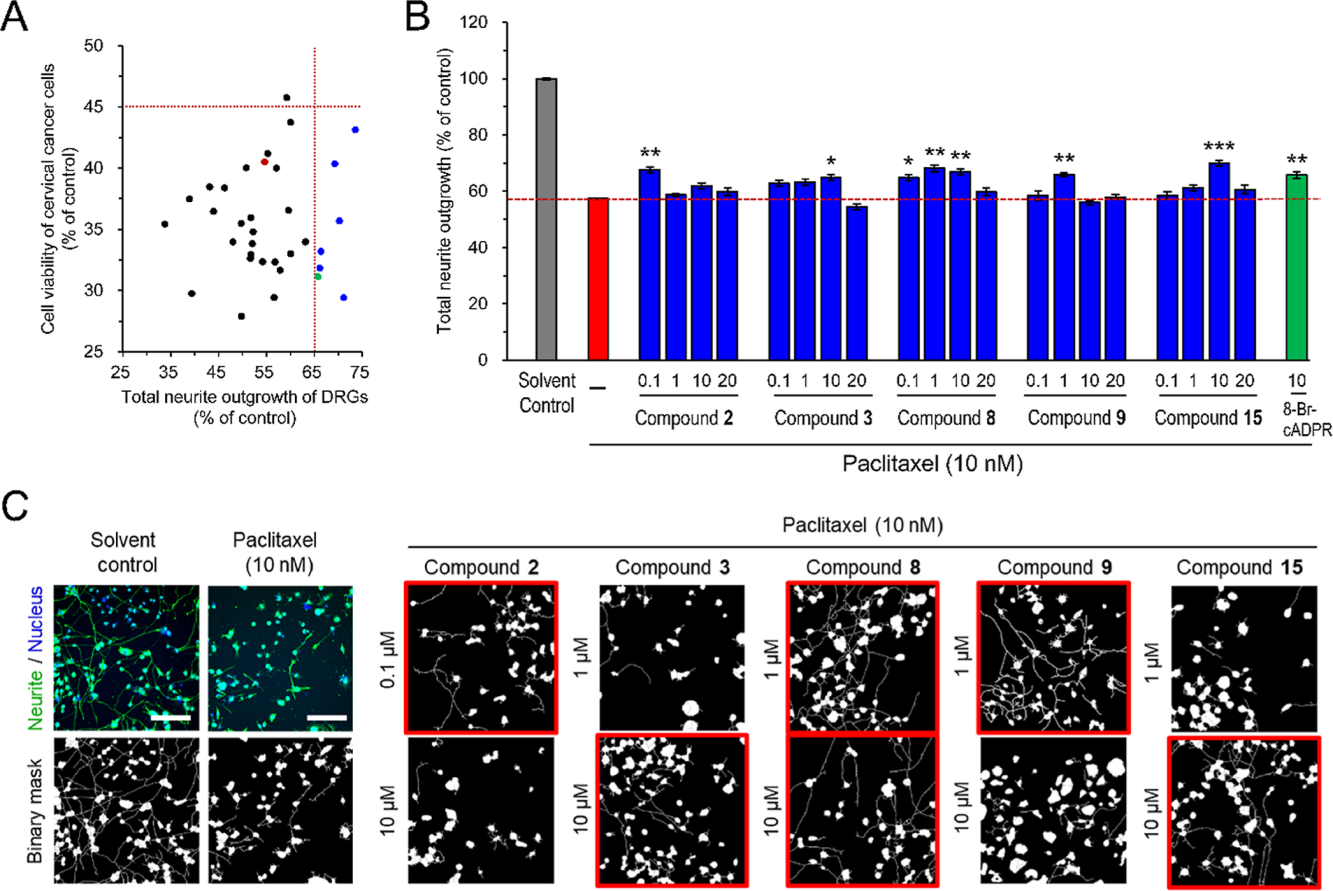
Candidate compounds showed significant neuroprotective effects against paclitaxel-induced neurotoxicity in ND7/23 DRG neurons. The well-differentiated ND7/23 DRG neurons or cervical cancer SiHa cells were pretreated with or without compounds **1**–**16** or the reference control 8-Br-cADPR (10 μM) for 1 day and then cotreated with paclitaxel (10 nM) for another 2 days. (A) Selection criteria for neuroprotective candidates. *X*-axis showed neuroprotective efficacy measured as the percent of control neurite outgrowth in DRG neurons; *Y*-axis showed maintenance of paclitaxel’s anticancer activity measured as percent viability in SiHa cells. Ideal candidates (blue dots) demonstrated significant neuroprotection (>65% neurite outgrowth) while preserving paclitaxel’s cytotoxicity (<45% cancer cell viability). Red dot: paclitaxel-only control; green dot: 8-Br-cADPR reference compound. (B) Neuroprotective evaluation of compounds **1**–**16** was assayed with quantitative analyses of the total neurite outgrowth in well-differentiated ND7/23 DRG neurons. Data were presented as the mean ± SEM from at least 48 views. *P < 0.05, **P < 0.01; ***P < 0.001 versus paclitaxel group, by Student’s *t*-test or one-way ANOVA post Tukey, Games Howell or Dunn’s. (C) Representative high-content images of well-differentiated ND7/23 DRG neurons for neuroprotective evaluation. Binary masks representing the area of neuronal cell bodies and extending neurites were generated by the analytic module of neurite outgrowth analyses. Green, neurofilament H staining neurite. Blue, 7-AAD staining nucleus. Red boxes highlighted conditions showing significant protection against paclitaxel-induced neurite damage. Scale bar, 200 μm.

Axon damage and degeneration are considered important mechanisms underlying the pathogenesis of PIPN.^27^ Thus, we further investigated the neuroprotective effect of compounds **1**–**16** under the non-neurotoxic concentration on morphological changes in the neurite network by the previously established robust and efficient high-content image screening platform based on the well-differentiated DRG neurons.^28^ 8-Br-cADPR, a known neuroprotective agent against paclitaxel-induced neurotoxicity,^29^ was used as a reference control. The primary screen showed that while the treatment with 10 nM paclitaxel drastically decreased the total neurite outgrowth of well-differentiated ND7/23 DRG neurons to approximately 54%, the pretreatment with compounds **1**–**16** at concentrations of 1 and 10 μM exhibited various effects (Table S23 and Figure 8A). Subsequently, we categorized the compounds showing significant protection on paclitaxel-induced neurite damage (i.e., with the total neurite outgrowth of DRG neurons higher than 65%) without compromising the anticancer activity (i.e., with the cell viability of cervical cancer cells lower than 45%) as potential neuroprotective candidates (Figure 8A). Based on these criteria, compounds **2**, **3**, **8**, **9**, and **15** (Figure 8A, blue dots) that demonstrated significant protection against paclitaxel-induced neurite damage without compromising anticancer activity were selected for the secondary assay with a more extensive concentration range. The treatment of **2**, **3**, **8**, **9**, and **15** at the expanded concentration range of 0.1–20 μM did not further emphasize the paclitaxel-induced neurite damage. The neuroprotective effects of compounds **2**, **3**, **8**, **9**, and **15** showed distinct concentration–response patterns. Compound **2** exhibited protection at 0.1 μM, restoring neurite outgrowth to approximately 67% of control levels. Compound **8** demonstrated broader effective concentration range (0.1–10 μM), maintaining consistent protection across these concentrations (approximately 64–66% of control). Compounds **3** and **15** required higher concentrations (10 μM) for significant protection, while compound **9** showed optimal effects at 1 μM. Importantly, these protective effects plateaued at higher concentrations without further enhancement, suggesting specific target engagement thresholds rather than nonspecific effects (Figures 8B,C). Taken together, the neuroprotection of these compounds to maintain significant neurite integrity while preserving chemotherapeutic efficacy established the foundation for developing into neuroprotective agents against PIPN.

**Figure 9:**
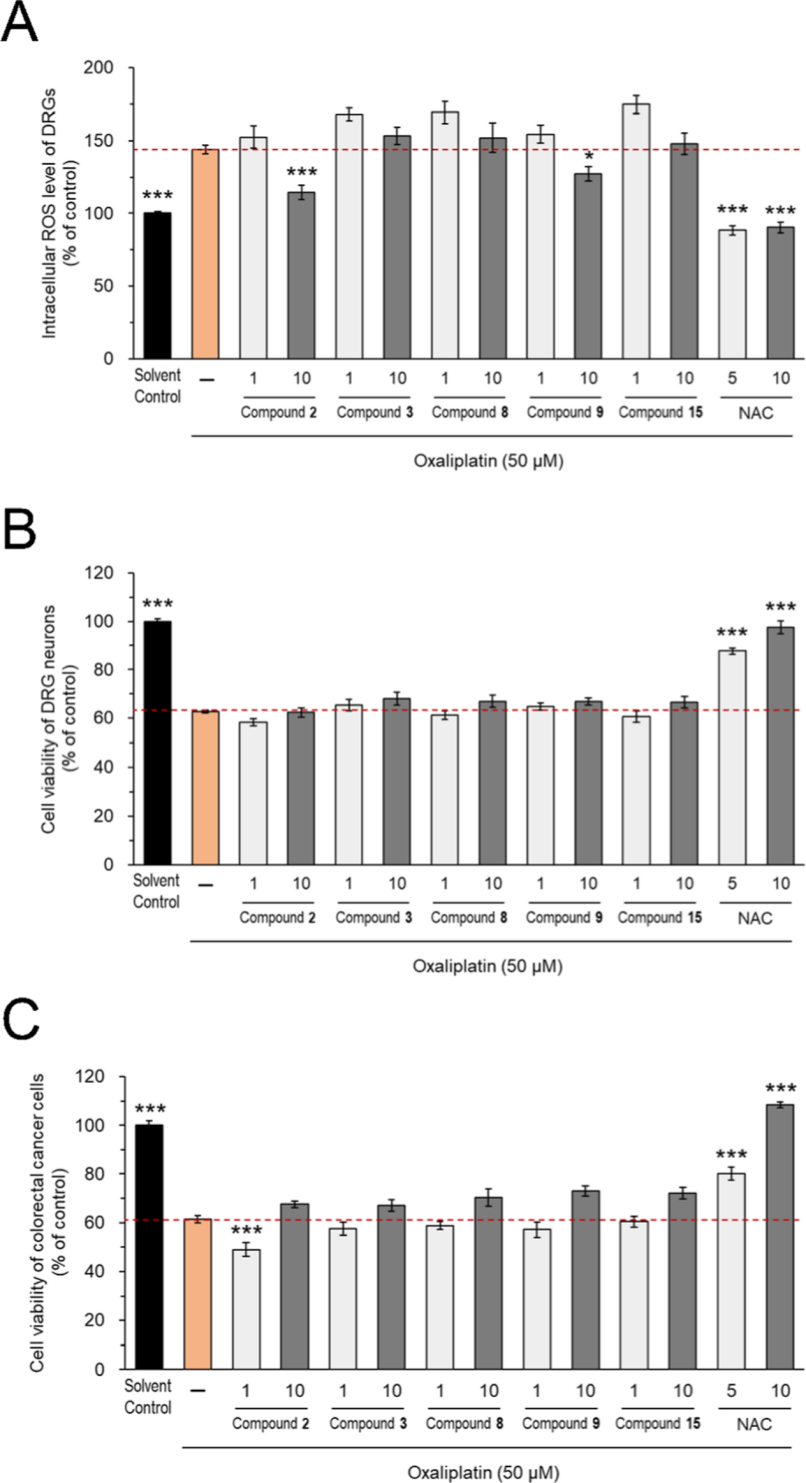
Candidate compounds showed antioxidant potentials against oxaliplatin-induced oxidative stress in ND7/23 DRG neurons. ND7/23 DRG neurons and colorectal cancer HT-29 cells were pretreated with or without compounds **2**, **3**, **8**, **9**, **15** (1 or 10 μM) or the reference control *N*-acetylcysteine (NAC) (5 and 10 mM) for 1 day and then cotreated with oxaliplatin (50 μM) for another 1 day. Intracellular reactive oxygen species (ROS) levels (A) and cell viability of DRG neurons (B), as well as the cell viability of colorectal cancer cells (C), were analyzed. Data were presented as the mean ± SEM from at least 21 wells from three independent experiments. *P < 0.05, ***P < 0.001 versus oxaliplatin group, by Student’s *t*-test or one-way ANOVA post Tukey, Games Howell, or Dunn’s.

Excessive production of reactive oxygen species (ROS), oxidative stress, and subsequent DNA damage are the main mechanisms involved in oxaliplatin-induced neuronal damage.^28^ For those neuroprotective compounds **2**, **3**, **8**, **9**, and **15**, we also explored their potentials against oxaliplatin-induced peripheral neuropathy (OIPN) by analyzing the intracellular ROS level and cell viability in oxaliplatin-treated ND7/23 DRG neurons. *N*-acetylcysteine (NAC), a well-known antioxidant and ROS scavenger,^29^ was used as a reference control. As shown in Figure 9A,B, 50 μM oxaliplatin caused ∼150% of intracellular ROS overproduction accompanied by the inhibition of ∼40% DRG cell viability, as compared with the control groups. Pretreatment with NAC at 5 and 10 mM in oxaliplatin-treated ND7/23 DRG neurons significantly ameliorated intracellular ROS overproduction and neuronal viability to the level of control DRG neurons (Figure 9A,B), but compromised the oxaliplatin-induced cytotoxicity in human colorectal cancer HT-29 cells (Figure 9C). Pretreatment with compounds **2** and **9** at 10 μM ameliorated oxaliplatin-induced intracellular ROS overproduction in ND7/23 DRG neurons without exaggerating the oxaliplatin-induced neuronal viability loss or compromising the anticancer effects of oxaliplatin in human colorectal cancer HT-29 cells (Figure 9A–C). Taking together the results from neuroprotective assays of PIPN and OIPN, we suggested that compounds **2**, **3**, **8**, **9**, and **15**, particularly compounds **2** and **9**, demonstrated dual protective actions, including preserving neurite integrity in paclitaxel-treated neurons and providing moderate protection against oxaliplatin-induced oxidative stress while importantly maintaining the anticancer efficacy of both chemotherapeutic agents.

## Conclusions

As described in this study, 16 novel zoanthamine-derived alkaloids (**1**–**16**) and two known compounds (**15** and **16**) were isolated from *Z. vietnamensis*, and their in vitro antioxidant and neuroprotective activity was assessed. Architecturally, compound **2** had a distinctive 6/6/5/6/7-fused pentacyclic carbon skeleton, and its biosynthesis process might be significantly impacted by the Baeyer–Villiger oxidation; compounds **7** and **8** are precursors for compounds with an ether linkage between C-2 and C-6. In the bioactive assay, compounds **2**, **3**, **8**, **9**, and **15** represent potential neuroprotective candidates for treating paclitaxel-induced peripheral neuropathy (PIPN). Additionally, compounds **2** and **9** showed moderate protective effects against oxaliplatin-induced oxidative stress, suggesting potential utility against both PIPN and OIPN through distinct mechanisms. The novel and complicated structure will add to the diversity of zoanthamine alkaloids, and their potent bioactivity could attract the curiosity of pharmacologists and synthetic chemists, which may aid in lead compound development.

## Experimental Section

### General Methods

Melting points were measured on a Stanford Research Systems (Sunnyvale, CA, US) OptiMelt automated melting point system. Specific rotations were obtained on a Jasco (Tokyo, Japan) P-2000 digital polarimeter. UV spectra were recorded on a Jasco V-650 UV–vis double-beam spectrophotometer. IR spectra were acquired on a Jasco FT-IR 4100 spectrometer using KBr plates. NMR spectra were collected on Varian (Palo Alto, CA, US) MR-400 MHz and VNMRS 600 MHz spectrometers with C_5_D_5_N (δ_C_ 123.5 and δ_H_ 7.21) as an internal standard. Structural assignments were made with additional information from gNOESY, gCOSY, gHSQC, and gHMBC experiments. HRESIMS data were obtained on a Bruker (Billerica, MA, US) 7T solariX spectrometer or a Thermo (Waltham, MA, US) Scientific Q Exactive Focus Orbitrap LC–MS/MS System. Merck (Darmstadt, Germany) silica gel 60 (230–400 mesh) and C_18_ silica gel (200–400 mesh) were used for column chromatography. Normal phase semipreparative HPLC was carried out using a Hitachi (Tokyo, Japan) L-6000 pump system equipped with a Hitachi L-4000 UV detector. Reverse-phase semipreparative HPLC was performed using a Shimadzu (Kyoto, Japan) CBM-20A system controller, a FRC-10A fraction collector, a LC-20AT pump, and a SPD-M20A PDA detector. Phenomenex (Torrance, CA, USA) Luna CN (5 μm, 250 × 10 mm), phenyl-hexyl (5 μm, 250 × 10 mm), silica (5 μm, 250 × 10 mm), Kinetex biphenyl (5 μm, 250 × 10 mm), and F_5_ (5 μm, 250 × 10 mm) columns were used for HPLC.

### Animal Material

The marine zoanthid was collected from the northern coastal area of Taiwan in April 2017 and identified as *Z. vietnamensis*. A voucher sample (code no. NSYSU-ZV) has been deposited in the Department of Marine Biotechnology and Resources, National Sun Yat-sen University, Kaohsiung, Taiwan.

### Extraction and Isolation

The zoanthid (approximately 1.1 kg, dry weight) was extracted repetitively with 95% EtOH to yield a crude extract (248.2 g). A portion of the ethanolic extract was fractionated by a silica gel open column with a stepwise solvent system of increasing polarity, starting from hexanes/EtOAc/MeOH 3/1/0 to 0/4/1, to produce nine fractions (A–I). The D fraction (6.4 g) was then chromatographed on a silica gel column (hexanes/CH_2_Cl_2_/MeOH, 1/0/0 to 0/30/1) to afford five fractions (D-1 to D-5). Fraction D-2 (2.9 g) was applied on a C_18_ open column (H_2_O/MeOH, 1/0 to 0/1) to furnish five fractions (D-2-1 to D-2-5). D-2-1 (201.6 mg) was isolated by NP-HPLC (CN column, flow = 2.0 mL/min, *n*-hexane/CH_2_Cl_2_/MeOH = 65/30/5, isocratic) to obtain **1** (19.9 mg). D-2-3 (898.5 mg) was subjected to a silica gel column using a step gradient elution of hexanes/EtOAc/acetone (3/1/0 to 0/0/1) to give five fractions, including **4** (D-2-3-3, 72.0 mg). D-2-3-4 (651.9 mg) was separated by a silica column (CH_2_Cl_2_/MeOH, 100/1 to 10/1) to get four fractions (D-2-3-4-1 to D-2-3-4-4). D-2-3-4-2 (25.3 mg) was further purified by semipreparative NP-HPLC (silica column, flow = 2.0 mL/min, *n*-hexane/CH_2_Cl_2_/MeOH = 30/10/1, isocratic) to afford **3** (1.5 mg) and **15** (1.0 mg). D-2-3-4-3 (202.6 mg) was chromatographed over a silica column (CH_2_Cl_2_/acetone, 100/1 to 0/1) to yield four fractions (D-2-3-4-3-1 to D-2-3-4-3-4). D-2-3-4-3-1 (31.9 mg) was purified by RP-HPLC (phenyl-hexyl column, flow = 2.0 mL/min, gradient program: 0–60 min, 30–100% MeCN_(aq)_) to afford **8** (2.5 mg). D-2-5 (781.1 mg) was separated by a silica column using hexanes/acetone/MeOH (5/1/0 to 0/0/1) to get four fractions (D-2-5-1 to D-2-5-4). D-2-5-2 (319.8 mg) was further fractionated on a silica column with a gradient elution of hexanes/EtOAc/acetone (3/1/0 to 0/0/1) to provide three fractions (D-2-5-2-1 to D-2-5-2-3). D-2-5-2-2 (56.1 mg) was separated by repeated RP-HPLC (phenyl-hexyl and CN column, flow = 2.0 mL/min, 25% MeCN_(aq)_, isocratic) to obtain **10** (1.1 mg). D-2-5-2-2-3 (19.5 mg) was purified by repeated RP-HPLC (CN column, flow = 2.0 mL/min, gradient program: 0–60 min, 20–100% MeCN_(aq)_; F_5_ column, flow = 2.0 mL/min, 55% MeCN_(aq)_, isocratic) to afford **7** (1.4 mg). D-2-5-3 (142.3 mg) was subjected to a silica gel column (hexanes/EtOAc/acetone, 1/1/0 to 0/0/1), followed by RP-HPLC separation (phenyl-hexyl column, flow = 2.0 mL/min, 50% MeCN_(aq)_, isocratic) to yield **13** (1.0 mg). Compound **9** (2.5 mg) was isolated from D-2–5–4 (87.6 mg) by RP-HPLC (phenyl-hexyl column, flow = 2.0 mL/min, gradient program: 0–60 min, 25–100% MeCN_(aq)_). D-3 (2.5 g) was fractionated by a C_18_ column (H_2_O/MeOH, 1/0 to 0/1) to give five fractions (D-3-1 to D-3-5). D-3-1 (173.8 mg) was chromatographed on a silica column (hexanes/acetone/MeOH, 15/1/0 to 0/5/1) to produce six fractions (D-3-1-1 to D-3-1-6). Separation of D-3-1-4 (5.2 mg) by RP-HPLC (phenyl-hexyl column, flow = 2.0 mL/min, 25% MeCN_(aq)_, isocratic) obtained **2** (1.4 mg). D-3-2 (134.9 mg) was separated using NP-HPLC (CN column, flow = 2.0 mL/min, *n*-hexane/CH_2_Cl_2_/MeOH = 65/30/5, isocratic) to furnish D-3-2-3 (17.6 mg) and **16** (22.4 mg). Compounds **5** (4.0 mg), **6** (1.3 mg), and **12** (1.0 mg) were isolated from D-3-2-3 by repeated RP-HPLC (phenyl-hexyl column, flow = 2.0 mL/min, 70% MeOH_(aq)_, isocratic; biphenyl column, flow = 2.0 mL/min, 70% MeCN_(aq)_, isocratic). D-3-3 (512.0 mg) was purified over silica open column using hexanes/EtOAc/acetone (2/1/0 to 0/0/1) stepwise gradient as eluents to yield three fractions (D-3-3-1 to D-3-3-3). D-3-3-2 (53.6 mg) was subjected to RP-HPLC (phenyl-hexyl column, flow = 2.0 mL/min, 25% MeCN_(aq)_, isocratic; CN column, flow = 2.0 mL/min, 20% MeCN_(aq)_, isocratic) to get **11** (1.2 mg). D-4 (322.2 mg) was fractionated on a silica column eluted with a gradient elution of CH_2_Cl_2_/acetone (70/1 to 0/1) to obtain six fractions (D-4-1 to D-4-6). D-4–4 (59.5 mg) was purified by semipreparative RP-HPLC (phenyl-hexyl column, flow = 2.0 mL/min, 25% MeCN_(aq)_, isocratic) to get **14** (2.5 mg).

Zoanide A (**1**): colorless acicular crystals; mp 182–184 °C; [α]_D_^25^ −8 (*c* 0.05, MeOH); UV (MeOH) λ_max_ (log ε) 233 (3.48) nm; IR (neat) ν_max_ 2952, 1776, 1712, 1663, 1622, 1415, 1379, 1185, 1014 cm^–1^; ^1^H and ^13^C{^1^H} NMR data see Table 1; HRMS (ESI) *m*/*z*: [M + Na]^+^ calcd for C_30_H_39_NO_7_Na, 548.2619; found, 548.2616.

Zoanide B (**2**): white amorphous powder; [α]_D_^25^ −8 (*c* 0.05, MeOH); UV (MeOH) λ_max_ (log ε) 236 (3.33) nm; IR (neat) ν_max_ 3394, 2924, 1772, 1651, 1459, 1378, 1290, 1201, 1076, 1026 cm^–1^; ^1^H and ^13^C{^1^H} NMR data see Table 1; HRMS (ESI) *m*/*z*: [M + Na]^+^ calcd for C_30_H_37_NO_9_Na, 578.2361; found, 578.2352.

(18*S*,21*S*)-Kuroshine E (**3**): colorless acicular crystals; mp 246–248 °C; [α]_D_^25^ −40 (*c* 0.05, MeOH); UV (MeOH) λ_max_ (log ε) 236 (4.06) nm; IR (neat) ν_max_ 2923, 1767, 1718, 1688, 1455, 1436, 1381, 1261, 1031, 1006 cm^–1^; ^1^H and ^13^C{^1^H} NMR data see Table 1; HRMS (ESI) *m*/*z*: [M + Na]^+^ calcd for C_30_H_37_NO_7_Na, 546.2462; found, 546.2465.

(18*S*,21*S*)-11-Dehydroxykuroshine A (**4**): yellow amorphous power; [α]_D_^25^ −114 (*c* 0.05, MeOH); UV (MeOH) λ_max_ (log ε) 237 (4.00) nm; IR (neat) ν_max_ 2950, 1775, 1714, 1666, 1436, 1379, 1280, 1190, 1026 cm^–1^; ^1^H and ^13^C{^1^H} NMR data see Table 1; HRMS (ESI) *m*/*z*: [M + Na]^+^ calcd for C_30_H_39_NO_6_Na, 532.2670; found, 532.2674.

1-Keto-kuroshine A (**5**): white amorphous powder; [α]_D_^25^ +37 (*c* 0.05, MeOH); UV (MeOH) λ_max_ (log ε) 236 (3.66) nm; IR (neat) ν_max_ 3432, 2921, 1770, 1693, 1659, 1394, 1205, 1146, 1041 cm^–1^; ^1^H and ^13^C{^1^H} NMR data see Table 1; HRMS (ESI) *m*/*z*: [M + Na]^+^ calcd for C_30_H_37_NO_8_Na, 562.2411; found, 562.2410.

1-Keto-11-dehydroxykuroshine A (**6**): white amorphous powder; [α]_D_^25^ +20 (*c* 0.05, MeOH); UV (MeOH) λ_max_ (log ε) 236 (3.39) nm; IR (neat) ν_max_ 2923, 1768, 1712, 1662, 1660, 1390, 1189, 1042 cm^–1^; ^1^H and ^13^C{^1^H} NMR data see Table 2; HRMS (ESI) *m*/*z*: [M + Na]^+^ calcd for C_30_H_37_NO_7_Na, 546.2462; found, 546.2464.

Kuroshine L (**7**): white amorphous powder; [α]_D_^25^ −80 (*c* 0.05, MeOH); UV (MeOH) λ_max_ (log ε) 235 (3.81) nm; IR (neat) ν_max_ 3503, 2959, 1769, 1718, 1669, 1435, 1380, 1264, 1188, 1030 cm^–1^; ^1^H and ^13^C{^1^H} NMR data see Table 2; HRMS (ESI) *m*/*z*: [M + Na]^+^ calcd for C_30_H_37_NO_7_Na, 546.2462; found, 546.2460.

Kuroshine M (**8**): brown oil; [α]_D_^25^ −64 (*c* 0.05, MeOH); UV (MeOH) λ_max_ (log ε) 235 (3.71) nm; IR (neat) ν_max_ 3444, 2924, 1769, 1711, 1667, 1456, 1435, 1382, 1204 cm^–1^; ^1^H and ^13^C{^1^H} NMR data see Table 2; HRMS (ESI) *m*/*z*: [M + Na]^+^ calcd for C_30_H_37_NO_7_Na, 546.2462; found, 546.2461.

Kuroshine N (**9**): white amorphous powder; [α]_D_^25^ +58 (*c* 0.05, MeOH); UV (MeOH) λ_max_ (log ε) 235 (3.93) nm; IR (neat) ν_max_ 3413, 2955, 1776, 1732, 1710, 1661, 1657, 1458, 1425, 1382, 1204, 1186, 1140, 1022 cm^–1^; ^1^H and ^13^C{^1^H} NMR data see Table 2; HRMS (ESI) *m*/*z*: [M + Na]^+^ calcd for C_30_H_37_NO_8_Na, 562.2411; found, 562.2407.

Kuroshine O (**10**): white amorphous powder; [α]_D_^25^ −11 (*c* 0.05, MeOH); UV (MeOH) λ_max_ (log ε) 242 (4.10) nm; IR (neat) ν_max_ 2962, 1770, 1710, 1669, 1637, 1402, 1261, 1095, 1029 cm^–1^; ^1^H and ^13^C{^1^H} NMR data see Table 2; HRMS (ESI) *m*/*z*: [M + Na]^+^ calcd for C_30_H_37_NO_6_Na, 530.2513; found, 530.2516.

Kuroshine P (**11**): white amorphous powder; [α]_D_^25^ +8 (*c* 0.05, MeOH); UV (MeOH) λ_max_ (log ε) 230 (3.26) nm; IR (neat) ν_max_ 3421, 2921, 1765, 1730, 1659, 1458, 1380, 1282, 1198, 1032 cm^–1^; ^1^H and ^13^C{^1^H} NMR data see Table 3; HRMS (ESI) *m*/*z*: [M + Na]^+^ calcd for C_30_H_41_NO_6_Na, 534.2826; found, 534.2824.

1-Keto-zoanthenamine (**12**): white amorphous powder; [α]_D_^25^ +68 (*c* 0.05, MeOH); UV (MeOH) λ_max_ (log ε) 235 (3.73) nm; IR (neat) ν_max_ 3413, 2925, 1771, 1710, 1661, 1399, 1266, 1197, 1029 cm^–1^; ^1^H and ^13^C{^1^H} NMR data see Table 3; HRMS (ESI) *m*/*z*: [M + Na]^+^ calcd for C_30_H_37_NO_7_Na, 546.2462; found, 546.2462.

1-Keto-11-hydroxyzoanthenamine (**13**): white amorphous powder; [α]_D_^25^ −66 (*c* 0.05, MeOH); UV (MeOH) λ_max_ (log ε) 233 (3.73) nm; IR (neat) ν_max_ 3426, 2923, 1771, 1683, 1388, 1204, 1137 cm^–1^; ^1^H and ^13^C{^1^H} NMR data see Table 3; HRMS (ESI) *m*/*z*: [M + Na]^+^ calcd for C_30_H_37_NO_8_Na, 562.2411; found, 562.2418.

26-Norzoanthenamine (**14**): brown oil; [α]_D_^25^ +21 (*c* 0.05, MeOH); UV (MeOH) λ_max_ (log ε) 232 (3.79) nm; IR (neat) ν_max_ 3440, 2922, 1767, 1663, 1381, 1200, 1024 cm^–1^; ^1^H and ^13^C{^1^H} NMR data see Table 3; HRMS (ESI) *m*/*z*: [M + H]^+^ calcd for C_29_H_38_NO_6_, 496.2694; found, 496.2694.

### X-ray Crystallographic Analyses

Colorless needle crystals of **1**, **3**, **16**, kuroshine E, and 28-deoxyzoanthenamine were obtained from a solvent mixture of MeOH and CH_2_Cl_2_ by slow evaporation at room temperature. The X-ray crystallographic data of these compounds were collected on Agilent Gemini single-crystal X-ray diffractometer and Bruker D8 Venture single-crystal X-ray diffractometer, respectively. Crystallographic data for **1**, **3**, **16**, kuroshine E, and 28-deoxyzoanthenamine have been deposited in the Cambridge Crystallographic Data Centre.

#### Crystallographic Data for **1**

C_30_H_39_NO_7_·2(CH_3_OH): C_32_H_47_NO_9_, *M* = 589.70, monoclinic, crystal size 0.35 × 0.25 × 0.20 mm^3^, space group P2_1_; unit cell dimensions *a* = 13.1044(6) Å, *b* = 7.6452(5) Å, *c* = 14.5480(6) Å, α = 90°, β = 97.943(4)°, γ = 90°, *V* = 1443.52(13) Å^3^; *Z* = 2; *d* = 1.357 Mg/m^3^, *T* = 100(2) K, μ (Cu Kα) = 0.806 mm^–1^. The total number of independent reflections measured was 12,830, of which 4992 were observed [*R*(int) = 0.0504]. Completeness to θ = 67.68°: 100.0%, absorption correction: semiempirical from equivalents. Max. and min transmission: 1.00000 and 0.62155. The final *R*_1_ value was 0.0471 [*I* > 2σ(*I*)], and *wR*_2_ value was 0.1321 (all data). The goodness of fit on *F*^2^ was 1.055. The absolute structure parameter is −0.12(11). CCDC number 2055085.

#### Crystallographic Data for **3**

C_30_H_37_NO_7_·Cl: C_30_H_37_ClNO_7_, *M* = 559.05, orthorhombic, crystal size 0.250 × 0.200 × 0.025 mm^3^, space group *P*2_1_2_1_2_1_; unit cell dimensions *a* = 7.2186(3) Å, *b* = 18.6256(8) Å, *c* = 43.1681(18) Å, α = β = γ = 90°, *V* = 5804.0(4) Å^3^; *Z* = 8; *d* = 1.280 Mg/m^3^, *T* = 100(2) K, μ (Cu Kα) = 1.552 mm^–1^. The total number of independent reflections measured was 60,135, of which 10,517 were observed [*R*(int) = 0.0391]. Completeness to θ = 67.68°: 99.2%, absorption correction: semiempirical from equivalents. Max. and min transmission: 1.0000 and 0.8516. The final *R*_1_ value was 0.0365 [*I* > 2σ(*I*)], and *wR*_2_ value was 0.1179 (all data). The goodness of fit on *F*^2^ was 1.041. The absolute structure parameter is 0.017(5). CCDC number 2206603.

#### Crystallographic Data for **16**

C_30_H_39_NO_7_, *M* = 525.62, monoclinic, crystal size 0.200 × 0.200 × 0.150 mm^3^, space group P2_**1**_; unit cell dimensions *a* = 8.2154(3) Å, *b* = 12.6152(5) Å, *c* = 12.7280(5) Å, α = 90°, β = 102.7039(11)°, γ = 90°, *V* = 1286.82(9) Å^3^; *Z* = 2; *d* = 1.357 Mg/m^3^, *T* = 100(2) K, μ (Cu Kα) = 0.781 mm^–1^. The total number of independent reflections measured was 30,452, of which 5265 were observed [*R*(int) = 0.0355]. Completeness to θ = 67.68°: 99.9%, absorption correction: semiempirical from equivalents. Max. and min transmission: 1.0000 and 0.8989. The final *R*_1_ value was 0.0484 [*I* > 2σ(*I*)], and *wR*_2_ value was 0.1547 (all data). The goodness of fit on *F*^2^ was 1.506. The absolute structure parameter is −0.08(13). CCDC number 2248823.

#### Crystallographic Data for Kuroshine E

C_30_H_37_NO_7_, *M* = 523.60, orthorhombic, crystal size 0.300 × 0.250 × 0.200 mm^3^, space group *P*2_1_2_1_2_1_; unit cell dimensions *a* = 7.7384(2) Å, *b* = 13.8193(4) Å, *c* = 23.7794(6) Å, α = β = γ = 90°, *V* = 2542.95(12) Å^3^; *Z* = 4; *d* = 1.368 Mg/m^3^, *T* = 100(2) K, μ (Cu Kα) = 0.790 mm^–1^. The total number of independent reflections measured was 34,805, of which 5332 were observed [*R*(int) = 0.0324]. Completeness to θ = 67.68°: 100.0%, absorption correction: semiempirical from equivalents. Max. and min transmission: 1.0000 and 0.8960. The final *R*_1_ value was 0.0310 [*I* > 2σ(*I*)], and *wR*_2_ value was 0.0896 (all data). The goodness of fit on *F*^2^ was 1.061. The absolute structure parameter is −0.03(15). CCDC number 2212110.

#### Crystallographic Data for 28-Deoxyzoanthenamine

C_30_H_39_NO_5_, *M* = 493.62, monoclinic, crystal size 0.200 × 0.150 × 0.100 mm^3^, space group P2_**1**_; unit cell dimensions *a* = 10.3705(4) Å, *b* = 10.0946(3) Å, *c* = 13.1069(4) Å, α = 90°, β = 111.0710(11)°, γ = 90°, *V* = 1280.36(7) Å^3^; *Z* = 2; *d* = 1.280 Mg/m^3^, *T* = 100(2) K, μ (Cu Kα) = 0.690 mm^–1^. The total number of independent reflections measured was 23,449, of which 5143 were observed [*R*(int) = 0.0369]. Completeness to θ = 67.68°: 98.9%, absorption correction: semiempirical from equivalents. Max. and min transmission: 1.0000 and 0.9019. The final *R*_1_ value was 0.0456 [*I* > 2σ(*I*)], and *wR*_2_ value was 0.1182 (all data). The goodness of fit on *F*^2^ was 1.016. The absolute structure parameter is −0.10(9). CCDC number 2212109.

### NMR Calculations

Conformational analyses were performed in the Spartan 20 program (Wave function Inc., Irvine, CA, USA) utilizing the Merck Molecular Force Field (MMFF) force field.^30,31^ These conformers within 40 kJ/mol of the lowest energy conformer were reoptimized at the B3LYP/6-31G(d,p) level in the gas phase and selected for ^1^H and ^13^C NMR computations at the mPW1PW91/6-311G(d,p) level with polarizable continuum model (PCM) in pyridine by Gaussian 16 program package (Gaussian Inc., Wallingford, CT, USA).^32^ All conformers used for calculations in this study were characterized as stable points on potential energy surfaces (PES) without imaginary frequencies. The calculated chemical shifts of each configuration were averaged according to their Boltzmann distributions. The experimental and Boltzmann-weighted NMR data were analyzed by the improved DP4+ probability^24^ method to determine the absolute configurations.

### Evaluation of Neuroprotective Effect Against Chemotherapy-Induced Neurotoxicity

#### Dorsal Root Ganglia (DRG) Neuron Cell Culture

ND7/23 cells, a fusion cell line of N18TG2 mouse neuroblastoma and rat dorsal root ganglia (DRG),^33^ were cultured in Dulbecco’s Modified Eagle Medium supplemented with 3700 mg/L sodium bicarbonate, 10% fetal bovine serum (FBS), 1% nonessential amino acids (NEAA) (Simply Biotech, California, U.S.A.), 100 U/mL penicillin, 100 μg/mL streptomycin, and 250 ng/mL amphotericin B. Cells were maintained at 37 °C in a humidified incubator with 5% CO_2_.

#### Neurotoxicity of Compounds **1**–**16** in ND7/23 DRG Neurons

The neurotoxicity of testing compounds on ND7/23 cells were analyzed by the resazurin-based cell viability assay as previously described.^28^ Briefly, ND7/23 cells were plated in the 96-well plate at the density of 500 cells/well for 24 h. ND7/23 cells were treated with or without testing compounds for 72 h. After treatment, 10 μL resazurin (0.2 mg/mL) (Cayman Chemical, Michigan, U.S.A.) was added into each well, and then the plate was incubated at 37 °C for 2 h. Fluorescence with an excitation wavelength of 530 nm and emission wavelength of 590 nm was analyzed by the SpectraMax iD3 multimode microplate reader (Molecular Devices, San Jose, U.S.A.). The fluorescent intensity was normalized to the control group. The treatment that reduced the viability of ND7/23 to 85% were considered neurotoxic.

#### Differentiation of ND7/23 DRG Neurons

The differentiation of ND7/23 cells into the mature DRG neurons was induced as previously described.^26^ Briefly, ND7/23 cells were first plated in the poly-l-lysine (Sigma-Aldrich, Saint Louis, U.S.A.)-coated 96-well thin-bottom optic microplate (Greiner Bio-One, Frickenhausen, Germany) at the cell density of 500 cells/well with regular culture medium for 24 h. The differentiation medium supplemented with 0.5% FBS, 100 ng/mL nerve growth factor (NGF) (Sigma-Aldrich, Saint Louis, U.S.A.), and 1 mM dibutyryl-cyclic adenosine monophosphate (db-cAMP) (Cayman Chemical, Michigan, U.S.A.) was then applied to induce the differentiation of ND7/23 cells, and then renewed every other day. After the 6 day induction, the maturely differentiated ND7/23 DRGs were subjected to neuroprotective assays.^28^

#### Neuroprotective Evaluation—Paclitaxel-Induced Neurite Damage

The neuroprotective potentials of testing compounds against paclitaxel-induced neurite damage was examined as previously described.^28^ Briefly, the well-differentiated ND7/23 DRG neuron cells were pretreated with or without testing compounds for 1 day and then treated with 10 nM paclitaxel for another 2 days. After treatment, cells were washed with phosphate-buffered saline (PBS) and then fixed with 4% paraformaldehyde (Acros, New Jersey, U.S.A.) for 15 min. Fixed cells were permeabilized with 0.05% Triton X-100 (Bio Basic, Markham, Canada) in PBS for 20 min, and then blocked overnight at 4 °C with Super Blocking Reagents (Thermo Scientific, Waltham, U.S.A.). Subsequently, ND7/23 cells were immunostained with rat antineurofilament H (MAB5448, Sigma-Aldrich, Saint Louis, U.S.A.) overnight at 4 °C, and then reacted with the Alexa Fluor 488 goat antirat secondary antibody (Jackson ImmunoResearch, West Grove, U.S.A.) and counterstained with a DNA binding dye, 7-aminoactinomycin D (7-AAD) (AAT Bioquest, California, U.S.A), at room temperature for 1 h. For image acquisition and analyses of neurite outgrowth, images of stained DRG neurons were automatedly acquired by the ImageXpress Micro Widefield High Content Screening System (Molecular Devices, San Jose, U.S.A.) with a 20× objective (numerical aperture value: 0.45). To evaluate the neuroprotective effect of testing compounds on neurite integrity, the total neurite outgrowth was analyzed by measuring the total length of neurite outgrowth in μm (corrected for diagonal lengths) associated with each cell body.^28^

#### Neuroprotective Evaluation—Oxaliplatin-Induced Oxidative Stress Overload

To investigate the antioxidant effect of testing compounds against oxaliplatin-induced oxidative stress overload, ND7/23 cells were plated in the 96-well plate at the density of 2500 cells/well for 24 h, pretreated with or without testing compounds for 24 h, and then treated with 50 μM oxaliplatin for another 24 h. After treatment, cells were washed with PBS, then stained by the superoxide indicator dihydroethidium (DHE) (Cayman Chemical, Michigan, U.S.A.) for 1 h.^34^ Fluorescence with an excitation wavelength of 520 nm and emission wavelength of 600 nm was analyzed by the SpectraMax iD3 multimode microplate reader. The relative fluorescent intensity of DHE compared to solvent control group was normalized against the cell viability.

### Interference with the Anticancer Effect of Paclitaxel and Oxaliplatin

#### Cancer Cell Culture

Human cervical cancer cell line SiHa (American Type Culture Collection, Virginia, U.S.A) was cultured in Dulbecco’s Modified Eagle Medium (Caisson Biotech, Texas, U.S.A.) supplemented with 3700 mg/L sodium bicarbonate (Bio Basic, Markham, Canada), 10% fetal bovine serum (FBS) (Sigma-Aldrich, Saint Louis, U.S.A.), 100 U/mL penicillin, 100 μg/mL streptomycin, and 250 ng/mL amphotericin B (Simply Biotech, California, U.S.A.). Human colorectal cancer cell line HT-29 (American Type Culture Collection, Virginia, U.S.A) were cultured in McCoy’s 5A Medium (HiMedia, Pennsylvania, U.S.A) supplemented with 2500 mg/L sodium bicarbonate, 10% FBS, 100 U/mL penicillin, 100 μg/mL streptomycin, and 250 ng/mL amphotericin B. Cells were maintained at 37 °C in a humidified incubator with 5% CO_2_.

#### Cytotoxicity of Compounds **1**–**16** in Cervical Cancer SiHa and Colorectal Cancer HT-29 Cells

The cell viability of SiHa and HT-29 cells was determined by the resazurin-based viability assay, as previously described.^18^ Cells were plated in the 96-well plate at the density of 2500 cells/well for 24 h, pretreated with or without testing compounds for 1 day, and then cotreated with 10 nM paclitaxel or 50 μM oxaliplatin for the indicated time. The cell viability was normalized to the solvent control group.

### Statistical Analyses

Data were expressed as the mean ± standard error of the mean (S.E.M.), while statistical analyses were calculated using Microsoft Excel 2019 with a free Excel add-in Real Statistics Resource Pack developed by Dr. Charles Zaiontz (Release 8.4).^35^ Differences between groups were compared using the Student’s *t*-test or one-way analysis of variance (ANOVA) followed by Dunnett, Tukey, or Games Howell post hoc analyses. The criterion for statistical significance was *P* < 0.05.

## Data Availability

The data underlying this study are available in the published article and its Supporting Information.

## Supporting Information Available

UV, IR, HRESIMS, 1D/2D NMR spectra for compounds **1**–**14**; computational calculation details of compounds **2**, **7**, and **8**; effects of compounds **1**–**16** on the cell viability of ND7/23 DRG neurons; neuroprotective potentials and anticancer interfering effects of compounds **1**–**16**; and the detailed X-ray crystallographic data of **1**, **3**, **16**, kuroshine E, and 28-deoxyzoanthenamine (PDF) X-ray crystallographic data of **1** (CIF) X-ray crystallographic data of **3** (CIF) X-ray crystallographic data of **16** (CIF) X-ray crystallographic data of kuroshine E (CIF) X-ray crystallographic data of 28-deoxyzoanthenamine (CIF) (PDF)

## References

[ref1] Guillen P. O.; Jaramillo K. B.; Genta-Jouve G.; Thomas O. P. Marine natural products from zoantharians: bioactivity, biosynthesis, systematics, and ecological roles. Nat. Prod. Rep. 2020, 37, 515–540. 10.1039/C9NP00043G. 31670367

[ref2] Kokke W. C. M. C.; Withers N. W.; Massey I. J.; Fenical W.; Djerassi C. Isolation and synthesis of 23-methyl-22-dehydrocholesterol-a marine sterol of biosynthetic significance. Tetrahedron Lett. 1979, 20, 3601–3604. 10.1016/S0040-4039(01)95474-7.

[ref3] Kelecom A. Studies of Brazilian marine invertebrates. VIII. Zoanthosterol, a new sterol from the zoanthid *Zoanthus sociatus* (hexacorallia, zoanthidea). Bull. Soc. Chim. Belg. 1981, 90, 971–976. 10.1002/bscb.19810900913.

[ref4] Suksamrarn A.; Jankam A.; Tarnchompoo B.; Putchakarn S. Ecdysteroids from a *Zoanthus* sp. J. Nat. Prod. 2002, 65, 1194–1197. 10.1021/np010645s. 12193031

[ref5] Cheng Y.-B.; Lee J.-C.; Lo I.-W.; Chen S.-R.; Hu H.-C.; Wu Y.-H.; Wu Y.-C.; Chang F.-R. Ecdysones from *Zoanthus* spp. with inhibitory activity against dengue virus 2. Bioorg. Med. Chem. Lett. 2016, 26, 2344–2348. 10.1016/j.bmcl.2016.03.029. 26988299

[ref6] Cariello L.; Crescenzi S.; Zanetti L.; Prota G. A survey on the distribution of zoanthoxanthins in some marine invertebrates. Comp. Biochem. Physiol. 1979, 63B, 77–82. 10.1016/0305-0491(79)90237-2.

[ref7] Behenna D. C.; Stockdill J. L.; Stoltz B. M. The biology and chemistry of the zoanthamine alkaloids. Angew. Chem., Int. Ed. 2008, 47, 2365–2386. 10.1002/anie.200703172. 18307180

[ref8] Guillen P. O.; Gegunde S.; Jaramillo K. B.; Alfonso A.; Calabro K.; Alonso E.; Rodriguez J.; Botana L. M.; Thomas O. P. Zoanthamine alkaloids from the zoantharian *Zoanthus* cf. *pulchellus* and their effects in neuroinflammation. Mar. Drugs 2018, 16, 242 10.3390/md16070242. 30036989 PMC6071026

[ref9] Yamaguchi K.; Yada M.; Tsuji T.; Kuramoto M.; Uemura D. Suppressive Effect of Norzoanthamine Hydrochloride on Experimental Osteoporosis in Ovariectomized Mice. Biol. Pharm. Bull. 1999, 22, 920–924. 10.1248/bpb.22.920. 10513613

[ref10] Chen S.-R.; Wang S.-W.; Sheu J.-H.; Chang T.-H.; Cheng Y.-B. Zoanthamine alkaloid derivatives from the zoantharian *Zoanthus vietnamensis* with antimetastatic activity. J. Org. Chem. 2020, 85, 12553–12560. 10.1021/acs.joc.0c01731. 32893629

[ref11] Yoshimura F.; Sasaki M.; Hattori I.; Komatsu K.; Sakai M.; Tanino K.; Miyashita M. Synthetic studies of the zoanthamine alkaloids: the total syntheses of norzoanthamine and zoanthamine. Chem.—Eur. J. 2009, 15, 6626–6644. 10.1002/chem.200900310. 19479925

[ref12] Yoshimura F.; Takahashi Y.; Tanino K.; Miyashita M. Synthetic studies of the zoanthamine alkaloids: total synthesis of zoanthenol based on an isoaromatization strategy. Chem.–Asian J. 2011, 6, 922–931. 10.1002/asia.201000552. 21344668

[ref13] Yoshimura F.; Tanino K.; Miyashita M. Total Synthesis of Zoanthamine Alkaloids. Acc. Chem. Res. 2012, 45, 746–755. 10.1021/ar200267a. 22340011

[ref14] Xin Z.; Wang H.; He H.; Zhao X.; Gao S. Asymmetric total synthesis of norzoanthamine. Angew. Chem., Int. Ed. 2021, 60, 12807–12812. 10.1002/anie.202102643. 33822444

[ref15] Chen Y.; Xin Z.; Wang H.; He H.; Gao S. Asymmetric total synthesis of norzoanthamine and formal synthesis of zoanthenol. Org. Chem. Front. 2023, 10, 651 10.1039/D2QO01834A.

[ref16] Loprinzi C. L.; Lacchetti C.; Bleeker J.; Cavaletti G.; Chauhan C.; Hertz D. L.; Kelley M. R.; Lavino A.; Lustberg M. B.; Paice J. A.; Schneider B. P.; Lavoie Smith E. M.; Smith M. L.; Smith T. J.; Wagner-Johnston N.; Hershman D. L. Prevention and management of chemotherapy-induced peripheral neuropathy in survivors of adult cancers: ASCO guideline update. J. Clin. Oncol. 2020, 38, 3325–3348. 10.1200/JCO.20.01399. 32663120

[ref17] Burgess J.; Ferdousi M.; Gosal D.; Boon C.; Matsumoto K.; Marshall A.; Mak T.; Marshall A.; Frank B.; Malik R.; Alam U. Chemotherapy-induced peripheral neuropathy: epidemiology, pathomechanisms and treatment. Oncol. Ther. 2021, 9, 385–450. 10.1007/s40487-021-00168-y. 34655433 PMC8593126

[ref18] Han Y.; Smith M. T. Pathobiology of cancer chemotherapy-induced peripheral neuropathy (CIPN). Front. Pharmacol 2013, 4, 156 10.3389/fphar.2013.00156. 24385965 PMC3866393

[ref19] Hsu Y.-M.; Chang F.-R.; Lo I.-W.; Lai K.-H.; El-Shazly M.; Wu T.-Y.; Du Y.-C.; Hwang T.-L.; Cheng Y.-B.; Wu Y.-C. Zoanthamine-type alkaloids from the zoanthid *Zoanthus kuroshio* collected in Taiwan and their effects on inflammation. J. Nat. Prod. 2016, 79, 2674–2680. 10.1021/acs.jnatprod.6b00625. 27759384

[ref20] Chen S.-R.; Wang S.-W.; Chang F.-R.; Cheng Y.-B. Anti-lymphangiogenic alkaloids from the zoanthid *Zoanthus vietnamensis* collected in Taiwan. J. Nat. Prod. 2019, 82, 2790–2799. 10.1021/acs.jnatprod.9b00451. 31584818

[ref21] Chen S.-R.; Wang S.-W.; Lin Y.-C.; Yu C.-L.; Yen J.-Y.; Chen Y.-F.; Cheng Y.-B. Additional alkaloids from *Zoanthus vietnamensis* with neuroprotective and anti-angiogenic effects. Bioorg. Chem. 2021, 109, 104700 10.1016/j.bioorg.2021.104700. 33607361

[ref22] Grimblat N.; Sarotti A. M. Computational chemistry to the rescue: modern toolboxes for the assignment of complex molecules by GIAO NMR calculations. Chem.—Eur. J. 2016, 22, 12246–12261. 10.1002/chem.201601150. 27405775

[ref23] Willoughby P. H.; Jansma M. J.; Hoye T. R. A guide to small-molecule structure assignment through computation of (^1^H and ^13^C) NMR chemical shifts. Nat. Protoc. 2014, 9, 643–660. 10.1038/nprot.2014.042. 24556787

[ref24] Grimblat N.; Zanardi M. M.; Sarotti A. M. Beyond DP4: an improved probability for the stereochemical assignment of isomeric compounds using quantum chemical calculations of NMR shifts. J. Org. Chem. 2015, 80, 12526–12534. 10.1021/acs.joc.5b02396. 26580165

[ref25] Cheng Y.-B.; Lo I.-W.; Shyur L.-F.; Yang C.-C.; Hsu Y.-M.; Su J.-H.; Lu M.-C.; Chiou S.-F.; Lan C.-C.; Wu Y.-C.; Chang F.-R. New alkaloids from Formosan zoanthid *Zoanthus kuroshio*. Tetrahedron 2015, 71, 8601–8606. 10.1016/j.tet.2015.09.023.

[ref26] Cheng Y.-B.; Lan C.-C.; Liu W.-C.; Lai W.-C.; Tsai Y.-C.; Chiang M.-Y.; Wu Y.-C.; Chang F.-R. Kuroshines A and B, new alkaloids from *Zoanthus kuroshio*. Tetrahedron Lett. 2014, 55, 5369–5372. 10.1016/j.tetlet.2014.07.101.

[ref27] Fukuda Y.; Li Y.; Segal R. A. A mechanistic understanding of axon degeneration in chemotherapy-induced peripheral neuropathy. Front. Neurosci. 2017, 11, 481 10.3389/fnins.2017.00481. 28912674 PMC5583221

[ref28] Chang Y.-C.; Lo Y.-C.; Chang H.-S.; Lin H.-C.; Chiu C.-C.; Chen Y.-F. An efficient cellular image-based platform for high-content screening of neuroprotective agents against chemotherapy-induced neuropathy. Neurotoxicology 2023, 96, 118–128. 10.1016/j.neuro.2023.04.007. 37086979

[ref29] Li Y.; Pazyra-Murphy M. F.; Avizonis D.; de Sá Tavares Russo M.; Tang S.; Chen C. Y.; Hsueh Y. P.; Bergholz J. S.; Jiang T.; Zhao J. J.; Zhu J.; Ko K. W.; Milbrandt J.; DiAntonio A.; Segal R. A. Sarm1 activation produces cADPR to increase intra-axonal Ca^+2^ and promote axon degeneration in PIPN. J. Cell Biol. 2022, 22, e202106080 10.1083/jcb.202106080. PMC8704956 34935867

[ref30] Kang L.; Tian Y.; Xu S.; Chen H. Oxaliplatin-induced peripheral neuropathy: clinical features, mechanisms, prevention and treatment. J. Neurol. 2021, 268, 3269–3282. 10.1007/s00415-020-09942-w. 32474658

[ref31] Dekhuijzen P. N. R. Antioxidant properties of N-acetylcysteine: their relevance in relation to chronic obstructive pulmonary disease. Eur. Respir. J. 2004, 23, 629–636. 10.1183/09031936.04.00016804. 15083766

[ref32] Frisch M. J.; Trucks G. W.; Schlegel H. B.; Scuseria G. E.; Robb M. A.; Cheeseman J. R.; Scalmani G.; Barone V.; Petersson G. A.; Nakatsuji H.; Li X.; Caricato M.; Marenich A. V.; Bloino J.; Janesko B. G.; Gomperts R.; Mennucci B.; Hratchian H. P.; Ortiz J. V.; Izmaylov A. F.; Sonnenberg J. L.; Williams-Young D.; Ding F.; Lipparini F.; Egidi F.; Goings J.; Peng B.; Petrone A.; Henderson T.; Ranasinghe D.; Zakrzewski V. G.; Gao J.; Rega N.; Zheng G.; Liang W.; Hada M.; Ehara M.; Toyota K.; Fukuda R.; Hasegawa J.; Ishida M.; Nakajima T.; Honda Y.; Kitao O.; Nakai H.; Vreven T.; Throssell K.; Montgomery J. A. J.; Peralta J. E.; Ogliaro F.; Bearpark M. J.; Heyd J. J.; Brothers E. N.; Kudin K. N.; Staroverov V. N.; Keith T. A.; Kobayashi R.; Normand J.; Raghavachari K.; Rendell A. P.; Burant J. C.; Iyengar S. S.; Tomasi J.; Cossi M.; Millam J. M.; Klene M.; Adamo C.; Cammi R.; Ochterski J. W.; Martin R. L.; Morokuma K.; Farkas O.; Foresman J. B.; Fox D. J. Gaussian 16. Revision C.01; Gaussian Inc.: Wallingford CT, 2016.

[ref33] Haberberger R. V.; Barry C.; Matusica D. Immortalized dorsal root ganglion neuron cell lines. Front. Cell. Neurosci. 2020, 14, 184 10.3389/fncel.2020.00184. 32636736 PMC7319018

[ref34] Zhao H.; Kalivendi S.; Zhang H.; Joseph J.; Nithipatikom K.; Vásquez-Vivar J.; Kalyanaraman B. Superoxide reacts with hydroethidine but forms a fluorescent product that is distinctly different from ethidium: potential implications in intracellular fluorescence detection of superoxide. Free Radic. Biol. Med. 2003, 34, 1359–1368. 10.1016/S0891-5849(03)00142-4. 12757846

[ref35] Zaiontz C. Real Statistics Resource Pack Software, Release 7.6. Copyright (2013–2021), 2020. www.real-statistics.com.

