# Supplementary material for: Discovery of Zoanthamine Alkaloids from Zoanthus vietnamensis with Antioxidant and Neuroprotective Activities
Source: J Org Chem. 2025 Apr 2;90(14):5019–35. doi: 10.1021/acs.joc.5c00280 (PMC11998011; doi:10.1021/acs.joc.5c00280)

Supporting Information

Discovery of Zoanthamine Alkaloids from *Zoanthus vietnamensis* with Antioxidant and Neuroprotective Activities

Shu-Rong Chen, Yang-Chen Chang, Yi Chen, Yih-Fung Chen, Yu-Chi Lin, Cheng-chau Chiu, and Yuan-Bin Cheng*

Corresponding Author

Yuan-Bin Cheng − Department of Marine Biotechnology and Resources, National Sun Yat-sen University, Kaohsiung 80424, Taiwan; orcid.org/0000-0001-6581-1320; E-mail: jmb@mail.nsysu.edu.tw; Tel: +886-7-5252-000-5212

Authors

Shu-Rong Chen − Department of Marine Biotechnology and Resources, National Sun Yat-sen University, Kaohsiung 80424, Taiwan

Yang-Chen Chang − Graduate Institute of Natural Products, College of Pharmacy, Kaohsiung Medical University, Kaohsiung 80708, Taiwan

Yi Chen − Department of Marine Biotechnology and Resources, National Sun Yat-sen University, Kaohsiung 80424, Taiwan

Yih-Fung Chen − Graduate Institute of Natural Products, College of Pharmacy, Kaohsiung Medical University, Kaohsiung 80708, Taiwan; School of Pharmacy, College of Pharmacy, Kaohsiung Medical University, Kaohsiung 80708, Taiwan

Yu-Chi Lin − National Research Institute of Chinese Medicine, Ministry of Health and Welfare, Taipei 11221, Taiwan

Cheng-chau Chiu − Department of Chemistry, National Sun Yat-sen University, Kaohsiung 80424, Taiwan

Table of Contents

Table S1. Energy analyses of 10*R*-**2** (four conformers)....S9
Table S2. Cartesian coordinates of the low-energy conformers of 10*R*-**2** re-optimized at B3LYP/6-31G(d,p) level. S10
Table S3. Energy analyses of 10*S*-**2** (three conformers)....S14
Table S4. Cartesian coordinates of the low-energy conformers of 10*S*-**2** re-optimized at B3LYP/6-31G(d,p) level....S15
Table S5. Experimental and calculated ^{1}H NMR data for compound **2**. S18
Table S6. Experimental and calculated ^{13}C{^{1}H} NMR data for compound **2**. S19
Table S7. DP4+ analyses of calculated and experimental NMR chemical shifts of **2** (unscaled). Isomer 1: 10*R*-**2**; Isomer 2: 10*S*-**2**. S20
Table S8. Energy analyses of 2*R*-**7** (four conformers)....S21
Table S9. Cartesian coordinates of the low-energy conformers of 2*R*-**7** re-optimized at B3LYP/6-31G(d,p) level. S22
Table S10. Energy analyses of 2*S*-**7** (one conformer)....S26
Table S11. Cartesian coordinates of the low-energy conformers of 2*S*-**7** re-optimized at B3LYP/6-31G(d,p) level....S27
Table S12. Experimental and calculated ^{1}H NMR data for compound **7**. S28
Table S13. Experimental and calculated ^{13}C{^{1}H} NMR data for compound **7**. S29
Table S14. DP4+ analyses of calculated and experimental NMR chemical shifts of **7** (unscaled). Isomer 1: 2*R*-**7**; Isomer 2: 2*S*-**7**. S30
Table S15. Energy analyses of 2*R*-**8** (four conformers)....S31
Table S16. Cartesian coordinates of the low-energy conformers of 2*R*-**8** re-optimized at B3LYP/6-31G(d,p) level. S32
Table S17. Energy analyses of 2*S*-**8** (two conformers)....S36
Table S18. Cartesian coordinates of the low-energy conformers of 2*S*-**8** re-optimized at B3LYP/6-31G(d,p) level....S37
Table S19. Experimental and calculated ^{1}H NMR data for compound **8**. S39
Table S20. Experimental and calculated ^{13}C{^{1}H} NMR data for compound **8**. S40
Table S21. DP4+ analyses of calculated and experimental NMR chemical shifts of **8** (unscaled). Isomer 1: 2*R*-**8**; Isomer 2: 2*S*-**8**. S41
Table S22. The effect of compounds **1**–**16** on ND7/23 DRG neurons viability. S42
Table S23. Neuroprotective potentials and anticancer interfering effects of compounds **1**–**16**. S43

Table S24. Crystal data and experimental details for **1** S44
Table S25. Bond lengths [Å] and angles [°] for **1** S45
Table S26. Crystal data and experimental details for **3** S49
Table S27. Bond lengths [Å] and angles [°] for **3** S50
Table S28. Crystal data and experimental details for **16** S57
Table S29. Bond lengths [Å] and angles [°] for **16** S58
Table S30. Crystal data and experimental details for kuroshine E S62
Table S31. Bond lengths [Å] and angles [°] for kuroshine E S63
Table S32. Crystal data and experimental details for 28-deoxyzoanthenamine S67
Table S33. Bond lengths [Å] and angles [°] for 28-deoxyzoanthenamine S68
Figure S1. ^{1}H NMR spectrum of **1** (C_5D_5N, 600 MHz) S72
Figure S2. ^{13}C{^{1}H} NMR and DEPT spectra of **1** (C_5D_5N, 150 MHz) S73
Figure S3. COSY spectrum of **1** S74
Figure S4. HSQC spectrum of **1** S75
Figure S5. HMBC spectrum of **1** S76
Figure S6. NOESY spectrum of **1** S77
Figure S7. HRESIMS spectrum of **1** S78
Figure S8. UV spectrum of **1** S79
Figure S9. IR spectrum of **1** S79
Figure S10. ^{1}H NMR spectrum of **2** (C_5D_5N, 600 MHz) S80
Figure S11. ^{13}C{^{1}H} NMR and DEPT spectra of **2** (C_5D_5N, 150 MHz) S81
Figure S12. COSY spectrum of **2** S82
Figure S13. HSQC spectrum of **2** S83
Figure S14. HMBC spectrum of **2** S84
Figure S15. NOESY spectrum of **2** S85

Figure S16. HRESIMS spectrum of **2** S86
Figure S17. UV spectrum of **2** S87
Figure S18. IR spectrum of **2** S87
Figure S19. ^{1}H NMR spectrum of **3** (C_5D_5N, 600 MHz) S88
Figure S20. ^{13}C{^{1}H} NMR and DEPT spectra of **3** (C_5D_5N, 150 MHz) S89
Figure S21. COSY spectrum of **3** S90
Figure S22. HSQC spectrum of **3** S91
Figure S23. HMBC spectrum of **3** S92
Figure S24. NOESY spectrum of **3** S93
Figure S25. HRESIMS spectrum of **3** S94
Figure S26. UV spectrum of **3** S95
Figure S27. IR spectrum of **3** S95
Figure S28. ^{1}H NMR spectrum of **4** (C_5D_5N, 400 MHz) S96
Figure S29. ^{13}C{^{1}H} NMR and DEPT spectra of **4** (C_5D_5N, 100 MHz) S97
Figure S30. COSY spectrum of **4** S98
Figure S31. HSQC spectrum of **4** S99
Figure S32. HMBC spectrum of **4** S100
Figure S33. NOESY spectrum of **4** S101
Figure S34. HRESIMS spectrum of **4** S102
Figure S35. UV spectrum of **4** S103
Figure S36. IR spectrum of **4** S103
Figure S37. ^{1}H NMR spectrum of **5** (C_5D_5N, 400 MHz) S104
Figure S38. ^{13}C{^{1}H} NMR and DEPT spectra of **5** (C_5D_5N, 100 MHz) S105
Figure S39. COSY spectrum of **5** S106
Figure S40. HSQC spectrum of **5** S107

Figure S41. HMBC spectrum of **5** S108
Figure S42. NOESY spectrum of **5** S109
Figure S43. HRESIMS spectrum of **5** S110
Figure S44. UV spectrum of **5** S111
Figure S45. IR spectrum of **5** S111
Figure S46. ^{1}H NMR spectrum of **6** (C_5D_5N, 600 MHz) S112
Figure S47. $^{13}C\{^{1}H\}$ NMR and DEPT spectra of **6** (C_5D_5N, 150 MHz) S113
Figure S48. COSY spectrum of **6** S114
Figure S49. HSQC spectrum of **6** S115
Figure S50. HMBC spectrum of **6** S116
Figure S51. NOESY spectrum of **6** S117
Figure S52. HRESIMS spectrum of **6** S118
Figure S53. UV spectrum of **6** S119
Figure S54. IR spectrum of **6** S119
Figure S55. ^{1}H NMR spectrum of **7** (C_5D_5N, 600 MHz) S120
Figure S56. $^{13}C\{^{1}H\}$ NMR and DEPT spectra of **7** (C_5D_5N, 150 MHz) S121
Figure S57. COSY spectrum of **7** S122
Figure S58. HSQC spectrum of **7** S123
Figure S59. HMBC spectrum of **7** S124
Figure S60. NOESY spectrum of **7** S125
Figure S61. HRESIMS spectrum of **7** S126
Figure S62. UV spectrum of **7** S127
Figure S63. IR spectrum of **7** S127
Figure S64. ^{1}H NMR spectrum of **8** (C_5D_5N, 400 MHz) S128
Figure S65. $^{13}C\{^{1}H\}$ NMR spectrum of **8** (C_5D_5N, 100 MHz) S129

Figure S66. COSY spectrum of **8** S130
Figure S67. HSQC spectrum of **8** S131
Figure S68. HMBC spectrum of **8** S132
Figure S69. NOESY spectrum of **8** S133
Figure S70. HRESIMS spectrum of **8** S134
Figure S71. UV spectrum of **8** S135
Figure S72. IR spectrum of **8** S135
Figure S73. ^{1}H NMR spectrum of **9** (C_5D_5N, 600 MHz) S136
Figure S74. ^{13}C{^{1}H} NMR and DEPT spectra of **9** (C_5D_5N, 150 MHz) S137
Figure S75. COSY spectrum of **9** S138
Figure S76. HSQC spectrum of **9** S139
Figure S77. HMBC spectrum of **9** S140
Figure S78. NOESY spectrum of **9** S141
Figure S79. HRESIMS spectrum of **9** S142
Figure S80. UV spectrum of **9** S143
Figure S81. IR spectrum of **9** S143
Figure S82. ^{1}H NMR spectrum of **10** (C_5D_5N, 600 MHz) S144
Figure S83. ^{13}C{^{1}H} NMR and DEPT spectra of **10** (C_5D_5N, 150 MHz) S145
Figure S84. COSY spectrum of **10** S146
Figure S85. HSQC spectrum of **10** S147
Figure S86. HMBC spectrum of **10** S148
Figure S87. NOESY spectrum of **10** S149
Figure S88. HRESIMS spectrum of **10** S150
Figure S89. UV spectrum of **10** S151
Figure S90. IR spectrum of **10** S151

Figure S91. ^{1}H NMR spectrum of **11** (C_5D_5N, 600 MHz) S152
Figure S92. $^{13}C\{^1H\}$ NMR and DEPT spectra of **11** (C_5D_5N, 150 MHz) S153
Figure S93. COSY spectrum of **11** S154
Figure S94. HSQC spectrum of **11** S155
Figure S95. HMBC spectrum of **11** S156
Figure S96. NOESY spectrum of **11** S157
Figure S97. HRESIMS spectrum of **11** S158
Figure S98. UV spectrum of **11** S159
Figure S99. IR spectrum of **11** S159
Figure S100. ^{1}H NMR spectrum of **12** (C_5D_5N, 600 MHz) S160
Figure S101. $^{13}C\{^1H\}$ NMR and DEPT spectra of **12** (C_5D_5N, 150 MHz) S161
Figure S102. COSY spectrum of **12** S162
Figure S103. HSQC spectrum of **12** S163
Figure S104. HMBC spectrum of **12** S164
Figure S105. NOESY spectrum of **12** S165
Figure S106. HRESIMS spectrum of **12** S166
Figure S107. UV spectrum of **12** S167
Figure S108. IR spectrum of **12** S167
Figure S109. ^{1}H NMR spectrum of **13** (C_5D_5N, 600 MHz) S168
Figure S110. $^{13}C\{^1H\}$ NMR and DEPT spectra of **13** (C_5D_5N, 150 MHz) S169
Figure S111. COSY spectrum of **13** S170
Figure S112. HSQC spectrum of **13** S171
Figure S113. HMBC spectrum of **13** S172
Figure S114. NOESY spectrum of **13** S173
Figure S115. HRESIMS spectrum of **13** S174

Figure S116. UV spectrum of **13** S175
Figure S117. IR spectrum of **13** S175
Figure S118. ^{1}H NMR spectrum of **14** (C_5D_5N, 400 MHz)....S176
Figure S119. $^{13}C\{^{1}H\}$ NMR and DEPT spectra of **14** (C_5D_5N, 100 MHz) S177
Figure S120. COSY spectrum of **14** S178
Figure S121. HSQC spectrum of **14** S179
Figure S122. HMBC spectrum of **14** S180
Figure S123. NOESY spectrum of **14**....S181
Figure S124. HRESIMS spectrum of **14**....S182
Figure S125. UV spectrum of **14** S183
Figure S126. IR spectrum of **14** S183
Figure S127. X-ray ORTEP drawings of 18-*epi*-kuroshine E (**16**) (displacement ellipsoids are drawn at the 50% probability level). S184
Figure S128. X-ray ORTEP drawings of kuroshine E (displacement ellipsoids are drawn at the 50% probability level)....S185
Figure S129. X-ray ORTEP drawings of 28-deoxyzoanthenamine (displacement ellipsoids are drawn at the 50% probability level)....S186

Table S1. Energy analyses of 10*R*-**2** (four conformers).

NO.	3D conformers B3LYP/6-31G(d,p)	E (Hartree)	ΔE (kJ/mol)	Boltzmann distribution
10*R*-**2** (conformer 1)	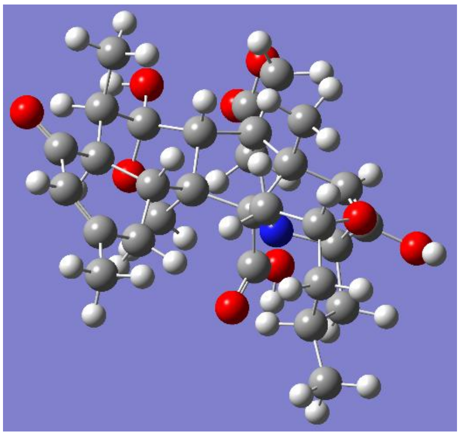	-1896.615210	9.845620668	1.71%
10*R*-**2** (conformer 2)	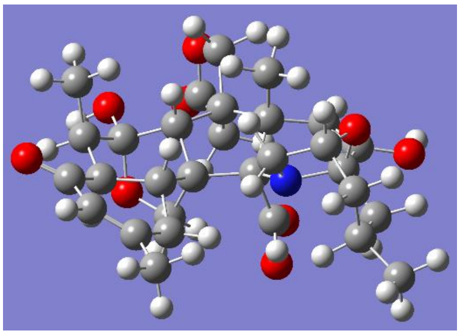	-1896.618960	0	90.69%
10*R*-**2** (conformer 3)	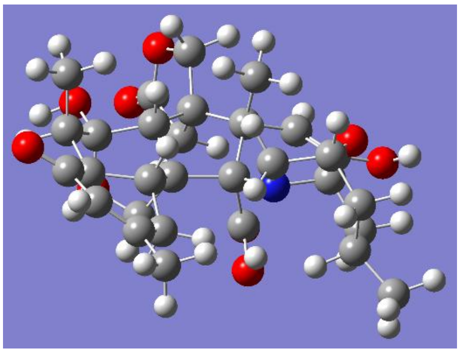	-1896.616175	7.312014282	4.75%
10*R*-**2** (conformer 4)	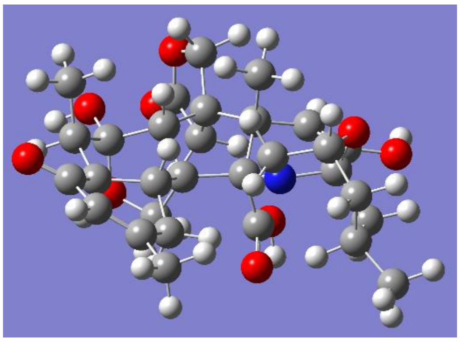	-1896.615693	8.577504726	2.85%

Table S2. Cartesian coordinates of the low-energy conformers of 10*R*-**2** re-optimized at B3LYP/6-31G(d,p) level.

10*R*-**2** (conformer 1)							
Atomic	Standard Orientation (Å)			Atomic	Standard Orientation (Å)		
Type	X	Y	Z	Type	X	Y	Z
C	-1.408203	0.322765	1.860825	O	0.564644	-2.17441	2.147568
C	-0.791279	4.723862	-0.193254	H	-1.155403	-0.576971	2.420109
C	0.309648	4.181167	0.364407	H	-1.128015	1.17552	2.484612
C	0.31083	2.741515	0.826162	H	-0.809288	5.75938	-0.523063
C	-0.690011	1.886527	0.025161	H	1.317735	2.322036	0.792801
C	-2.093789	2.556951	0.192065	H	0.043454	2.729071	1.892644
C	-2.066977	3.995874	-0.331637	H	-0.406943	1.940847	-1.033293
C	-0.752354	0.386421	0.430816	H	-2.231033	2.68947	1.275773
C	-1.861461	-0.325397	-0.391124	H	-1.831882	0.005436	-1.426768
C	-3.111756	0.264477	0.245034	H	-4.187412	2.112467	0.262609
C	-3.314295	1.716931	-0.272853	H	-1.300357	-3.44737	1.094136
C	0.532588	-0.56557	0.241629	H	-2.223352	-2.08863	1.72372
C	0.008982	-1.739896	-0.683789	H	-2.65913	-1.975515	-2.32774
C	-1.539392	-1.857299	-0.400891	H	-1.661899	-3.403051	-1.972608
C	-2.017582	-2.665837	0.827351	H	0.633249	-3.932096	-0.503046
C	-3.312284	-3.339748	0.401517	H	1.354235	0.90404	-2.122787
O	-3.430934	-3.2794	-0.951588	H	2.160256	1.909578	-0.90964
C	-2.297345	-2.627101	-1.529759	H	3.596484	0.702868	-2.908581
N	1.83255	-0.101785	-0.272509	H	4.772348	2.00004	-1.074993
C	2.842249	-1.197993	-0.443519	H	5.565254	0.472661	-1.458736
C	2.271882	-2.619044	-0.421812	H	3.778868	1.0912	0.943767
C	0.964463	-2.89801	-0.498377	H	3.702435	-1.208839	1.567907
C	2.093892	0.895744	-1.314943	H	4.820352	-1.687548	0.291861
C	3.467503	0.445388	-1.854014	H	1.461119	6.010938	0.2763
C	4.662522	0.909761	-1.01282	H	1.846121	4.95424	1.651697
C	4.536075	0.46334	0.460467	H	2.410232	4.525621	0.044821
C	4.016462	-0.98579	0.544616	H	-4.350577	0.995291	-2.036714
O	-4.122636	-3.890187	1.099496	H	-2.740248	1.634748	-2.401025
C	1.567728	4.970033	0.590186	H	-4.050251	2.748599	-2.029341
O	-3.067352	4.55132	-0.768451	H	-4.962141	-0.200066	0.547932
C	-3.629712	1.775452	-1.776671	H	3.992979	-3.379791	-0.77278
O	-4.221317	-0.549999	0.032419	H	5.745067	0.35215	2.271727
O	-2.822313	0.328376	1.655275	H	6.191828	1.680662	1.192083
O	3.19642	-3.626208	-0.281746	H	6.649004	0.020375	0.785578
O	3.37506	-0.986156	-1.766111	H	1.209455	-1.255589	-2.470633
C	5.854507	0.638591	1.220435	H	-0.191098	-2.228611	-2.801
C	0.163982	-1.382965	-2.207716	H	-0.389588	-0.499363	-2.532917
C	0.920782	-0.946913	1.713579	H	0.835806	-2.213723	3.081915
O	1.470746	-0.156711	2.451238				

10*R*-**2** (conformer 2)							
Atomic	Standard Orientation (Å)			Atomic	Standard Orientation (Å)		
Type	X	Y	Z	Type	X	Y	Z
C	-1.449124	0.309638	1.887128	O	1.617177	-0.208125	2.411721
C	-0.944471	4.720601	-0.177508	H	-1.172808	-0.562264	2.474337
C	0.167851	4.217713	0.391694	H	-1.219054	1.192503	2.491016
C	0.215554	2.776992	0.85125	H	-0.996149	5.754973	-0.507026
C	-0.746964	1.878076	0.048665	H	1.244501	2.413404	0.8053
C	-2.174401	2.50786	0.197558	H	-0.049095	2.75171	1.918299
C	-2.19122	3.946074	-0.327969	H	-0.457401	1.937357	-1.007156
C	-0.771268	0.372598	0.465894	H	-2.328872	2.637722	1.279144
C	-1.85015	-0.359349	-0.380623	H	-1.809437	-0.010849	-1.410543
C	-3.126247	0.189721	0.241918	H	-4.254393	2.005736	0.245604
C	-3.365533	1.6341	-0.280336	H	-1.228475	-3.467643	1.088073
C	0.529537	-0.569933	0.278814	H	-2.189021	-2.142607	1.702461
C	0.056637	-1.723963	-0.686308	H	-2.598441	-1.994661	-2.347003
C	-1.49279	-1.880441	-0.411523	H	-1.565964	-3.40059	-2.010968
C	-1.96371	-2.710373	0.805067	H	0.679944	-3.89599	-0.416207
C	-3.23447	-3.412856	0.358316	H	1.280255	0.95173	-1.968218
O	-3.341582	-3.342837	-0.998002	H	2.170848	1.965542	-0.824942
C	-2.224849	-2.650384	-1.557402	H	3.446538	0.738209	-2.923608
N	1.859893	-0.035351	-0.139638	H	4.783736	2.04075	-1.211265
C	2.886388	-1.147875	-0.398295	H	5.522718	0.498274	-1.643288
C	2.323645	-2.568396	-0.318636	H	3.973276	1.188553	0.889233
C	1.023379	-2.864747	-0.458063	H	3.887807	-1.138264	1.54207
C	2.076715	0.952425	-1.22239	H	4.882128	-1.642325	0.17788
C	3.402004	0.490127	-1.859803	H	1.255545	6.085502	0.316775
C	4.667147	0.952654	-1.12863	H	1.659882	5.046455	1.698601
C	4.660204	0.518866	0.352802	H	2.260479	4.636532	0.098707
C	4.123632	-0.921626	0.495863	H	-4.357764	0.876396	-2.052481
O	-4.039581	-3.992975	1.037679	H	-2.767888	1.572269	-2.403726
C	1.395186	5.049505	0.633374	H	-4.12035	2.638803	-2.04584
O	-3.204953	4.466937	-0.774897	H	-4.951424	-0.357316	0.556244
C	-3.667398	1.681884	-1.787393	H	2.929731	-4.359278	-0.030212
O	-4.208925	-0.656486	0.011971	H	6.016213	0.393481	2.05664
O	-2.861259	0.260611	1.653587	H	6.401155	1.702047	0.928109
O	3.322175	-3.478994	-0.111281	H	6.777612	0.02538	0.50202
O	3.304721	-0.934561	-1.752931	H	1.265036	-1.213029	-2.458044
C	6.042119	0.669374	0.997311	H	-0.115194	-2.207198	-2.803841
C	0.219986	-1.358566	-2.203646	H	-0.349594	-0.484896	-2.529096
C	0.861473	-1.094354	1.7318	H	1.99026	0.368607	1.704437
O	0.461133	-2.104775	2.254953				

10*R*-**2** (conformer 3)							
Atomic	Standard Orientation (Å)			Atomic	Standard Orientation (Å)		
Type	X	Y	Z	Type	X	Y	Z
C	-1.439148	0.313494	1.88701	O	1.603229	-0.187185	2.408815
C	-0.949702	4.71543	-0.20581	H	-1.159755	-0.557517	2.474469
C	0.164966	4.216982	0.362459	H	-1.203781	1.197208	2.487611
C	0.215052	2.779006	0.830571	H	-1.003532	5.747749	-0.541342
C	-0.748902	1.874225	0.035977	H	1.244604	2.416695	0.784285
C	-2.176277	2.504101	0.188102	H	-0.046936	2.759716	1.898355
C	-2.196592	3.938879	-0.346394	H	-0.462947	1.927695	-1.021411
C	-0.770768	0.371246	0.461122	H	-2.324151	2.640654	1.269818
C	-1.854662	-0.366107	-0.374446	H	-1.821725	-0.022494	-1.406278
C	-3.126916	0.185771	0.253557	H	-4.255467	2.00159	0.253337
C	-3.37013	1.626898	-0.276295	H	-1.221398	-3.476761	1.091737
C	0.529892	-0.570647	0.270805	H	-2.16969	-2.148501	1.721783
C	0.052541	-1.729069	-0.681945	H	-2.602309	-2.013176	-2.333887
C	-1.494485	-1.886676	-0.400414	H	-1.572064	-3.418791	-1.986926
C	-1.956756	-2.714909	0.820534	H	0.708474	-3.907696	-0.479198
C	-3.234981	-3.410641	0.384216	H	1.307289	0.934566	-2.007162
O	-3.347217	-3.347571	-0.972603	H	2.153356	1.96541	-0.845639
C	-2.229363	-2.663489	-1.539394	H	3.506799	0.761656	-2.899866
N	1.857442	-0.038285	-0.159592	H	4.782094	2.057965	-1.133917
C	2.880374	-1.148308	-0.402208	H	5.546012	0.528495	-1.563486
C	2.32468	-2.574596	-0.367595	H	3.928419	1.179667	0.935685
C	1.024137	-2.869173	-0.478441	H	3.831213	-1.151695	1.565471
C	2.083646	0.949152	-1.239951	H	4.880964	-1.629581	0.238938
C	3.432077	0.504319	-1.840029	H	1.250929	6.085059	0.271911
C	4.671909	0.968302	-1.067045	H	1.659278	5.05637	1.660096
C	4.63105	0.518943	0.409809	H	2.256864	4.635782	0.061664
C	4.09461	-0.924357	0.528633	H	-4.373606	0.856741	-2.036404
O	-4.042106	-3.979801	1.070047	H	-2.787211	1.552434	-2.403461
C	1.392251	5.051443	0.595444	H	-4.138562	2.619461	-2.042967
O	-3.21227	4.456521	-0.792347	H	-4.947949	-0.363343	0.587616
C	-3.682503	1.664843	-1.781483	H	4.025156	-3.377313	-0.748634
O	-4.210619	-0.661762	0.035974	H	5.946106	0.382292	2.144639
O	-2.852353	0.266117	1.662921	H	6.349903	1.70536	1.040145
O	3.257648	-3.569891	-0.191469	H	6.748473	0.036911	0.605173
O	3.342603	-0.923561	-1.745625	H	1.252623	-1.212558	-2.460353
C	5.996005	0.669927	1.089426	H	-0.121776	-2.217308	-2.800394
C	0.209375	-1.365754	-2.202062	H	-0.366855	-0.496192	-2.526499
C	0.867517	-1.088592	1.722274	H	1.968762	0.400094	1.709513
O	0.485697	-2.10693	2.241054				

10*R*-**2** (conformer 4)							
Atomic	Standard Orientation (Å)			Atomic	Standard Orientation (Å)		
Type	X	Y	Z	Type	X	Y	Z
C	-1.420497	0.317837	1.859606	O	0.560378	-2.171291	2.168566
C	-0.797313	4.728046	-0.169673	H	-1.169171	-0.58252	2.418504
C	0.302198	4.184095	0.38982	H	-1.147453	1.169738	2.48762
C	0.303888	2.742411	0.844833	H	-0.815225	5.765068	-0.49473
C	-0.692623	1.89002	0.035995	H	1.311262	2.324126	0.812715
C	-2.098276	2.557163	0.198413	H	0.032775	2.724719	1.910336
C	-2.071147	3.999141	-0.317139	H	-0.404997	1.949818	-1.020637
C	-0.754191	0.387798	0.434599	H	-2.243116	2.683561	1.281846
C	-1.856245	-0.321946	-0.39814	H	-1.819031	0.01218	-1.432505
C	-3.112219	0.262741	0.23132	H	-4.191796	2.108297	0.251079
C	-3.313868	1.717414	-0.279976	H	-1.295751	-3.435016	1.097571
C	0.53386	-0.56244	0.250919	H	-2.240113	-2.082754	1.706015
C	0.015839	-1.734248	-0.68551	H	-2.653693	-1.965091	-2.337266
C	-1.533909	-1.853548	-0.410269	H	-1.646984	-3.388301	-1.994889
C	-2.017223	-2.663011	0.815674	H	0.61275	-3.919159	-0.43897
C	-3.300679	-3.352164	0.380635	H	1.325438	0.923647	-2.083366
O	-3.415478	-3.286789	-0.972111	H	2.173139	1.914557	-0.886872
C	-2.28703	-2.620444	-1.544703	H	3.538415	0.690413	-2.931504
N	1.836339	-0.094885	-0.250025	H	4.777607	1.990813	-1.144595
C	2.851276	-1.192781	-0.43866	H	5.546426	0.451922	-1.537121
C	2.276294	-2.610268	-0.377899	H	3.83106	1.10617	0.906541
C	0.968194	-2.891065	-0.475507	H	3.755844	-1.190337	1.549173
C	2.085397	0.903406	-1.295315	H	4.824455	-1.696924	0.236112
C	3.438914	0.439252	-1.872118	H	1.451769	6.015683	0.314877
C	4.661182	0.901797	-1.070045	H	1.83394	4.951838	1.685736
C	4.570511	0.467352	0.409057	H	2.402899	4.532239	0.078353
C	4.047426	-0.97895	0.515909	H	-4.336297	1.004407	-2.055658
O	-4.105743	-3.917524	1.073068	H	-2.723735	1.647028	-2.404099
C	1.558562	4.973222	0.623549	H	-4.037448	2.757854	-2.0362
O	-3.070673	4.554845	-0.755913	H	-4.965898	-0.203044	0.511793
C	-3.61801	1.783649	-1.785791	H	2.87094	-4.420238	-0.214786
O	-4.218459	-0.553493	0.006272	H	5.825783	0.362408	2.188934
O	-2.833395	0.319106	1.644072	H	6.254508	1.678549	1.086576
O	3.267704	-3.53809	-0.215978	H	6.686504	0.010193	0.682392
O	3.340562	-0.988781	-1.775031	H	1.227954	-1.253856	-2.463206
C	5.909691	0.639391	1.13263	H	-0.175527	-2.220394	-2.802141
C	0.18032	-1.376528	-2.206667	H	-0.36791	-0.490662	-2.534718
C	0.91333	-0.942718	1.726606	H	0.816976	-2.190319	3.107842
O	1.45228	-0.148	2.466619				

Table S3. Energy analyses of 10*S*-**2** (three conformers).

NO.	**3D conformers** B3LYP/6-31G(d,p)	**E (Hartree)**	**ΔE (kJ/mol)**	**Boltzmann distribution**
10*S*-**2** (conformer 1)	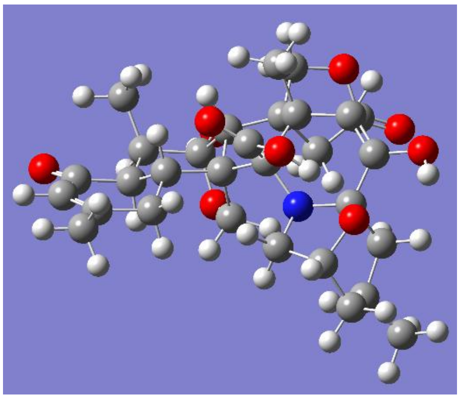	-1896.653860	2.88804873	23.55%
10*S*-**2** (conformer 2)	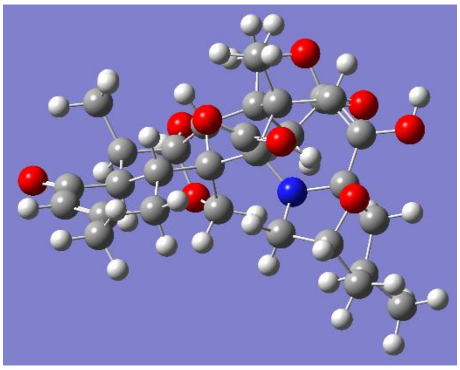	-1896.654960	0	75.49%
10*S*-**2** (conformer 3)	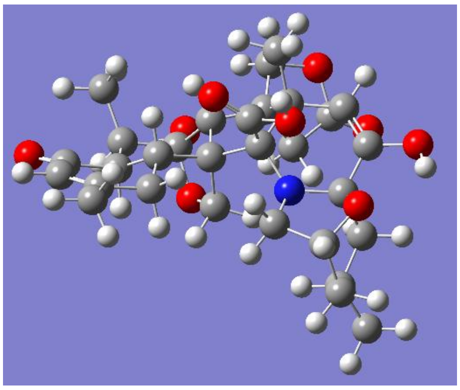	-1896.650846	10.80130225	0.97%

Table S4. Cartesian coordinates of the low-energy conformers of 10*S*-**2** re-optimized at B3LYP/6-31G(d,p) level.

10*S*-**2** (conformer 1)							
Atomic	Standard Orientation (Å)			Atomic	Standard Orientation (Å)		
Type	X	Y	Z	Type	X	Y	Z
C	-0.65696	0.024823	-1.61923	O	1.017855	-2.022422	2.536923
C	-4.873031	-2.032827	-0.09526	H	0.40594	0.099586	-1.837563
C	-3.777042	-2.769889	0.166373	H	-1.037652	-0.878965	-2.109332
C	-2.387168	-2.19616	0.002141	H	-5.87394	-2.439959	0.021303
C	-2.350796	-0.663983	0.145005	H	-1.74198	-2.664741	0.746813
C	-3.408538	-0.068501	-0.838534	H	-2.006694	-2.504989	-0.984722
C	-4.803772	-0.655635	-0.60489	H	-2.655127	-0.441682	1.169731
C	-0.952229	0.025151	-0.084478	H	-3.15015	-0.439712	-1.841884
C	-1.143903	1.544163	0.145909	H	-1.756727	1.724333	1.03112
C	-1.935911	1.927082	-1.103016	H	-3.905755	1.723664	-1.900178
C	-3.407973	1.477173	-0.952685	H	0.591888	2.717749	-1.65677
C	0.339951	-0.361876	0.810838	H	1.794077	1.55132	-1.089568
C	0.835325	1.051294	1.42377	H	-0.007595	3.666764	2.037682
C	0.248638	2.137229	0.418614	H	-0.579635	4.14678	0.426693
C	1.174985	2.392525	-0.790043	H	2.709378	1.866538	2.295744
C	2.060819	3.546564	-0.349413	H	0.375819	-2.84607	0.207225
O	1.472987	4.176465	0.709826	H	0.815072	-2.511448	-1.467927
C	0.183986	3.583147	0.969546	H	2.416367	-4.049945	0.313583
N	1.36916	-0.979222	-0.066705	H	2.952692	-3.933379	-2.129791
C	2.801621	-0.871099	0.191937	H	4.370715	-3.505005	-1.169795
C	3.183855	0.177067	1.19126	H	2.454772	-1.623438	-2.632902
C	2.326062	1.092354	1.637204	H	4.691758	-0.67354	-0.802719
C	1.141297	-2.397693	-0.428933	H	3.426793	0.28958	-1.536127
C	2.514887	-3.084689	-0.187709	H	-4.891844	-4.557403	0.657316
C	3.364544	-3.17354	-1.454102	H	-3.286043	-4.854305	-0.041549
C	3.425221	-1.801317	-2.151797	H	-3.420668	-4.304625	1.623826
C	3.638877	-0.69296	-1.10465	H	-3.911108	3.288255	0.136482
O	3.111454	3.903838	-0.814327	H	-3.886442	1.849329	1.166468
C	-3.860913	-4.197672	0.624433	H	-5.225442	2.095747	0.049592
O	-5.819539	-0.038218	-0.904204	H	-2.340027	3.464912	-2.197086
C	-4.149612	2.221344	0.170632	H	4.852233	-0.698174	1.481118
O	-1.81983	3.290895	-1.399373	H	5.495217	-1.92241	-2.82612
O	-1.332843	1.165643	-2.165606	H	4.506403	-0.779755	-3.747101
O	4.50825	0.205561	1.535481	H	4.318292	-2.520214	-4.006242
O	3.15836	-2.183052	0.747924	H	0.618044	0.509536	3.539367
C	4.496892	-1.752807	-3.245496	H	0.610307	2.230805	3.240361
C	0.253899	1.274955	2.852507	H	-0.83583	1.261938	2.891151
C	0.015007	-1.277359	2.037848	H	1.846425	-1.992617	2.010631
O	-1.060661	-1.292594	2.594783				

10*S*-**2** (conformer 2)							
Atomic	Standard Orientation (Å)			Atomic	Standard Orientation (Å)		
Type	X	Y	Z	Type	X	Y	Z
C	-0.669171	0.01131	-1.628856	O	1.030903	-1.986375	2.545967
C	-4.877453	-2.036752	-0.069182	H	0.392628	0.081735	-1.852849
C	-3.780101	-2.769681	0.198345	H	-1.054213	-0.896051	-2.108682
C	-2.390993	-2.196915	0.025625	H	-5.877728	-2.44362	0.053666
C	-2.354467	-0.663168	0.149845	H	-1.745579	-2.655069	0.776408
C	-3.416205	-0.079401	-0.837089	H	-2.010777	-2.518384	-0.957224
C	-4.810375	-0.665531	-0.594049	H	-2.655153	-0.429315	1.173051
C	-0.957114	0.025648	-0.092524	H	-3.160153	-0.460747	-1.837199
C	-1.154262	1.54589	0.124819	H	-1.765698	1.730954	1.010099
C	-1.948875	1.916507	-1.125704	H	-3.919918	1.7005	-1.916617
C	-3.419414	1.465113	-0.967756	H	0.584164	2.739586	-1.678085
C	0.343406	-0.346782	0.802369	H	1.767052	1.542593	-1.128695
C	0.827095	1.068798	1.404443	H	-0.037304	3.688737	1.992755
C	0.235208	2.145024	0.390589	H	-0.573051	4.162171	0.366874
C	1.164976	2.392415	-0.818031	H	2.687759	1.957771	2.191127
C	2.070769	3.522499	-0.358515	H	0.337083	-2.798323	0.193417
O	1.476447	4.168265	0.688536	H	0.835885	-2.502347	-1.47104
C	0.173095	3.594082	0.928945	H	2.350781	-4.03032	0.405989
N	1.374433	-0.956041	-0.080612	H	2.947348	-4.013173	-2.02439
C	2.814744	-0.873766	0.193936	H	4.351688	-3.572368	-1.047134
C	3.193453	0.230698	1.135648	H	2.502116	-1.713288	-2.620673
C	2.319467	1.131671	1.584655	H	4.714853	-0.754998	-0.76073
C	1.131768	-2.377392	-0.424579	H	3.488402	0.218001	-1.56138
C	2.482582	-3.084899	-0.124772	H	-4.892216	-4.552922	0.710627
C	3.35829	-3.236418	-1.367332	H	-3.286997	-4.855589	0.012638
C	3.457686	-1.891071	-2.11037	H	-3.41964	-4.287823	1.671917
C	3.672903	-0.754855	-1.094562	H	-3.923301	3.286335	0.104145
O	3.145342	3.850183	-0.791348	H	-3.894587	1.857703	1.148452
C	-3.861731	-4.19242	0.672181	H	-5.235968	2.090913	0.031702
O	-5.827306	-0.05219	-0.898269	H	-2.35557	3.443854	-2.2334
C	-4.160141	2.219442	0.149448	H	4.746298	1.017275	1.925594
O	-1.836344	3.278944	-1.433117	H	5.539716	-2.069189	-2.730533
O	-1.345668	1.149206	-2.182512	H	4.59259	-0.943617	-3.714607
O	4.532825	0.240707	1.38931	H	4.380565	-2.688872	-3.917568
O	3.124594	-2.169561	0.795623	H	0.625086	0.545081	3.524712
C	4.55482	-1.898278	-3.179715	H	0.613008	2.265355	3.211298
C	0.256055	1.304844	2.834206	H	-0.833379	1.291272	2.880654
C	0.02655	-1.254995	2.038543	H	1.862441	-1.976088	2.014795
O	-1.050122	-1.263572	2.596034				

10*S*-**2** (conformer 3)							
Atomic	Standard Orientation (Å)			Atomic	Standard Orientation (Å)		
Type	X	Y	Z	Type	X	Y	Z
C	-0.591889	0.020595	-1.573209	O	1.047751	-1.797076	2.662362
C	-4.866125	-1.953545	-0.090112	H	0.475702	0.087468	-1.768939
C	-3.783972	-2.702488	0.194041	H	-0.96256	-0.888541	-2.060417
C	-2.384692	-2.148214	0.045865	H	-5.873982	-2.346814	0.013947
C	-2.329884	-0.615161	0.169428	H	-1.752536	-2.618004	0.801135
C	-3.365267	-0.018085	-0.834532	H	-1.992394	-2.472718	-0.930852
C	-4.771199	-0.581191	-0.610608	H	-2.64803	-0.372583	1.185708
C	-0.917117	0.045906	-0.047524	H	-3.098532	-0.404377	-1.829969
C	-1.075591	1.570545	0.156191	H	-1.683589	1.782808	1.038673
C	-1.854666	1.94616	-1.104568	H	-3.820302	1.771578	-1.917939
C	-3.336217	1.525024	-0.963374	H	0.660908	2.720064	-1.671312
C	0.351519	-0.365872	0.85988	H	1.877158	1.569033	-1.099957
C	0.931223	1.055339	1.411084	H	0.076142	3.677983	2.028548
C	0.330945	2.143331	0.410488	H	-0.498125	4.150787	0.417575
C	1.247614	2.403043	-0.804589	H	2.867506	1.818727	2.184012
C	2.130294	3.564193	-0.373172	H	0.464628	-2.98216	0.614954
O	1.553186	4.18667	0.697336	H	0.565732	-2.741397	-1.135374
C	0.26815	3.590962	0.961057	H	2.495956	-4.152594	0.35124
N	1.326128	-1.114091	0.040488	H	2.623593	-4.020816	-2.16156
C	2.781182	-0.986225	0.166202	H	4.170283	-3.605505	-1.417458
C	3.232948	0.07579	1.118013	H	2.06917	-1.709154	-2.565464
C	2.43121	1.043273	1.560803	H	4.542786	-0.774081	-1.050883
C	1.075182	-2.550823	-0.18308	H	3.203614	0.196222	-1.63263
C	2.490524	-3.185933	-0.158837	H	-4.931143	-4.47157	0.678274
C	3.133774	-3.269455	-1.545365	H	-3.321003	-4.79476	0.001874
C	3.099829	-1.890925	-2.233172	H	-3.470125	-4.237379	1.663495
C	3.460278	-0.784819	-1.222956	H	-3.819305	3.356118	0.099889
O	3.169386	3.933774	-0.853014	H	-3.823729	1.929842	1.147382
C	-3.894732	-4.126961	0.657591	H	-5.151459	2.183288	0.017909
O	-5.775227	0.047788	-0.924709	H	-2.218112	3.468966	-2.23316
C	-4.074601	2.29354	0.145293	H	4.863242	-0.868587	1.314363
O	-1.715887	3.303443	-1.422237	H	5.049216	-2.011773	-3.201798
O	-1.255756	1.156154	-2.148265	H	3.939996	-0.863896	-3.96553
O	4.56567	0.048732	1.414273	H	3.712999	-2.603421	-4.201453
O	3.240216	-2.266268	0.648364	H	0.767543	0.581558	3.548741
C	4.000851	-1.839126	-3.471256	H	0.840046	2.286812	3.20265
C	0.414783	1.344468	2.853836	H	-0.672707	1.401895	2.931439
C	-0.023984	-1.163112	2.134104	H	0.734785	-2.201151	3.489988
O	-1.105401	-1.198797	2.683936				

Table S5. Experimental and calculated ^{1}H NMR data for compound **2**.

No.	**2**, exptl. δ_H [a]	10*R*-**2**, calcd. δ_H [b]	10*S*-**2**, calcd. δ_H [b]
1	3.55	3.35	2.71
	3.82	3.92	2.93
2	4.53	4.75	4.57
3	1.45	1.52	1.50
	1.60	1.70	1.60
4	2.21	2.06	2.07
5	1.77	1.72	1.73
	2.60	1.75	2.10
8	5.81	5.94	4.55
13	2.92	2.66	3.29
14	2.77	2.64	2.18
	3.59	2.75	2.21
16	5.97	6.24	5.99
18	2.91	2.69	2.54
19	2.96	2.20	2.13
21	2.70	2.85	2.23
23	2.96	2.62	2.32
	4.39	3.21	3.63
25	4.83	4.11	4.36
	4.97	4.31	4.58
26	1.45	1.17	1.02
27	1.71	2.20	2.01
28	4.14	4.14	4.08
	4.50	4.21	4.76
29	1.56	1.56	1.15
30	1.05	1.03	1.01

[a] Recorded in C_5D_5N at 600 MHz.

[b] Calculated in C_5D_5N.

Table S6. Experimental and calculated $^{13}C\{^{1}H\}$ NMR data for compound **2**.

No.	**2**, exptl. δ_C [a]	10*R*-**2**, calcd. δ_C [b]	10*S*-**2**, calcd. δ_C [b]
1	45.0	51.38	51.90
2	68.5	74.86	70.32
3	38.6	34.19	32.29
4	26.4	23.17	21.53
5	48.6	36.59	38.65
6	93.6	97.66	87.33
7	138.4	149.82	146.36
8	107.3	111.60	99.15
9	54.7	56.05	53.64
10	83.4	86.44	79.69
11	184.7	174.84	170.45
12	61.6	61.16	62.94
13	41.8	40.92	39.01
14	34.0	32.66	30.85
15	162.5	166.97	163.91
16	126.2	127.49	122.49
17	199.4	198.75	193.92
18	50.3	49.71	46.62
19	43.2	42.43	41.05
20	111.0	107.18	105.40
21	50.8	57.77	46.71
22	55.0	50.23	53.67
23	36.6	33.57	33.69
24	177.8	178.27	172.99
25	73.7	70.39	66.52
26	13.3	7.31	6.89
27	24.2	21.10	20.67
28	71.6	71.07	66.47
29	22.1	21.71	23.10
30	22.1	16.69	16.06

[a] Recorded in C_5D_5N at 150 MHz.

[b] Calculated in C_5D_5N.

Table S7. DP4+ analyses of calculated and experimental NMR chemical shifts of **2** (unscaled).

Isomer 1: 10*R*-**2**; Isomer 2: 10*S*-**2**.

Functional	Solvent?		Basis Set		Type of Data	
mPW1PW91	PCM		6-311G(d,p)		Unscaled Shifts	
	Isomer 1	Isomer 2	Isomer 3	Isomer 4	Isomer 5	Isomer 6
sDP4+ (H data)	100.00%	0.00%	-	-	-	-
sDP4+ (C data)	0.05%	99.95%	-	-	-	-
sDP4+ (all data)	99.20%	0.80%	-	-	-	-
uDP4+ (H data)	100.00%	0.00%	-	-	-	-
uDP4+ (C data)	100.00%	0.00%	-	-	-	-
uDP4+ (all data)	100.00%	0.00%	-	-	-	-
DP4+ (H data)	100.00%	0.00%	-	-	-	-
DP4+ (C data)	100.00%	0.00%	-	-	-	-
DP4+ (all data)	100.00%	0.00%	-	-	-	-

Functional		Solvent?		Basis Set		Type of Data	
mPW1PW91		PCM		6-311G(d,p)		Unscaled Shifts	
		DP4+	100.00%	0.00%	0.00%	-	-
Nuclei	sp2?	Experimental	Isomer 1	Isomer 2	Isomer 3	Isomer 4	Isomer 5
C		45.0	51.38	51.90			
C		68.5	74.86	70.32			
C		38.6	34.19	32.29			
C		26.4	23.17	21.53			
C		48.6	36.59	38.65			
C		93.6	97.66	87.33			
C	x	138.4	149.82	146.36			
C	x	107.3	111.60	99.15			
C		54.7	56.05	53.64			
C		83.4	86.44	79.69			
C	x	184.7	174.84	170.45			
C		61.6	61.16	62.94			
C		41.8	40.92	39.01			
C		34.0	32.66	30.85			
C	x	162.5	166.97	163.91			
C	x	126.2	127.49	122.49			
C	x	199.4	198.75	193.92			
C		50.3	49.71	46.62			
C		43.2	42.43	41.05			
C		111.0	107.18	105.40			
C		50.8	57.77	46.71			
C		55.0	50.23	53.67			
C		36.6	33.57	33.69			
C	x	177.8	178.27	172.99			
C		73.7	70.39	66.52			
C		13.3	7.31	6.89			
C		24.2	21.10	20.67			
C		71.6	71.07	66.47			
C		22.1	21.71	23.10			
C		22.1	16.69	16.06			
H		3.55	3.35	2.71			
H		3.82	3.92	2.93			
H		4.53	4.75	4.57			
H		1.45	1.52	1.50			
H		1.60	1.70	1.60			
H		2.21	2.06	2.07			
H		1.77	1.72	1.73			
H		2.60	1.75	2.10			
H	x	5.81	5.94	4.55			
H		2.92	2.66	3.29			
H		2.77	2.64	2.18			
H		3.59	2.75	2.21			
H	x	5.97	6.24	5.99			
H		2.91	2.69	2.54			
H		2.96	2.20	2.13			
H		2.70	2.85	2.23			
H		2.96	2.62	2.32			
H		4.39	3.21	3.63			
H		4.83	4.11	4.36			
H		4.97	4.31	4.58			
H		1.45	1.17	1.02			
H		1.71	2.20	2.01			
H		4.14	4.14	4.08			
H		4.50	4.21	4.76			
H		1.56	1.56	1.15			
H		1.05	1.03	1.01			

Table S8. Energy analyses of 2*R*-**7** (four conformers).

**NO.**	**3D conformers** B3LYP/6-31G(d,p)	**E (Hartree)**	**ΔE (kJ/mol)**	**Boltzmann distribution**
2*R*-**7** (conformer 1)	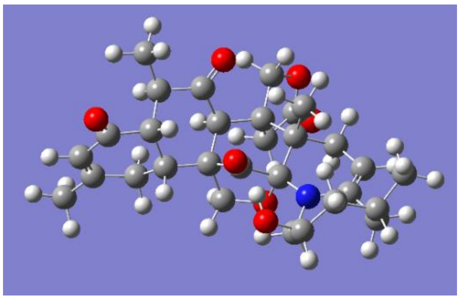	-1746.220300	2.439088427	13.25%
2*R*-**7** (conformer 2)	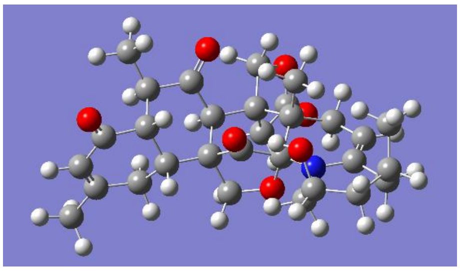	-1746.220679	1.444024365	19.79%
2*R*-**7** (conformer 3)	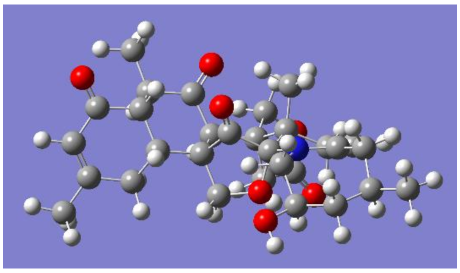	-1746.221229	0	35.44%
2*R*-**7** (conformer 4)	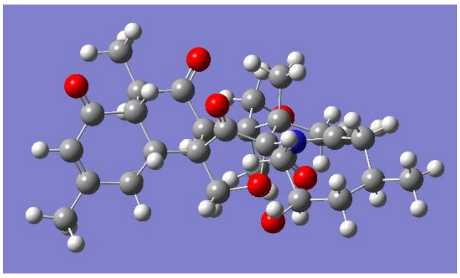	-1746.221118	0.291430372	31.51%

Table S9. Cartesian coordinates of the low-energy conformers of 2*R*-**7** re-optimized at B3LYP/6-31G(d,p) level.

2*R*-**7** (conformer 1)							
Atomic Type	Standard Orientation (Å) X	Y	Z	Atomic Type	Standard Orientation (Å) X	Y	Z
C	1.227533	0.001294	-0.603639	H	4.781392	1.624896	-0.198728
C	1.349363	1.282034	0.315776	H	2.409931	2.568953	-1.118237
O	0.031813	-1.774214	0.63317	H	2.793415	2.879809	0.563049
N	2.428856	-0.761591	-0.680044	H	2.94031	-1.963313	-2.338366
C	3.687682	-0.119157	-0.586633	H	1.345641	-2.24297	-1.69737
C	3.79446	1.187231	-0.296356	H	3.370868	-3.951443	-1.259978
C	2.594471	2.07839	-0.156229	H	3.219254	-2.268875	1.278315
C	2.376324	-2.042563	-1.402892	H	4.041517	-3.81248	1.117687
C	2.858042	-3.267119	-0.57241	H	5.773893	-2.87473	-0.266311
C	3.796597	-2.889624	0.581273	H	4.86043	-1.419431	-1.817374
C	5.099512	-2.152734	0.215023	H	5.781631	-0.352402	-0.79202
C	4.898186	-0.997361	-0.806107	H	0.762473	0.324545	2.230953
C	1.6179	0.811429	1.766837	H	2.459199	0.118519	1.777481
C	5.788844	-1.644224	1.489385	H	1.894752	1.660058	2.396535
C	0.020903	-0.795037	-0.081942	H	6.781884	-1.238025	1.26881
C	-1.192329	-0.069813	-0.64873	H	5.91027	-2.44864	2.222432
C	-0.64048	0.210934	-2.062762	H	5.199647	-0.848563	1.959164
O	0.775278	0.396028	-1.936515	H	-1.079046	1.098566	-2.528786
C	-1.304673	1.251063	0.205315	H	-0.838931	-0.650204	-2.717495
C	-1.902681	0.908371	1.583738	H	-2.092353	1.848785	-0.275928
C	-3.240835	0.165265	1.564015	H	-3.943322	0.800188	1.001486
C	-3.044827	-1.159688	0.760117	H	-2.331519	-1.777646	1.311903
C	-2.493181	-0.900042	-0.658398	H	-2.23151	-1.8809	-1.077424
C	-4.346949	-1.966536	0.705248	H	-6.172894	-2.301724	-0.422795
C	-5.222749	-1.775573	-0.463127	H	-3.735399	0.770905	-1.364089
C	-4.896839	-1.00468	-1.518322	H	-3.213932	-0.313061	-2.626986
C	-3.566031	-0.293358	-1.588279	H	-6.787466	-1.29449	-2.525182
C	-5.828202	-0.795433	-2.678367	H	-6.016231	0.274032	-2.838483
C	-3.777181	-0.04132	2.982228	H	-5.383438	-1.174909	-3.607094
O	-1.370942	1.225704	2.630566	H	-4.763637	-0.506211	2.963162
O	-4.660592	-2.717242	1.618473	H	-3.84449	0.916167	3.503963
C	-0.002928	2.138828	0.194443	H	-3.111098	-0.687408	3.559083
C	-0.014897	3.070441	-1.055727	H	0.497477	2.688178	-1.934153
C	0.608321	4.378409	-0.595505	H	-1.046457	3.307149	-1.346295
O	0.596421	4.410718	0.763623	H	-1.131638	3.53489	1.470852
C	-0.089115	3.253919	1.286249	H	0.363955	3.002986	2.238748
O	1.04468	5.274726	-1.269014	H	1.243914	-3.419441	0.494468
O	1.753852	-4.015808	-0.076552				

2*R*-**7** (conformer 2)							
Atomic	Standard Orientation (Å)			Atomic	Standard Orientation (Å)		
Type	X	Y	Z	Type	X	Y	Z
C	1.216632	0.018643	-0.561654	H	4.686079	1.800258	-0.248466
C	1.279768	1.310653	0.350596	H	2.289522	2.662436	-1.061072
O	0.022598	-1.769799	0.672924	H	2.673794	2.942718	0.624024
N	2.445855	-0.706843	-0.61067	H	2.916504	-2.038415	-2.202909
C	3.673732	-0.001909	-0.573525	H	1.367869	-2.273834	-1.444492
C	3.72034	1.309996	-0.291193	H	2.626365	-4.108575	-0.813626
C	2.494417	2.152917	-0.113509	H	4.8139	-4.072977	0.29783
C	2.411075	-2.038206	-1.229008	H	4.820898	-3.547504	-1.380527
C	2.991839	-3.173203	-0.348382	H	6.354138	-2.205627	-0.222418
C	4.521061	-3.250684	-0.365669	H	4.938192	-1.093818	-1.921522
C	5.305339	-1.980868	0.020309	H	5.757063	-0.035734	-0.79853
C	4.945899	-0.765718	-0.87193	H	0.699625	0.365407	2.272879
C	1.554128	0.850337	1.80411	H	2.394541	0.156191	1.8158
C	5.255878	-1.645929	1.517749	H	1.830528	1.703584	2.427802
C	0.015515	-0.799851	-0.057713	H	5.861039	-0.759329	1.737898
C	-1.202912	-0.113918	-0.656452	H	5.652082	-2.479024	2.107724
C	-0.629152	0.181243	-2.058946	H	4.233855	-1.468726	1.85437
O	0.779371	0.392812	-1.907127	H	-1.077981	1.059827	-2.532219
C	-1.372644	1.198496	0.20022	H	-0.798982	-0.682665	-2.718386
C	-1.983816	0.830325	1.566455	H	-2.170514	1.773001	-0.292072
C	-3.301404	0.052242	1.518628	H	-4.010651	0.672732	0.948452
C	-3.056728	-1.261621	0.709856	H	-2.341172	-1.865572	1.273767
C	-2.480948	-0.979389	-0.694995	H	-2.186101	-1.950817	-1.114309
C	-4.336821	-2.100228	0.621246	H	-6.129327	-2.473684	-0.547828
C	-5.191921	-1.923939	-0.564651	H	-3.749941	0.663686	-1.417292
C	-4.862367	-1.139089	-1.608296	H	-3.173899	-0.397652	-2.675518
C	-3.548718	-0.394222	-1.644647	H	-6.722941	-1.46839	-2.65807
C	-5.772712	-0.945927	-2.78764	H	-5.982598	0.119569	-2.946851
C	-3.855975	-0.179235	2.925897	H	-5.298771	-1.309803	-3.708178
O	-1.478875	1.155591	2.623722	H	-4.828336	-0.671791	2.886769
O	-4.650639	-2.865381	1.522337	H	-3.959447	0.772385	3.45237
C	-0.099021	2.123135	0.212864	H	-3.181525	-0.809859	3.510087
C	-0.126609	3.058676	-1.034065	H	0.409761	2.697917	-1.907467
C	0.445193	4.38582	-0.561095	H	-1.161671	3.261621	-1.33733
O	0.416978	4.411471	0.797637	H	-1.285516	3.474174	1.48692
C	-0.232853	3.229328	1.309003	H	0.22173	2.987882	2.263383
O	0.856149	5.30014	-1.226315	H	1.671072	-2.712838	1.021553
O	2.562235	-3.098096	0.99692				

2*R*-**7** (conformer 3)							
Atomic	Standard Orientation (Å)			Atomic	Standard Orientation (Å)		
Type	X	Y	Z	Type	X	Y	Z
C	-1.161047	-0.024892	-0.143383	H	-4.49296	1.980212	-0.664166
C	-1.054212	1.511294	-0.485576	H	-2.410512	2.234996	1.085314
O	0.268281	-1.283645	-1.716502	H	-2.384496	3.220699	-0.364405
N	-2.358022	-0.641374	-0.621722	H	-1.451722	-2.488073	-0.280183
C	-3.543864	0.113917	-0.742775	H	-2.797348	-2.561356	-1.386445
C	-3.567851	1.436641	-0.508016	H	-3.192925	-1.850211	1.547291
C	-2.366919	2.18542	-0.008438	H	-4.879824	-3.418818	-0.467495
C	-2.451171	-2.095345	-0.457096	H	-5.340788	-3.136944	1.208465
C	-3.323976	-2.564368	0.720555	H	-5.536877	-0.726357	0.83434
C	-4.805219	-2.693816	0.355828	H	-5.484421	0.139226	-1.559337
C	-5.519987	-1.391107	-0.040502	H	-4.588966	-1.297557	-2.010435
C	-4.79602	-0.618127	-1.173041	H	-0.025045	1.35215	-2.444215
C	-0.981647	1.659063	-2.026673	H	-1.766097	1.062581	-2.492343
C	-6.972043	-1.689546	-0.441216	H	-1.150404	2.699208	-2.316203
C	0.126789	-0.677832	-0.679941	H	-7.524692	-0.766744	-0.645692
C	1.179758	-0.358342	0.378203	H	-7.500558	-2.227566	0.352921
C	0.316871	-0.588095	1.635121	H	-7.011364	-2.309646	-1.3448
O	-1.026442	-0.214292	1.299277	H	0.644508	-0.002052	2.499488
C	1.503058	1.169621	0.160721	H	0.33912	-1.651362	1.914252
C	2.40597	1.321124	-1.079117	H	2.161489	1.457958	0.993444
C	3.690612	0.488117	-1.076969	H	4.251324	0.780921	-0.175241
C	3.292836	-1.014605	-0.926034	H	2.715887	-1.294223	-1.811532
C	2.430945	-1.259262	0.330919	H	2.064874	-2.292581	0.263853
C	4.53338	-1.91403	-0.926115	H	6.043144	-2.84346	0.329813
C	5.117452	-2.275776	0.376804	H	3.499592	-0.124205	1.880704
C	4.56724	-1.935681	1.558144	H	2.683043	-1.546693	2.474055
C	3.267255	-1.169549	1.62562	H	6.1689	-2.783765	2.736665
C	5.20724	-2.28404	2.872026	H	5.36775	-1.382894	3.477781
C	4.541185	0.78488	-2.313958	H	4.553888	-2.94105	3.460143
O	2.14579	2.075403	-1.997236	H	5.488494	0.245586	-2.275191
O	5.039391	-2.290311	-1.974397	H	4.743425	1.856067	-2.385839
C	0.242811	2.114542	0.243795	H	4.018848	0.483911	-3.225257
C	-0.025897	2.496247	1.731227	H	-0.729965	1.861114	2.261331
C	-0.514442	3.935094	1.700983	H	0.912973	2.502291	2.299725
O	-0.187455	4.479855	0.499489	H	1.648543	3.772533	-0.119289
C	0.589276	3.545481	-0.280078	H	0.363236	3.71974	-1.326144
O	-1.087701	4.547172	2.564173	H	-3.380662	-4.242791	1.726536
O	-2.787308	-3.843355	1.076948				

2*R*-**7** (conformer 4)							
Atomic	Standard Orientation (Å)			Atomic	Standard Orientation (Å)		
Type	X	Y	Z	Type	X	Y	Z
C	-1.162963	-0.021603	-0.151771	H	-4.498486	1.980932	-0.646696
C	-1.055083	1.515372	-0.492303	H	-2.408393	2.247008	1.07809
O	0.272846	-1.278376	-1.720994	H	-2.385452	3.224294	-0.376962
N	-2.358951	-0.637422	-0.635541	H	-1.441607	-2.480585	-0.319928
C	-3.546977	0.117314	-0.741849	H	-2.795858	-2.546798	-1.421372
C	-3.56959	1.439698	-0.505585	H	-3.136959	-1.924879	1.545952
C	-2.366816	2.190929	-0.015295	H	-4.904313	-3.407107	-0.468763
C	-2.44577	-2.094587	-0.483717	H	-5.299981	-3.154427	1.229435
C	-3.302817	-2.597629	0.69838	H	-5.49751	-0.729532	0.874161
C	-4.794355	-2.698613	0.370266	H	-5.500836	0.142584	-1.524629
C	-5.50666	-1.388574	-0.004886	H	-4.614457	-1.293546	-1.993293
C	-4.805466	-0.614794	-1.151213	H	-0.028399	1.35094	-2.451919
C	-0.982612	1.663808	-2.033193	H	-1.771768	1.07275	-2.498046
C	-6.970264	-1.674992	-0.371029	H	-1.145014	2.705129	-2.321972
C	0.126997	-0.673664	-0.684051	H	-7.521545	-0.747305	-0.556096
C	1.175726	-0.355419	0.378161	H	-7.481672	-2.214685	0.432842
C	0.308421	-0.58397	1.632416	H	-7.03656	-2.288741	-1.277621
O	-1.034346	-0.2123	1.29072	H	0.632035	0.003897	2.496905
C	1.501139	1.171914	0.159554	H	0.330368	-1.646463	1.91404
C	2.405954	1.319886	-1.079244	H	2.158241	1.460612	0.993106
C	3.691023	0.487542	-1.07162	H	4.249232	0.78234	-0.169027
C	3.292743	-1.01489	-0.918536	H	2.718706	-1.296038	-1.805421
C	2.426773	-1.256841	0.33615	H	2.060713	-2.290273	0.270127
C	4.532842	-1.914917	-0.912815	H	6.038939	-2.841279	0.349672
C	5.113126	-2.273463	0.392523	H	3.490316	-0.119179	1.887423
C	4.559165	-1.930994	1.571491	H	2.671931	-1.540656	2.480572
C	3.258854	-1.164949	1.633422	H	6.156835	-2.777015	2.756794
C	5.195006	-2.276546	2.888077	H	5.354087	-1.373997	3.492098
C	4.544836	0.781382	-2.307027	H	4.539499	-2.931682	3.475841
O	2.14628	2.070007	-2.000879	H	5.49189	0.241898	-2.264628
O	5.040684	-2.294895	-1.958942	H	4.747635	1.852349	-2.380655
C	0.241766	2.117758	0.238756	H	4.024734	0.478703	-3.219035
C	-0.029176	2.501493	1.72529	H	-0.734518	1.8677	2.255401
C	-0.516503	3.940845	1.692074	H	0.908776	2.507596	2.29527
O	-0.186171	4.483677	0.490409	H	1.65058	3.773224	-0.122591
C	0.591343	3.547612	-0.285937	H	0.368497	3.720927	-1.332847
O	-1.090153	4.554997	2.553364	H	-3.068641	-4.524366	0.466015
O	-2.808185	-3.870239	1.129772				

Table S10. Energy analyses of 2*S*-**7** (one conformer).

NO.	**3D conformers** B3LYP/6-31G(d,p)	**E (Hartree)**	**ΔE (kJ/mol)**	**Boltzmann distribution**
2*S*-**7** (conformer 1)	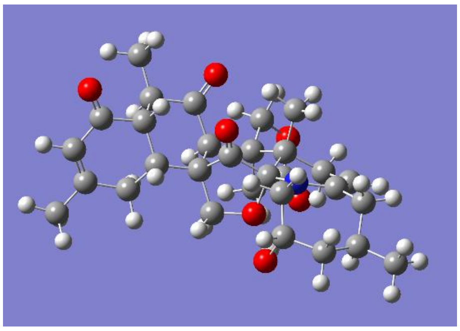	-1746.223468	0	100.00%

Table S11. Cartesian coordinates of the low-energy conformers of 2*S*-**7** re-optimized at B3LYP/6-31G(d,p) level.

2*S*-**7** (conformer 1)							
Atomic	Standard Orientation (Å)			Atomic	Standard Orientation (Å)		
Type	X	Y	Z	Type	X	Y	Z
C	-1.191124	-0.067703	-0.239092	H	-4.588905	1.83536	-0.503876
C	-1.108688	1.482955	-0.546764	H	-2.453023	2.216813	1.033936
O	0.349795	-1.176852	-1.817162	H	-2.487011	3.148759	-0.446831
N	-2.368046	-0.704145	-0.73849	H	-1.340773	-2.492664	-0.990048
C	-3.585556	0.017668	-0.750051	H	-2.837634	-2.41873	-1.871121
C	-3.628373	1.333028	-0.481813	H	-2.719232	-4.0258	0.046538
C	-2.430364	2.126701	-0.057005	H	-4.867734	-3.560971	-0.769725
C	-2.374452	-2.168316	-0.910564	H	-4.933375	-3.618221	0.988539
C	-3.047904	-2.989156	0.210992	H	-5.066714	-1.142338	1.083648
C	-4.582126	-3.015555	0.142871	H	-5.635596	0.001596	-1.204328
C	-5.308265	-1.662814	0.147212	H	-4.78905	-1.33842	-1.945808
C	-4.858893	-0.74657	-1.023005	H	-0.085831	1.36823	-2.511975
C	-1.041415	1.663339	-2.083188	H	-1.828602	1.073448	-2.554452
C	-6.82768	-1.880613	0.102207	H	-1.217873	2.708131	-2.349756
C	0.135335	-0.663069	-0.742905	H	-7.36886	-0.931923	0.181954
C	1.137456	-0.364874	0.365072	H	-7.158408	-2.525842	0.922712
C	0.240217	-0.625235	1.589841	H	-7.129296	-2.359159	-0.837695
O	-1.092171	-0.237683	1.21758	H	0.535548	-0.059527	2.478085
C	1.448076	1.168452	0.159189	H	0.239638	-1.695113	1.839488
C	2.373146	1.323567	-1.063832	H	2.083236	1.464904	1.006285
C	3.677185	0.523659	-1.017558	H	4.202583	0.831578	-0.099858
C	3.306463	-0.987272	-0.874017	H	2.768809	-1.278733	-1.779733
C	2.403893	-1.248297	0.351388	H	2.055932	-2.286908	0.269873
C	4.564357	-1.861573	-0.828482	H	6.043958	-2.766958	0.47925
C	5.106678	-2.216895	0.493576	H	3.394268	-0.102116	1.944088
C	4.506518	-1.892003	1.654669	H	2.582952	-1.542593	2.500766
C	3.190949	-1.150267	1.676368	H	6.078352	-2.714247	2.889835
C	5.102831	-2.23366	2.990545	H	5.222881	-1.332284	3.605217
C	4.557928	0.834527	-2.229532	H	4.440466	-2.905593	3.551007
O	2.108839	2.051062	-2.001797	H	5.514692	0.315361	-2.160885
O	5.115326	-2.224004	-1.858825	H	4.740168	1.909588	-2.298579
C	0.175397	2.097572	0.207819	H	4.069044	0.520721	-3.154852
C	-0.11882	2.484636	1.689198	H	-0.818842	1.844238	2.218244
C	-0.625433	3.917436	1.642082	H	0.813493	2.507654	2.267899
O	-0.285228	4.457895	0.441905	H	1.572817	3.767228	-0.129097
C	0.519817	3.529406	-0.314824	H	0.318123	3.696694	-1.366821
O	-1.221072	4.527238	2.491111	H	-2.371473	-1.679372	1.509561
O	-2.563656	-2.632433	1.497884				

Table S12. Experimental and calculated ^{1}H NMR data for compound **7**.

No.	**7**, exptl. δ_H [a]	2*R*-**7**, calcd. δ_H [b]	2*S*-**7**, calcd. δ_H [b]
1	3.23	2.66	3.34
	3.74	3.42	3.63
2	4.15	3.84	3.68
3	1.37	1.20	1.16
	2.26	1.88	1.77
4	1.73	1.74	1.67
5	2.05	2.19	2.10
7	4.26	4.54	4.58
8	1.94	1.79	1.85
	2.90	2.68	2.75
13	2.37	2.59	2.60
14	1.89	2.05	2.03
	2.84	2.71	2.70
16	6.02	6.22	6.22
18	3.47	3.19	3.29
19	2.86	2.73	2.75
21	4.23	3.69	3.72
23	3.18	2.57	2.53
	4.35	4.06	3.98
25	4.29	3.90	3.87
	5.28	5.21	5.21
26	1.10	0.88	0.89
27	1.91	2.18	2.17
28	4.10	4.20	4.23
	4.48	4.42	4.34
29	1.32	1.02	1.07
30	0.91	1.06	1.02

[a] Recorded in C_5D_5N at 600 MHz.

[b] Calculated in C_5D_5N.

Table S13. Experimental and calculated $^{13}C\{^1H\}$ NMR data for compound **7**.

No.	**7**, exptl. δ_C [a]	2*R*-**7**, calcd. δ_C [b]	2*S*-**7**, calcd. δ_C [b]
1	57.6	52.12	48.54
2	71.7	69.38	65.11
3	48.0	41.98	42.36
4	34.1	31.31	29.76
5	40.9	36.91	38.69
6	141.8	143.45	143.35
7	91.6	91.83	91.29
8	29.5	27.90	29.02
9	49.0	50.07	50.94
10	97.7	96.89	96.37
11	210.6	221.28	219.59
12	55.5	56.17	57.17
13	35.7	35.02	35.04
14	28.9	25.57	25.68
15	160.0	167.42	167.32
16	125.4	126.00	126.00
17	196.5	197.35	197.23
18	50.8	47.79	47.70
19	41.9	41.40	41.44
20	207.6	215.27	215.25
21	67.0	67.18	67.87
22	49.1	49.12	49.24
23	43.8	41.19	41.86
24	177.0	177.45	177.30
25	74.6	71.00	71.26
26	12.3	8.25	8.22
27	23.9	21.13	21.10
28	70.0	66.57	66.28
29	12.3	15.19	15.60
30	23.9	17.62	18.84

[a] Recorded in C_5D_5N at 150 MHz.

[b] Calculated in C_5D_5N.

Table S14. DP4+ analyses of calculated and experimental NMR chemical shifts of **7** (unscaled).

Isomer 1: 2*R*-**7**; Isomer 2: 2*S*-**7**.

Functional	Solvent?		Basis Set		Type of Data	
mPW1PW91	PCM		6-311G(d,p)		Unscaled Shifts	
	Isomer 1	Isomer 2	Isomer 3	Isomer 4	Isomer 5	Isomer 6
sDP4+ (H data)	1.19%	98.81%	-	-	-	-
sDP4+ (C data)	99.90%	0.10%	-	-	-	-
sDP4+ (all data)	92.39%	7.61%	-	-	-	-
uDP4+ (H data)	1.14%	98.86%	-	-	-	-
uDP4+ (C data)	45.14%	54.86%	-	-	-	-
uDP4+ (all data)	0.94%	99.06%	-	-	-	-
DP4+ (H data)	0.01%	99.99%	-	-	-	-
DP4+ (C data)	99.88%	0.12%	-	-	-	-
DP4+ (all data)	10.35%	89.65%	-	-	-	-

Functional		Solvent?		Basis Set		Type of Data	
mPW1PW91		PCM		6-311G(d,p)		Unscaled Shifts	
		DP4+	10.35%	89.65%	-	-	-
Nuclei	sp2?	Experimental	Isomer 1	Isomer 2	Isomer 3	Isomer 4	Isomer 5
C		57.6	52.12	48.54			
C		71.7	69.38	65.11			
C		48.0	41.98	42.36			
C		34.1	31.31	29.76			
C		40.9	36.91	38.69			
C	x	141.8	143.45	143.35			
C	x	91.6	91.83	91.29			
C		29.5	27.90	29.02			
C		49.0	50.07	50.94			
C		97.7	96.89	96.37			
C	x	210.6	221.28	219.59			
C		55.5	56.17	57.17			
C		35.7	35.02	35.04			
C		28.9	25.57	25.68			
C	x	160.0	167.42	167.32			
C	x	125.4	126.00	126.00			
C	x	196.5	197.35	197.23			
C		50.8	47.79	47.70			
C		41.9	41.40	41.44			
C	x	207.6	215.27	215.25			
C		67.0	67.18	67.87			
C		49.1	49.12	49.24			
C		43.8	41.19	41.86			
C	x	177.0	177.45	177.30			
C		74.6	71.00	71.26			
C		12.3	8.25	8.22			
C		23.9	21.13	21.10			
C		70.0	66.57	66.28			
C		12.3	15.19	15.60			
C		23.9	17.62	18.84			
H		3.23	2.66	3.34			
H		3.74	3.42	3.63			
H		4.15	3.84	3.68			
H		1.37	1.20	1.16			
H		2.26	1.88	1.77			
H		1.73	1.74	1.67			
H		2.05	2.19	2.10			
H	x	4.26	4.54	4.58			
H		1.94	1.79	1.85			
H		2.90	2.68	2.75			
H		2.37	2.59	2.60			
H		1.89	2.05	2.03			
H		2.84	2.71	2.70			
H	x	6.02	6.22	6.22			
H		3.47	3.19	3.29			
H		2.86	2.73	2.75			
H		4.23	3.69	3.72			
H		3.18	2.57	2.53			
H		4.35	4.06	3.98			
H		4.29	3.90	3.87			
H		5.28	5.21	5.21			
H		1.10	0.88	0.89			
H		1.91	2.18	2.17			
H		4.10	4.20	4.23			
H		4.48	4.42	4.34			
H		1.32	1.02	1.07			
H		0.91	1.06	1.02			

Table S15. Energy analyses of 2*R*-**8** (four conformers).

NO.	3D conformers B3LYP/6-31G(d,p)	E (Hartree)	ΔE (kJ/mol)	Boltzmann distribution
2*R*-**8** (conformer 1)	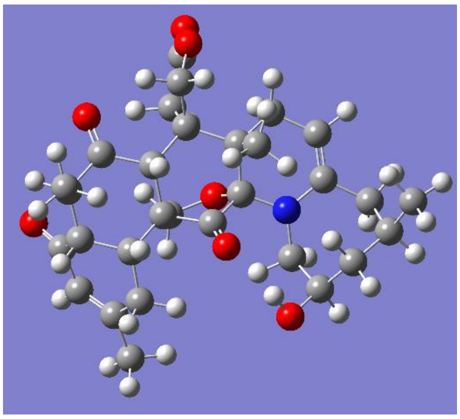	-1746.219086	2.260554505	18.40%
2*R*-**8** (conformer 2)	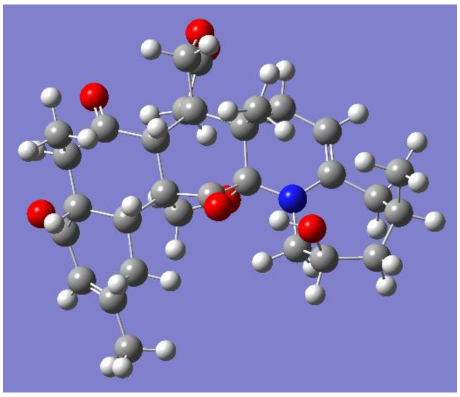	-1746.218623	3.476160471	11.27%
2*R*-**8** (conformer 3)	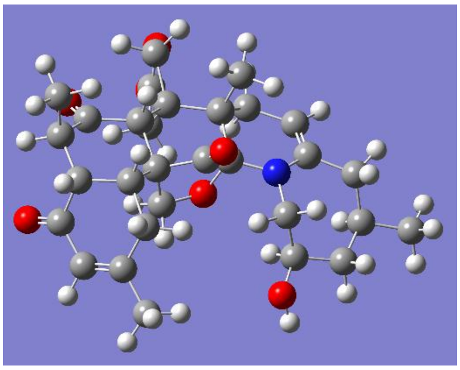	-1746.219947	0	45.81%
2*R*-**8** (conformer 4)	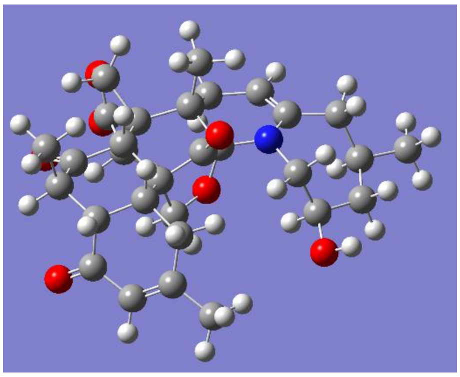	-1746.219357	1.549044319	24.52%

Table S16. Cartesian coordinates of the low-energy conformers of 2*R*-**8** re-optimized at B3LYP/6-31G(d,p) level.

2*R*-**8** (conformer 1)							
Atomic	Standard Orientation (Å)			Atomic	Standard Orientation (Å)		
Type	X	Y	Z	Type	X	Y	Z
C	1.071966	0.104393	-0.337572	H	4.163613	2.502607	-0.832411
C	0.995229	1.566603	0.25681	H	1.530226	2.639106	-1.579217
O	0.646236	-1.437644	1.543298	H	2.001867	3.454	-0.102109
N	2.40327	-0.371139	-0.535274	H	3.002501	-1.761681	-1.999904
C	3.477858	0.522892	-0.741419	H	1.57315	-2.203623	-1.113052
C	3.303807	1.85413	-0.709874	H	3.933182	-3.383985	-0.65818
C	1.939947	2.469725	-0.577603	H	3.726942	-1.267614	1.525288
C	2.561521	-1.756384	-0.997507	H	4.85354	-2.614737	1.502642
C	3.376264	-2.669064	-0.039729	H	6.152267	-1.644091	-0.276466
C	4.342184	-1.888531	0.861576	H	4.780851	-0.720924	-1.893269
C	5.392555	-0.991502	0.175786	H	5.538887	0.685184	-1.197738
C	4.826984	-0.116219	-0.979607	H	2.424542	0.898466	1.769932
C	1.537819	1.529633	1.711457	H	1.832686	2.529167	2.038115
C	6.091258	-0.117771	1.228133	H	0.814495	1.140391	2.433093
C	0.247877	-0.755434	0.621022	H	6.93766	0.425325	0.794415
C	-1.196064	-0.51999	0.218023	H	6.470287	-0.722151	2.059006
C	-1.018496	-0.507801	-1.31527	H	5.3962	0.622453	1.640577
O	0.300936	0.021837	-1.565813	H	-1.078908	-1.51769	-1.731714
C	-1.489837	0.940299	0.76582	H	-1.75458	0.114094	-1.832933
C	-3.003382	1.257319	0.702629	H	-1.304327	0.870784	1.850438
C	-3.96794	0.193498	1.223455	H	-4.938635	0.471164	0.805629
C	-3.623561	-1.221193	0.731679	H	-4.175038	-1.940004	1.358969
C	-2.129446	-1.573378	0.862353	H	-1.867395	-1.551252	1.9266
C	-4.14458	-1.483855	-0.691224	H	-4.15825	-2.983351	-2.267324
C	-3.648263	-2.70276	-1.349492	H	-0.81194	-3.221829	0.271298
C	-2.620544	-3.429944	-0.866191	H	-2.199294	-3.705533	1.193474
C	-1.883585	-3.023852	0.388994	H	-2.761591	-4.948896	-2.399456
C	-2.149392	-4.691188	-1.532525	H	-1.105724	-4.593547	-1.856818
C	-4.051381	0.284687	2.762711	H	-2.173916	-5.531309	-0.827026
O	-3.432824	2.34045	0.352368	H	-4.839731	-0.379504	3.130421
O	-4.970963	-0.754758	-1.22283	H	-4.298715	1.303319	3.074712
C	-0.531	2.058048	0.210091	H	-3.117043	-0.004634	3.255211
C	-0.937209	2.61719	-1.182172	H	-0.422968	2.165795	-2.025776
C	-0.644163	4.105633	-1.114954	H	-2.012259	2.526501	-1.343188
O	-0.468536	4.472406	0.18892	H	0.003869	3.440939	1.889264
C	-0.701859	3.357897	1.064508	H	-1.716786	3.439577	1.45547
O	-0.563398	4.893102	-2.020034	H	2.031092	-2.872752	1.343236
O	2.513418	-3.477926	0.757372				

2*R*-**8** (conformer 2)							
Atomic	Standard Orientation (Å)			Atomic	Standard Orientation (Å)		
Type	X	Y	Z	Type	X	Y	Z
C	1.056039	0.065233	-0.292084	H	4.007492	2.543989	-1.044186
C	0.929148	1.538391	0.27187	H	1.388694	2.663277	-1.555012
O	0.57643	-1.463594	1.602357	H	1.907041	3.4362	-0.074621
N	2.408822	-0.372838	-0.441788	H	2.986095	-1.992248	-1.693109
C	3.424023	0.551184	-0.783423	H	1.662769	-2.298753	-0.60615
C	3.193868	1.874035	-0.791503	H	3.409809	-3.561028	0.234038
C	1.834872	2.464356	-0.575297	H	5.662602	-2.779295	0.782197
C	2.632289	-1.801992	-0.671843	H	5.280594	-2.78073	-0.934351
C	3.598028	-2.475221	0.338413	H	6.593597	-0.889375	-0.530518
C	5.078461	-2.26085	0.012596	H	4.696577	-0.5909	-2.080478
C	5.579291	-0.808207	-0.113388	H	5.386699	0.880398	-1.440579
C	4.785696	0.009646	-1.163684	H	2.34544	0.867779	1.795166
C	1.476927	1.524065	1.726016	H	1.796114	2.522353	2.032439
C	5.708684	-0.07186	1.227517	H	0.744932	1.168456	2.457236
C	0.211319	-0.792407	0.656312	H	4.746249	0.014707	1.73261
C	-1.224988	-0.580923	0.213545	H	6.121084	0.932565	1.079826
C	-1.009739	-0.572095	-1.31538	H	6.381829	-0.61652	1.89795
O	0.319491	-0.057528	-1.541254	H	-1.068766	-1.583162	-1.728913
C	-1.546902	0.870928	0.769569	H	-1.731487	0.050518	-1.851494
C	-3.064003	1.173269	0.712579	H	-1.359578	0.797878	1.853744
C	-4.01457	0.095222	1.226072	H	-4.991305	0.370209	0.82088
C	-3.657077	-1.304199	0.703419	H	-4.204807	-2.041965	1.311711
C	-2.16056	-1.649783	0.833187	H	-1.904546	-1.647859	1.898809
C	-4.169992	-1.536782	-0.727082	H	-4.176978	-3.008595	-2.330436
C	-3.670378	-2.743347	-1.406232	H	-0.838781	-3.278235	0.208381
C	-2.643573	-3.476747	-0.931933	H	-2.221754	-3.788647	1.124383
C	-1.911365	-3.09006	0.332655	H	-2.780994	-4.972329	-2.488176
C	-2.168244	-4.725843	-1.618465	H	-1.126362	-4.617988	-1.945963
C	-4.086142	0.156904	2.767122	H	-2.188277	-5.57761	-0.92686
O	-3.503641	2.255302	0.372707	H	-4.863343	-0.523447	3.129073
O	-4.992184	-0.79707	-1.249638	H	-4.343564	1.166552	3.099435
C	-0.60622	1.998372	0.214439	H	-3.144528	-0.129175	3.247309
C	-1.026553	2.528626	-1.184928	H	-0.49193	2.087176	-2.02159
C	-0.789833	4.027932	-1.134983	H	-2.096406	2.397861	-1.35159
O	-0.619382	4.415381	0.162376	H	-0.068883	3.418919	1.858847
C	-0.795771	3.305441	1.056371	H	-1.800663	3.364211	1.475732
O	-0.74556	4.806908	-2.049957	H	2.41835	-1.898661	1.790113
O	3.368451	-2.066449	1.673149				

2*R*-**8** (conformer 3)							
Atomic	Standard Orientation (Å)			Atomic	Standard Orientation (Å)		
Type	X	Y	Z	Type	X	Y	Z
C	0.998102	0.25916	0.435559	H	3.954144	2.824869	0.137282
C	0.666128	1.783459	0.657721	H	1.591448	2.581582	-1.165006
O	0.127764	-0.930558	2.414837	H	1.630495	3.672018	0.203246
N	2.354155	-0.093416	0.721922	H	1.739027	-2.080863	0.799843
C	3.386743	0.848941	0.539966	H	3.287778	-1.797548	1.547409
C	3.12685	2.129563	0.22647	H	2.888641	-1.449273	-1.449055
C	1.741704	2.620201	-0.081054	H	5.230881	-2.458037	0.243333
C	2.666706	-1.522907	0.6875	H	5.257884	-2.317091	-1.511962
C	3.324473	-2.01405	-0.612507	H	5.078294	0.12437	-1.396634
C	4.847551	-1.862817	-0.598109	H	5.426909	1.254818	0.863759
C	5.38138	-0.422433	-0.493104	H	4.915746	-0.235167	1.630448
C	4.808689	0.364466	0.716648	H	1.650979	1.560692	2.594239
C	0.789063	2.082124	2.176412	H	0.936102	3.150338	2.350802
C	6.916137	-0.437872	-0.437533	H	-0.086604	1.76444	2.748869
C	-0.018098	-0.502844	1.291999	H	7.32342	0.578276	-0.451824
C	-1.285849	-0.528695	0.451814	H	7.33916	-0.980123	-1.289803
C	-0.662538	-0.764653	-0.938887	H	7.271945	-0.927609	0.476911
O	0.632995	-0.129394	-0.915562	H	-0.525061	-1.829952	-1.140676
C	-1.822394	0.956474	0.597338	H	-1.249969	-0.339717	-1.758427
C	-3.27139	1.073509	0.067379	H	-1.952062	1.095997	1.683613
C	-4.271132	0.000806	0.50004	H	-5.094511	0.084982	-0.213328
C	-3.704943	-1.425743	0.409256	H	-4.364084	-2.086866	0.99533
C	-2.292453	-1.563311	1.007222	H	-2.35077	-1.337115	2.078407
C	-3.782613	-1.991958	-1.019783	H	-3.164858	-3.668135	-2.259288
C	-2.989015	-3.200788	-1.29389	H	-0.751529	-3.10563	1.153129
C	-2.074112	-3.690484	-0.432362	H	-2.328383	-3.616547	1.673308
C	-1.813931	-3.026393	0.89961	H	-1.542273	-5.376885	-1.679163
C	-1.253216	-4.911822	-0.734016	H	-0.186682	-4.653135	-0.780074
C	-4.805554	0.339155	1.908854	H	-1.356522	-5.655408	0.066396
O	-3.657935	2.024311	-0.586732	H	-5.620607	-0.34339	2.169191
O	-4.507256	-1.494038	-1.871183	H	-5.200446	1.358729	1.93563
C	-0.818701	2.068889	0.110499	H	-4.03776	0.251057	2.684756
C	-0.852591	2.323739	-1.42223	H	-0.09582	1.79309	-1.992855
C	-0.685108	3.824628	-1.579671	H	-1.831865	2.082914	-1.838005
O	-0.910307	4.431697	-0.37765	H	-0.875539	3.775299	1.558794
C	-1.310804	3.46697	0.609733	H	-2.398206	3.495064	0.690204
O	-0.400116	4.445621	-2.569599	H	3.442068	-3.784037	-1.444385
O	2.956735	-3.398135	-0.703251				

2*R*-**8** (conformer 4)							
Atomic	Standard Orientation (Å)			Atomic	Standard Orientation (Å)		
Type	X	Y	Z	Type	X	Y	Z
C	0.999773	0.270026	0.447518	H	3.940902	2.84308	0.105192
C	0.657298	1.793139	0.666853	H	1.570765	2.607331	-1.155403
O	0.122098	-0.921329	2.423126	H	1.614594	3.686537	0.220845
N	2.357176	-0.073103	0.742619	H	1.749502	-2.05766	0.877893
C	3.384145	0.87087	0.536231	H	3.309626	-1.749017	1.596928
C	3.115825	2.148735	0.219209	H	2.83409	-1.537657	-1.407914
C	1.726775	2.637102	-0.072175	H	5.260799	-2.419094	0.239797
C	2.674852	-1.503126	0.735259	H	5.20684	-2.332473	-1.519443
C	3.312194	-2.041596	-0.562439	H	5.007025	0.120332	-1.438561
C	4.830344	-1.856386	-0.606657	H	5.430934	1.283306	0.801079
C	5.348463	-0.408091	-0.538015	H	4.94559	-0.199026	1.597235
C	4.810646	0.390515	0.680245	H	1.646728	1.582294	2.601269
C	0.778072	2.093933	2.184997	H	0.913504	3.163708	2.358898
C	6.884398	-0.405892	-0.537887	H	-0.093119	1.766435	2.75875
C	-0.017618	-0.496978	1.297896	H	7.278968	0.614329	-0.582842
C	-1.279174	-0.532041	0.449402	H	7.282033	-0.957415	-1.396165
C	-0.647048	-0.760019	-0.938741	H	7.278925	-0.876273	0.371061
O	0.646302	-0.120141	-0.905666	H	-0.503984	-1.823569	-1.144727
C	-1.826914	0.948731	0.597915	H	-1.23141	-0.334214	-1.759879
C	-3.27551	1.05727	0.064658	H	-1.960434	1.084179	1.684268
C	-4.268459	-0.024362	0.490454	H	-5.090864	0.056747	-0.224403
C	-3.691966	-1.44655	0.394835	H	-4.348064	-2.114576	0.976473
C	-2.280256	-1.576696	0.996133	H	-2.343415	-1.357061	2.068397
C	-3.763031	-2.007019	-1.036975	H	-3.125274	-3.668146	-2.286404
C	-2.956929	-3.206062	-1.317183	H	-0.728103	-3.107659	1.137753
C	-2.039269	-3.692668	-0.456894	H	-2.303249	-3.634577	1.648613
C	-1.790279	-3.035462	0.880866	H	-1.486126	-5.364417	-1.714098
C	-1.203894	-4.902428	-0.765515	H	-0.140325	-4.631281	-0.810629
C	-4.808569	0.304118	1.899454	H	-1.300142	-5.652527	0.029819
O	-3.667194	2.007778	-0.586741	H	-5.619198	-0.385495	2.154916
O	-4.492915	-1.512341	-1.885709	H	-5.211017	1.320637	1.9296
C	-0.82928	2.069069	0.117034	H	-4.041945	0.218371	2.676786
C	-0.861542	2.327376	-1.415183	H	-0.100565	1.802786	-1.985934
C	-0.703232	3.82988	-1.568379	H	-1.838631	2.081722	-1.833242
O	-0.935728	4.432258	-0.365285	H	-0.903242	3.771244	1.569665
C	-1.33282	3.462353	0.618216	H	-2.420662	3.482494	0.69499
O	-0.418825	4.455277	-2.555605	H	3.516191	-3.929286	-0.088896
O	2.973831	-3.427607	-0.713721		3.940902	2.84308	0.105192

Table S17. Energy analyses of 2*S*-**8** (two conformers).

**NO.**	**3D conformers** B3LYP/6-31G(d,p)	**E (Hartree)**	**ΔE (kJ/mol)**	**Boltzmann distribution**
2*S*-**8** (conformer 1)	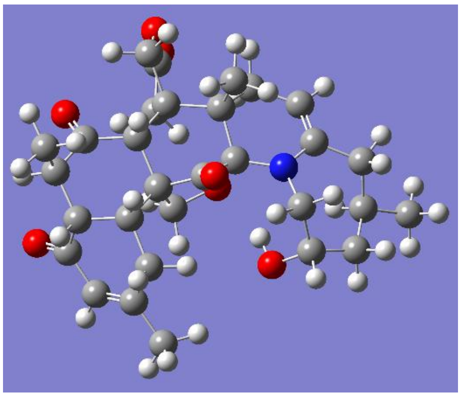	-1746.221283	0	80.97%
2*S*-**8** (conformer 2)	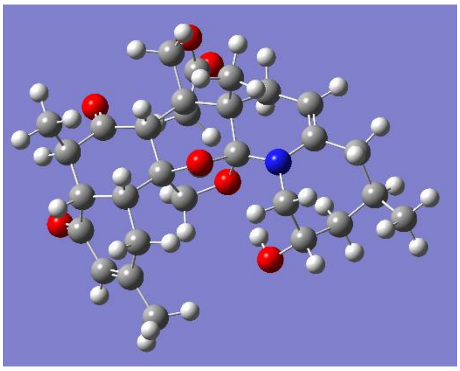	-1746.219916	3.589056921	19.03%

Table S18. Cartesian coordinates of the low-energy conformers of 2*S*-**8** re-optimized at B3LYP/6-31G(d,p) level.

2*S*-**8** (conformer 1)							
Atomic	Standard Orientation (Å)			Atomic	Standard Orientation (Å)		
Type	X	Y	Z	Type	X	Y	Z
C	1.06389	0.290175	0.535764	H	3.925894	2.799292	-0.265094
C	0.678879	1.8206	0.709989	H	1.50092	2.720174	-1.12273
O	0.065234	-0.819752	2.502575	H	1.658033	3.70777	0.304969
N	2.422389	-0.006458	0.866595	H	1.955371	-1.749447	1.903367
C	3.42563	0.901143	0.451016	H	3.59808	-1.222576	2.107376
C	3.124067	2.154195	0.077402	H	3.254308	-3.333332	0.778158
C	1.724959	2.674126	-0.053677	H	5.370838	-2.379142	0.554726
C	2.803168	-1.338724	1.363744	H	4.832028	-2.841276	-1.055222
C	3.244761	-2.350661	0.287552	H	4.350076	-0.449135	-1.583099
C	4.665845	-2.117321	-0.248978	H	5.470871	1.241456	0.102626
C	5.019355	-0.706137	-0.750636	H	5.213104	-0.002638	1.303999
C	4.839538	0.382129	0.34508	H	1.710596	1.710457	2.622932
C	0.800797	2.157422	2.218114	H	0.873205	3.236136	2.371156
C	6.459786	-0.691297	-1.283838	H	-0.035854	1.784865	2.814823
C	0.011302	-0.467819	1.34422	H	6.713276	0.281693	-1.717289
C	-1.205449	-0.543823	0.438137	H	6.602904	-1.451654	-2.05861
C	-0.529092	-0.743551	-0.929603	H	7.178318	-0.896916	-0.480841
O	0.762895	-0.094024	-0.844733	H	-0.359914	-1.800184	-1.140597
C	-1.796792	0.917377	0.601932	H	-1.08646	-0.309958	-1.764001
C	-3.243604	0.995121	0.056113	H	-1.94527	1.032161	1.688686
C	-4.207351	-0.121635	0.45675	H	-5.030537	-0.046386	-0.257773
C	-3.591698	-1.525033	0.326986	H	-4.234489	-2.226147	0.883764
C	-2.183176	-1.633026	0.939252	H	-2.264339	-1.449575	2.016676
C	-3.636344	-2.045041	-1.120959	H	-2.941422	-3.648516	-2.413024
C	-2.793392	-3.209972	-1.429667	H	-0.566785	-3.100858	1.015606
C	-1.863347	-3.691803	-0.579724	H	-2.114845	-3.711864	1.529591
C	-1.635845	-3.067078	0.777494	H	-1.250344	-5.308095	-1.8805
C	-0.978823	-4.855282	-0.924367	H	0.067669	-4.525505	-0.97357
C	-4.757608	0.158472	1.872209	H	-1.028803	-5.626497	-0.145329
O	-3.655543	1.944737	-0.583687	H	-5.549088	-0.55926	2.109514
O	-4.373952	-1.545071	-1.96013	H	-5.188583	1.16229	1.92582
C	-0.81704	2.061952	0.147097	H	-3.99025	0.075544	2.649038
C	-0.84707	2.329657	-1.383651	H	-0.077486	1.821302	-1.958193
C	-0.710684	3.836502	-1.524729	H	-1.820106	2.072949	-1.804531
O	-0.961018	4.424667	-0.318556	H	-0.961086	3.7497	1.612787
C	-1.360544	3.439469	0.649154	H	-2.449996	3.434884	0.699126
O	-0.427096	4.473035	-2.504912	H	2.012711	-1.565769	-1.023698
O	2.267732	-2.463787	-0.74379				

2*S*-**8** (conformer 2)							
Atomic	Standard Orientation (Å)			Atomic	Standard Orientation (Å)		
Type	X	Y	Z	Type	X	Y	Z
C	1.081171	0.246339	0.433269	H	4.009869	2.825339	-0.022066
C	0.740307	1.776597	0.630109	H	1.613197	2.550792	-1.229585
O	0.214614	-0.909402	2.430599	H	1.680166	3.660792	0.120586
N	2.439609	-0.081494	0.712549	H	1.951879	-2.057205	1.206521
C	3.464843	0.867011	0.478087	H	3.577896	-1.591337	1.613683
C	3.189148	2.132256	0.12498	H	3.786026	-3.107451	-0.170219
C	1.791957	2.605688	-0.150808	H	4.714552	-1.887944	-2.05372
C	2.811401	-1.498679	0.839816	H	3.67793	-0.508007	-1.748562
C	3.300463	-2.166996	-0.458635	H	5.907528	0.05755	-1.198028
C	4.275731	-1.282713	-1.252753	H	5.53351	1.249825	0.71332
C	5.400587	-0.580488	-0.462808	H	4.976708	-0.118768	1.638507
C	4.880437	0.37446	0.663632	H	1.771642	1.588024	2.546617
C	0.898693	2.099905	2.140489	H	1.046907	3.171045	2.293413
C	6.447061	-1.556646	0.092633	H	0.037411	1.78952	2.738136
C	0.066876	-0.505322	1.299184	H	7.264767	-1.018564	0.584527
C	-1.211101	-0.535073	0.474322	H	6.882241	-2.167461	-0.705341
C	-0.618586	-0.7686	-0.927273	H	6.013191	-2.237852	0.834215
O	0.695805	-0.155974	-0.924962	H	-0.494595	-1.832749	-1.13482
C	-1.748659	0.947098	0.635265	H	-1.21033	-0.328685	-1.734364
C	-3.213121	1.059445	0.147241	H	-1.846797	1.088324	1.724376
C	-4.195332	-0.012218	0.618833	H	-5.048646	0.076001	-0.057804
C	-3.632466	-1.437893	0.491412	H	-4.270782	-2.105674	1.09264
C	-2.200501	-1.582036	1.039794	H	-2.224629	-1.377807	2.116493
C	-3.764149	-1.984621	-0.941912	H	-3.166862	-3.618791	-2.24415
C	-2.965191	-3.174386	-1.273051	H	-0.645877	-3.115905	1.099153
C	-2.010024	-3.669611	-0.459354	H	-2.203999	-3.652293	1.655264
C	-1.716647	-3.038861	0.88243	H	-1.494725	-5.31669	-1.763943
C	-1.164498	-4.853531	-0.831561	H	-0.116721	-4.543219	-0.945678
C	-4.667584	0.315923	2.051895	H	-1.182962	-5.611192	-0.038144
O	-3.618109	2.003672	-0.504912	H	-5.471138	-0.368324	2.34169
O	-4.532416	-1.482125	-1.751487	H	-5.060791	1.335245	2.103757
C	-0.758799	2.055456	0.115863	H	-3.867594	0.221667	2.793705
C	-0.832459	2.299607	-1.417449	H	-0.092556	1.765516	-2.006879
C	-0.665921	3.799512	-1.590141	H	-1.823786	2.058993	-1.80396
O	-0.861747	4.415091	-0.387504	H	-0.78719	3.773694	1.552679
C	-1.240847	3.457678	0.615101	H	-2.326334	3.484792	0.717152
O	-0.402668	4.411722	-2.591118	H	1.755515	-1.723841	-1.524026
O	2.204691	-2.554574	-1.288696				

Table S19. Experimental and calculated ^{1}H NMR data for compound **8**.

No.	**8**, exptl. δ_H [a]	2*R*-**8**, calcd. δ_H [b]	2*S*-**8**, calcd. δ_H [b]
1	3.13	2.60	3.39
	3.88	3.49	3.83
2	4.09	3.85	3.65
3	2.23	1.24	1.32
	2.26	1.88	1.76
4	1.74	1.70	1.73
5	2.12	2.17	2.17
7	4.32	4.48	4.62
8	1.64	1.70	1.80
	2.76	2.66	2.78
13	3.43	3.53	3.63
14	2.19	2.33	2.19
	2.36	2.90	2.88
16	5.90	6.26	6.26
18	2.96	2.75	2.76
19	3.26	3.59	3.61
21	3.54	2.90	2.91
23	3.73	3.40	3.26
	3.83	3.54	3.55
25	4.18	4.22	4.28
	4.45	4.23	4.29
26	1.25	1.33	1.34
27	1.66	2.11	2.14
28	4.03	3.78	3.72
	4.25	3.91	3.89
29	0.96	0.84	0.93
30	0.93	1.05	1.02

[a] Recorded in C_5D_5N at 400 MHz.

[b] Calculated in C_5D_5N.

Table S20. Experimental and calculated $^{13}C\{^{1}H\}$ NMR data for compound **8**.

No.	**8**, exptl. δ_C [a]	2*R*-**8**, calcd. δ_C [b]	2*S*-**8**, calcd. δ_C [b]
1	58.0	52.28	46.29
2	71.8	69.49	66.12
3	48.1	41.95	40.83
4	34.0	30.96	28.96
5	40.8	36.58	38.80
6	142.1	143.17	142.74
7	90.4	89.96	91.80
8	26.4	26.13	27.99
9	49.0	48.95	50.44
10	96.7	94.55	93.76
11	208.5	219.32	217.43
12	55.7	56.95	58.03
13	31.9	30.95	31.04
14	31.3	30.33	30.56
15	160.6	168.90	169.90
16	126.1	127.06	127.17
17	196.6	195.66	195.66
18	49.7	48.08	47.91
19	43.6	42.95	43.09
20	210.3	216.93	217.31
21	56.2	53.74	54.56
22	47.0	48.93	49.27
23	34.3	31.14	31.97
24	177.9	178.51	178.64
25	73.6	68.02	68.58
26	16.7	15.09	15.06
27	23.6	20.83	20.86
28	66.5	63.38	63.64
29	18.9	16.89	17.55
30	23.8	17.99	18.32

[a] Recorded in C_5D_5N at 100 MHz.

[b] Calculated in C_5D_5N.

Table S21. DP4+ analyses of calculated and experimental NMR chemical shifts of **8** (unscaled).

Isomer 1: 2*R*-**8**; Isomer 2: 2*S*-**8**.

Functional	Solvent?	Basis Set	Type of Data
mPW1PW91	PCM	6-311G(d,p)	Unscaled Shifts

	Isomer 1	Isomer 2	Isomer 3	Isomer 4	Isomer 5	Isomer 6
sDP4+ (H data)	67.39%	32.61%	-	-	-	-
sDP4+ (C data)	100.00%	0.00%	-	-	-	-
sDP4+ (all data)	100.00%	0.00%	-	-	-	-
uDP4+ (H data)	11.93%	88.07%	-	-	-	-
uDP4+ (C data)	14.91%	85.09%	-	-	-	-
uDP4+ (all data)	2.32%	97.68%	-	-	-	-
DP4+ (H data)	21.86%	78.14%	-	-	-	-
DP4+ (C data)	100.00%	0.00%	-	-	-	-
DP4+ (all data)	99.99%	0.01%	-	-	-	-

Functional	Solvent?	Basis Set	Type of Data
mPW1PW91	PCM	6-311G(d,p)	Unscaled Shifts

		DP4+	99.99%	0.01%	-	-	-
Nuclei	sp2?	Experimental	Isomer 1	Isomer 2	Isomer 3	Isomer 4	Isomer 5
C		58.00	52.28	46.29			
C		71.80	69.49	66.12			
C		48.10	41.95	40.83			
C		34.00	30.96	28.96			
C		40.80	36.58	38.80			
C	x	142.10	143.17	142.74			
C	x	90.40	89.96	91.80			
C		26.40	26.13	27.99			
C		49.00	48.95	50.44			
C		96.70	94.55	93.76			
C	x	208.50	219.32	217.43			
C		55.70	56.95	58.03			
C		31.90	30.95	31.04			
C		31.30	30.33	30.56			
C	x	160.60	168.90	169.90			
C	x	126.10	127.06	127.17			
C	x	196.60	195.66	195.66			
C		49.70	48.08	47.91			
C		43.60	42.95	43.09			
C	x	210.30	216.93	217.31			
C		56.20	53.74	54.56			
C		47.00	48.93	49.27			
C		34.30	31.14	31.97			
C	x	177.90	178.51	178.64			
C		73.60	68.02	68.58			
C		16.70	15.09	15.06			
C		23.60	20.83	20.86			
C		66.50	63.38	63.64			
C		18.90	16.89	17.55			
C		23.80	17.99	18.32			
H		3.13	2.60	3.39			
H		3.88	3.49	3.83			
H		4.09	3.85	3.65			
H		2.23	1.24	1.32			
H		2.26	1.88	1.76			
H		1.74	1.70	1.73			
H		2.12	2.17	2.17			
H	x	4.32	4.48	4.62			
H		1.64	1.70	1.80			
H		2.76	2.66	2.78			
H		3.43	3.53	3.63			
H		2.19	2.33	2.19			
H		2.36	2.90	2.88			
H	x	5.90	6.26	6.26			
H		2.96	2.75	2.76			
H		3.26	3.59	3.61			
H		3.54	2.90	2.91			
H		3.73	3.40	3.26			
H		3.83	3.54	3.55			
H		4.18	4.22	4.28			
H		4.45	4.23	4.29			
H		1.25	1.33	1.34			
H		1.66	2.11	2.14			
H		4.03	3.78	3.72			
H		4.25	3.91	3.89			
H		0.96	0.84	0.93			
H		0.93	1.05	1.02			

Table S22. The effect of compounds **1**–**16** on ND7/23 DRG neurons viability.

Compound	Viability (%)[a]	
	1 μM	10 μM
**1**	95.1 ± 1.1	95.6 ± 1.4
**2**	118.7 ± 4.9	112.8 ± 3.5
**3**	94.4 ± 2.4	100.9 ± 4.8
**4**	99.3 ± 3.1	97.9 ± 1.6
**5**	91.3 ± 4.2	89.2 ± 1.9
**6**	103.0 ± 1.6	112.1 ± 2.9
**7**	102.5 ± 1.6	90.1 ± 4.2
**8**	91.0 ± 5.3	95.0 ± 1.7
**9**	96.5 ± 5.2	103.1 ± 4.8
**10**	114.8 ± 5.7	97.6 ± 5.0
**11**	120.0 ± 1.1	121.7 ± 1.4
**12**	108.2 ± 4.0	102.4 ± 1.0
**13**	106.4 ± 1.8	94.2 ± 2.2
**14**	99.1 ± 3.1	99.1 ± 4.5
**15**	98.7 ± 0.7	100.5 ± 6.1
**16**	108.0 ± 1.1	97.4 ± 1.1

[a]ND7/23 DRG neurons were treated with control or candidate compounds for 72 hours and cell viabilities were examined with the resazurin-based assay. Data was normalized to the solvent control group. Values were presented as the mean ± SEM from at least 3 replicate wells.

Table S23. Neuroprotective potentials and anticancer interfering effects of compounds **1–16**.

Treatment	Conc. (μM)	Total neurite outgrowth of ND7/23 DRG neurons (%)[a]	Cell viability of cervical cancer SiHa cells (%)[b]
Control	--	100.0 ± 0.2 ***	100.0 ± 0.8 ***
Paclitaxel	10[c]	54.6 ± 0.1	40.5 ± 0.8
Cotreatment with paclitaxel			
**1**	1	51.6 ± 2.1	32.6 ± 2.5 **
	10	56.6 ± 0.7	29.4 ± 1.5 ***
**2**	1	59.6 ± 0.5	36.5 ± 2.2
	10	66.4 ± 2.3 ***	33.2 ± 1.8 **
**3**	1	50.8 ± 1.2	40.0 ± 2.7
	10	73.6 ± 0.3 ***	43.1 ± 2.8
**4**	1	60.1 ± 1.8	43.7 ± 2.0
	10	59.2 ± 2.1	45.8 ± 1.4
**5**	1	52.2 ± 1.2	34.8 ± 2.0 *
	10	51.8 ± 1.9	32.9 ± 1.1 **
**6**	1	48.0 ± 1.3	34.0 ± 2.0 *
	10	43.9 ± 1.4 **	36.5 ± 1.8
**7**	1	54.2 ± 1.2	32.3 ± 3.0
	10	56.8 ± 2.2	32.3 ± 2.9 *
**8**	1	66.2 ± 3.3	31.8 ± 2.3 ***
	10	71.2 ± 0.5 ***	29.4 ± 2.1 ***
**9**	1	69.3 ± 0.3 ***	40.4 ± 1.6
	10	55.2 ± 1.8	41.2 ± 0.9
**10**	1	63.2 ± 1.3 *	34.0 ± 1.2 *
	10	49.7 ± 1.4	35.5 ± 1.7
**11**	1	51.7 ± 1.4	36.0 ± 1.8
	10	39.4 ± 1.6 ***	29.8 ± 1.6 ***
**12**	1	57.8 ± 1.2 *	31.7 ± 2.1 ***
	10	49.8 ± 1.1	27.9 ± 1.7 ***
**13**	1	52.1 ± 4.1	33.8 ± 1.4 **
	10	33.8 ± 0.9 **	35.4 ± 1.5
**14**	1	57.1 ± 0.8	40.0 ± 1.9
	10	46.3 ± 0.4 *	38.4 ± 2.2
**15**	1	60.0 ± 2.6	33.0 ± 2.1 **
	10	70.3 ± 1.9 ***	35.7 ± 2.1
**16**	1	43.1 ± 2.9 *	38.5 ± 1.1
	10	38.9 ± 1.7	37.5 ± 1.3

[a]The well-differentiated ND7/23 DRG neurons were pretreated with or without candidate compounds for 1 day and then treated with paclitaxel for another 2 days. Cells were fixed for the staining for neurofilament and nucleus. High-content analyses of the total neurite outgrowth were performed based on the neurofilament staining. Data was normalized to the solvent control group. Values were presented as the mean ± SEM from quadruple treatments. *P<0.05; **P < 0.01; ***P < 0.001 versus paclitaxel group, by one-way ANOVA *post Tukey or Games Howell.* [b]The SiHa cervical cancer cells were pretreated with or without candidate compounds for 24 h and then treated with paclitaxel for another 48 h and cell viabilities were examined with the resazurin-based assay. Data was normalized to the solvent control group. Values were presented as the mean ± SEM from at least 12 replicate wells. ***P < 0.001 versus paclitaxel group, by one-way ANOVA *post Tukey and Games Howell hoc test.* [c]The unit for the concentration of paclitaxel was nM.

Table S24. Crystal data and experimental details for **1**

	Crystal data	
Empirical formula	$C_{32} H_{47} N O_9$	
Formula weight	589.70	
Crystal system	Monoclinic	
Space group	$P2_1$	
Unit cell dimensions	a = 13.1044(6) Å	α = 90°.
	b = 7.6452(5) Å	β = 97.943(4)°.
	c = 14.5480(6) Å	γ = 90°.
Volume	1443.52(13) $Å^3$	
Z	2	
F(000)	636	
Density (calculated)	1.357 Mg/m^3	
Wavelength	1.54178 Å	
Cell parameters reflections used	7017	
Theta range for Cell parameters	4.1960 to 78.5260°.	
Absorption coefficient	0.806 mm^{-1}	
Temperature	100(2) K	
Crystal size	0.35 x 0.25 x 0.20 mm^3	
	Data collection	
Diffractometer	Xcalibur, Atlas, Gemini	
Absorption correction	Semi-empirical from equivalents	
Max. and min. transmission	1.00000 and 0.62155	
No. of measured reflections	12830	
No. of independent reflections	4992 [R(int) = 0.0504]	
No. of observed [I>2_igma(I)]	4690	
Completeness to theta = 67.679°	100.0 %	
Theta range for data collection	3.067 to 67.969°.	
	Refinement	
Final R indices [I>2sigma(I)]	R1 = 0.0471, wR2 = 0.1284	
R indices (all data)	R1 = 0.0503, wR2 = 0.1321	
Goodness-of-fit on F^2	1.055	
No. of reflections	4992	
No. of parameters	385	
No. of restraints	1	
Absolute structure parameter	-0.12(11)	
Largest diff. peak and hole	0.379 and -0.436 e.$Å^{-3}$	

Table S25. Bond lengths [Å] and angles [°] for **1**

O(1)-C(6)	1.426(4)
O(1)-C(2)	1.458(4)
O(2)-C(10)	1.230(4)
O(3)-C(24)	1.196(4)
O(4)-C(24)	1.363(4)
O(4)-C(25)	1.459(4)
O(5)-C(11)	1.208(4)
O(6)-C(20)	1.212(4)
O(7)-C(17)	1.223(4)
N(1)-C(10)	1.353(4)
N(1)-C(1)	1.470(4)
N(1)-C(6)	1.487(4)
C(1)-C(2)	1.539(4)
C(2)-C(3)	1.519(5)
C(3)-C(4)	1.532(5)
C(4)-C(5)	1.528(5)
C(4)-C(30)	1.529(5)
C(5)-C(6)	1.533(4)
C(6)-C(7)	1.501(5)
C(7)-C(8)	1.533(4)
C(8)-C(9)	1.553(4)
C(9)-C(10)	1.538(5)
C(9)-C(29)	1.548(5)
C(9)-C(22)	1.604(4)
C(11)-C(12)	1.531(4)
C(12)-C(28)	1.526(5)
C(12)-C(21)	1.577(4)
C(12)-C(13)	1.578(5)
C(13)-C(18)	1.541(4)
C(13)-C(14)	1.545(4)
C(14)-C(15)	1.497(5)
C(15)-C(16)	1.336(5)
C(15)-C(27)	1.506(5)
C(16)-C(17)	1.465(5)
C(17)-C(18)	1.532(5)
C(18)-C(19)	1.545(4)
C(19)-C(20)	1.524(5)

C(19)-C(26)	1.535(5)
C(20)-C(21)	1.540(4)
C(21)-C(22)	1.582(5)
C(22)-C(23)	1.553(4)
C(22)-C(25)	1.554(5)
C(23)-C(24)	1.510(5)
O(8)-C(31)	1.419(5)
O(9)-C(32)	1.428(5)

C(6)-O(1)-C(2)	103.6(2)
C(24)-O(4)-C(25)	109.8(3)
C(10)-N(1)-C(1)	119.0(3)
C(10)-N(1)-C(6)	124.8(3)
C(1)-N(1)-C(6)	108.5(2)
N(1)-C(1)-C(2)	102.1(3)
O(1)-C(2)-C(3)	109.1(3)
O(1)-C(2)-C(1)	101.7(3)
C(3)-C(2)-C(1)	113.7(3)
C(2)-C(3)-C(4)	111.1(3)
C(5)-C(4)-C(30)	111.1(3)
C(5)-C(4)-C(3)	109.6(3)
C(30)-C(4)-C(3)	111.7(3)
C(4)-C(5)-C(6)	112.8(3)
O(1)-C(6)-N(1)	101.5(2)
O(1)-C(6)-C(7)	110.8(3)
N(1)-C(6)-C(7)	110.2(3)
O(1)-C(6)-C(5)	107.9(3)
N(1)-C(6)-C(5)	111.3(3)
C(7)-C(6)-C(5)	114.5(3)
C(6)-C(7)-C(8)	109.1(3)
C(7)-C(8)-C(9)	111.6(3)
C(10)-C(9)-C(29)	105.2(2)
C(10)-C(9)-C(8)	111.2(3)
C(29)-C(9)-C(8)	108.3(3)
C(10)-C(9)-C(22)	109.4(3)
C(29)-C(9)-C(22)	111.6(3)
C(8)-C(9)-C(22)	110.9(2)
O(2)-C(10)-N(1)	120.2(3)
O(2)-C(10)-C(9)	120.5(3)
N(1)-C(10)-C(9)	119.3(3)

O(5)-C(11)-C(12)	124.7(3)
C(28)-C(12)-C(11)	112.7(3)
C(28)-C(12)-C(21)	113.8(3)
C(11)-C(12)-C(21)	108.2(3)
C(28)-C(12)-C(13)	112.3(3)
C(11)-C(12)-C(13)	101.1(3)
C(21)-C(12)-C(13)	107.9(2)
C(18)-C(13)-C(14)	109.6(3)
C(18)-C(13)-C(12)	112.6(3)
C(14)-C(13)-C(12)	112.5(3)
C(15)-C(14)-C(13)	113.2(3)
C(16)-C(15)-C(14)	122.5(3)
C(16)-C(15)-C(27)	122.0(3)
C(14)-C(15)-C(27)	115.5(3)
C(15)-C(16)-C(17)	122.3(3)
O(7)-C(17)-C(16)	121.3(3)
O(7)-C(17)-C(18)	121.0(3)
C(16)-C(17)-C(18)	117.5(3)
C(17)-C(18)-C(13)	112.1(3)
C(17)-C(18)-C(19)	111.0(3)
C(13)-C(18)-C(19)	112.8(3)
C(20)-C(19)-C(26)	112.1(3)
C(20)-C(19)-C(18)	106.5(3)
C(26)-C(19)-C(18)	114.7(3)
O(6)-C(20)-C(19)	120.5(3)
O(6)-C(20)-C(21)	124.1(3)
C(19)-C(20)-C(21)	115.3(3)
C(20)-C(21)-C(12)	106.4(2)
C(20)-C(21)-C(22)	114.4(3)
C(12)-C(21)-C(22)	119.4(3)
C(23)-C(22)-C(25)	98.4(3)
C(23)-C(22)-C(21)	115.1(3)
C(25)-C(22)-C(21)	114.6(3)
C(23)-C(22)-C(9)	110.9(3)
C(25)-C(22)-C(9)	110.1(3)
C(21)-C(22)-C(9)	107.5(3)
C(24)-C(23)-C(22)	106.0(3)
O(3)-C(24)-O(4)	121.0(3)
O(3)-C(24)-C(23)	129.9(3)
O(4)-C(24)-C(23)	109.0(3)

O(4)-C(25)-C(22)	107.4(2)

Symmetry transformations used to generate equivalent atoms:

Table S26. Crystal data and experimental details for **3**

	Crystal data	
Empirical formula	C_{30} H_{37} Cl N O_7	
Formula weight	559.05	
Crystal system	Orthorhombic	
Space group	$P2_12_12_1$	
Unit cell dimensions	a = 7.2186(3) Å	α = 90°.
	b = 18.6256(8) Å	β = 90°.
	c = 43.1681(18) Å	γ = 90°.
Volume	5804.0(4) $Å^3$	
Z	8	
F(000)	2376	
Density (calculated)	1.280 Mg/m^3	
Wavelength	1.54178 Å	
Cell parameters reflections used	9879	
Theta range for Cell parameters	2.58 to 79.09°.	
Absorption coefficient	1.552 mm^{-1}	
Temperature	100(2) K	
Crystal size	0.250 x 0.200 x 0.025 mm^3	
	Data collection	
Diffractometer	Bruker AXS D8 VENTURE, PhotonIII_C28	
Absorption correction	Semi-empirical from equivalents	
Max. and min. transmission	1.0000 and 0.8516	
No. of measured reflections	60135	
No. of independent reflections	10517 [R(int) = 0.0391]	
No. of observed [I>2_igma(I)]	9996	
Completeness to theta = 67.679°	99.2 %	
Theta range for data collection	2.047 to 67.995°.	
	Refinement	
Final R indices [I>2sigma(I)]	R1 = 0.0365, wR2 = 0.1140	
R indices (all data)	R1 = 0.0393, wR2 = 0.1179	
Goodness-of-fit on F^2	1.041	
No. of reflections	10517	
No. of parameters	720	
No. of restraints	0	
Absolute structure parameter	0.017(5)	
Largest diff. peak and hole	0.315 and -0.600 e.$Å^{-3}$	

Table S27. Bond lengths [Å] and angles [°] for **3**

O(1)-C(6)	1.424(3)
O(1)-C(2)	1.449(3)
O(2)-C(11)	1.209(3)
O(3)-C(17)	1.229(3)
O(4)-C(20)	1.210(3)
O(5)-C(24)	1.206(3)
O(6)-C(24)	1.347(3)
O(6)-C(25)	1.457(3)
O(7)-C(28)	1.443(3)
O(7)-C(10)	1.466(3)
N(1)-C(10)	1.399(3)
N(1)-C(1)	1.468(3)
N(1)-C(6)	1.471(3)
C(1)-C(2)	1.545(4)
C(2)-C(3)	1.518(4)
C(3)-C(4)	1.537(4)
C(4)-C(30)	1.526(4)
C(4)-C(5)	1.531(4)
C(5)-C(6)	1.546(3)
C(6)-C(7)	1.504(4)
C(7)-C(8)	1.541(4)
C(8)-C(9)	1.551(3)
C(9)-C(29)	1.555(3)
C(9)-C(10)	1.574(3)
C(9)-C(22)	1.582(3)
C(10)-C(11)	1.521(3)
C(11)-C(12)	1.518(3)
C(12)-C(28)	1.536(3)
C(12)-C(13)	1.532(3)
C(12)-C(21)	1.571(3)
C(13)-C(14)	1.534(3)
C(13)-C(18)	1.540(3)
C(14)-C(15)	1.504(3)
C(15)-C(16)	1.349(4)
C(15)-C(27)	1.502(4)
C(16)-C(17)	1.460(4)
C(17)-C(18)	1.510(4)

C(18)-C(19)	1.552(3)
C(19)-C(26)	1.521(3)
C(19)-C(20)	1.518(3)
C(20)-C(21)	1.543(3)
C(21)-C(22)	1.572(3)
C(22)-C(25)	1.554(3)
C(22)-C(23)	1.564(3)
C(23)-C(24)	1.506(4)
O(8)-C(36)	1.428(3)
O(8)-C(32)	1.449(3)
O(9)-C(41)	1.209(3)
O(10)-C(47)	1.233(3)
O(11)-C(50)	1.215(3)
O(12)-C(54)	1.203(4)
O(13)-C(54)	1.348(4)
O(13)-C(55)	1.458(3)
O(14)-C(58)	1.441(3)
O(14)-C(40)	1.471(3)
N(2)-C(40)	1.399(3)
N(2)-C(31)	1.466(3)
N(2)-C(36)	1.479(3)
C(31)-C(32)	1.548(4)
C(32)-C(33)	1.520(4)
C(33)-C(34)	1.532(4)
C(34)-C(35)	1.523(5)
C(34)-C(60)	1.524(4)
C(35)-C(36)	1.545(4)
C(36)-C(37)	1.502(4)
C(37)-C(38)	1.544(4)
C(38)-C(39)	1.545(4)
C(39)-C(59)	1.545(4)
C(39)-C(40)	1.573(4)
C(39)-C(52)	1.587(3)
C(40)-C(41)	1.516(3)
C(41)-C(42)	1.528(3)
C(42)-C(58)	1.535(3)
C(42)-C(43)	1.533(3)
C(42)-C(51)	1.571(3)
C(43)-C(48)	1.537(3)
C(43)-C(44)	1.541(3)

C(44)-C(45)	1.498(4)
C(45)-C(46)	1.351(4)
C(45)-C(57)	1.499(4)
C(46)-C(47)	1.456(4)
C(47)-C(48)	1.507(4)
C(48)-C(49)	1.554(3)
C(49)-C(50)	1.514(4)
C(49)-C(56)	1.519(3)
C(50)-C(51)	1.539(3)
C(51)-C(52)	1.562(4)
C(52)-C(55)	1.553(4)
C(52)-C(53)	1.561(3)
C(53)-C(54)	1.506(4)
Cl(1)-C(61)	1.765(4)
Cl(2)-C(61)	1.764(4)

C(6)-O(1)-C(2)	103.50(19)
C(24)-O(6)-C(25)	109.94(19)
C(28)-O(7)-C(10)	110.05(16)
C(10)-N(1)-C(1)	123.0(2)
C(10)-N(1)-C(6)	120.24(19)
C(1)-N(1)-C(6)	108.11(19)
N(1)-C(1)-C(2)	101.5(2)
O(1)-C(2)-C(3)	108.1(2)
O(1)-C(2)-C(1)	103.5(2)
C(3)-C(2)-C(1)	112.2(2)
C(2)-C(3)-C(4)	110.7(2)
C(30)-C(4)-C(5)	111.1(3)
C(30)-C(4)-C(3)	110.5(2)
C(5)-C(4)-C(3)	110.8(2)
C(4)-C(5)-C(6)	113.4(2)
O(1)-C(6)-N(1)	100.18(18)
O(1)-C(6)-C(7)	111.7(2)
N(1)-C(6)-C(7)	108.7(2)
O(1)-C(6)-C(5)	107.0(2)
N(1)-C(6)-C(5)	116.1(2)
C(7)-C(6)-C(5)	112.5(2)
C(6)-C(7)-C(8)	109.4(2)
C(7)-C(8)-C(9)	112.9(2)
C(8)-C(9)-C(29)	107.1(2)

C(8)-C(9)-C(10)	108.20(19)
C(29)-C(9)-C(10)	107.59(19)
C(8)-C(9)-C(22)	113.17(19)
C(29)-C(9)-C(22)	111.5(2)
C(10)-C(9)-C(22)	109.01(19)
N(1)-C(10)-O(7)	112.37(19)
N(1)-C(10)-C(11)	114.8(2)
O(7)-C(10)-C(11)	102.07(18)
N(1)-C(10)-C(9)	110.73(19)
O(7)-C(10)-C(9)	111.02(19)
C(11)-C(10)-C(9)	105.41(18)
O(2)-C(11)-C(12)	128.8(2)
O(2)-C(11)-C(10)	126.5(2)
C(12)-C(11)-C(10)	104.63(19)
C(11)-C(12)-C(28)	98.68(19)
C(11)-C(12)-C(13)	113.95(19)
C(28)-C(12)-C(13)	113.0(2)
C(11)-C(12)-C(21)	105.25(18)
C(28)-C(12)-C(21)	110.77(19)
C(13)-C(12)-C(21)	113.94(19)
C(14)-C(13)-C(12)	112.91(19)
C(14)-C(13)-C(18)	111.2(2)
C(12)-C(13)-C(18)	110.76(19)
C(15)-C(14)-C(13)	113.5(2)
C(16)-C(15)-C(14)	121.7(2)
C(16)-C(15)-C(27)	121.8(2)
C(14)-C(15)-C(27)	116.4(2)
C(15)-C(16)-C(17)	122.8(2)
O(3)-C(17)-C(16)	121.8(2)
O(3)-C(17)-C(18)	121.2(2)
C(16)-C(17)-C(18)	117.0(2)
C(17)-C(18)-C(13)	109.9(2)
C(17)-C(18)-C(19)	111.5(2)
C(13)-C(18)-C(19)	110.6(2)
C(26)-C(19)-C(20)	110.9(2)
C(26)-C(19)-C(18)	113.5(2)
C(20)-C(19)-C(18)	107.72(19)
O(4)-C(20)-C(19)	121.4(2)
O(4)-C(20)-C(21)	122.9(2)
C(19)-C(20)-C(21)	115.7(2)

C(20)-C(21)-C(12)	109.37(19)
C(20)-C(21)-C(22)	116.55(19)
C(12)-C(21)-C(22)	114.41(19)
C(25)-C(22)-C(23)	97.72(19)
C(25)-C(22)-C(21)	110.3(2)
C(23)-C(22)-C(21)	110.84(19)
C(25)-C(22)-C(9)	112.5(2)
C(23)-C(22)-C(9)	112.2(2)
C(21)-C(22)-C(9)	112.39(19)
C(24)-C(23)-C(22)	104.8(2)
O(5)-C(24)-O(6)	121.3(2)
O(5)-C(24)-C(23)	129.2(3)
O(6)-C(24)-C(23)	109.6(2)
O(6)-C(25)-C(22)	106.9(2)
O(7)-C(28)-C(12)	106.78(18)
C(36)-O(8)-C(32)	102.72(19)
C(54)-O(13)-C(55)	110.0(2)
C(58)-O(14)-C(40)	110.11(17)
C(40)-N(2)-C(31)	122.4(2)
C(40)-N(2)-C(36)	119.2(2)
C(31)-N(2)-C(36)	107.6(2)
N(2)-C(31)-C(32)	101.4(2)
O(8)-C(32)-C(33)	108.7(2)
O(8)-C(32)-C(31)	104.2(2)
C(33)-C(32)-C(31)	112.2(2)
C(32)-C(33)-C(34)	109.0(2)
C(35)-C(34)-C(33)	111.1(2)
C(35)-C(34)-C(60)	110.8(3)
C(33)-C(34)-C(60)	111.0(2)
C(34)-C(35)-C(36)	113.9(2)
O(8)-C(36)-N(2)	100.33(19)
O(8)-C(36)-C(37)	112.0(2)
N(2)-C(36)-C(37)	108.7(2)
O(8)-C(36)-C(35)	106.8(2)
N(2)-C(36)-C(35)	115.6(2)
C(37)-C(36)-C(35)	112.8(2)
C(36)-C(37)-C(38)	109.8(2)
C(39)-C(38)-C(37)	112.9(2)
C(59)-C(39)-C(38)	107.5(2)
C(59)-C(39)-C(40)	107.6(2)

C(38)-C(39)-C(40)	109.1(2)
C(59)-C(39)-C(52)	111.6(2)
C(38)-C(39)-C(52)	112.7(2)
C(40)-C(39)-C(52)	108.18(19)
N(2)-C(40)-O(14)	112.17(19)
N(2)-C(40)-C(41)	114.3(2)
O(14)-C(40)-C(41)	101.49(19)
N(2)-C(40)-C(39)	110.6(2)
O(14)-C(40)-C(39)	111.21(19)
C(41)-C(40)-C(39)	106.67(19)
O(9)-C(41)-C(40)	127.3(2)
O(9)-C(41)-C(42)	128.3(2)
C(40)-C(41)-C(42)	104.32(19)
C(41)-C(42)-C(58)	97.85(19)
C(41)-C(42)-C(43)	113.24(19)
C(58)-C(42)-C(43)	113.4(2)
C(41)-C(42)-C(51)	105.84(19)
C(58)-C(42)-C(51)	111.58(19)
C(43)-C(42)-C(51)	113.69(19)
C(42)-C(43)-C(48)	111.7(2)
C(42)-C(43)-C(44)	111.95(19)
C(48)-C(43)-C(44)	111.0(2)
C(45)-C(44)-C(43)	113.8(2)
C(46)-C(45)-C(57)	121.7(2)
C(46)-C(45)-C(44)	122.4(2)
C(57)-C(45)-C(44)	115.9(2)
C(45)-C(46)-C(47)	121.8(2)
O(10)-C(47)-C(46)	121.8(2)
O(10)-C(47)-C(48)	120.5(2)
C(46)-C(47)-C(48)	117.8(2)
C(47)-C(48)-C(43)	110.3(2)
C(47)-C(48)-C(49)	110.5(2)
C(43)-C(48)-C(49)	109.99(19)
C(50)-C(49)-C(56)	111.6(2)
C(50)-C(49)-C(48)	107.7(2)
C(56)-C(49)-C(48)	113.2(2)
O(11)-C(50)-C(49)	121.5(2)
O(11)-C(50)-C(51)	122.9(2)
C(49)-C(50)-C(51)	115.6(2)
C(50)-C(51)-C(52)	117.1(2)

C(50)-C(51)-C(42)	109.19(19)
C(52)-C(51)-C(42)	114.0(2)
C(55)-C(52)-C(53)	98.2(2)
C(55)-C(52)-C(51)	109.5(2)
C(53)-C(52)-C(51)	110.2(2)
C(55)-C(52)-C(39)	112.6(2)
C(53)-C(52)-C(39)	112.7(2)
C(51)-C(52)-C(39)	112.7(2)
C(54)-C(53)-C(52)	105.0(2)
O(12)-C(54)-O(13)	121.4(3)
O(12)-C(54)-C(53)	128.9(3)
O(13)-C(54)-C(53)	109.6(2)
O(13)-C(55)-C(52)	107.0(2)
O(14)-C(58)-C(42)	106.69(19)
Cl(2)-C(61)-Cl(1)	111.3(2)

Symmetry transformations used to generate equivalent atoms:

Table S28. Crystal data and experimental details for **16**

	Crystal data	
Empirical formula	$C_{30} H_{39} N O_7$	
Formula weight	525.62	
Crystal system	Monoclinic	
Space group	$P2_1$	
Unit cell dimensions	a = 8.2154(3) Å	α = 90°
	b = 12.6152(5) Å	β = 102.7039(11)°
	c = 12.7280(5) Å	γ = 90°
Volume	1286.82(9) Å^3	
Z	2	
F(000)	564	
Density (calculated)	1.357 Mg/m^3	
Wavelength	1.54178 Å	
Cell parameters reflections used	9583	
Theta range for Cell parameters	5.00 to 78.86°.	
Absorption coefficient	0.781 mm^{-1}	
Temperature	100(2) K	
Crystal size	0.200 x 0.200 x 0.150 mm^3	
	Data collection	
Diffractometer	Bruker AXS D8 VENTURE, PhotonIII_C28	
Absorption correction	Semi-empirical from equivalents	
Max. and min. transmission	1.0000 and 0.8989	
No. of measured reflections	30452	
No. of independent reflections	5265 [R(int) = 0.0355]	
No. of observed [I>2_igma(I)]	5234	
Completeness to theta = 67.679°	99.9 %	
Theta range for data collection	3.560 to 78.854°.	
	Refinement	
Final R indices [I>2sigma(I)]	R1 = 0.0484, wR2 = 0.1522	
R indices (all data)	R1 = 0.0494, wR2 = 0.1547	
Goodness-of-fit on F^2	1.506	
No. of reflections	5265	
No. of parameters	349	
No. of restraints	1	
Absolute structure parameter	-0.08(13)	
Largest diff. peak and hole	1.240 and -0.342 e.Å^{-3}	

Table S29. Bond lengths [Å] and angles [°] for **16**

O(1)-C(6)	1.431(3)
O(1)-C(2)	1.450(3)
O(2)-C(28)	1.442(3)
O(2)-C(10)	1.467(3)
O(3)-C(11)	1.408(3)
O(4)-C(17)	1.225(3)
O(5)-C(20)	1.218(3)
O(6)-C(24)	1.201(4)
O(7)-C(24)	1.361(3)
O(7)-C(25)	1.447(3)
N(1)-C(10)	1.425(3)
N(1)-C(1)	1.440(3)
N(1)-C(6)	1.471(3)
C(1)-C(2)	1.540(3)
C(2)-C(3)	1.509(4)
C(3)-C(4)	1.532(4)
C(4)-C(30)	1.523(4)
C(4)-C(5)	1.539(3)
C(5)-C(6)	1.544(4)
C(6)-C(7)	1.512(3)
C(7)-C(8)	1.536(3)
C(8)-C(9)	1.545(3)
C(9)-C(29)	1.538(3)
C(9)-C(10)	1.557(3)
C(9)-C(22)	1.592(3)
C(10)-C(11)	1.541(3)
C(11)-C(12)	1.524(3)
C(12)-C(28)	1.533(3)
C(12)-C(13)	1.556(3)
C(12)-C(21)	1.572(3)
C(13)-C(14)	1.531(3)
C(13)-C(18)	1.540(3)
C(14)-C(15)	1.510(3)
C(15)-C(16)	1.356(3)
C(15)-C(27)	1.499(4)
C(16)-C(17)	1.460(4)
C(17)-C(18)	1.521(3)

C(18)-C(19)	1.555(3)
C(19)-C(20)	1.516(3)
C(19)-C(26)	1.535(3)
C(20)-C(21)	1.517(3)
C(21)-C(22)	1.558(3)
C(22)-C(23)	1.548(3)
C(22)-C(25)	1.552(3)
C(23)-C(24)	1.503(3)
C(6)-O(1)-C(2)	102.96(19)
C(28)-O(2)-C(10)	109.34(17)
C(24)-O(7)-C(25)	110.04(18)
C(10)-N(1)-C(1)	126.7(2)
C(10)-N(1)-C(6)	121.5(2)
C(1)-N(1)-C(6)	109.66(19)
N(1)-C(1)-C(2)	101.8(2)
O(1)-C(2)-C(3)	108.0(2)
O(1)-C(2)-C(1)	103.2(2)
C(3)-C(2)-C(1)	111.8(2)
C(2)-C(3)-C(4)	112.2(2)
C(30)-C(4)-C(3)	111.4(2)
C(30)-C(4)-C(5)	112.4(2)
C(3)-C(4)-C(5)	109.9(2)
C(4)-C(5)-C(6)	111.3(2)
O(1)-C(6)-N(1)	101.4(2)
O(1)-C(6)-C(7)	109.8(2)
N(1)-C(6)-C(7)	109.6(2)
O(1)-C(6)-C(5)	107.6(2)
N(1)-C(6)-C(5)	113.2(2)
C(7)-C(6)-C(5)	114.4(2)
C(6)-C(7)-C(8)	110.1(2)
C(7)-C(8)-C(9)	112.4(2)
C(29)-C(9)-C(8)	108.64(19)
C(29)-C(9)-C(10)	111.22(19)
C(8)-C(9)-C(10)	106.05(18)
C(29)-C(9)-C(22)	110.01(18)
C(8)-C(9)-C(22)	111.21(19)
C(10)-C(9)-C(22)	109.66(17)
N(1)-C(10)-O(2)	111.68(19)
N(1)-C(10)-C(11)	112.09(19)

O(2)-C(10)-C(11)	101.13(17)
N(1)-C(10)-C(9)	109.51(19)
O(2)-C(10)-C(9)	107.20(17)
C(11)-C(10)-C(9)	114.90(18)
O(3)-C(11)-C(12)	113.05(18)
O(3)-C(11)-C(10)	116.4(2)
C(12)-C(11)-C(10)	100.52(17)
C(11)-C(12)-C(28)	98.18(17)
C(11)-C(12)-C(13)	112.60(18)
C(28)-C(12)-C(13)	113.60(18)
C(11)-C(12)-C(21)	106.39(17)
C(28)-C(12)-C(21)	113.10(17)
C(13)-C(12)-C(21)	112.00(17)
C(14)-C(13)-C(18)	108.72(18)
C(14)-C(13)-C(12)	114.66(19)
C(18)-C(13)-C(12)	111.48(19)
C(15)-C(14)-C(13)	111.0(2)
C(16)-C(15)-C(27)	122.1(2)
C(16)-C(15)-C(14)	121.0(2)
C(27)-C(15)-C(14)	116.8(2)
C(15)-C(16)-C(17)	122.5(2)
O(4)-C(17)-C(16)	121.5(2)
O(4)-C(17)-C(18)	121.1(2)
C(16)-C(17)-C(18)	117.3(2)
C(17)-C(18)-C(13)	110.94(19)
C(17)-C(18)-C(19)	109.85(19)
C(13)-C(18)-C(19)	110.99(18)
C(20)-C(19)-C(26)	109.7(2)
C(20)-C(19)-C(18)	109.56(19)
C(26)-C(19)-C(18)	112.2(2)
O(5)-C(20)-C(19)	121.2(2)
O(5)-C(20)-C(21)	124.8(2)
C(19)-C(20)-C(21)	113.9(2)
C(20)-C(21)-C(22)	119.3(2)
C(20)-C(21)-C(12)	106.01(17)
C(22)-C(21)-C(12)	115.48(17)
C(23)-C(22)-C(25)	99.15(19)
C(23)-C(22)-C(21)	114.32(19)
C(25)-C(22)-C(21)	108.42(18)
C(23)-C(22)-C(9)	116.2(2)

C(25)-C(22)-C(9)	109.06(19)
C(21)-C(22)-C(9)	108.94(18)
C(24)-C(23)-C(22)	105.5(2)
O(6)-C(24)-O(7)	121.5(2)
O(6)-C(24)-C(23)	129.0(3)
O(7)-C(24)-C(23)	109.5(2)
O(7)-C(25)-C(22)	106.86(19)
O(2)-C(28)-C(12)	105.73(18)

Symmetry transformations used to generate equivalent atoms:

Table S30. Crystal data and experimental details for kuroshine E

	Crystal data	
Empirical formula	$C_{30} H_{37} N O_7$	
Formula weight	523.60	
Crystal system	Orthorhombic	
Space group	$P2_12_12_1$	
Unit cell dimensions	a = 7.7384(2) Å	α = 90°.
	b = 13.8193(4) Å	β = 90°.
	c = 23.7794(6) Å	γ = 90°.
Volume	2542.95(12) Å^3	
Z	4	
F(000)	1120	
Density (calculated)	1.368 Mg/m^3	
Wavelength	1.54178 Å	
Cell parameters reflections used	9762	
Theta range for Cell parameters	3.70 to 78.17°.	
Absorption coefficient	0.790 mm^{-1}	
Temperature	100(2) K	
Crystal size	0.300 x 0.250 x 0.200 mm^3	
	Data collection	
Diffractometer	Bruker AXS D8 VENTURE, PhotonIII_C28	
Absorption correction	Semi-empirical from equivalents	
Max. and min. transmission	1.0000 and 0.8960	
No. of measured reflections	34805	
No. of independent reflections	5332 [R(int) = 0.0324]	
No. of observed [I>2_igma(I)]	5312	
Completeness to theta = 67.679°	100.0 %	
Theta range for data collection	3.699 to 78.277°.	
	Refinement	
Final R indices [I>2sigma(I)]	R1 = 0.0310, wR2 = 0.0894	
R indices (all data)	R1 = 0.0311, wR2 = 0.0896	
Goodness-of-fit on F^2	1.061	
No. of reflections	5332	
No. of parameters	347	
No. of restraints	0	
Absolute structure parameter	-0.03(15)	
Largest diff. peak and hole	0.290 and -0.302 e.Å^{-3}	

Table S31. Bond lengths [Å] and angles [°] for kuroshine E

O(1)-C(6)	1.426(2)
O(1)-C(2)	1.447(2)
O(2)-C(17)	1.227(2)
O(3)-C(20)	1.212(2)
O(4)-C(24)	1.206(2)
O(5)-C(24)	1.348(2)
O(5)-C(25)	1.4563(19)
O(6)-C(11)	1.209(2)
O(7)-C(28)	1.460(2)
O(7)-C(10)	1.4693(19)
N(1)-C(10)	1.406(2)
N(1)-C(6)	1.465(2)
N(1)-C(1)	1.468(2)
C(1)-C(2)	1.553(2)
C(2)-C(3)	1.523(3)
C(3)-C(4)	1.539(2)
C(4)-C(30)	1.530(3)
C(4)-C(5)	1.535(2)
C(5)-C(6)	1.537(3)
C(6)-C(7)	1.516(2)
C(7)-C(8)	1.532(2)
C(8)-C(9)	1.550(2)
C(9)-C(29)	1.549(2)
C(9)-C(10)	1.578(2)
C(9)-C(22)	1.582(2)
C(10)-C(11)	1.522(2)
C(11)-C(12)	1.526(2)
C(12)-C(28)	1.527(2)
C(12)-C(13)	1.531(2)
C(12)-C(21)	1.574(2)
C(13)-C(14)	1.531(2)
C(13)-C(18)	1.532(2)
C(14)-C(15)	1.503(2)
C(15)-C(16)	1.344(3)
C(15)-C(27)	1.502(3)
C(16)-C(17)	1.466(3)
C(17)-C(18)	1.529(2)

C(18)-C(19)	1.542(2)
C(19)-C(20)	1.520(2)
C(19)-C(26)	1.539(2)
C(20)-C(21)	1.542(2)
C(21)-C(22)	1.563(2)
C(22)-C(25)	1.548(2)
C(22)-C(23)	1.550(2)
C(23)-C(24)	1.509(2)
C(6)-O(1)-C(2)	102.76(12)
C(24)-O(5)-C(25)	109.57(13)
C(28)-O(7)-C(10)	110.91(12)
C(10)-N(1)-C(6)	119.96(14)
C(10)-N(1)-C(1)	123.26(13)
C(6)-N(1)-C(1)	108.18(13)
N(1)-C(1)-C(2)	101.24(13)
O(1)-C(2)-C(3)	108.05(15)
O(1)-C(2)-C(1)	103.22(13)
C(3)-C(2)-C(1)	113.25(14)
C(2)-C(3)-C(4)	110.49(14)
C(30)-C(4)-C(5)	111.62(15)
C(30)-C(4)-C(3)	111.60(15)
C(5)-C(4)-C(3)	109.69(15)
C(4)-C(5)-C(6)	113.25(14)
O(1)-C(6)-N(1)	100.60(13)
O(1)-C(6)-C(7)	110.68(14)
N(1)-C(6)-C(7)	108.22(14)
O(1)-C(6)-C(5)	108.12(13)
N(1)-C(6)-C(5)	115.60(14)
C(7)-C(6)-C(5)	112.92(15)
C(6)-C(7)-C(8)	109.03(14)
C(7)-C(8)-C(9)	112.80(14)
C(29)-C(9)-C(8)	107.84(14)
C(29)-C(9)-C(10)	107.46(13)
C(8)-C(9)-C(10)	109.82(13)
C(29)-C(9)-C(22)	111.34(13)
C(8)-C(9)-C(22)	112.14(13)
C(10)-C(9)-C(22)	108.13(12)
N(1)-C(10)-O(7)	112.77(13)
N(1)-C(10)-C(11)	114.58(14)

O(7)-C(10)-C(11)	101.13(12)
N(1)-C(10)-C(9)	110.80(13)
O(7)-C(10)-C(9)	112.26(12)
C(11)-C(10)-C(9)	104.74(13)
O(6)-C(11)-C(10)	127.59(14)
O(6)-C(11)-C(12)	128.11(15)
C(10)-C(11)-C(12)	104.28(13)
C(11)-C(12)-C(28)	99.47(12)
C(11)-C(12)-C(13)	111.51(13)
C(28)-C(12)-C(13)	117.98(13)
C(11)-C(12)-C(21)	106.20(12)
C(28)-C(12)-C(21)	108.78(13)
C(13)-C(12)-C(21)	111.71(12)
C(14)-C(13)-C(12)	112.92(13)
C(14)-C(13)-C(18)	109.53(14)
C(12)-C(13)-C(18)	111.85(13)
C(15)-C(14)-C(13)	111.51(14)
C(16)-C(15)-C(27)	123.16(16)
C(16)-C(15)-C(14)	120.82(16)
C(27)-C(15)-C(14)	116.01(16)
C(15)-C(16)-C(17)	122.19(16)
O(2)-C(17)-C(16)	121.12(17)
O(2)-C(17)-C(18)	120.60(17)
C(16)-C(17)-C(18)	118.25(15)
C(17)-C(18)-C(13)	111.58(14)
C(17)-C(18)-C(19)	111.67(14)
C(13)-C(18)-C(19)	112.42(13)
C(20)-C(19)-C(26)	107.93(14)
C(20)-C(19)-C(18)	111.17(13)
C(26)-C(19)-C(18)	113.89(15)
O(3)-C(20)-C(19)	119.94(15)
O(3)-C(20)-C(21)	123.07(16)
C(19)-C(20)-C(21)	116.81(14)
C(20)-C(21)-C(22)	115.42(13)
C(20)-C(21)-C(12)	108.70(12)
C(22)-C(21)-C(12)	114.99(12)
C(25)-C(22)-C(23)	99.59(12)
C(25)-C(22)-C(21)	107.54(12)
C(23)-C(22)-C(21)	117.09(13)
C(25)-C(22)-C(9)	110.69(13)

C(23)-C(22)-C(9)	110.86(13)
C(21)-C(22)-C(9)	110.45(12)
C(24)-C(23)-C(22)	103.84(13)
O(4)-C(24)-O(5)	120.89(16)
O(4)-C(24)-C(23)	128.56(16)
O(5)-C(24)-C(23)	110.55(14)
O(5)-C(25)-C(22)	106.74(13)
O(7)-C(28)-C(12)	105.58(12)

Symmetry transformations used to generate equivalent atoms:

Table S32. Crystal data and experimental details for 28-deoxyzoanthenamine

	Crystal data	
Empirical formula	$C_{30} H_{39} N O_5$	
Formula weight	493.62	
Crystal system	Monoclinic	
Space group	$P2_1$	
Unit cell dimensions	a = 10.3705(4) Å	α = 90°.
	b = 10.0946(3) Å	β = 111.0710(11)°.
	c = 13.1069(4) Å	γ = 90°.
Volume	1280.36(7) $Å^3$	
Z	2	
F(000)	532	
Density (calculated)	1.280 Mg/m^3	
Wavelength	1.54178 Å	
Cell parameters reflections used	9779	
Theta range for Cell parameters	3.61 to 78.29°.	
Absorption coefficient	0.690 mm^{-1}	
Temperature	100(2) K	
Crystal size	0.200 x 0.150 x 0.100 mm^3	
	Data collection	
Diffractometer	Bruker AXS D8 VENTURE, PhotonIII_C28	
Absorption correction	Semi-empirical from equivalents	
Max. and min. transmission	1.0000 and 0.9019	
No. of measured reflections	23449	
No. of independent reflections	5143 [R(int) = 0.0369]	
No. of observed [I>2_igma(I)]	5099	
Completeness to theta = 67.679°	98.9 %	
Theta range for data collection	4.569 to 78.527°.	
	Refinement	
Final R indices [I>2sigma(I)]	R1 = 0.0456, wR2 = 0.1180	
R indices (all data)	R1 = 0.0459, wR2 = 0.1182	
Goodness-of-fit on F^2	1.016	
No. of reflections	5143	
No. of parameters	330	
No. of restraints	1	
Absolute structure parameter	-0.10(9)	
Largest diff. peak and hole	1.094 and -0.306 e.$Å^{-3}$	

Table S33. Bond lengths [Å] and angles [°] for 28-deoxyzoanthenamine

O(1)-C(6)	1.433(3)
O(1)-C(2)	1.457(3)
O(2)-C(17)	1.224(4)
O(3)-C(20)	1.223(4)
O(4)-C(24)	1.201(4)
O(5)-C(24)	1.344(3)
O(5)-C(25)	1.450(3)
N(1)-C(10)	1.385(3)
N(1)-C(1)	1.462(3)
N(1)-C(6)	1.465(3)
C(1)-C(2)	1.544(4)
C(2)-C(3)	1.515(4)
C(3)-C(4)	1.533(4)
C(4)-C(30)	1.527(4)
C(4)-C(5)	1.540(4)
C(5)-C(6)	1.537(4)
C(6)-C(7)	1.509(4)
C(7)-C(8)	1.537(4)
C(8)-C(9)	1.549(3)
C(9)-C(10)	1.534(3)
C(9)-C(29)	1.556(3)
C(9)-C(22)	1.570(4)
C(10)-C(11)	1.352(4)
C(11)-C(12)	1.500(4)
C(12)-C(13)	1.560(4)
C(12)-C(21)	1.562(3)
C(12)-C(28)	1.593(4)
C(13)-C(14)	1.523(4)
C(13)-C(18)	1.539(4)
C(14)-C(15)	1.507(4)
C(15)-C(16)	1.337(4)
C(15)-C(27)	1.499(4)
C(16)-C(17)	1.460(4)
C(17)-C(18)	1.521(4)
C(18)-C(19)	1.539(4)
C(19)-C(20)	1.523(4)
C(19)-C(26)	1.533(5)

C(20)-C(21)	1.525(4)
C(21)-C(22)	1.541(4)
C(22)-C(23)	1.539(4)
C(22)-C(25)	1.559(4)
C(23)-C(24)	1.501(4)
C(6)-O(1)-C(2)	103.01(19)
C(24)-O(5)-C(25)	111.5(2)
C(10)-N(1)-C(1)	123.8(2)
C(10)-N(1)-C(6)	123.8(2)
C(1)-N(1)-C(6)	109.3(2)
N(1)-C(1)-C(2)	101.3(2)
O(1)-C(2)-C(3)	108.1(2)
O(1)-C(2)-C(1)	103.6(2)
C(3)-C(2)-C(1)	112.1(2)
C(2)-C(3)-C(4)	111.2(2)
C(30)-C(4)-C(3)	110.7(2)
C(30)-C(4)-C(5)	111.9(2)
C(3)-C(4)-C(5)	110.3(2)
C(6)-C(5)-C(4)	113.3(2)
O(1)-C(6)-N(1)	100.9(2)
O(1)-C(6)-C(7)	110.7(2)
N(1)-C(6)-C(7)	109.7(2)
O(1)-C(6)-C(5)	107.5(2)
N(1)-C(6)-C(5)	113.5(2)
C(7)-C(6)-C(5)	113.7(2)
C(6)-C(7)-C(8)	109.2(2)
C(7)-C(8)-C(9)	113.3(2)
C(10)-C(9)-C(8)	112.4(2)
C(10)-C(9)-C(29)	106.2(2)
C(8)-C(9)-C(29)	107.6(2)
C(10)-C(9)-C(22)	108.8(2)
C(8)-C(9)-C(22)	110.3(2)
C(29)-C(9)-C(22)	111.5(2)
C(11)-C(10)-N(1)	122.6(2)
C(11)-C(10)-C(9)	121.7(2)
N(1)-C(10)-C(9)	113.9(2)
C(10)-C(11)-C(12)	124.9(3)
C(11)-C(12)-C(13)	114.0(2)
C(11)-C(12)-C(21)	111.5(2)

C(13)-C(12)-C(21)	105.7(2)
C(11)-C(12)-C(28)	106.5(2)
C(13)-C(12)-C(28)	109.1(2)
C(21)-C(12)-C(28)	110.0(2)
C(14)-C(13)-C(18)	111.3(2)
C(14)-C(13)-C(12)	114.0(2)
C(18)-C(13)-C(12)	109.4(2)
C(15)-C(14)-C(13)	114.1(2)
C(16)-C(15)-C(27)	122.3(3)
C(16)-C(15)-C(14)	121.8(3)
C(27)-C(15)-C(14)	115.9(3)
C(15)-C(16)-C(17)	123.1(3)
O(2)-C(17)-C(16)	121.4(3)
O(2)-C(17)-C(18)	121.0(3)
C(16)-C(17)-C(18)	117.5(3)
C(17)-C(18)-C(19)	112.3(2)
C(17)-C(18)-C(13)	112.5(2)
C(19)-C(18)-C(13)	113.2(2)
C(20)-C(19)-C(26)	107.1(3)
C(20)-C(19)-C(18)	112.3(2)
C(26)-C(19)-C(18)	113.6(3)
O(3)-C(20)-C(19)	121.3(3)
O(3)-C(20)-C(21)	123.6(3)
C(19)-C(20)-C(21)	114.6(2)
C(20)-C(21)-C(22)	117.7(2)
C(20)-C(21)-C(12)	105.9(2)
C(22)-C(21)-C(12)	114.8(2)
C(23)-C(22)-C(21)	112.0(2)
C(23)-C(22)-C(25)	102.0(2)
C(21)-C(22)-C(25)	111.6(2)
C(23)-C(22)-C(9)	112.0(2)
C(21)-C(22)-C(9)	108.6(2)
C(25)-C(22)-C(9)	110.7(2)
C(24)-C(23)-C(22)	106.1(2)
O(4)-C(24)-O(5)	122.3(3)
O(4)-C(24)-C(23)	127.1(3)
O(5)-C(24)-C(23)	110.6(2)
O(5)-C(25)-C(22)	107.2(2)

Symmetry transformations used to generate equivalent atoms:

Figure S1. ^{1}H NMR spectrum of **1** (C_5D_5N, 600 MHz)

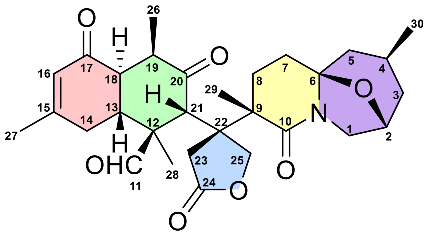

1

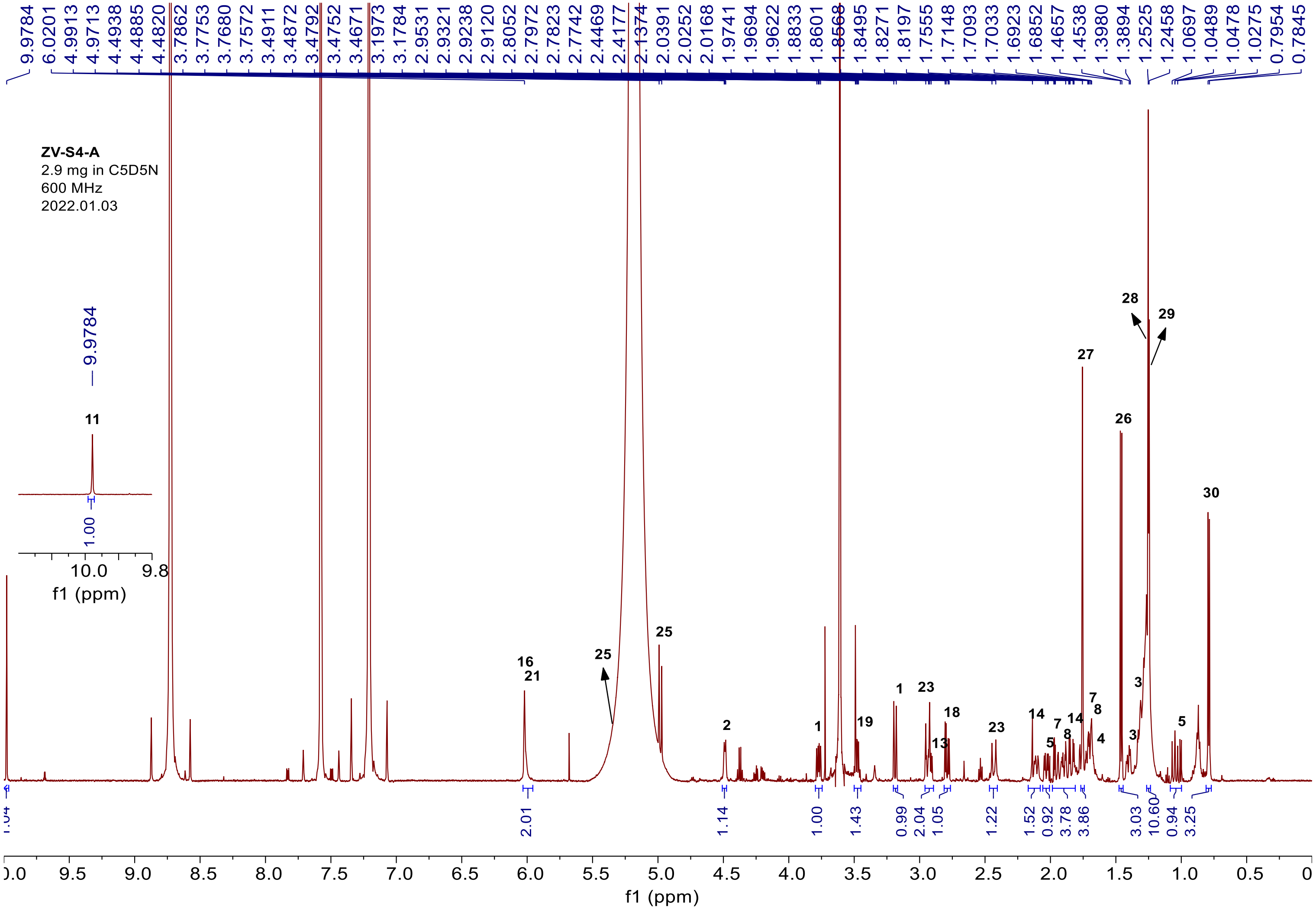

Figure S2. $^{13}C\{^1H\}$ NMR and DEPT spectra of **1** (C_5D_5N, 150 MHz)

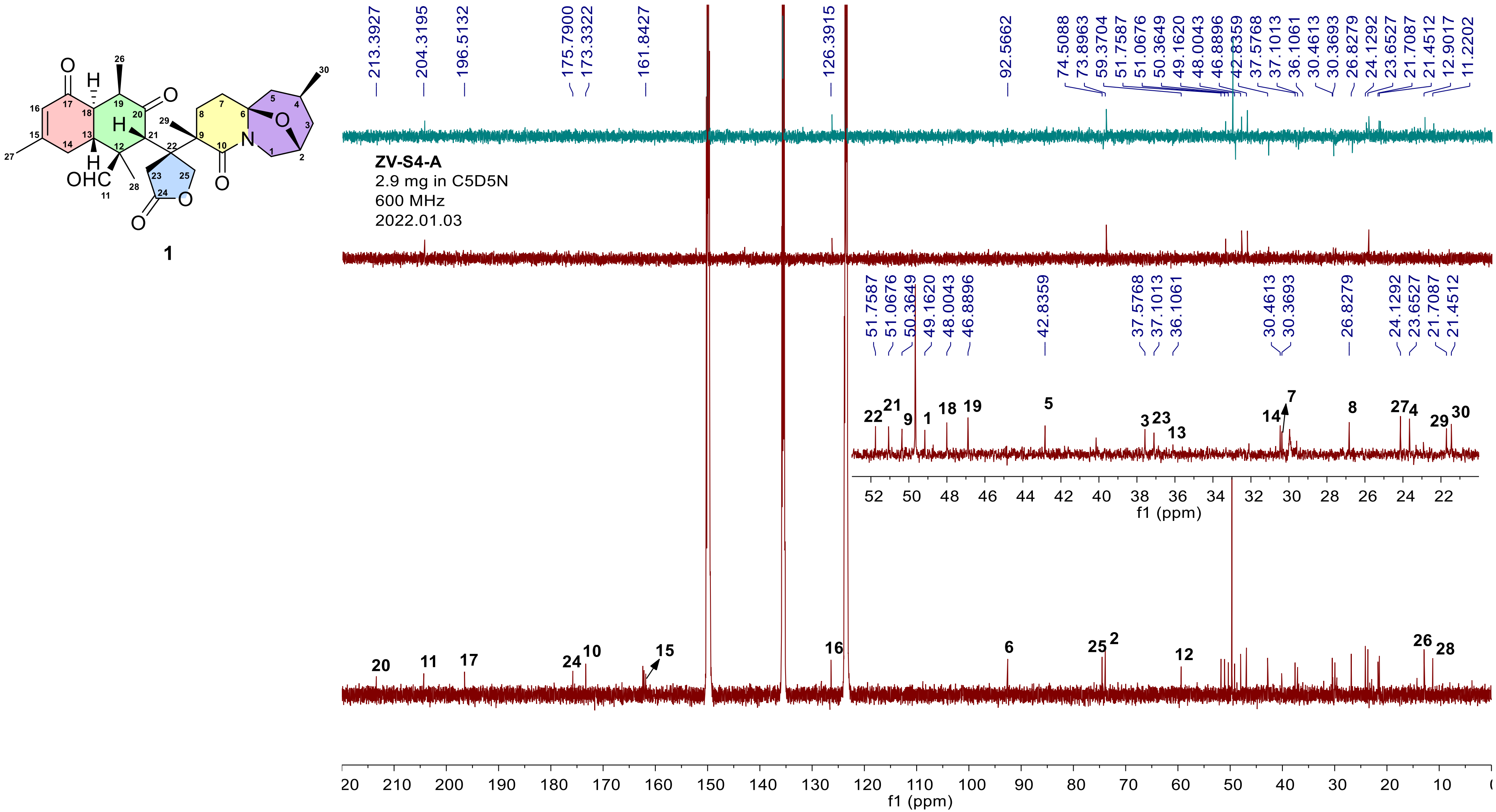

Figure S3. COSY spectrum of **1**

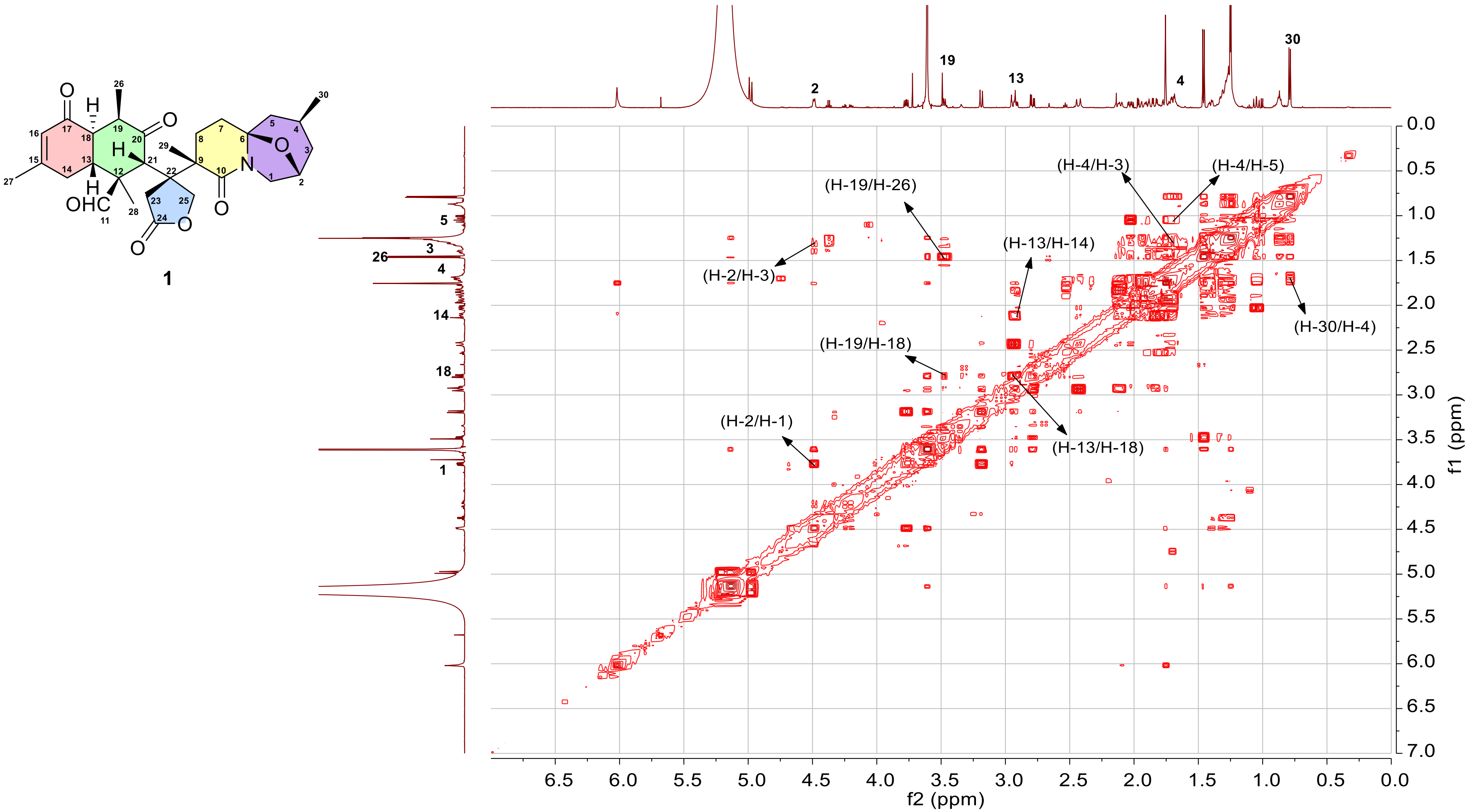

Figure S4. HSQC spectrum of **1**

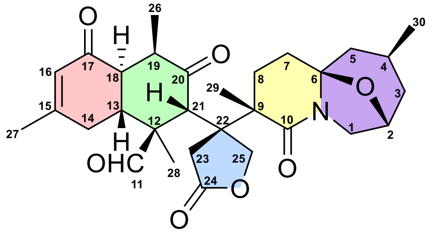

1

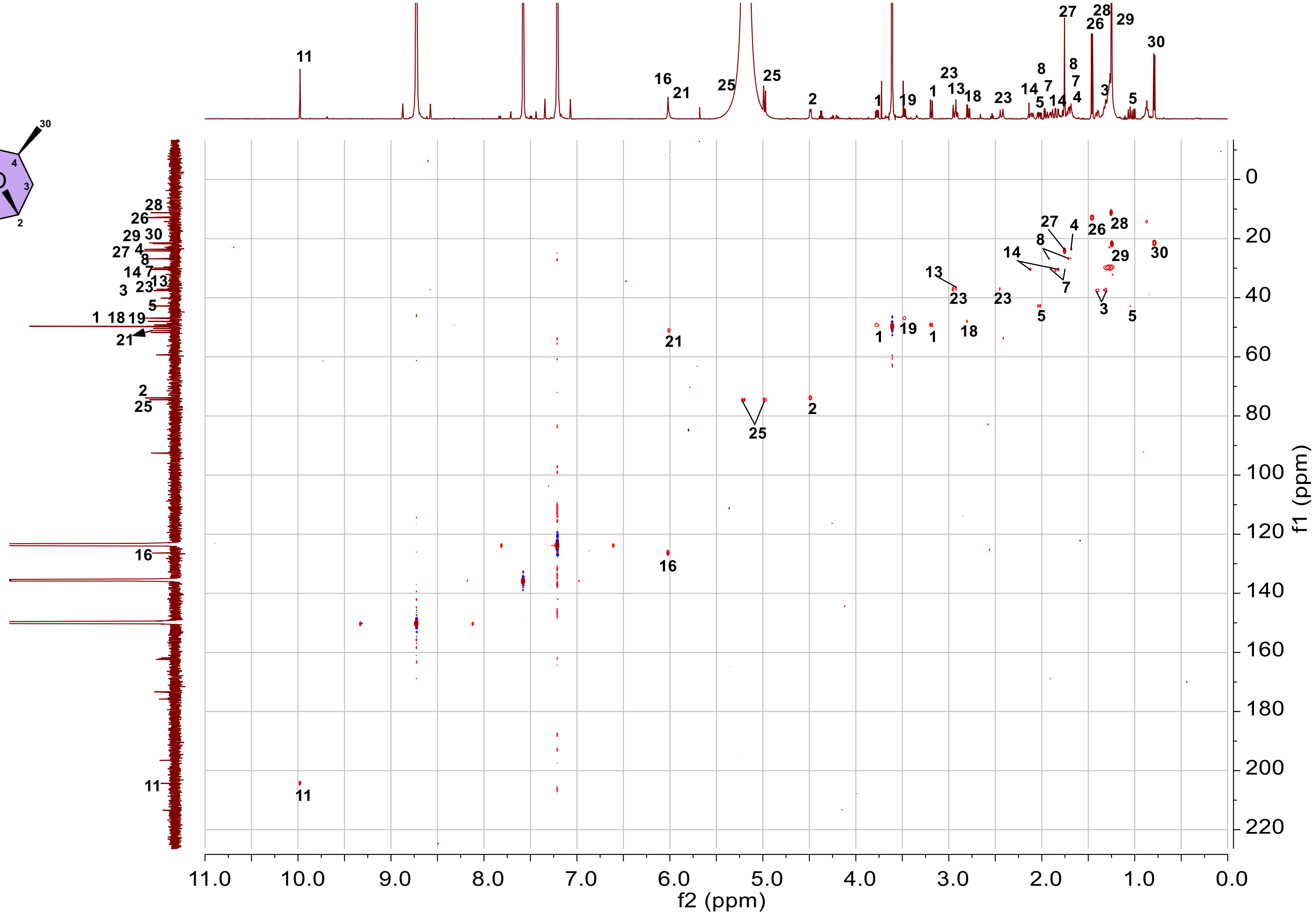

Figure S5. HMBC spectrum of **1**

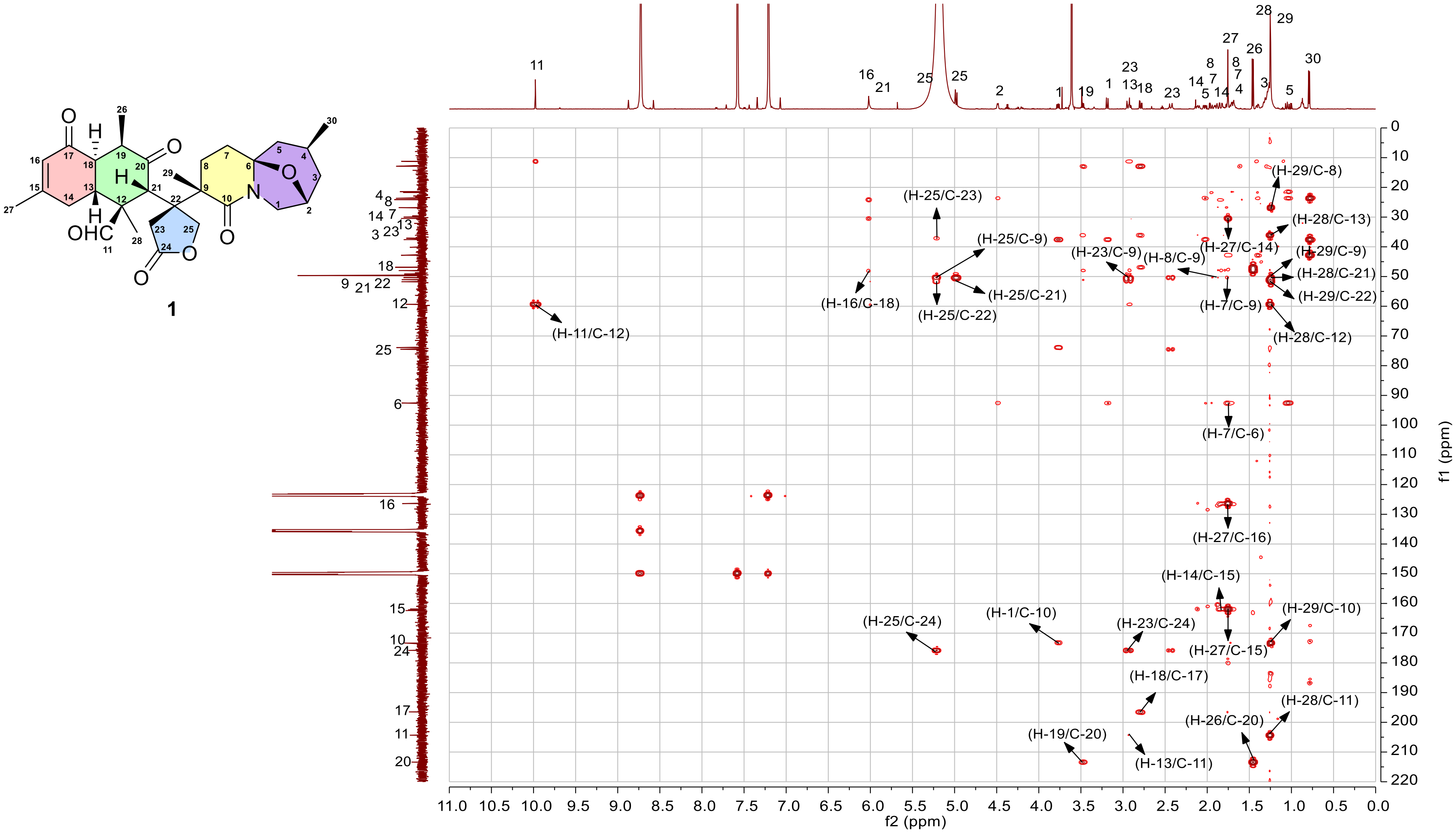

Figure S6. NOESY spectrum of **1**

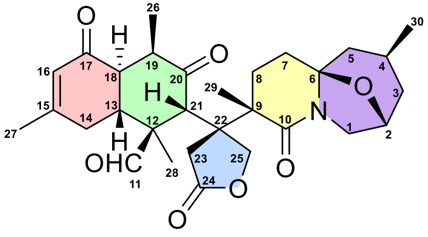

1

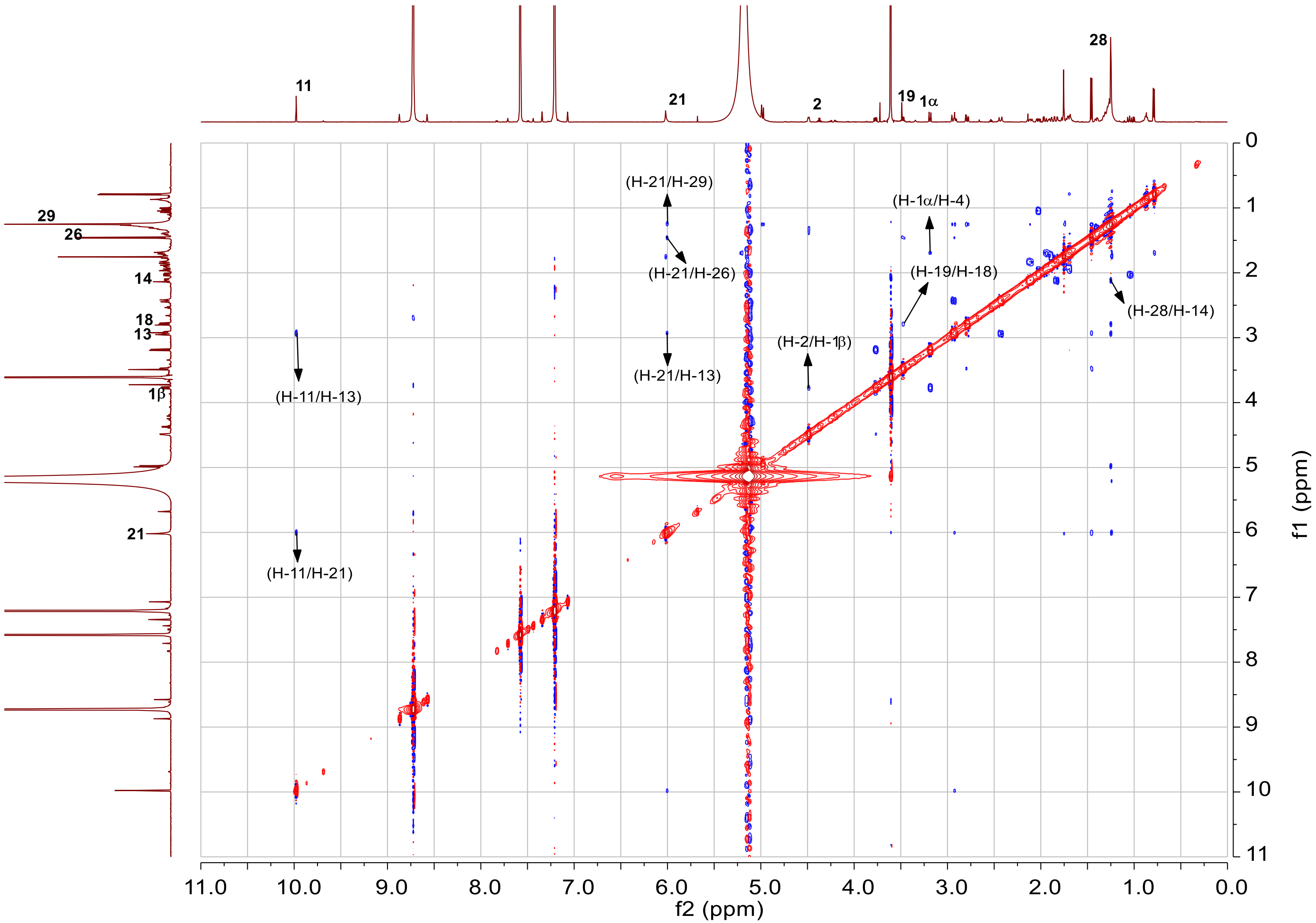

Figure S7. HRESIMS spectrum of **1**

Mass Spectrum SmartFormula Report

Analysis Info

Analysis Name	D:\QTOF\ZVS4GH1.d	2/24/2022 2:48:10 PM
Method	_tune_low_pos(50-1000)_20170502_Liao lab.m	Operator: YU HSIAO-CHING
Sample Name	ZV-S4-G	Instrument: BRUKER micrOTOF-Q
Comment	ESI Positive	

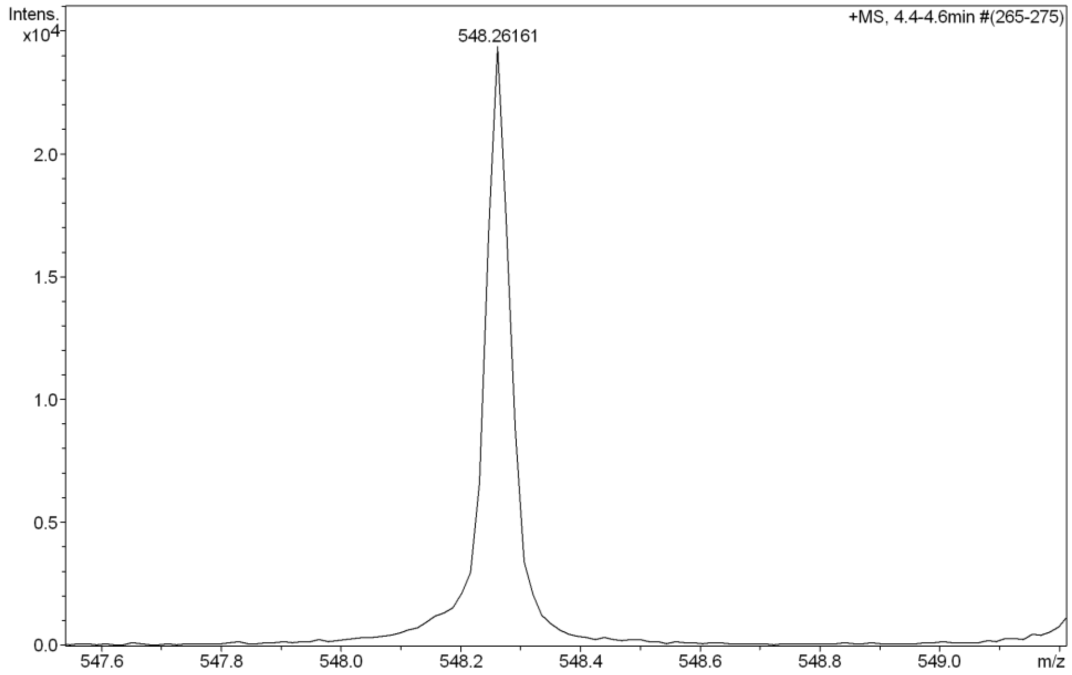

Meas. m/z	#	Formula	Score	m/z	err [mDa]	err [ppm]	mSigma	rdb	e⁻ Conf	N-Rule
548.26161	1	C 30 H 39 N Na O 7	100.00	548.26187	0.26	0.48	6.0	11.5	even	ok

Figure S8. UV spectrum of **1**

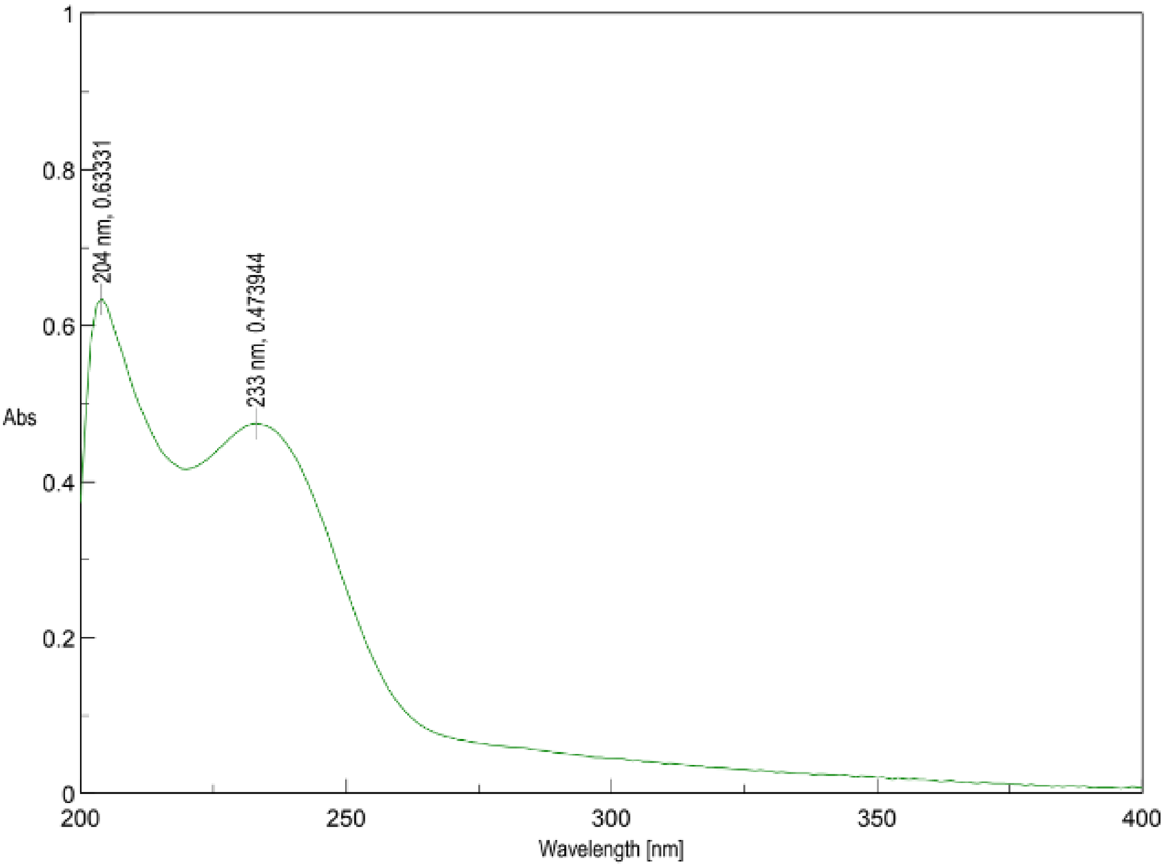

Figure S9. IR spectrum of **1**

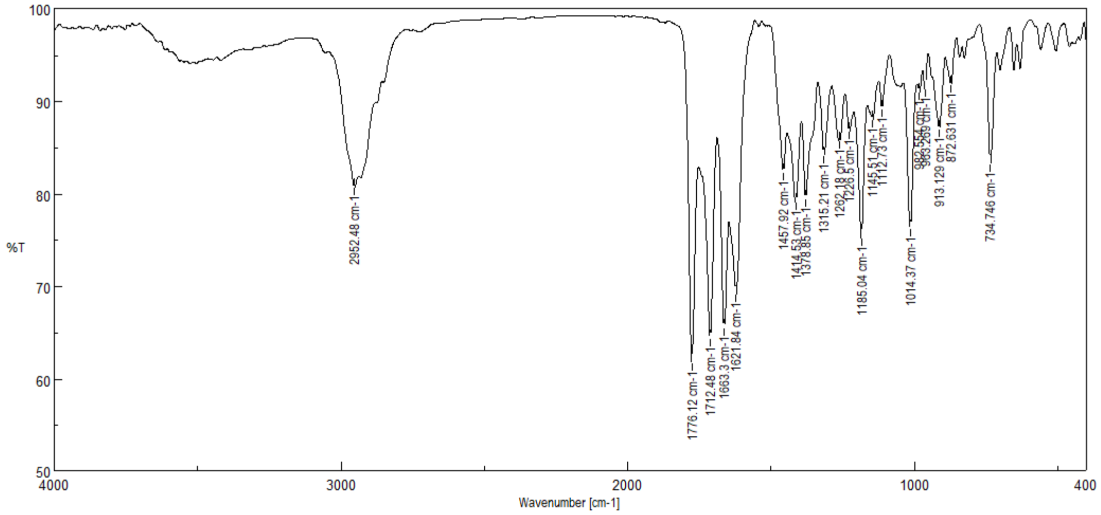

Figure S10. ^{1}H NMR spectrum of **2** (C_5D_5N, 600 MHz)

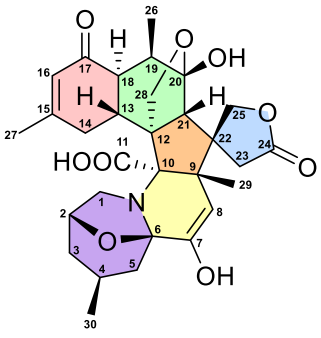

2

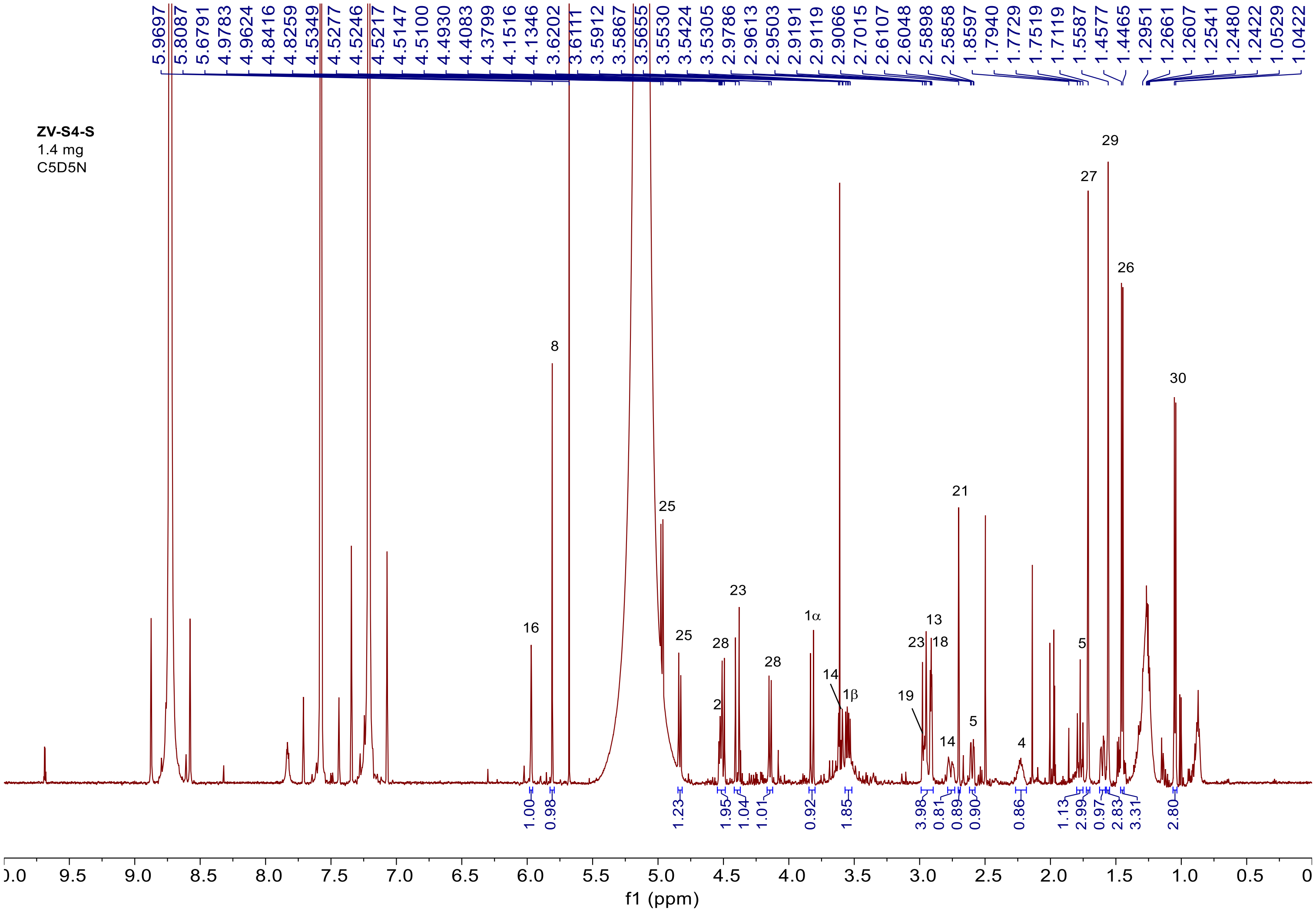

Figure S11. $^{13}C\{^{1}H\}$ NMR and DEPT spectra of **2** (C_5D_5N, 150 MHz)

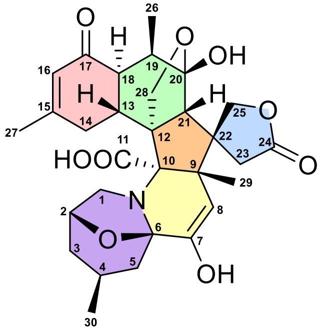

2

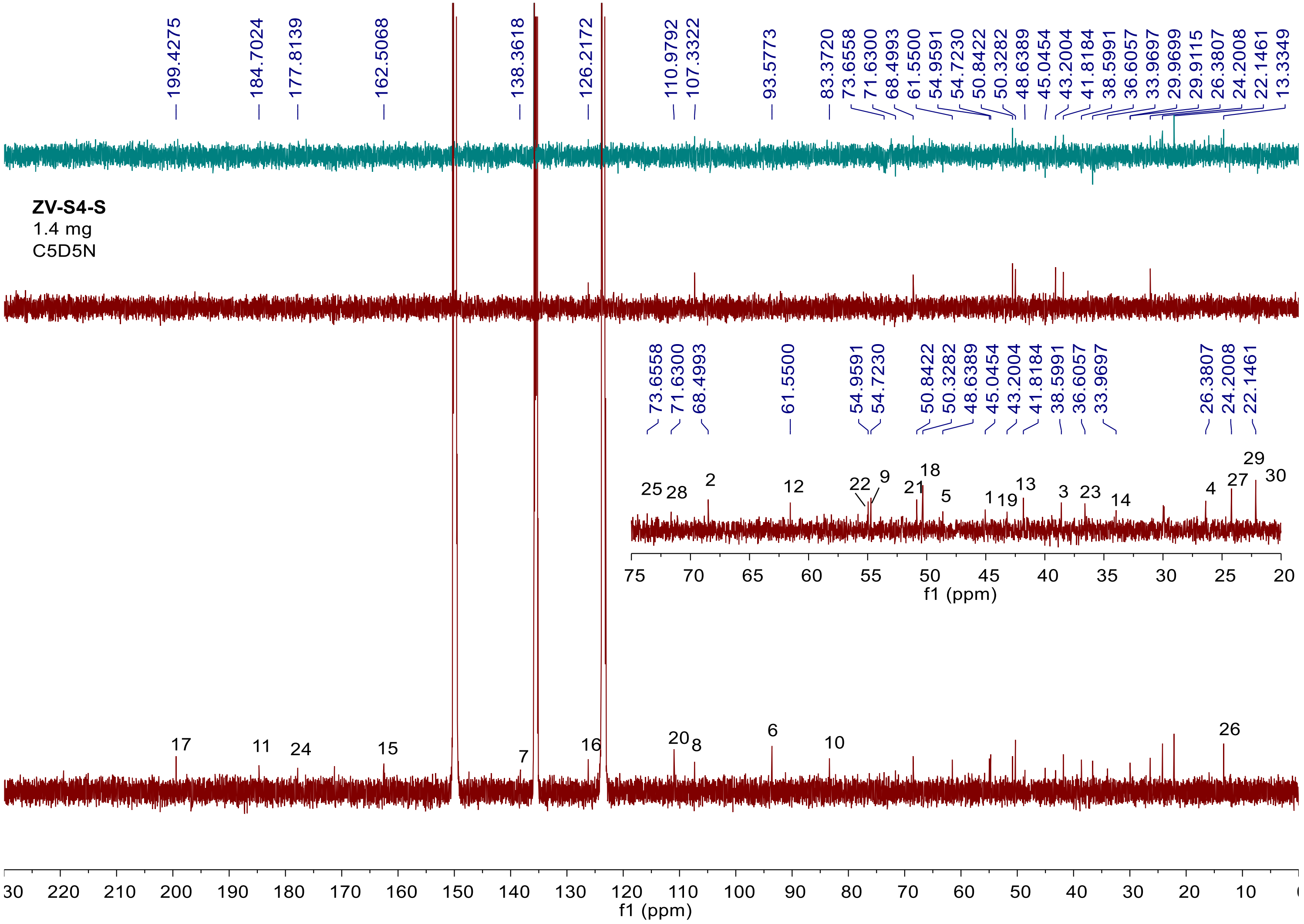

Figure S12. COSY spectrum of **2**

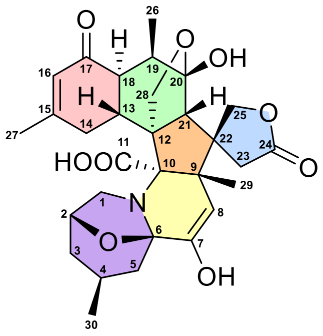

2

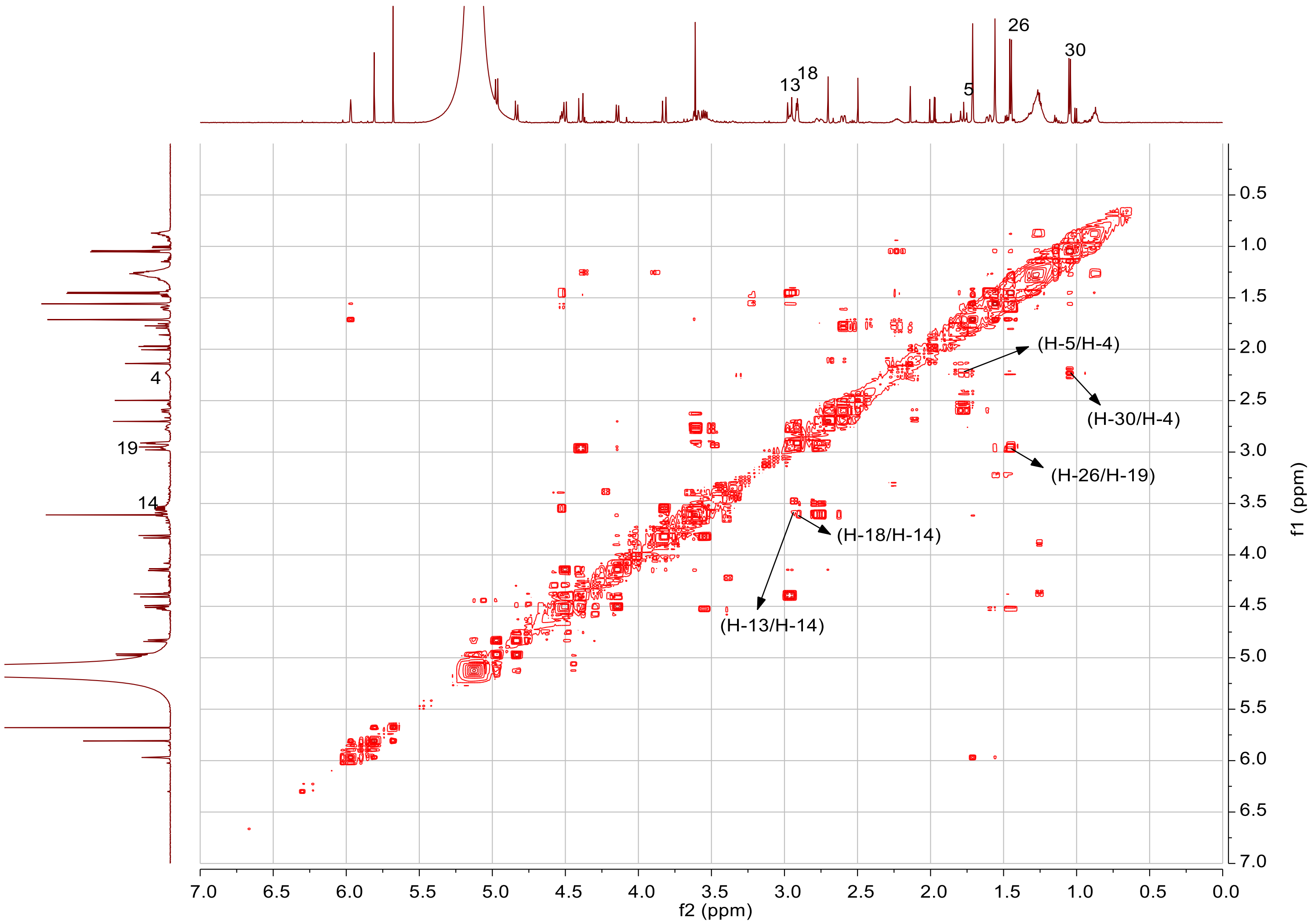

Figure S13. HSQC spectrum of **2**

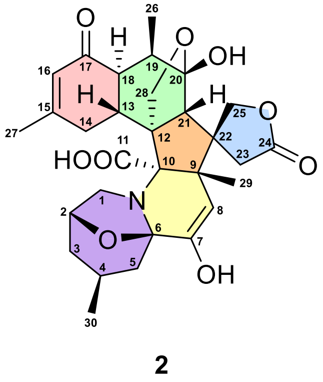

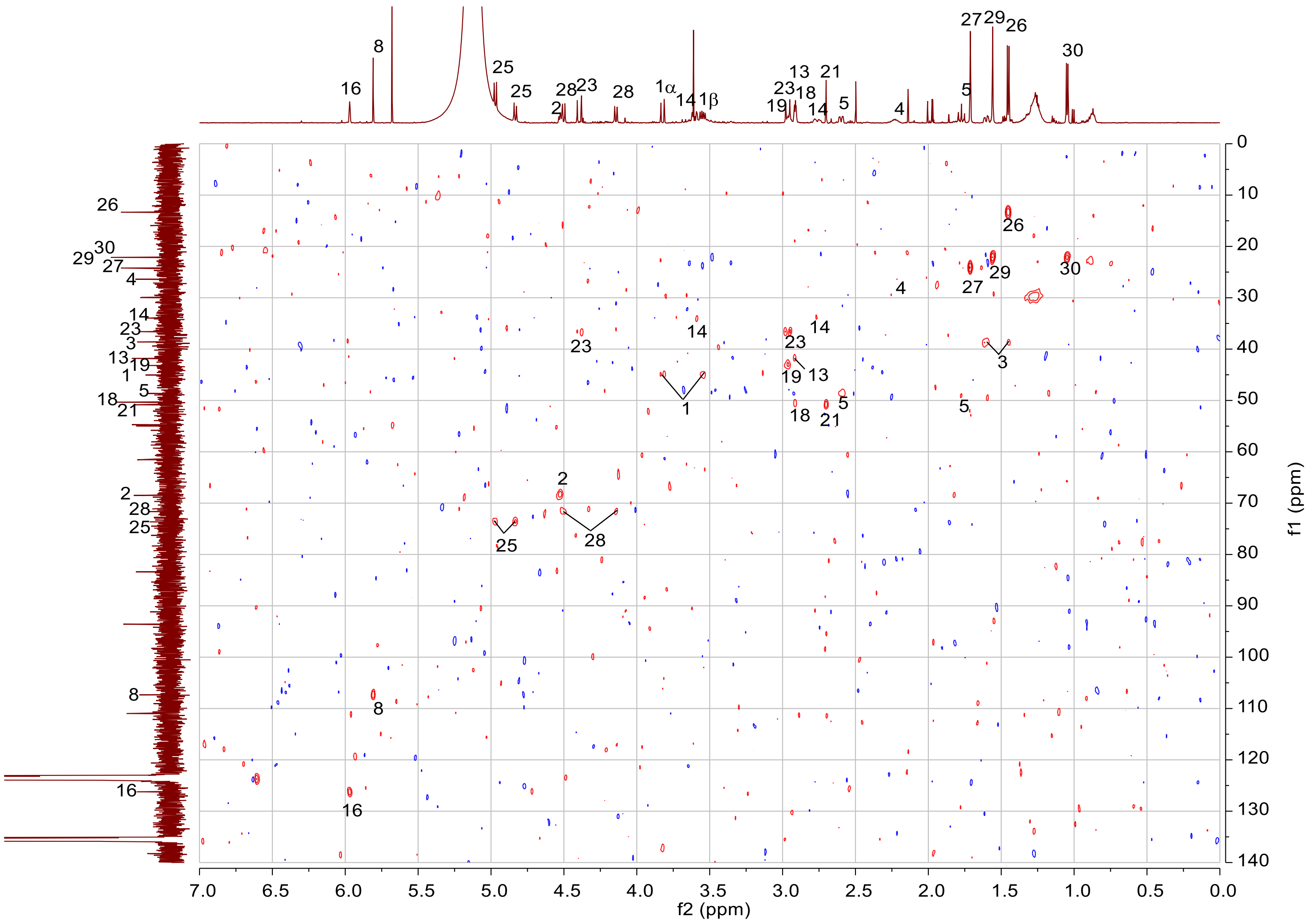

Figure S14. HMBC spectrum of **2**

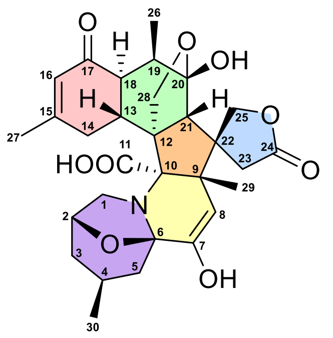

2

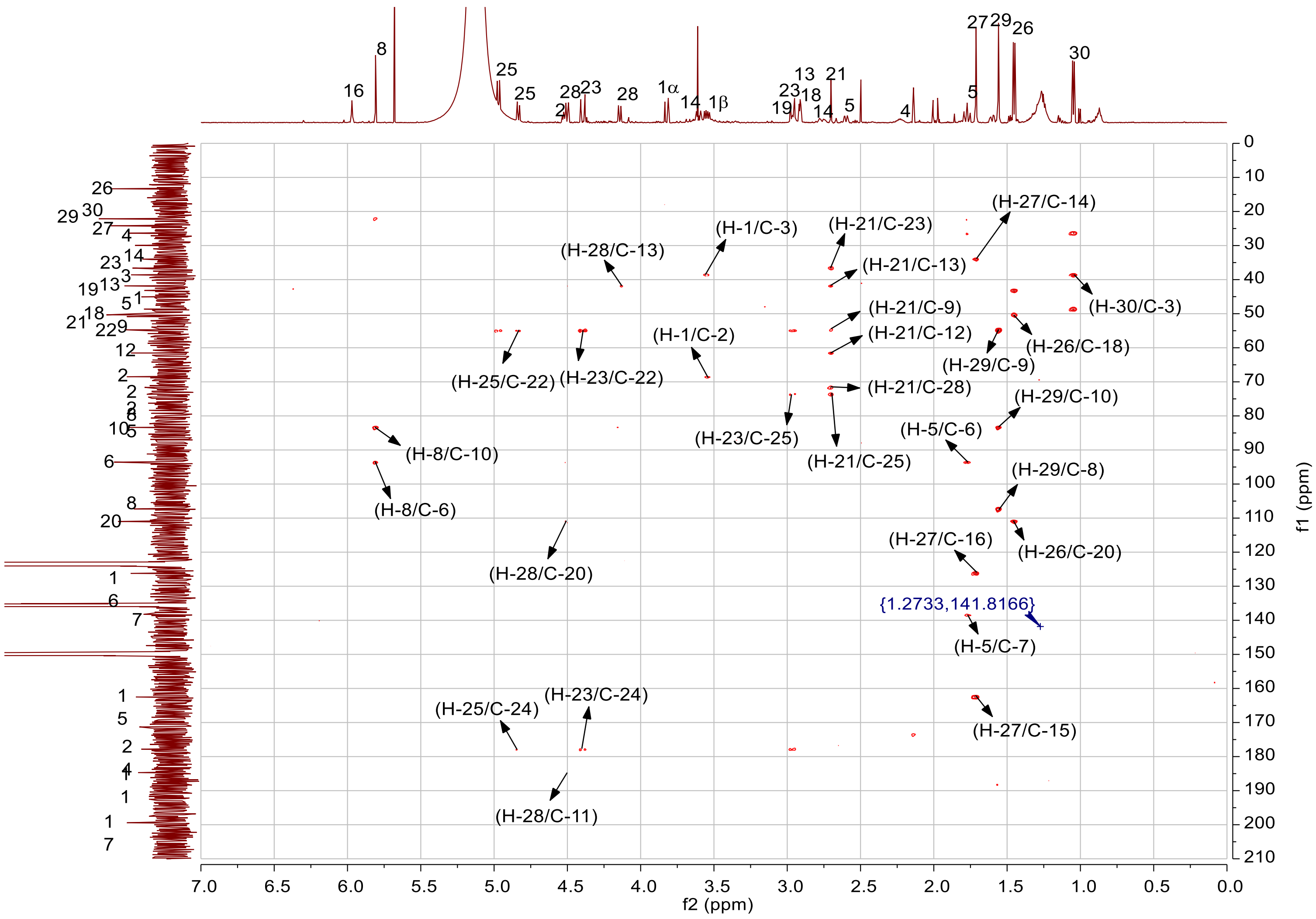

Figure S15. NOESY spectrum of **2**

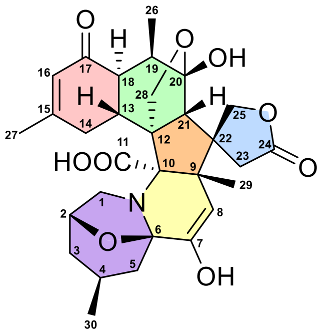

2

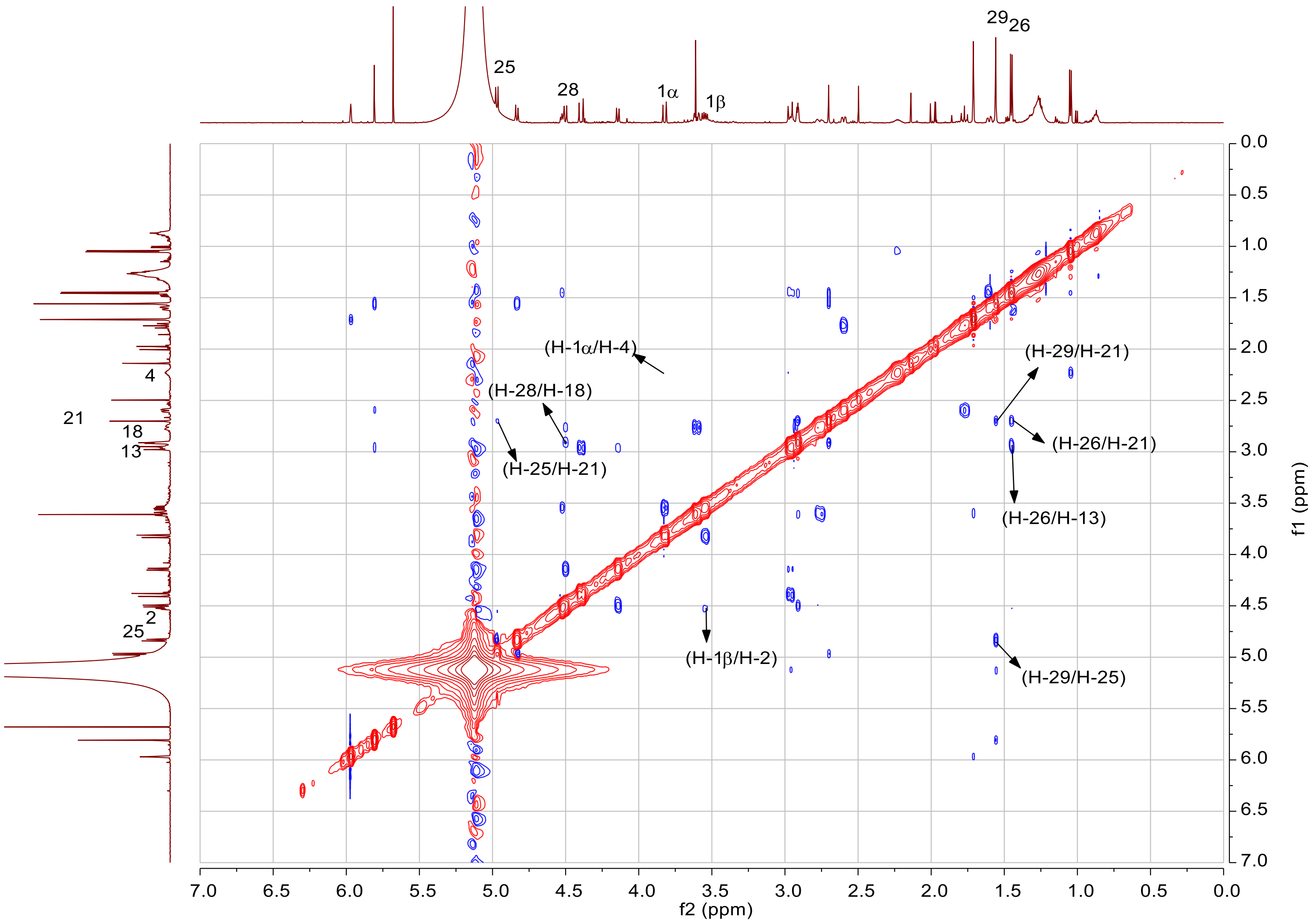

Figure S16. HRESIMS spectrum of **2**

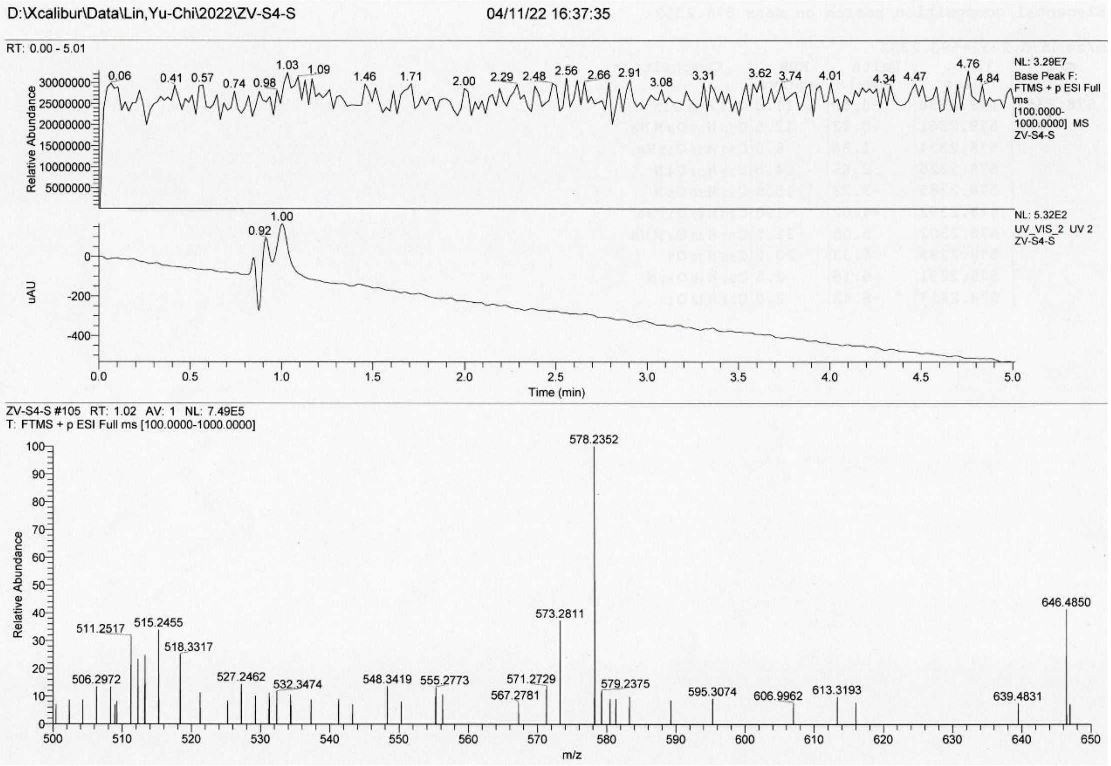

Elemental composition search on mass 578.2352

m/z= 573.2352-583.2352

m/z	Theo. Mass	Delta (mmu)	RDB equiv.	Composition
578.2352	578.2358	-0.55	11.0	$C_{29}H_{38}O_{12}$
	578.2361	-0.82	12.5	$C_{30}H_{37}O_{9}NNa$
	578.2334	1.86	8.0	$C_{27}H_{39}O_{12}Na$
	578.2326	2.65	24.5	$C_{39}H_{32}O_{4}N$
	578.2385	-3.23	15.5	$C_{32}H_{36}O_{9}N$
	578.2392	-4.02	-1.0	$C_{20}H_{43}O_{17}Na$
	578.2302	5.05	21.5	$C_{37}H_{33}O_{4}NNa$
	578.2299	5.33	20.0	$C_{36}H_{34}O_{7}$
	578.2291	6.16	2.5	$C_{21}H_{40}O_{17}N$
	578.2417	-6.42	2.0	$C_{22}H_{42}O_{17}$

Figure S17. UV spectrum of **2**

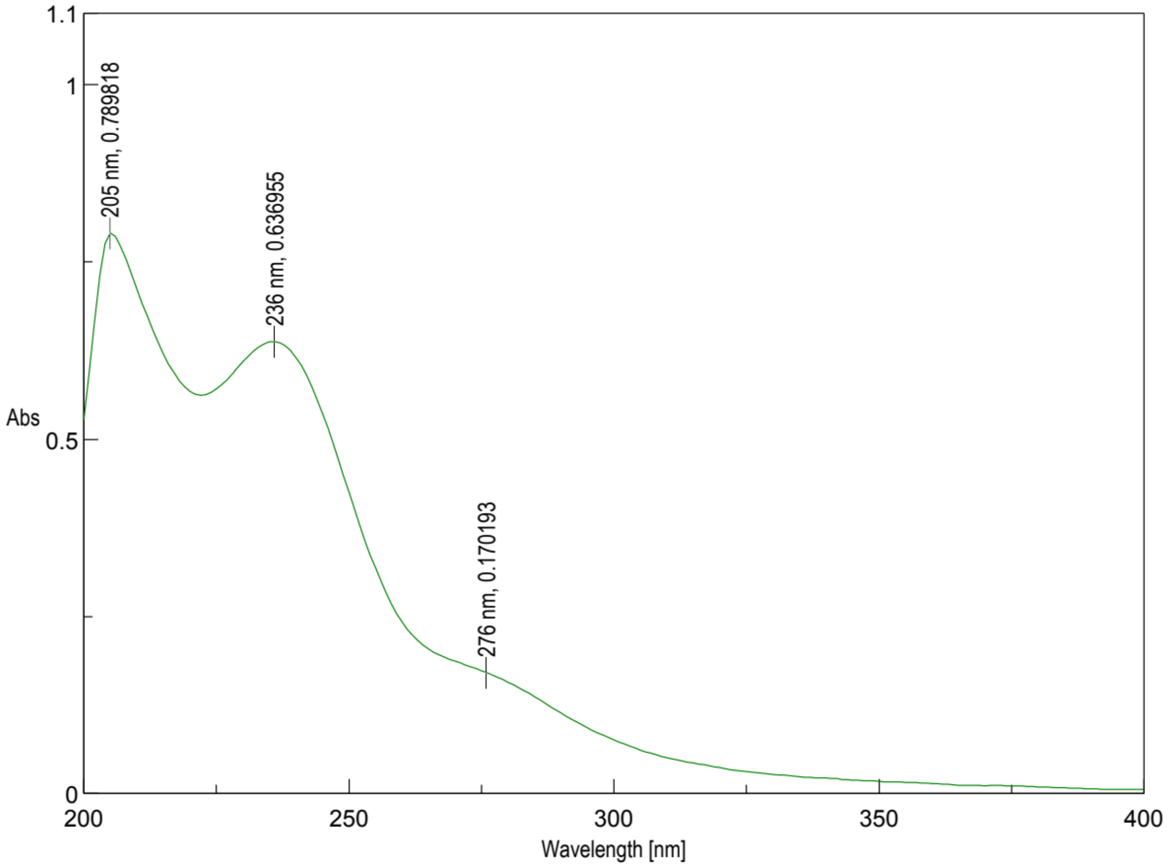

Figure S18. IR spectrum of **2**

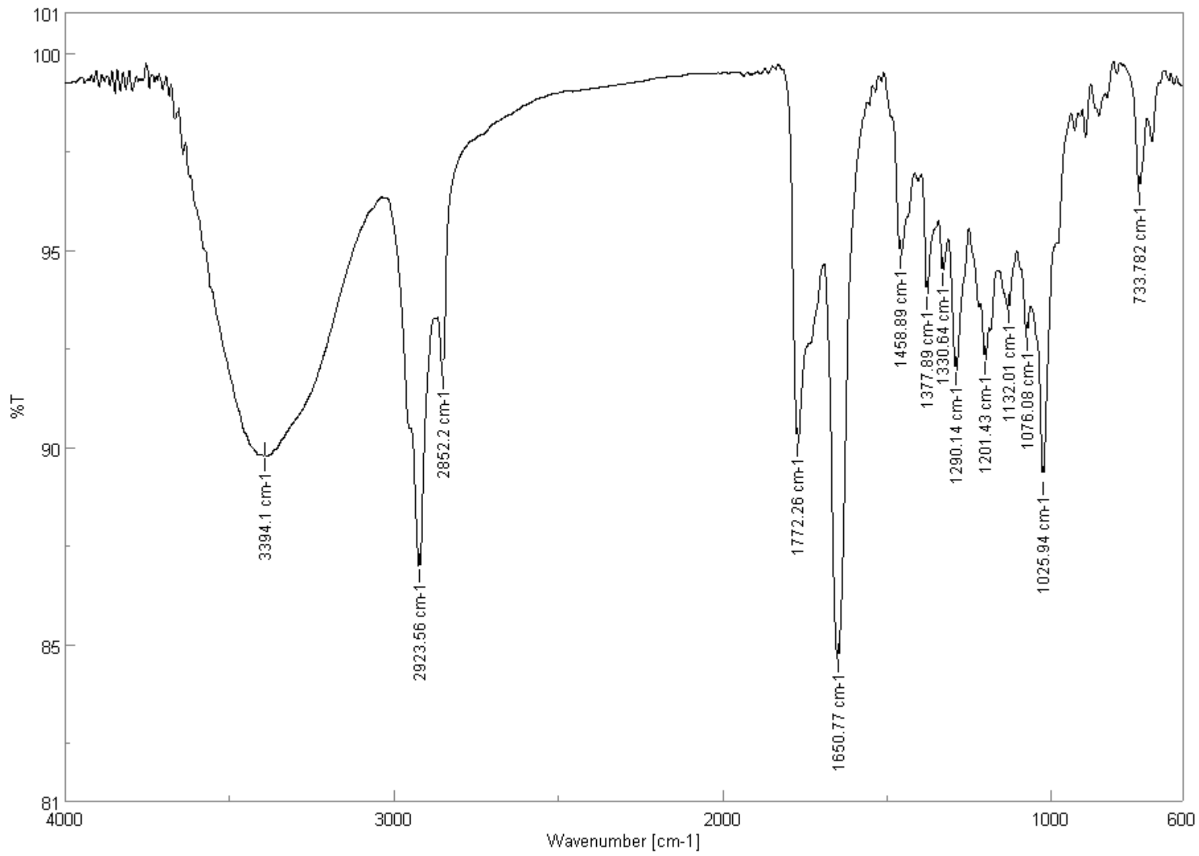

Figure S19. ^{1}H NMR spectrum of **3** (C_5D_5N, 600 MHz)

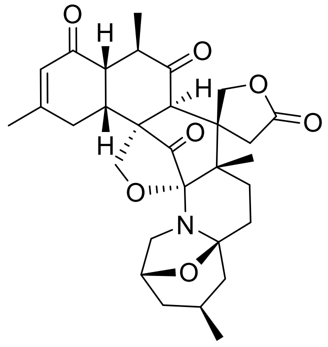

3

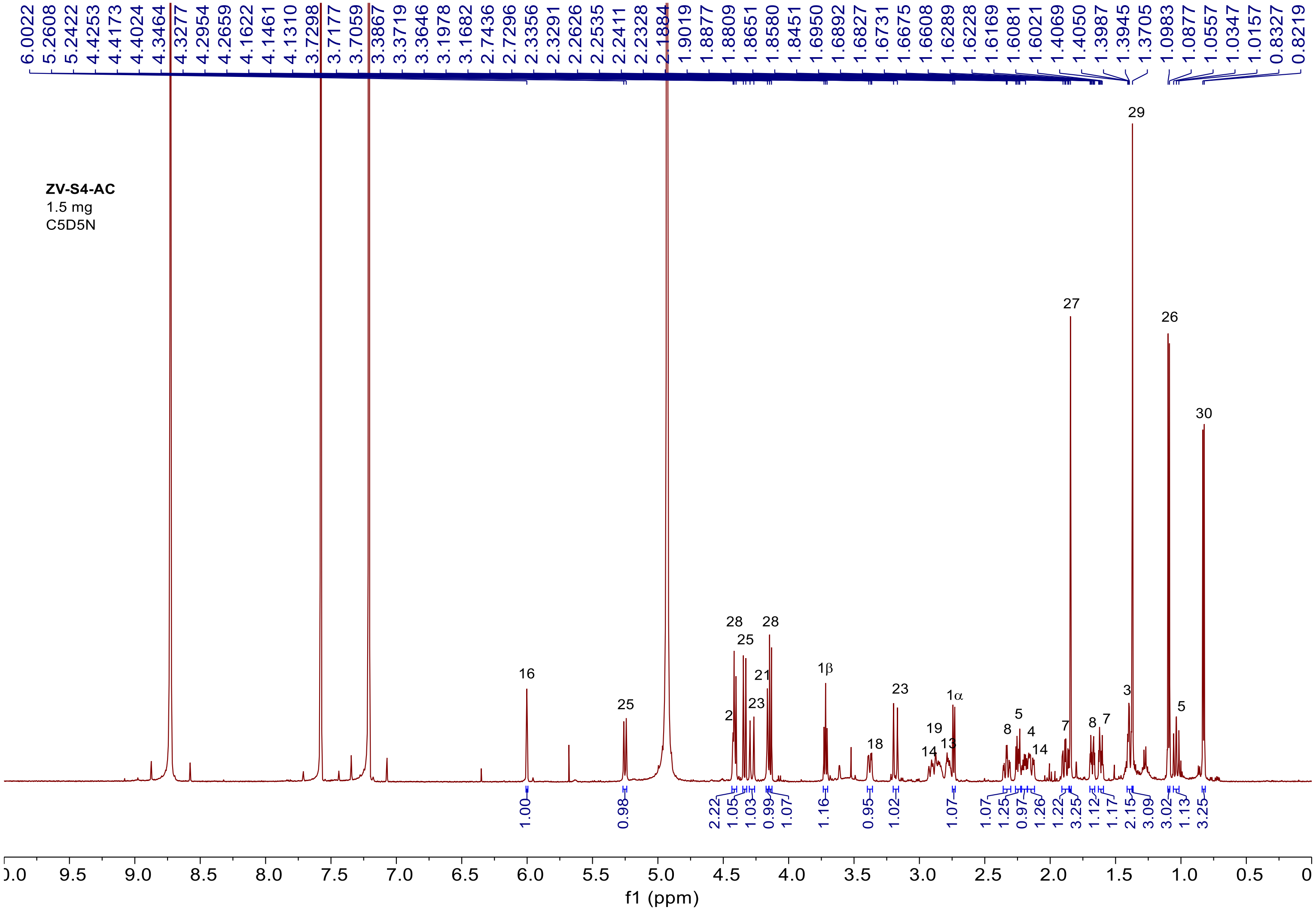

Figure S20. $^{13}C\{^{1}H\}$ NMR and DEPT spectra of **3** (C_5D_5N, 150 MHz)

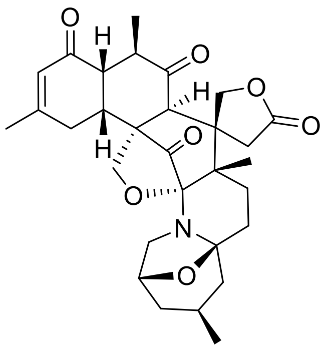

3

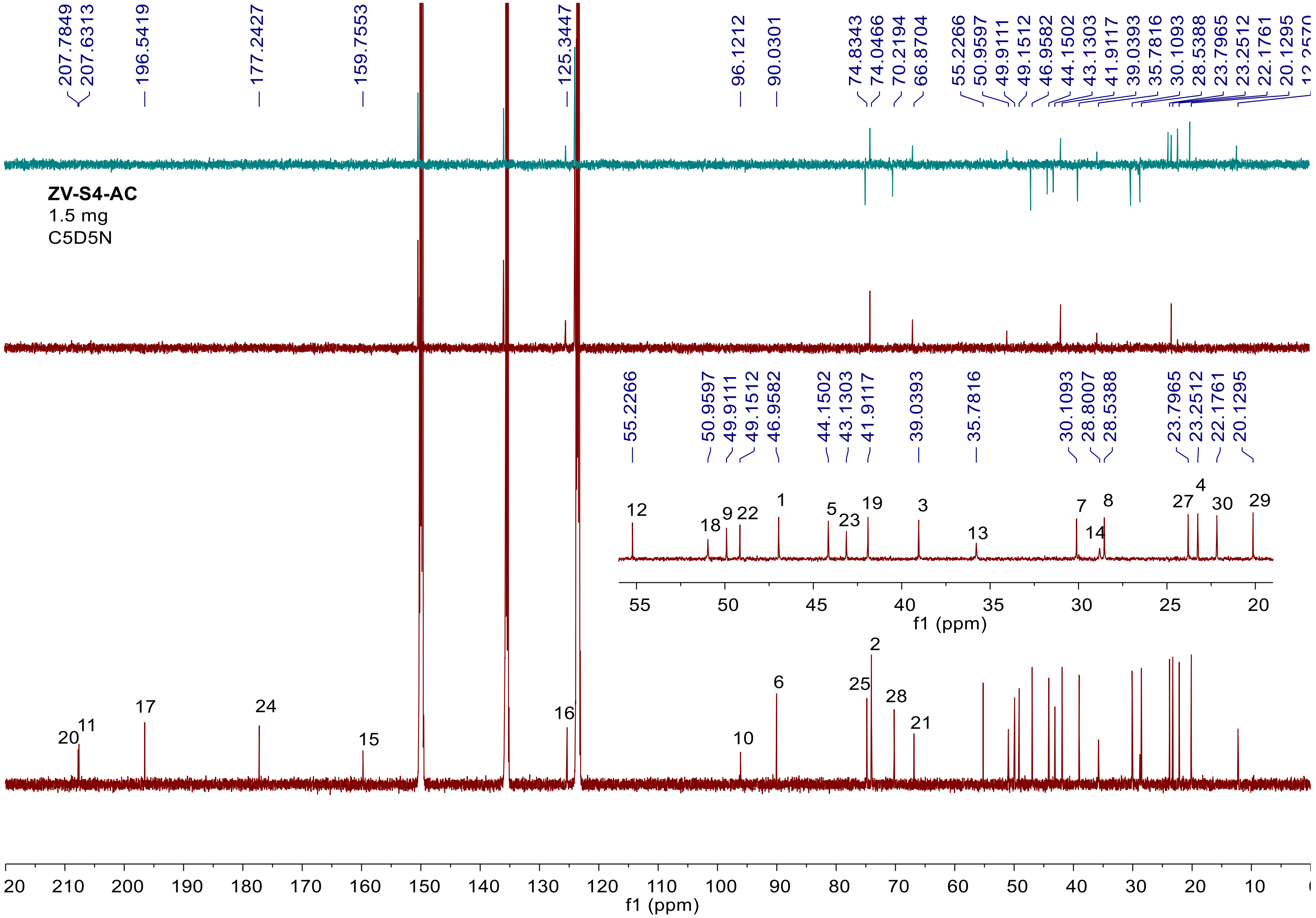

Figure S21. COSY spectrum of **3**

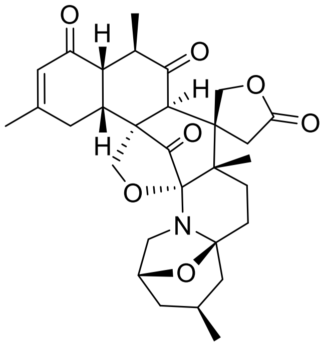

3

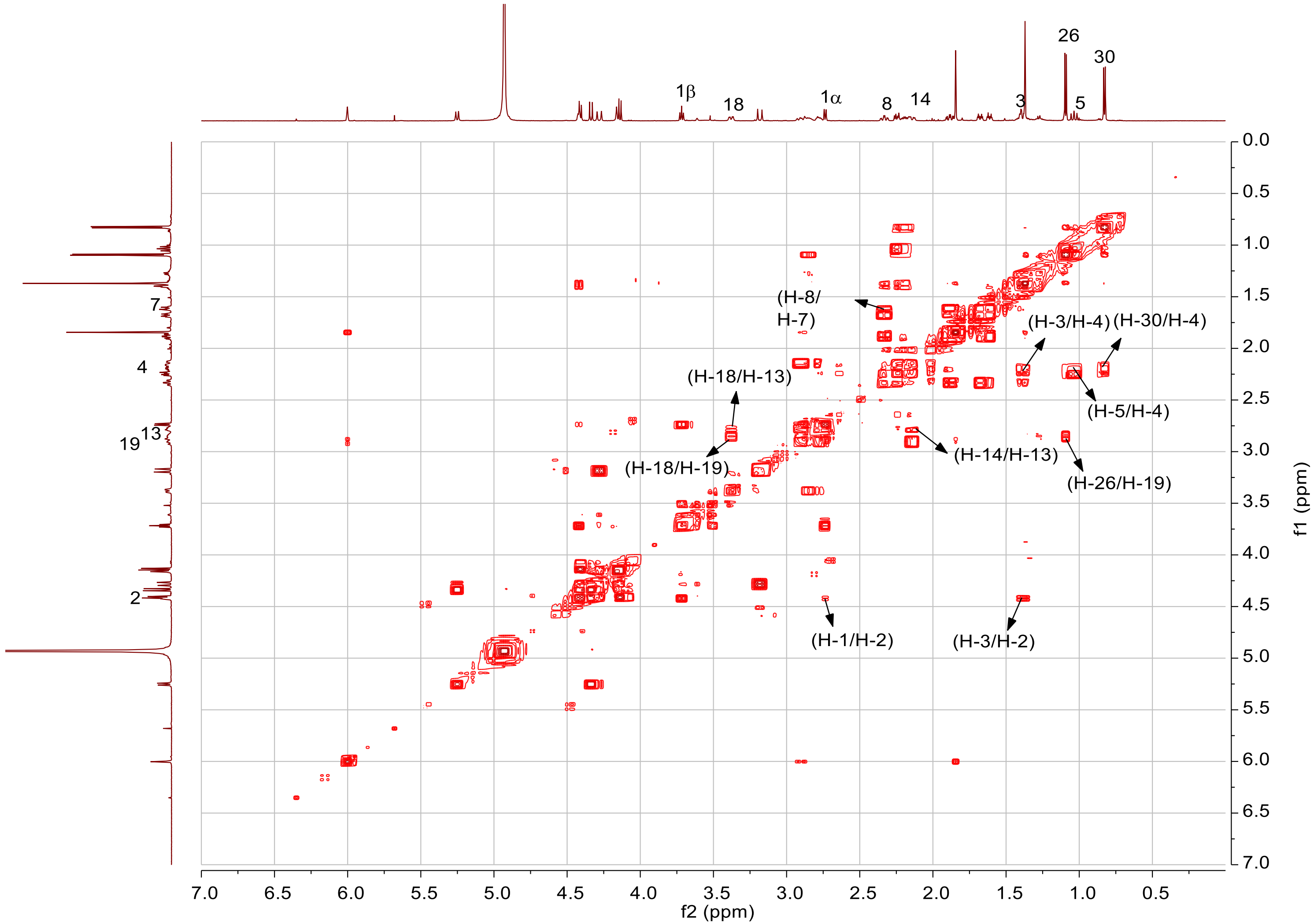

Figure S22. HSQC spectrum of **3**

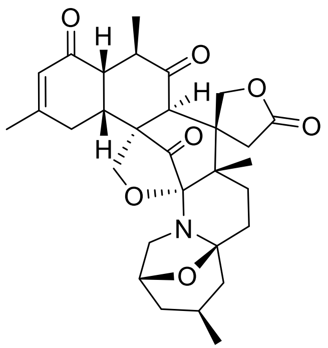

3

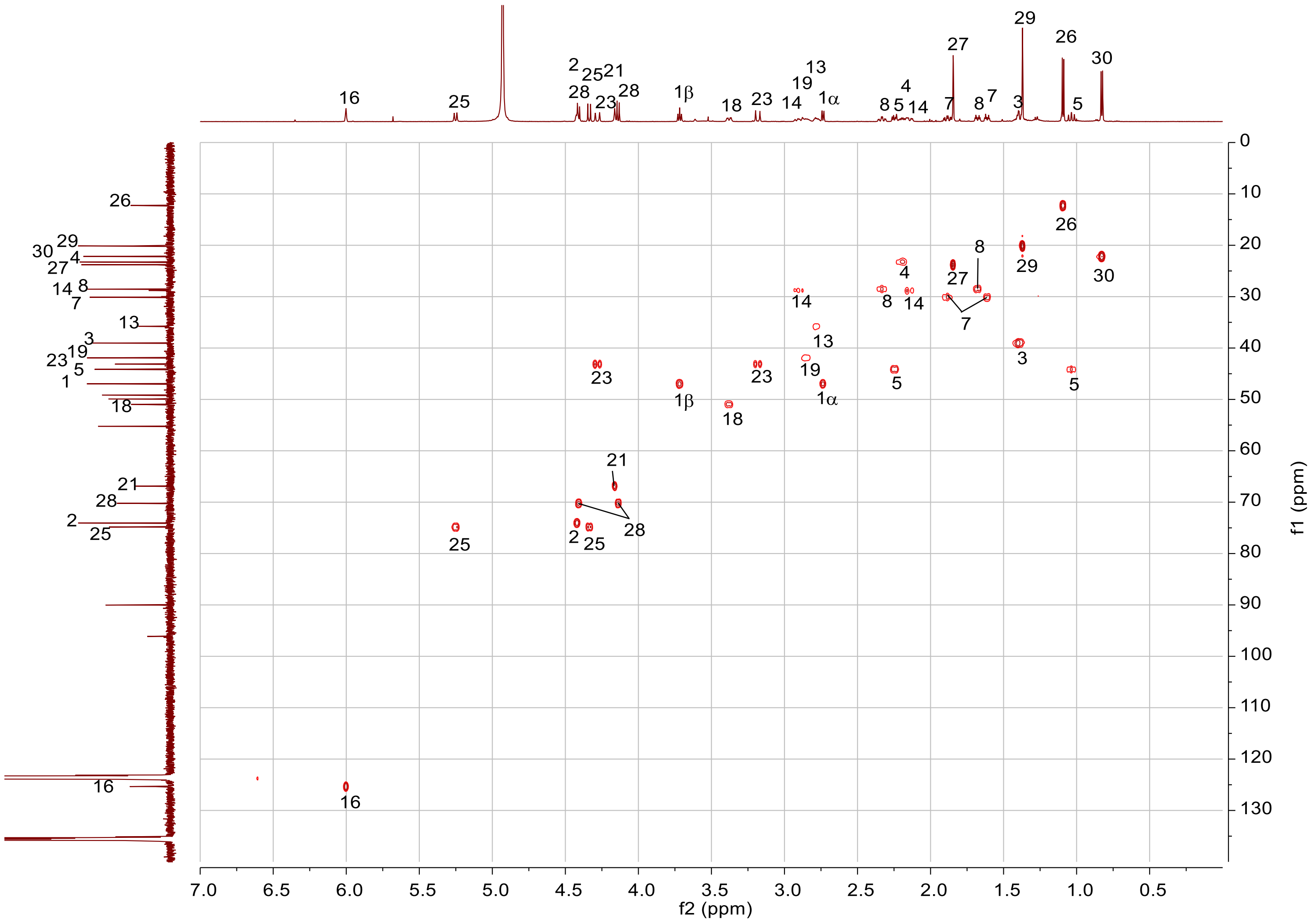

Figure S23. HMBC spectrum of **3**

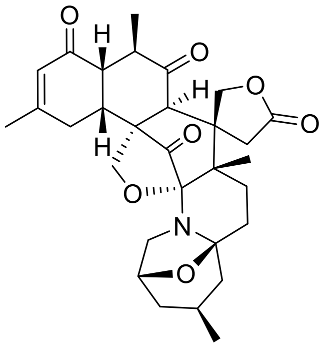

3

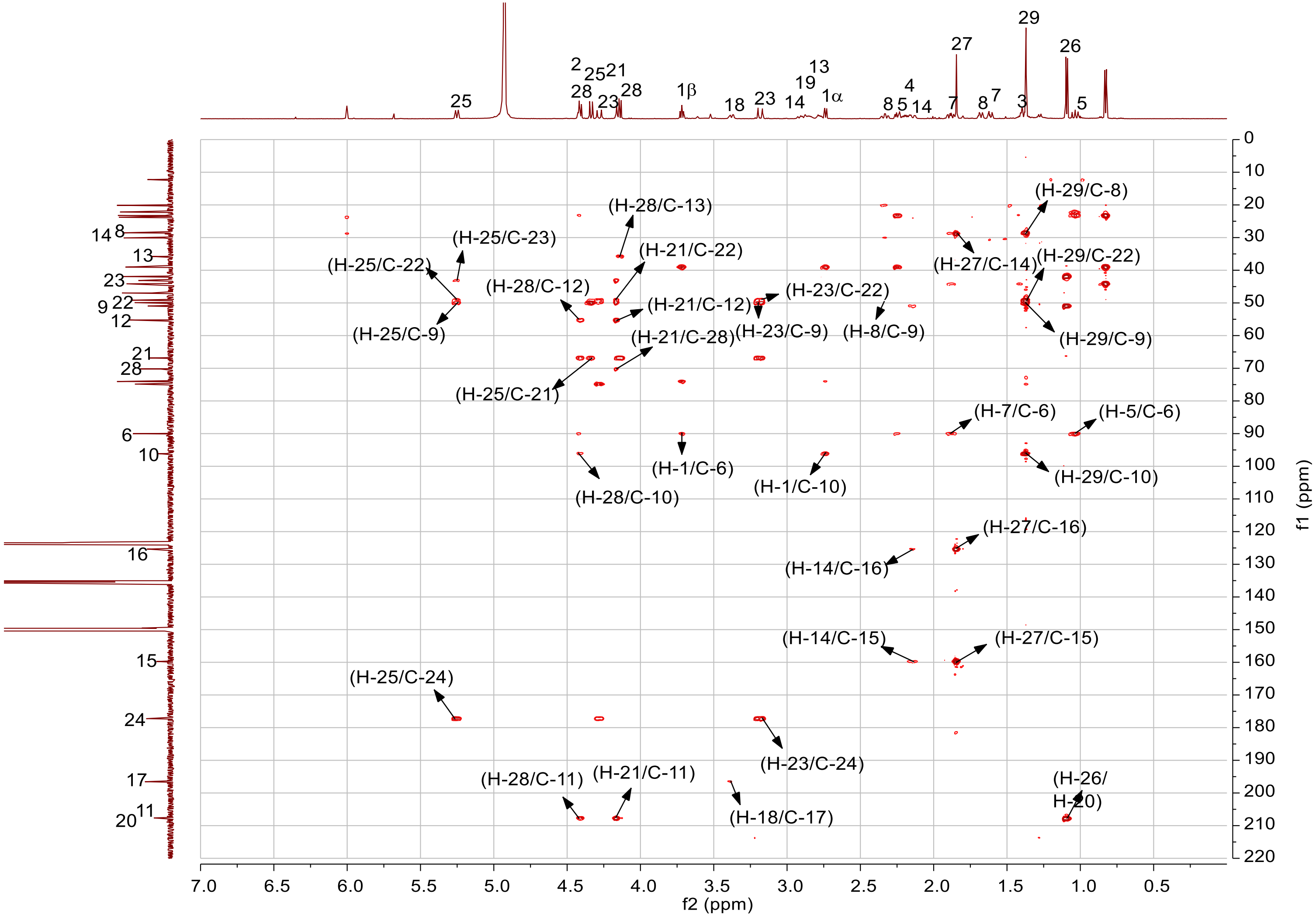

Figure S24. NOESY spectrum of **3**

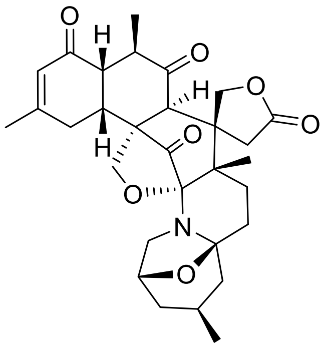

3

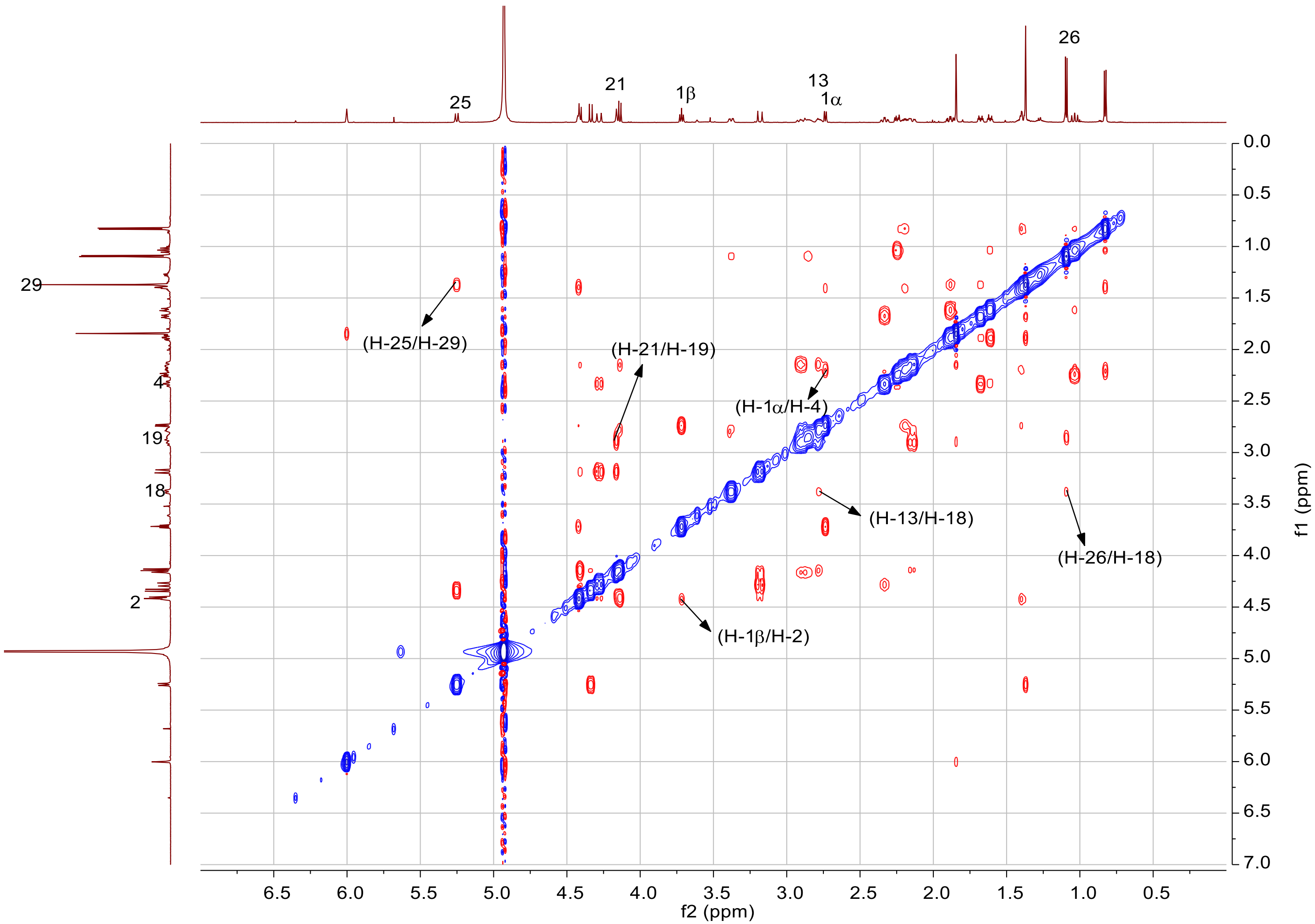

Figure S25. HRESIMS spectrum of **3**

Mass Spectrum SmartFormula Report

Analysis Info

Analysis Name	D:\0727\ZV-S4-AC.d	7/28/2022 11:23:52 AM
Method	tune_wide_pos_20220422.m	Operator: YU HSIAO-CHING
Sample Name	ZV-S4-AV	Instrument: BRUKER micrOTOF-Q
Comment	ESI Positive	

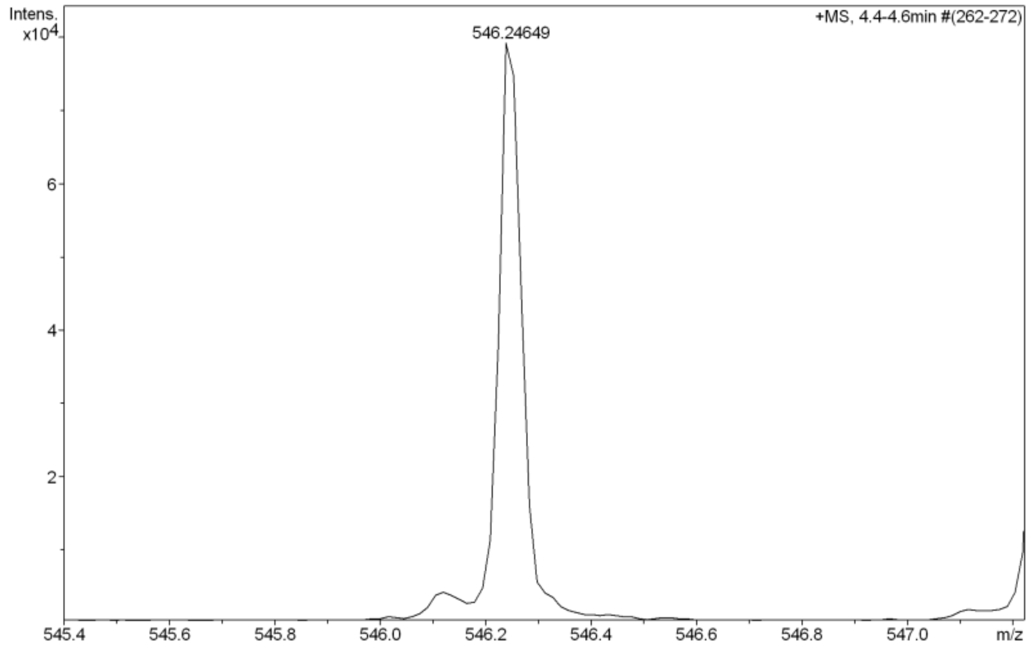

Meas. m/z	#	Formula	Score	m/z	err [mDa]	err [ppm]	mSigma	rdb	e⁻ Conf	N-Rule
546.24649	1	C 30 H 37 N Na O 7	100.00	546.24622	-0.27	-0.49	40.6	12.5	even	ok

Figure S26. UV spectrum of **3**

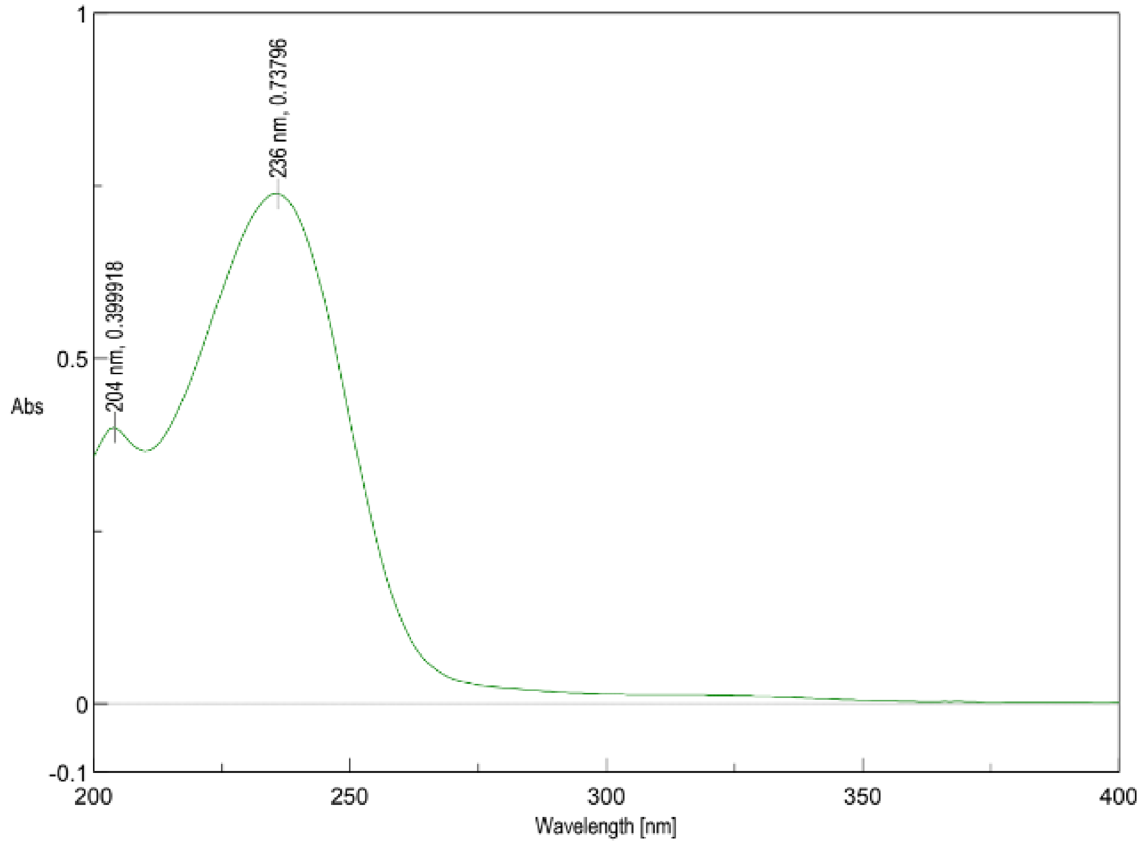

Figure S27. IR spectrum of **3**

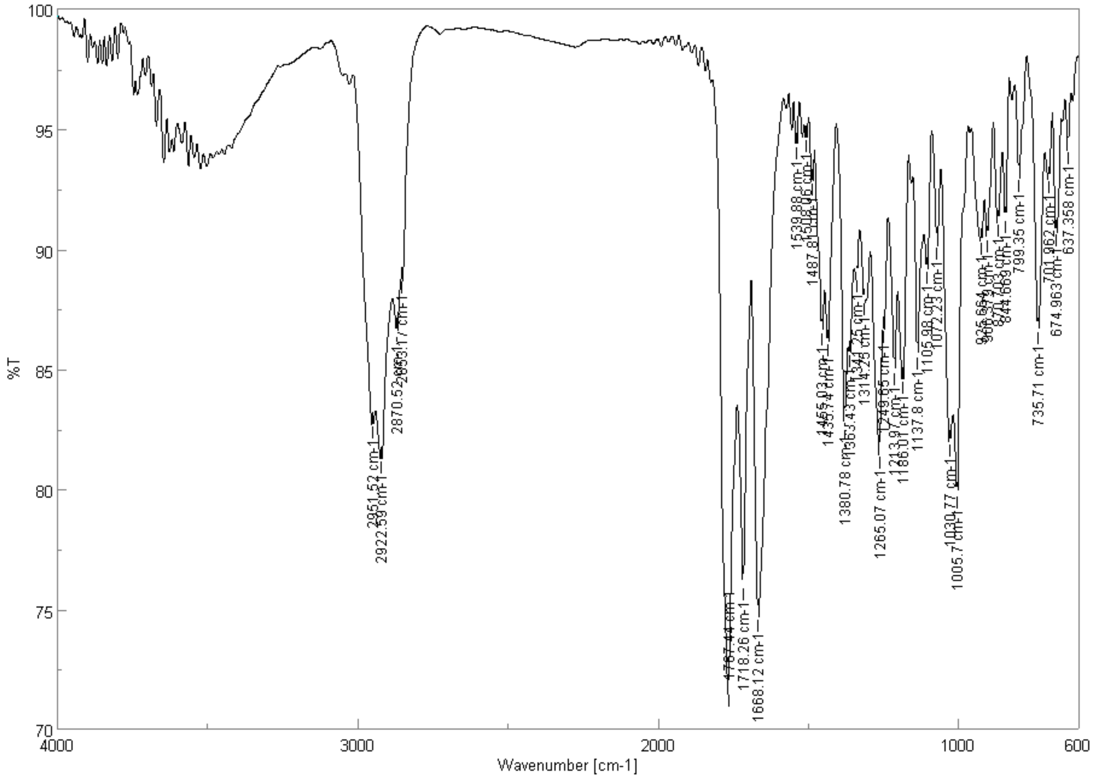

Figure S28. ^{1}H NMR spectrum of **4** (C_5D_5N, 400 MHz)

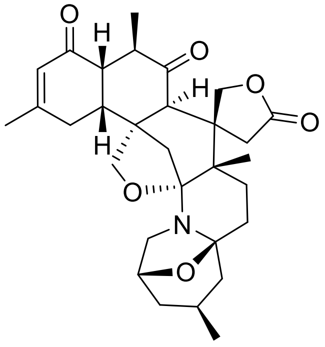

4

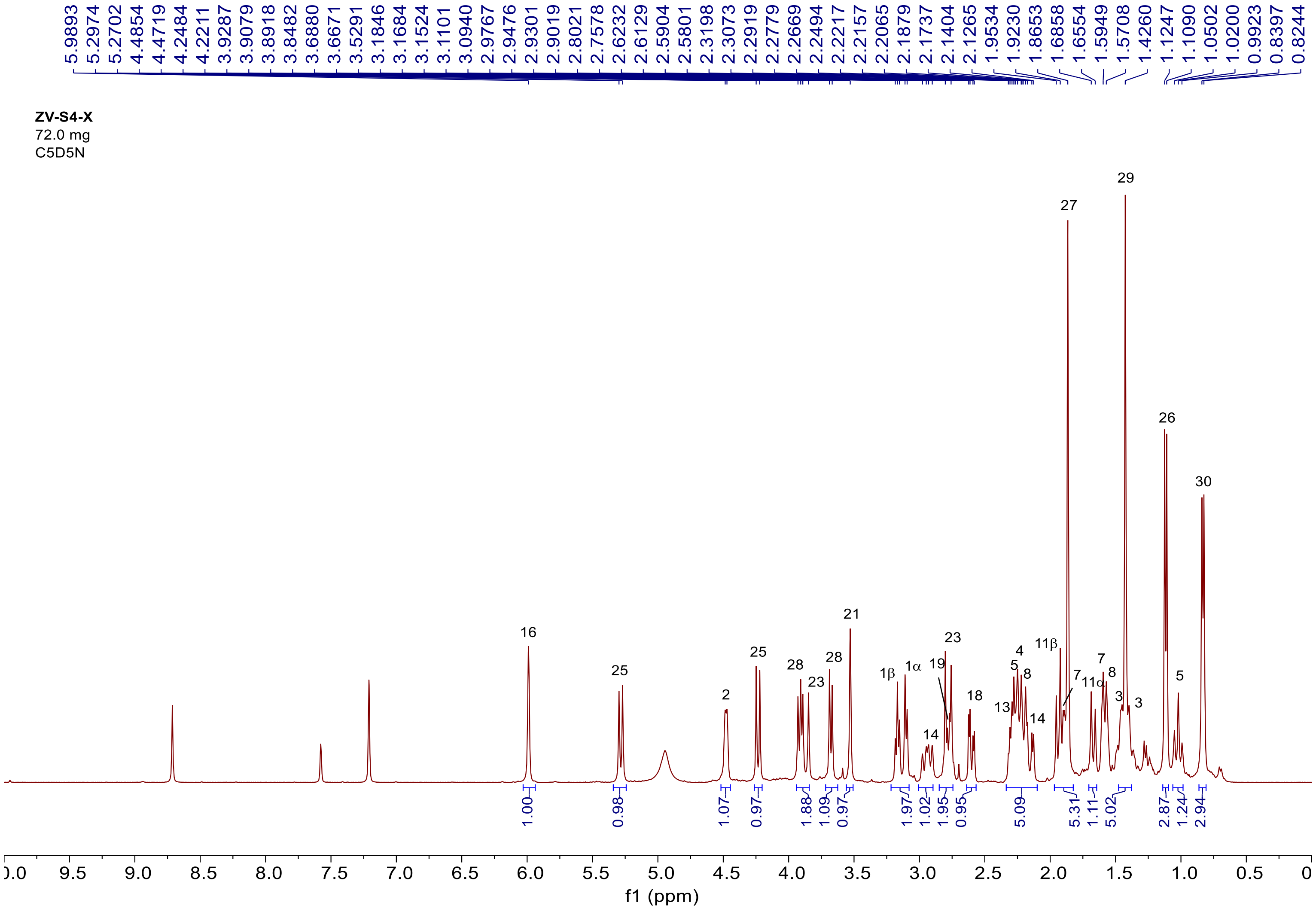

Figure S29. $^{13}C\{^1H\}$ NMR and DEPT spectra of **4** (C_5D_5N, 100 MHz)

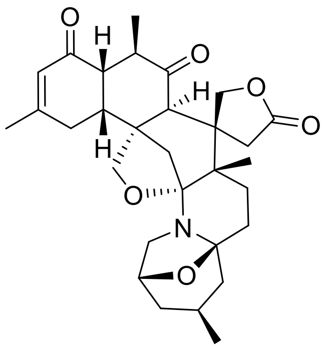
4

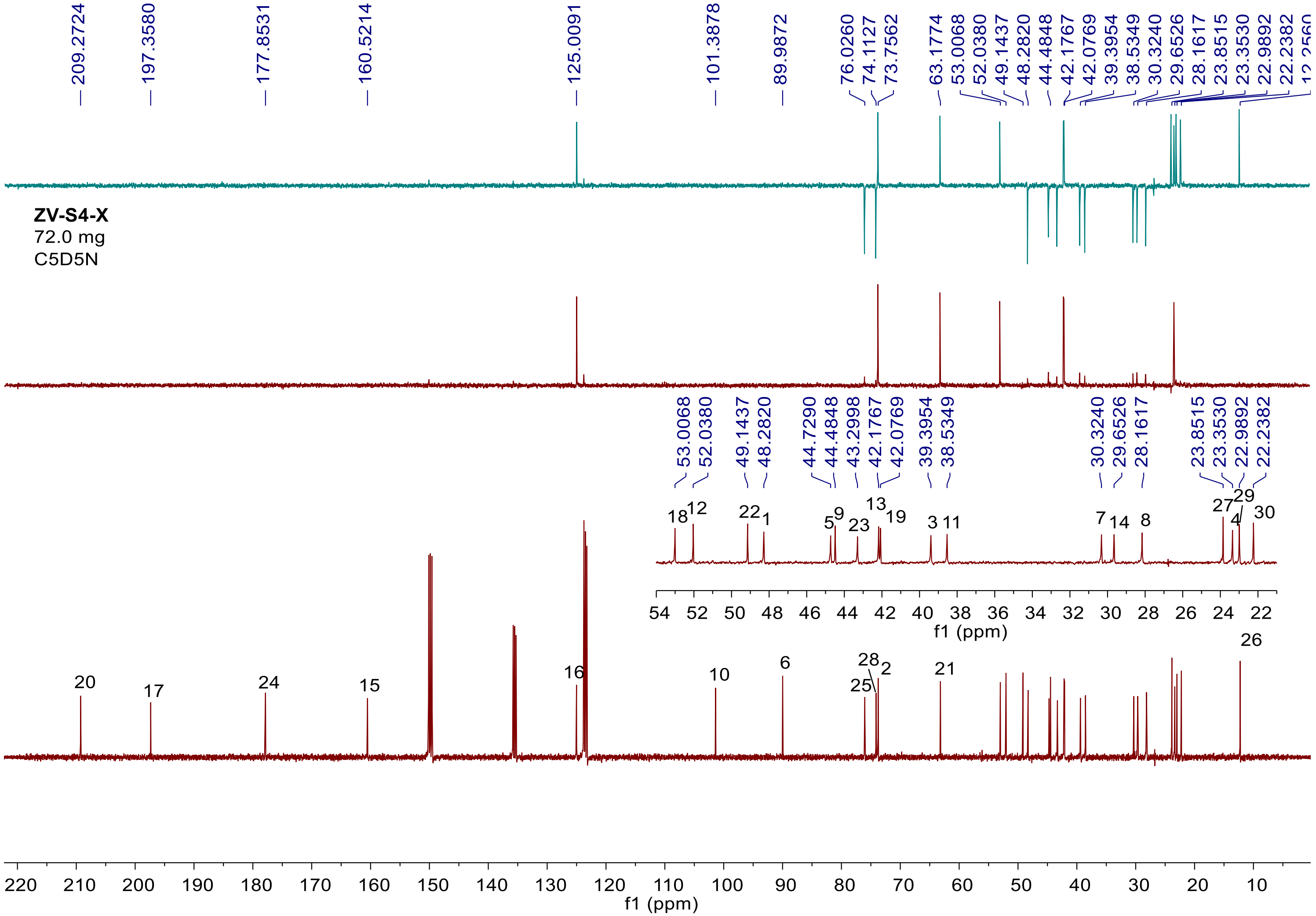

Figure S30. COSY spectrum of **4**

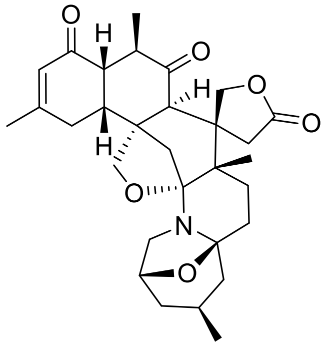

4

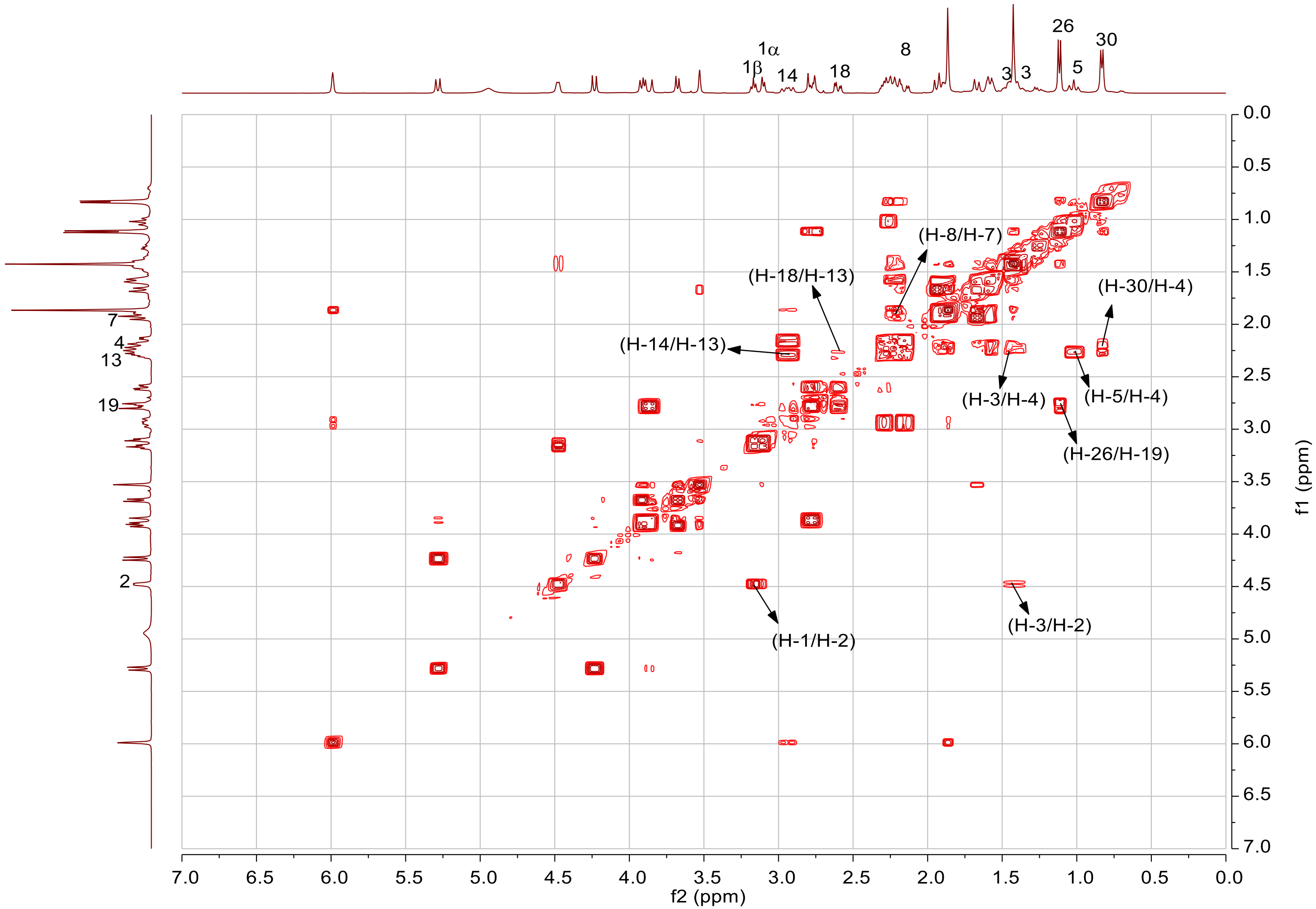

Figure S31. HSQC spectrum of **4**

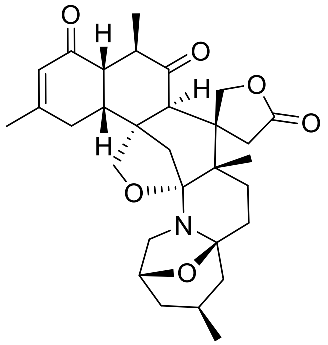

4

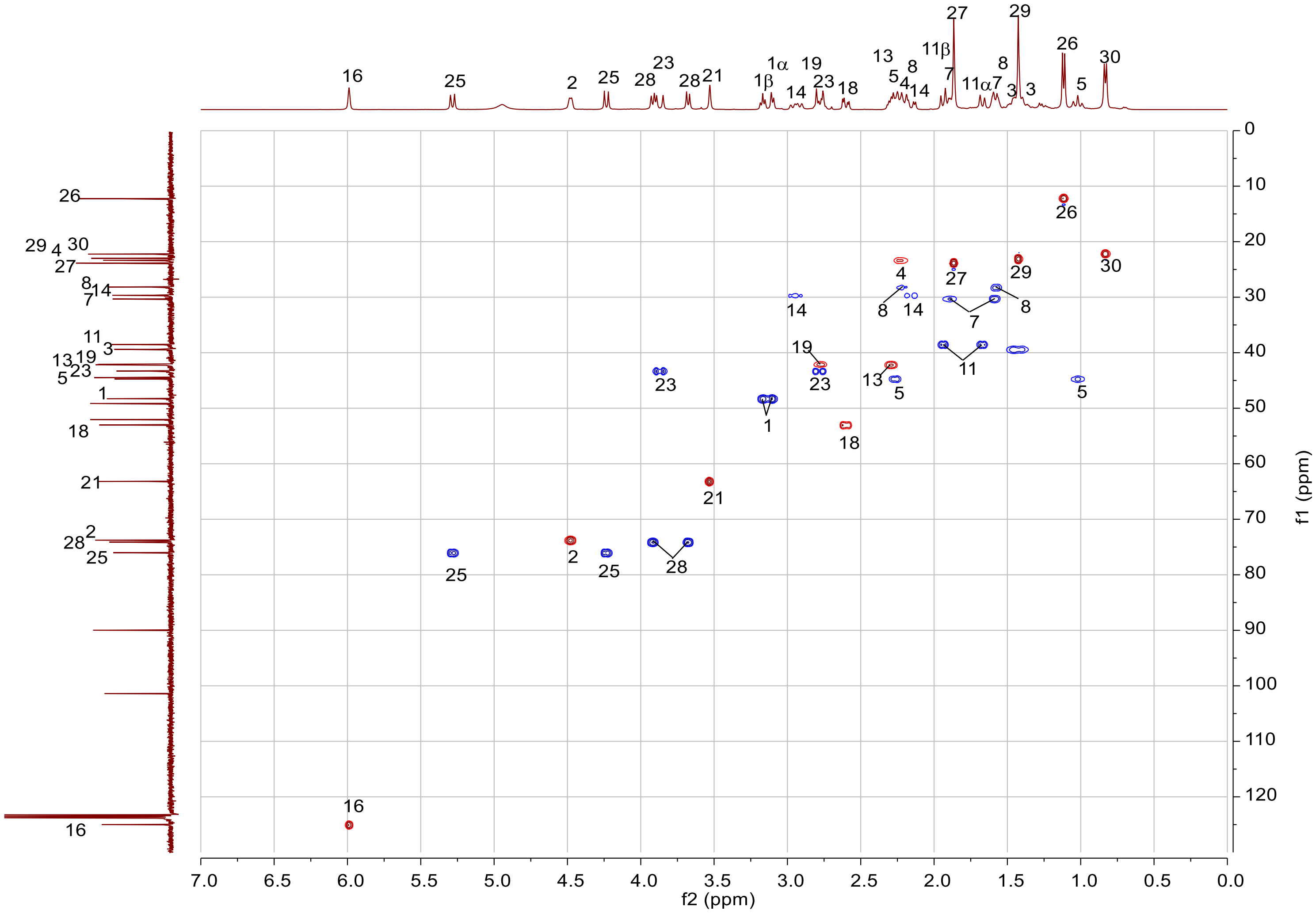

Figure S32. HMBC spectrum of **4**

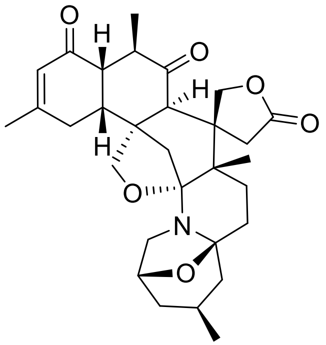

4

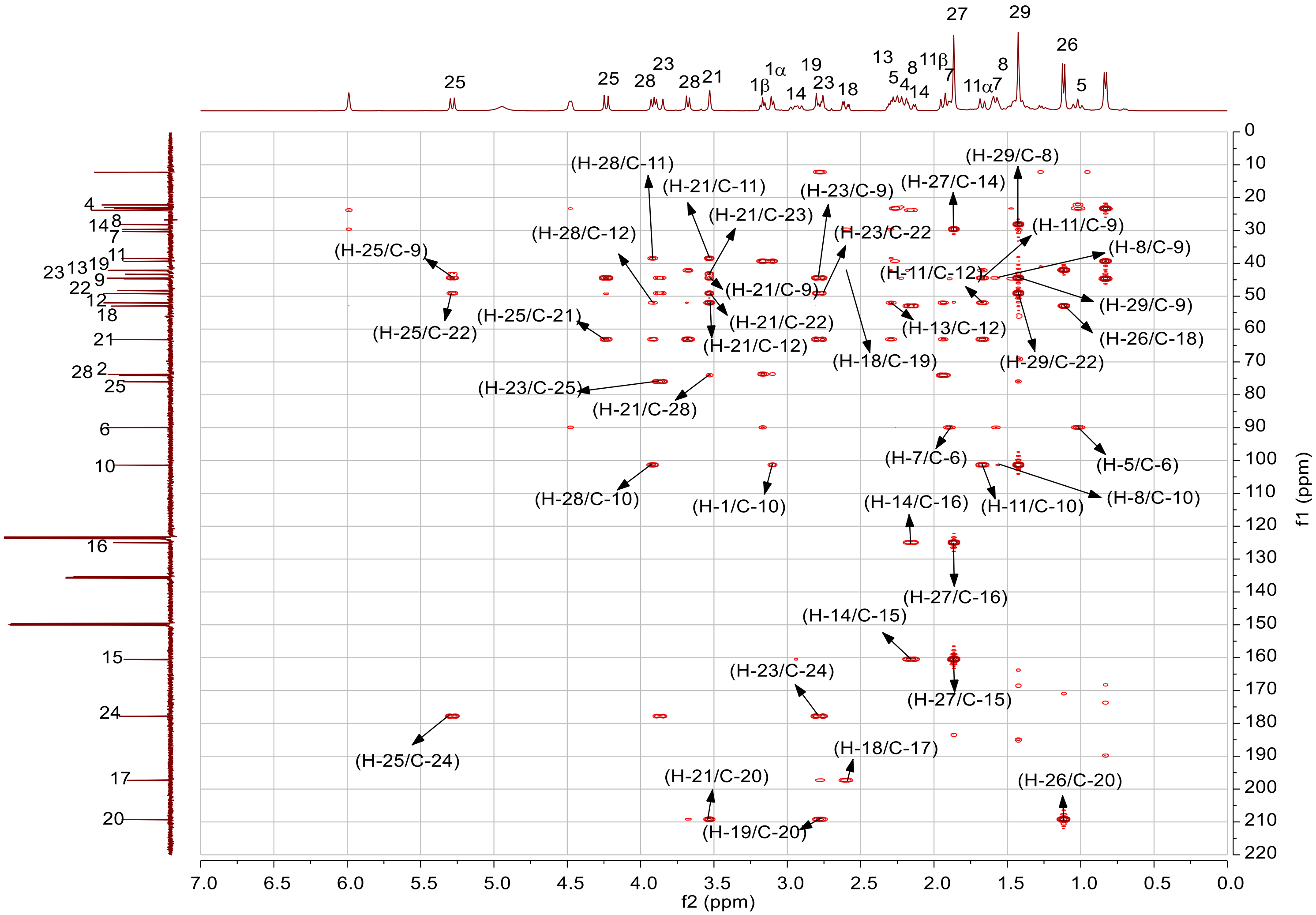

Figure S33. NOESY spectrum of **4**

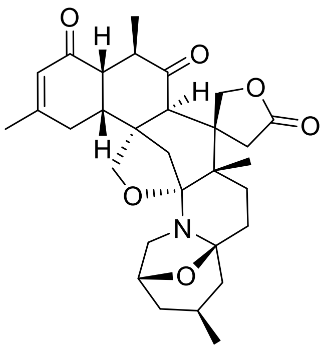

4

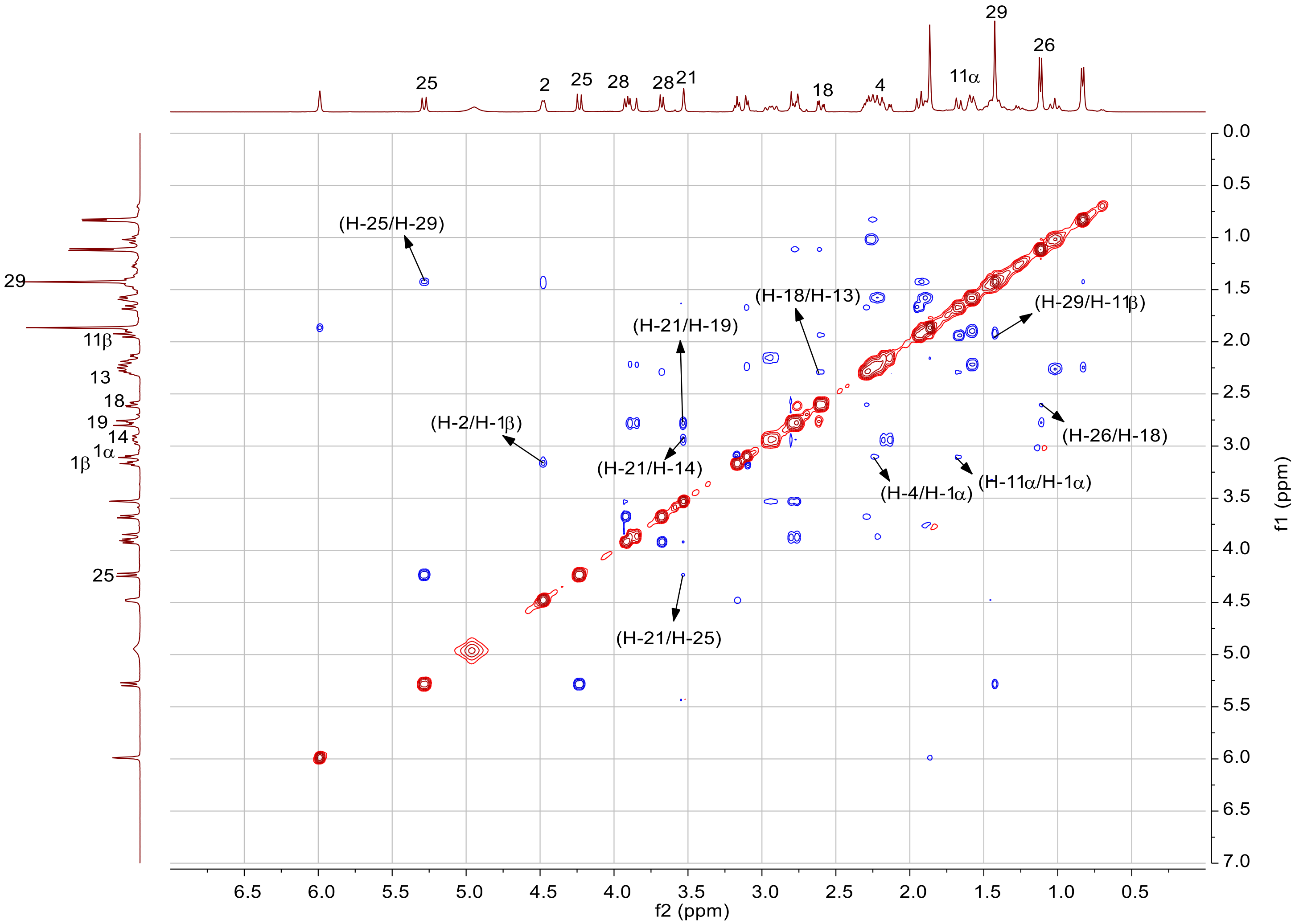

Figure S34. HRESIMS spectrum of **4**

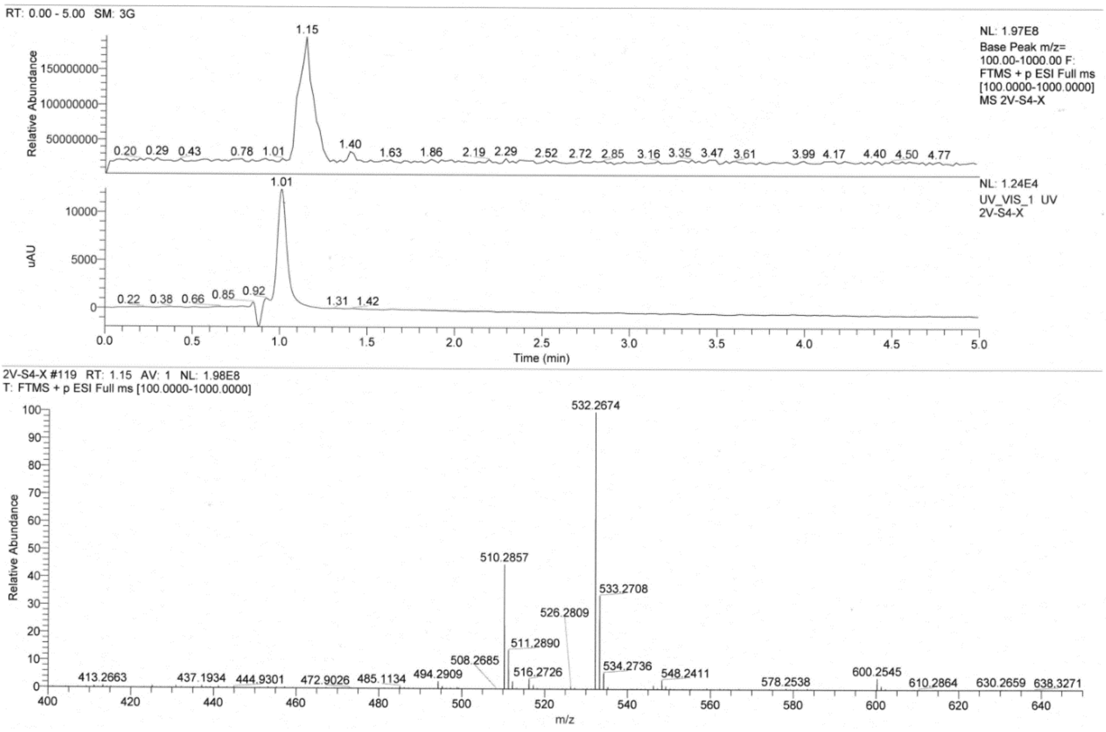

Elemental composition search on mass 532.2687

m/z= 527.2700-537.2674

m/z	Theo. Mass	Delta (mmu)	RDB equiv.	Composition
532.2674	532.2670	0.44	11.5	$C_{30}H_{39}O_6NNa$
	532.2667	0.72	10.0	$C_{29}H_{40}O_9$
	532.2694	-1.96	14.5	$C_{32}H_{38}O_6N$
	532.2643	3.12	7.0	$C_{27}H_{41}O_9Na$
	532.2635	3.91	23.5	$C_{39}H_{34}ON$
	532.2726	-5.16	1.0	$C_{22}H_{44}O_{14}$
	532.2728	-5.43	2.5	$C_{23}H_{43}O_{11}NNa$
	532.2737	-6.26	20.0	$C_{38}H_{37}ONa$
	532.2611	6.31	20.5	$C_{37}H_{35}ONNa$
	532.2608	6.59	19.0	$C_{36}H_{36}O_4$

Figure S35. UV spectrum of **4**

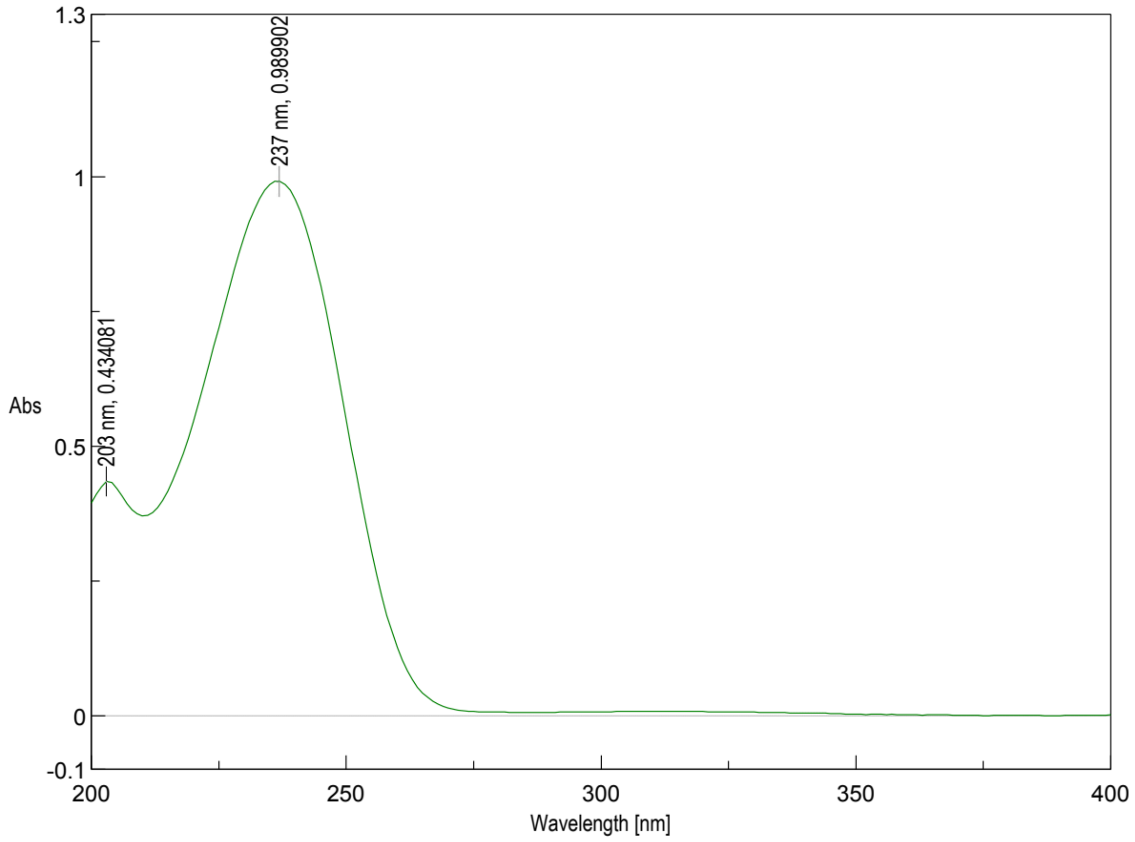

Figure S36. IR spectrum of **4**

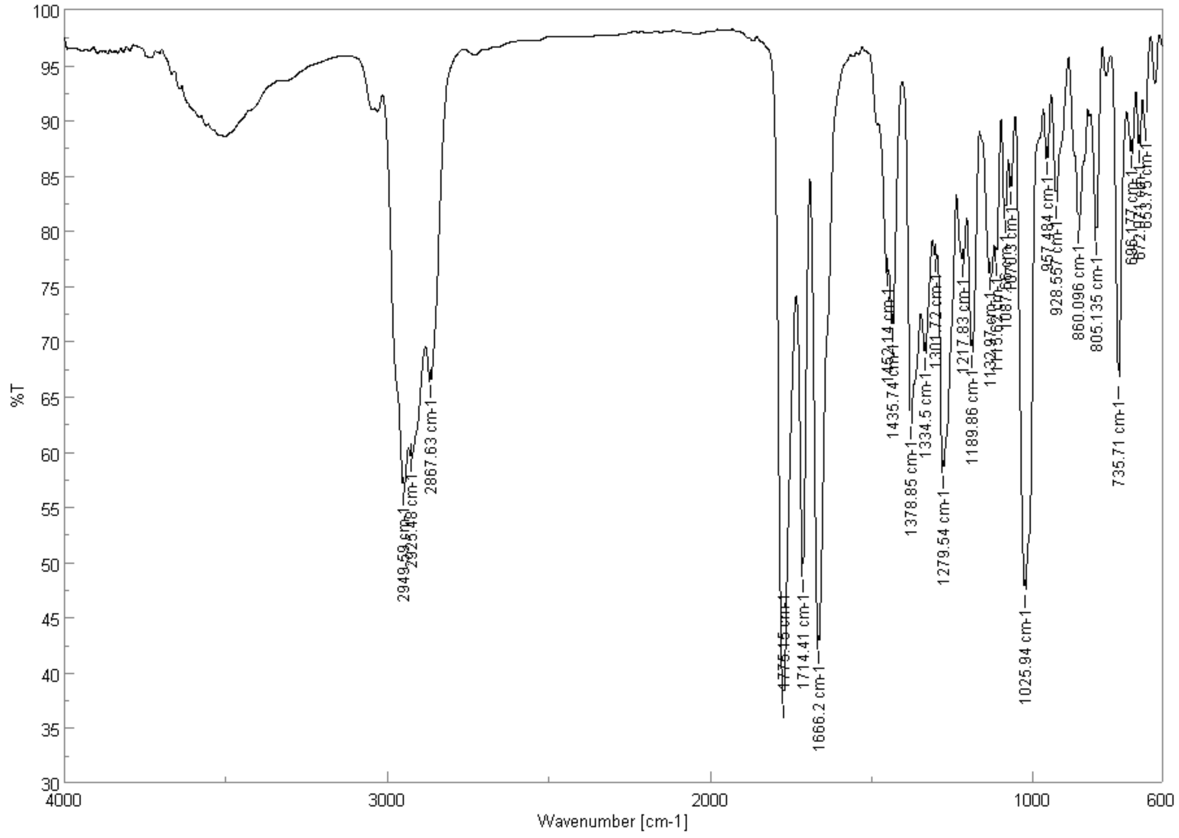

Figure S37. ^{1}H NMR spectrum of **5** (C_5D_5N, 400 MHz)

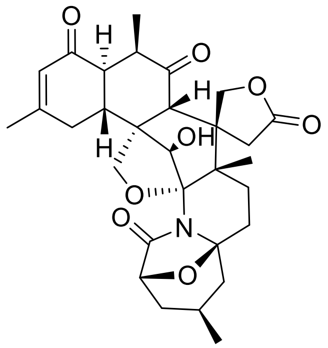

5

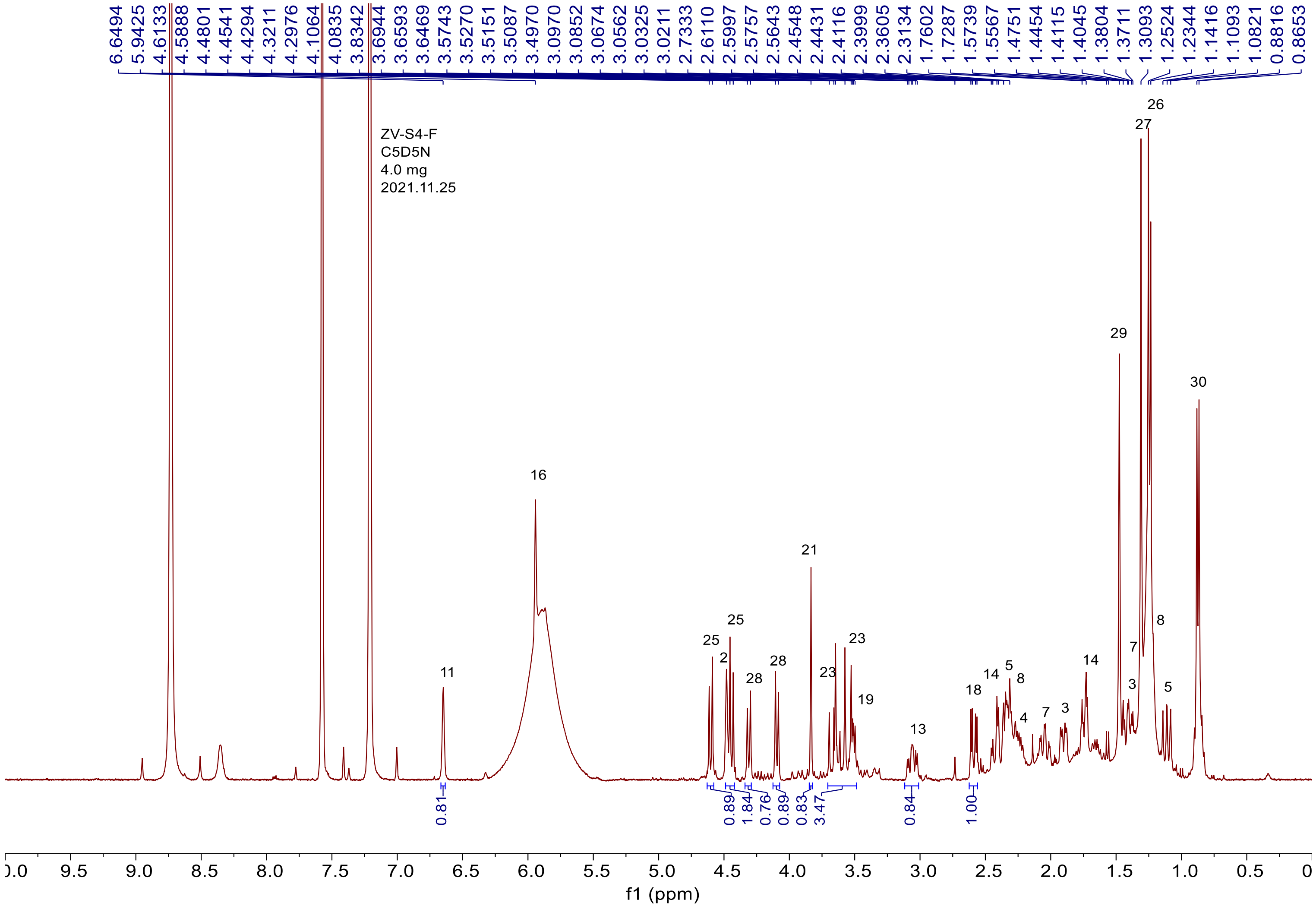

Figure S38. $^{13}C\{^{1}H\}$ NMR and DEPT spectra of **5** (C_5D_5N, 100 MHz)

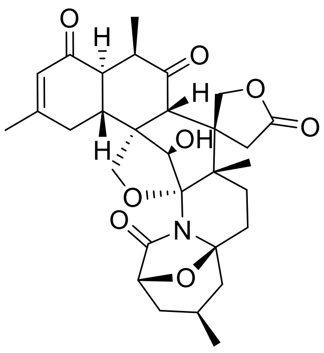

5

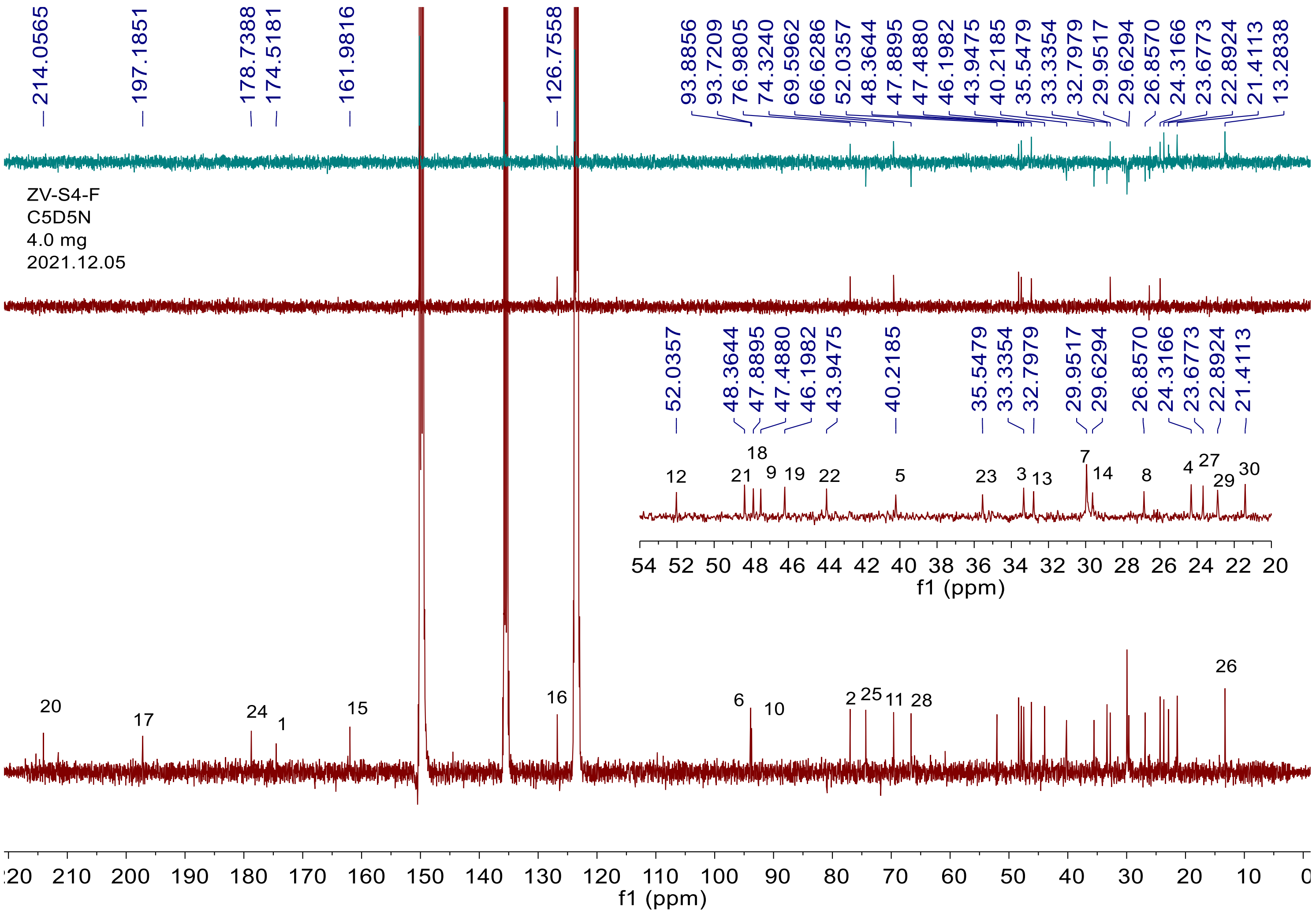

Figure S39. COSY spectrum of **5**

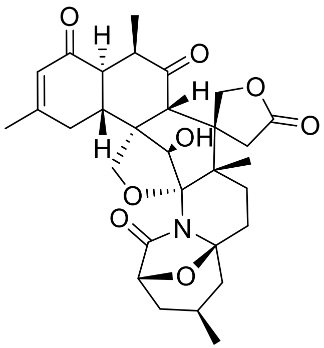

5

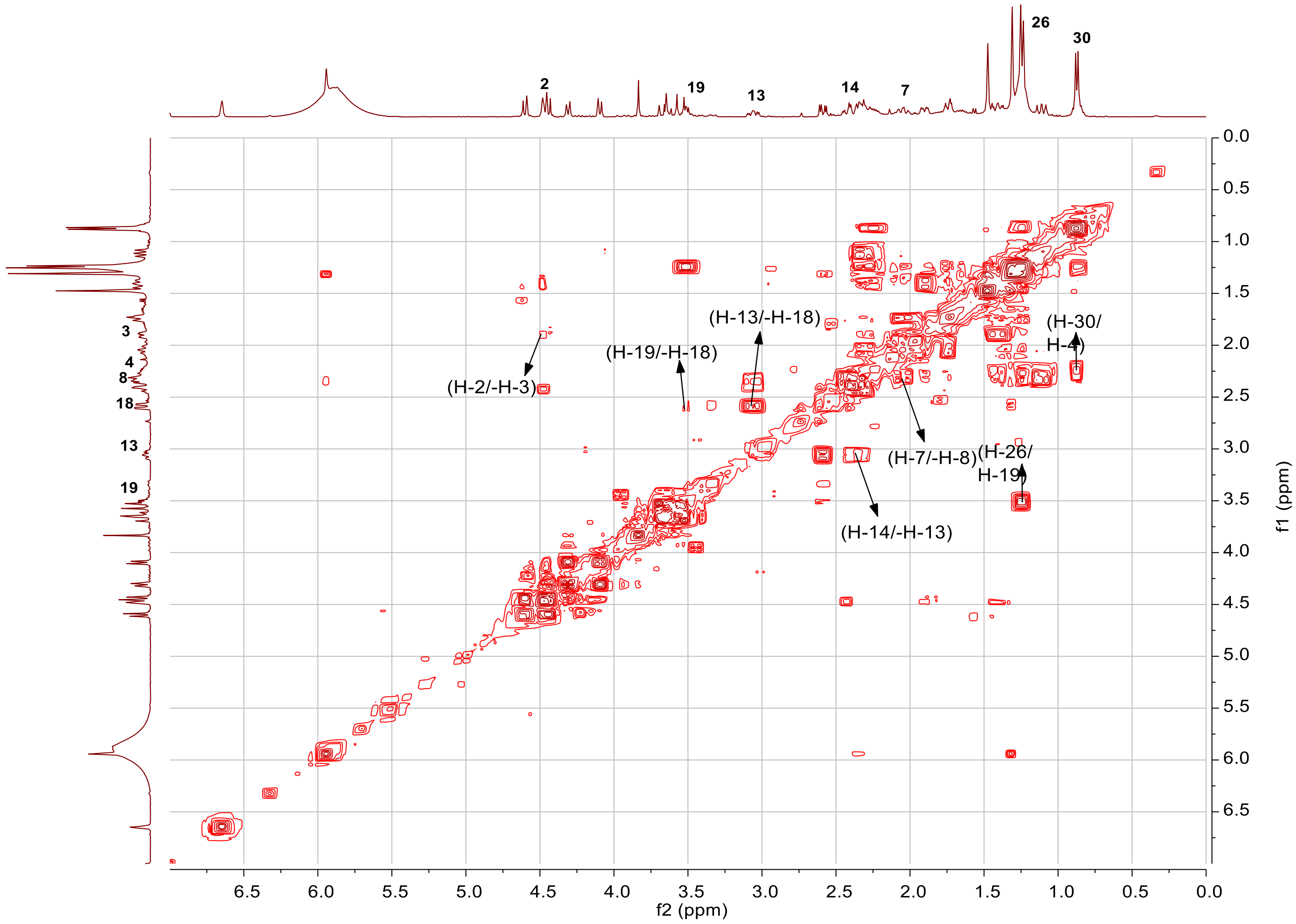

Figure S40. HSQC spectrum of **5**

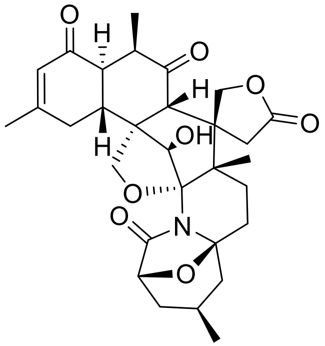

5

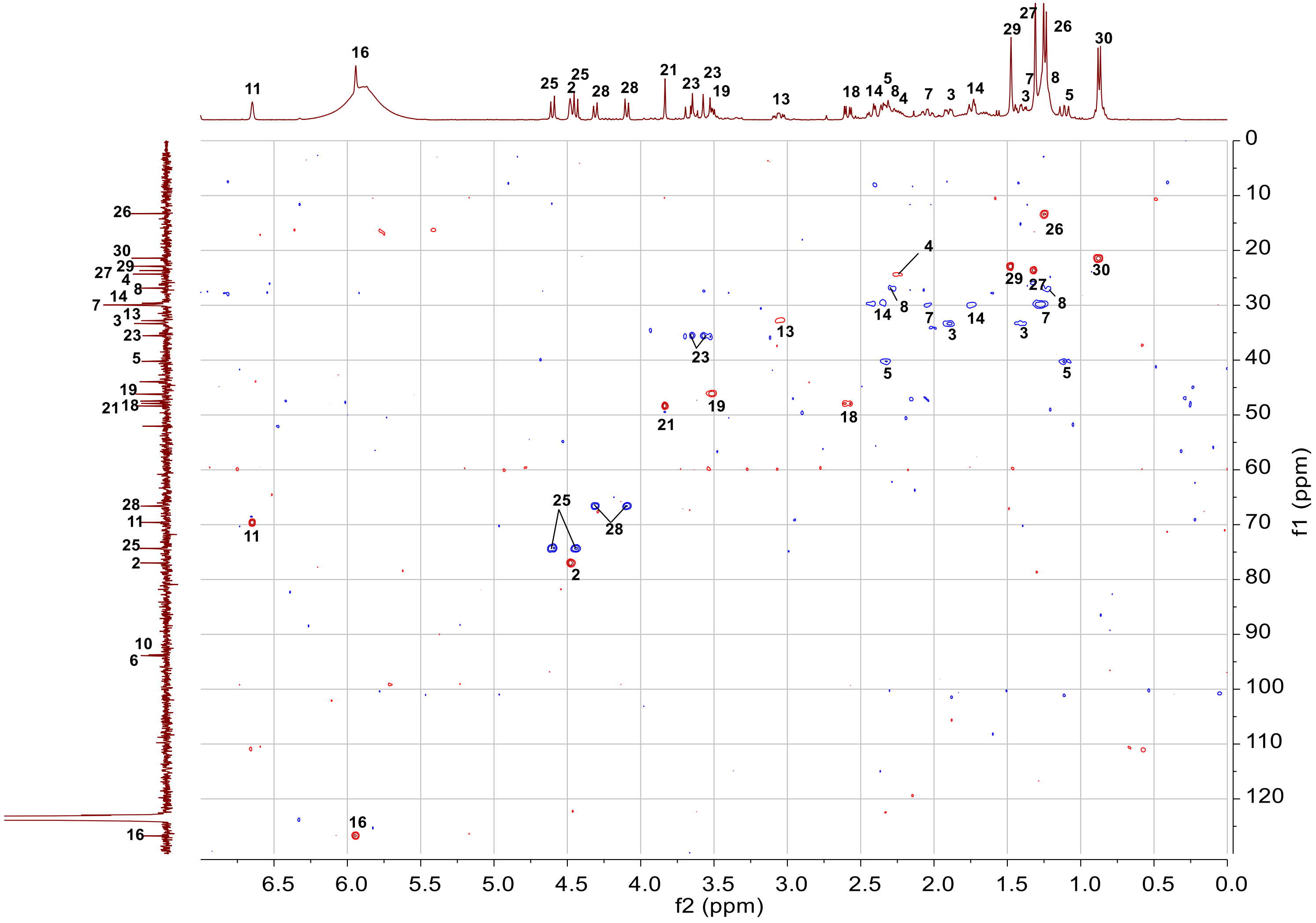

Figure S41. HMBC spectrum of **5**

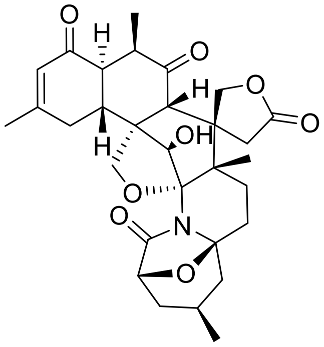

5

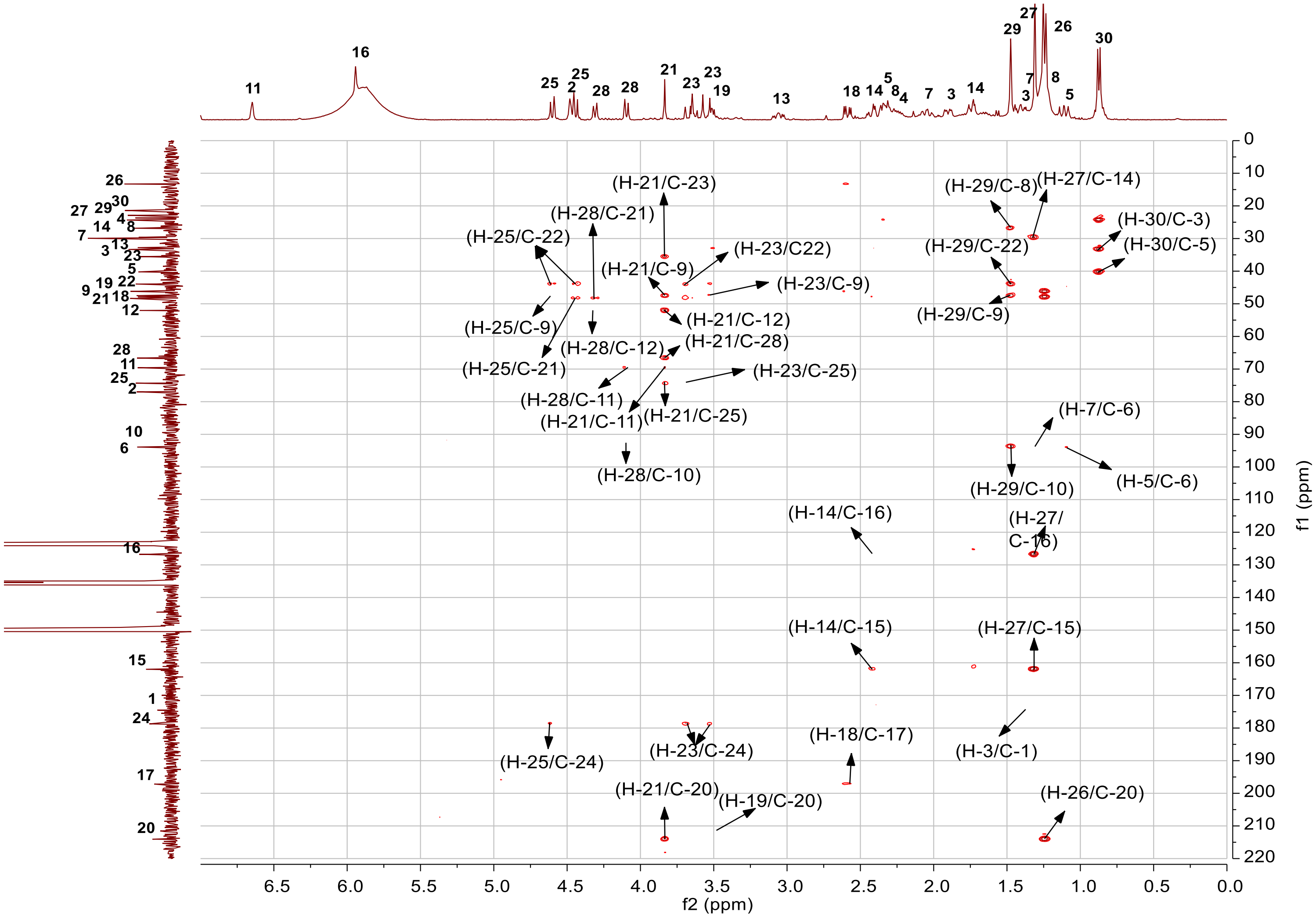

Figure S42. NOESY spectrum of **5**

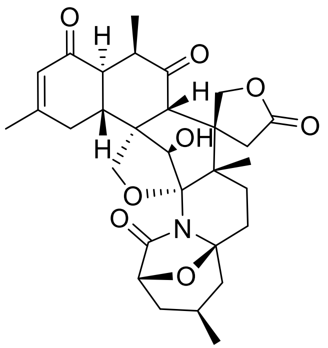

5

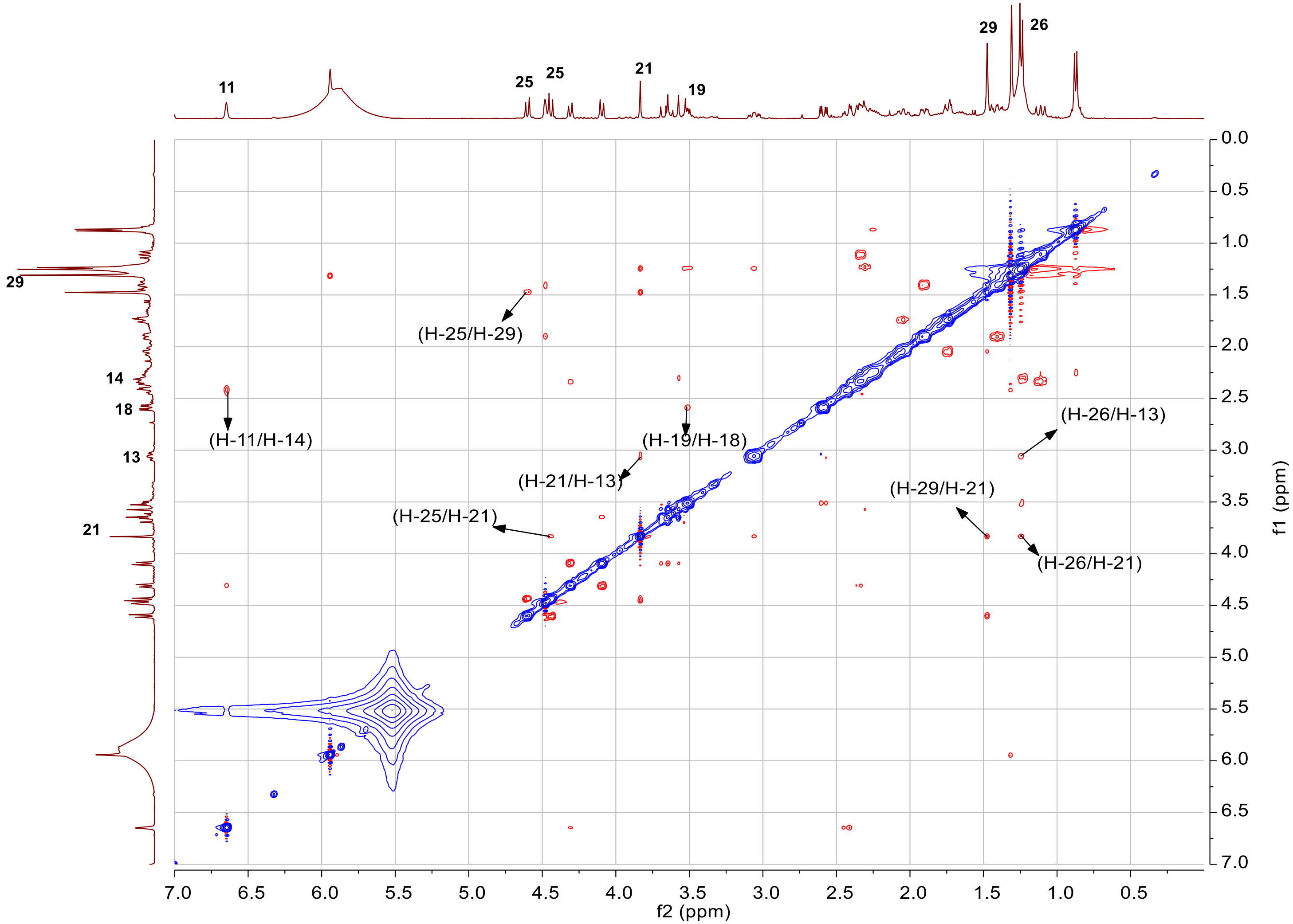

Figure S43. HRESIMS spectrum of **5**

Mass Spectrum SmartFormula Report

Analysis Info

Analysis Name	D:\QTOF\ZVS4FR1.d	2/10/2022 12:58:51 PM
Method	_tune_low_pos(50-1000)_20170502_Liao lab.m	Operator: YU HSIAO-CHING
Sample Name	ZV-S4-F	Instrument: BRUKER micrOTOF-Q
Comment		

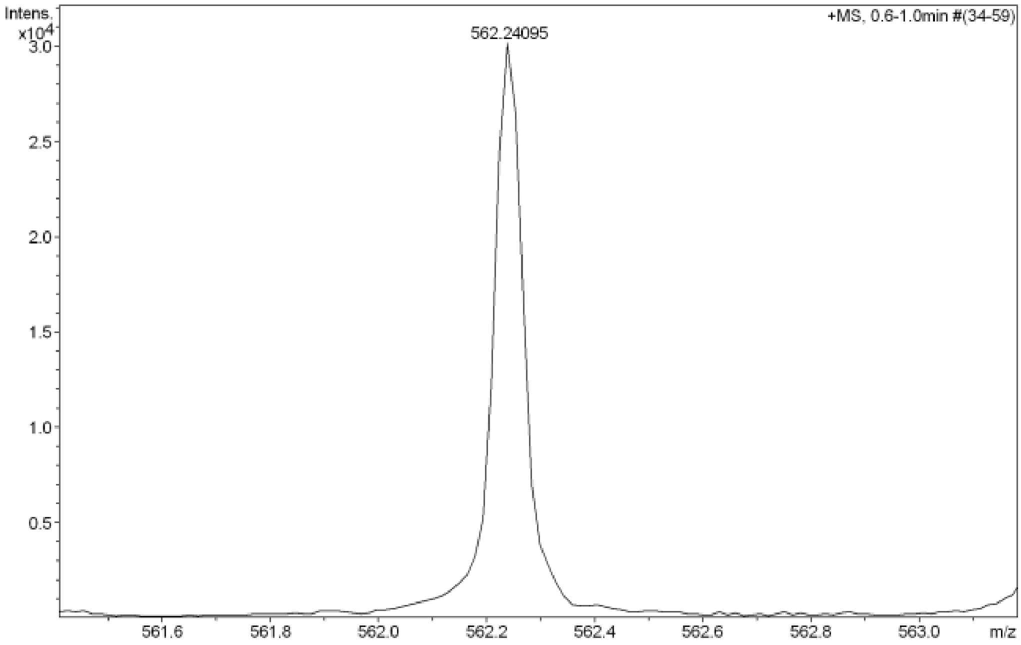

Meas. m/z	#	Formula	Score	m/z	err [mDa]	err [ppm]	mSigma	rdb	e⁻ Conf	N-Rule
562.24095	1	C 30 H 37 N Na O 8	100.00	562.24114	0.19	0.33	54.7	12.5	even	ok

Figure S44. UV spectrum of **5**

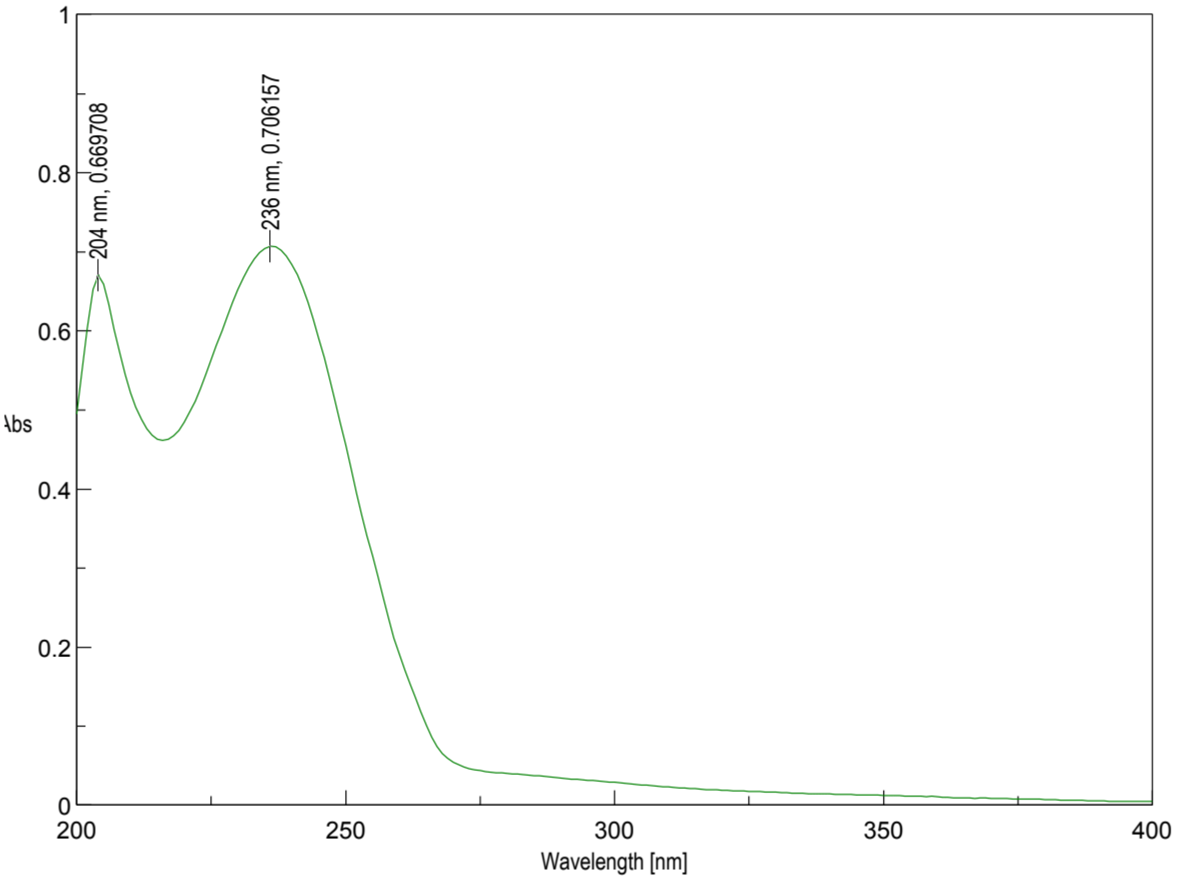

Figure S45. IR spectrum of **5**

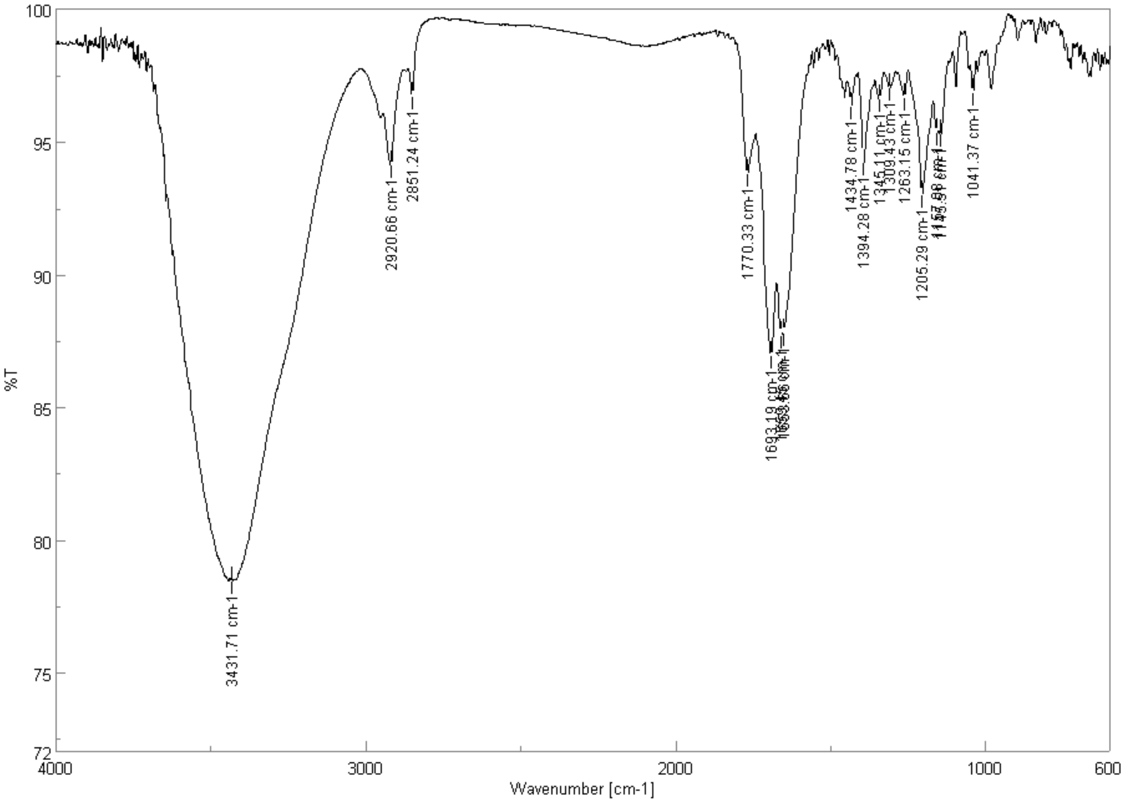

Figure S46. ^{1}H NMR spectrum of **6** (C_5D_5N, 600 MHz)

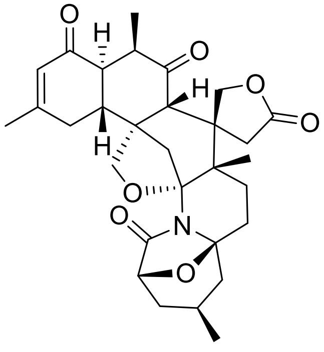

6

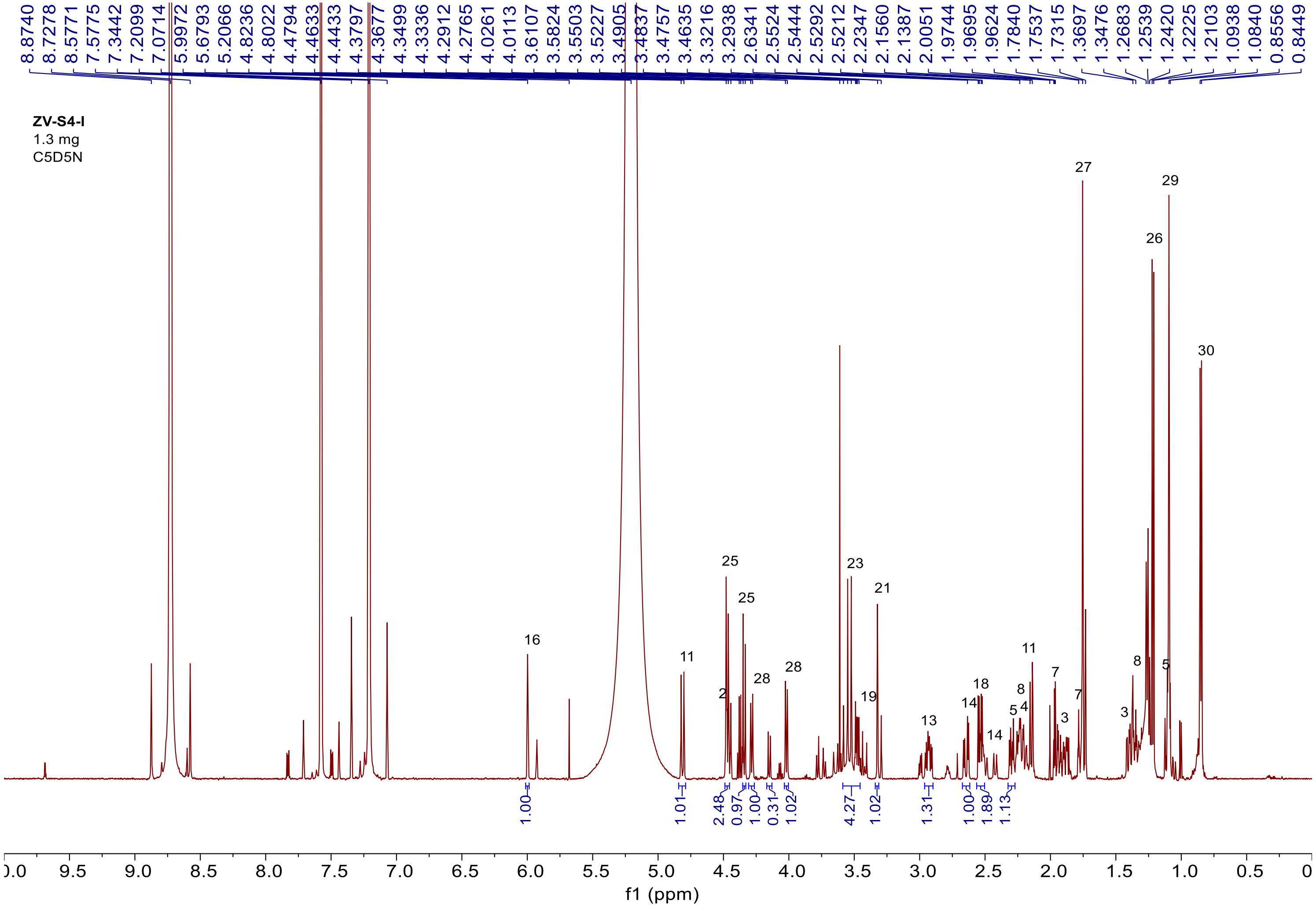

Figure S47. $^{13}C\{^{1}H\}$ NMR and DEPT spectra of **6** (C_5D_5N, 150 MHz)

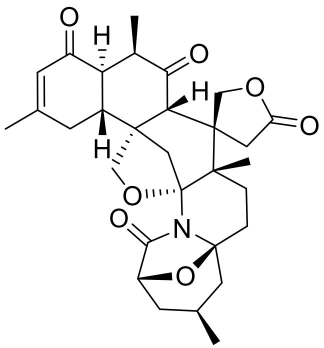

6

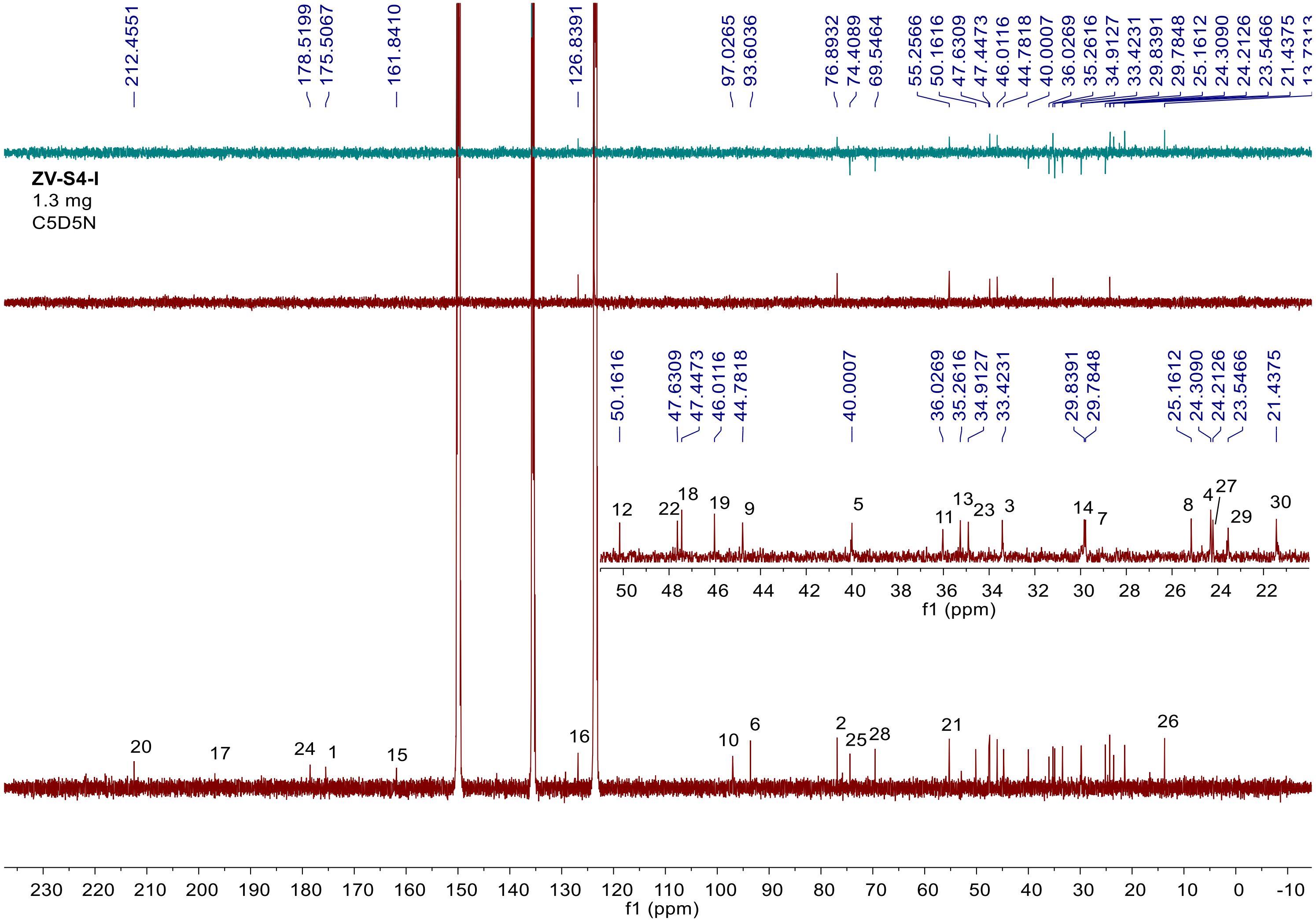

Figure S48. COSY spectrum of **6**

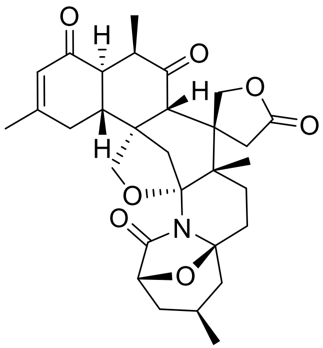

6

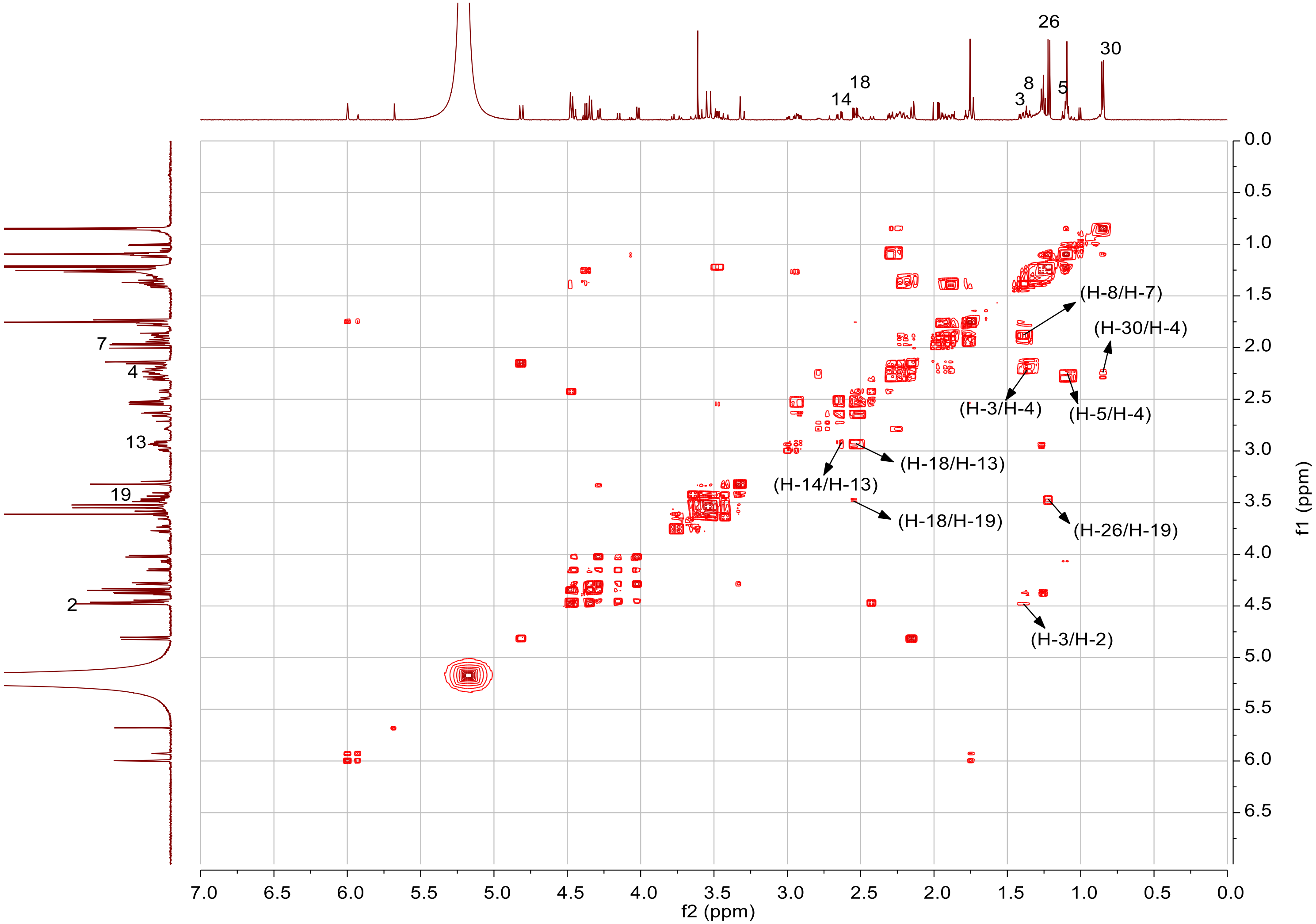

Figure S49. HSQC spectrum of **6**

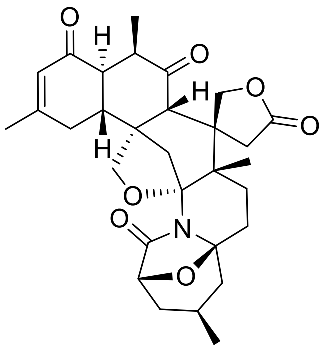

6

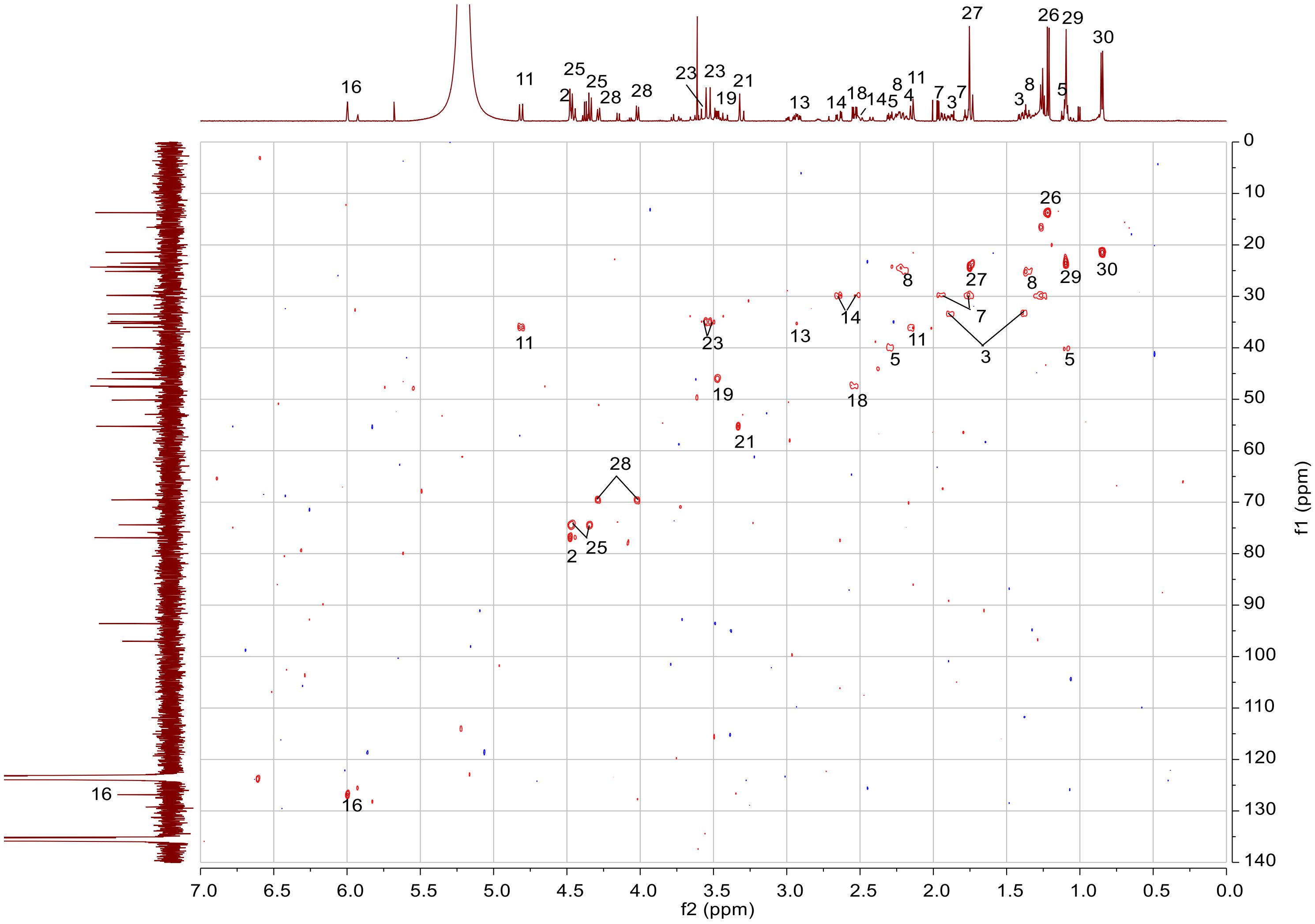

Figure S50. HMBC spectrum of **6**

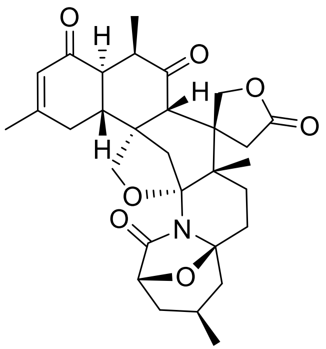

6

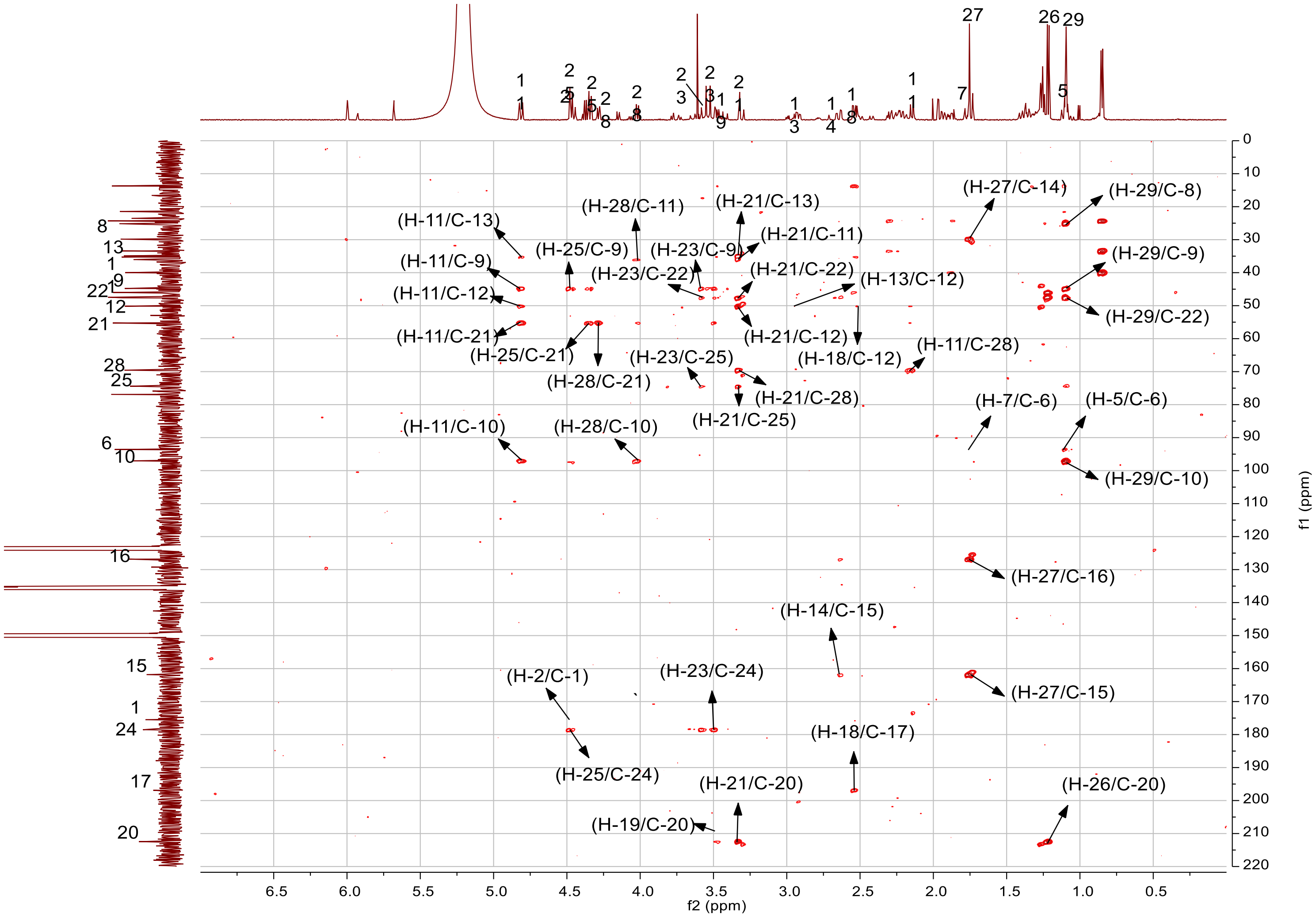

Figure S51. NOESY spectrum of **6**

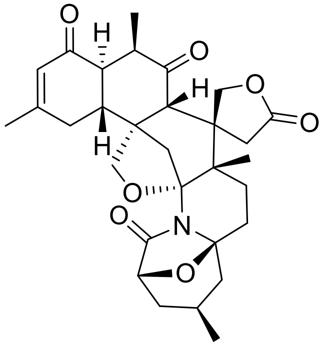

6

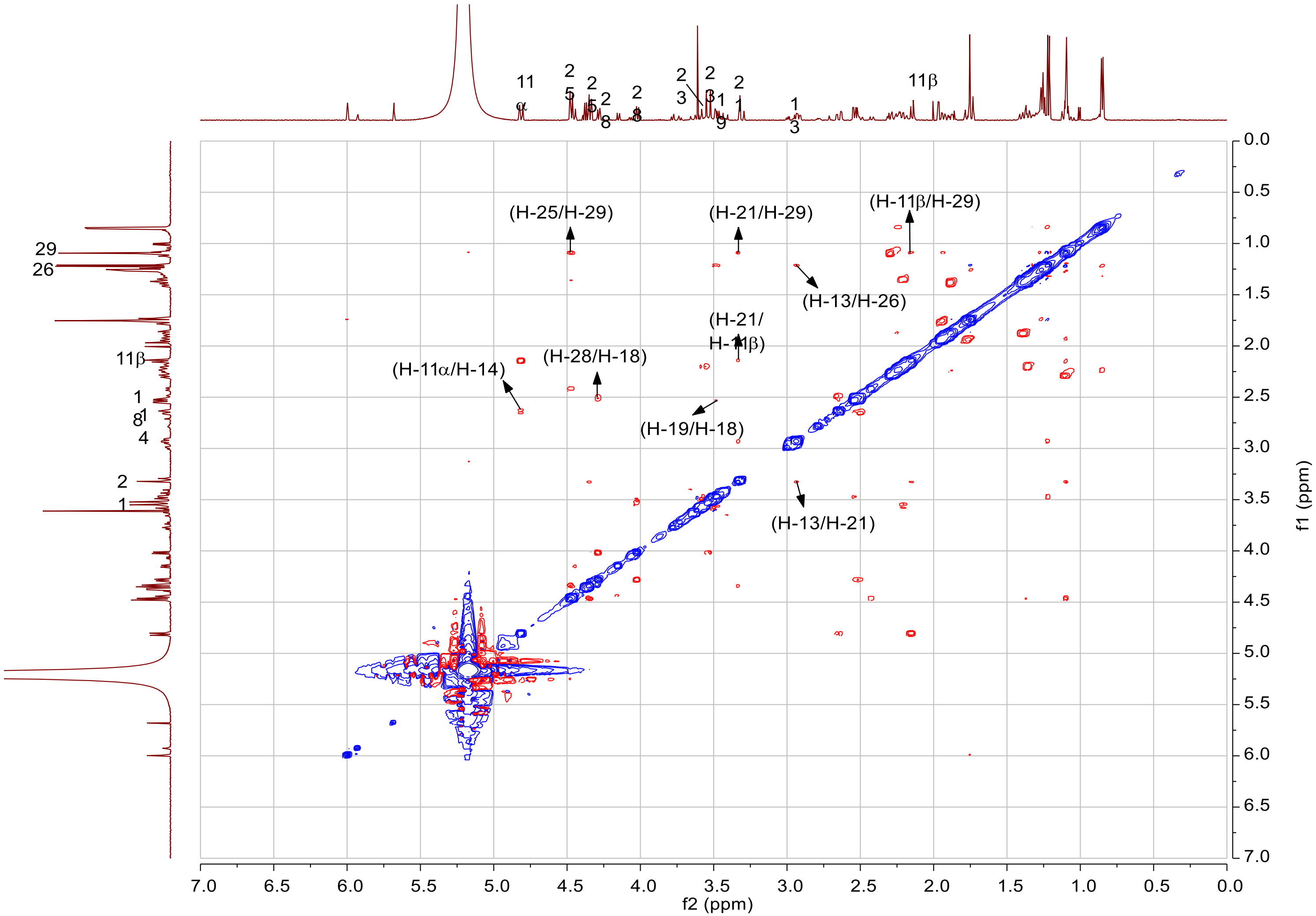

Figure S52. HRESIMS spectrum of **6**

Mass Spectrum SmartFormula Report

Analysis Info

Analysis Name	D:\QTOF\ZVS4IH2.d	2/24/2022 3:00:55 PM
Method	_tune_low_pos(50-1000)_20170502_Liao lab.m	Operator: YU HSIAO-CHING
Sample Name	ZV-S4-I	Instrument: BRUKER micrOTOF-Q
Comment	ESI Positive	

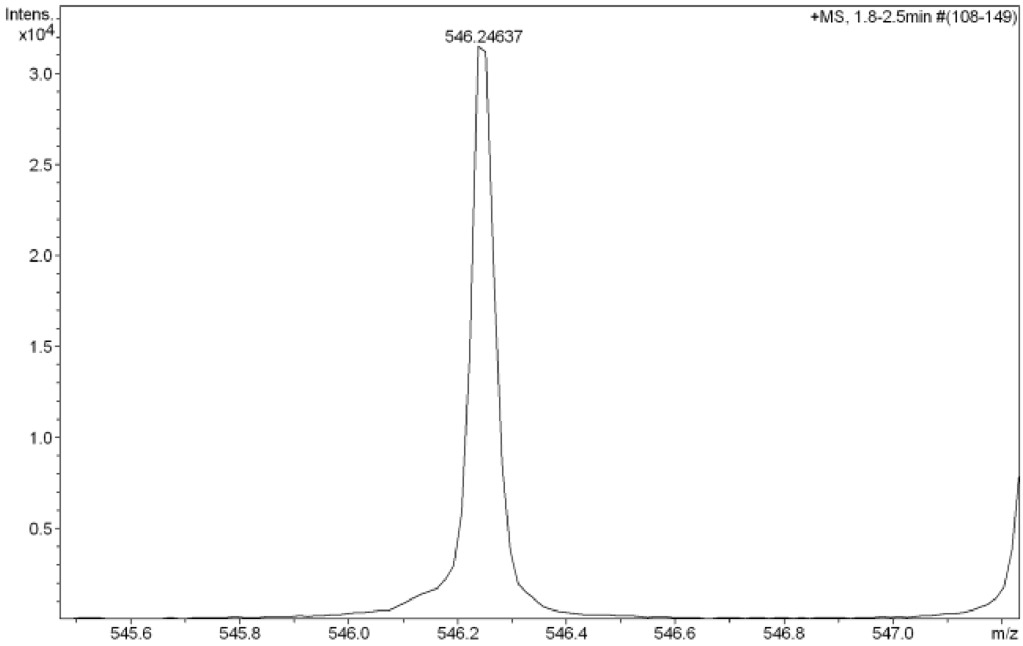

Meas. m/z	#	Formula	Score	m/z	err [mDa]	err [ppm]	mSigma	rdb	e⁻ Conf	N-Rule
546.24637	1	C 30 H 37 N Na O 7	100.00	546.24622	-0.14	-0.26	18.6	12.5	even	ok

Figure S53. UV spectrum of **6**

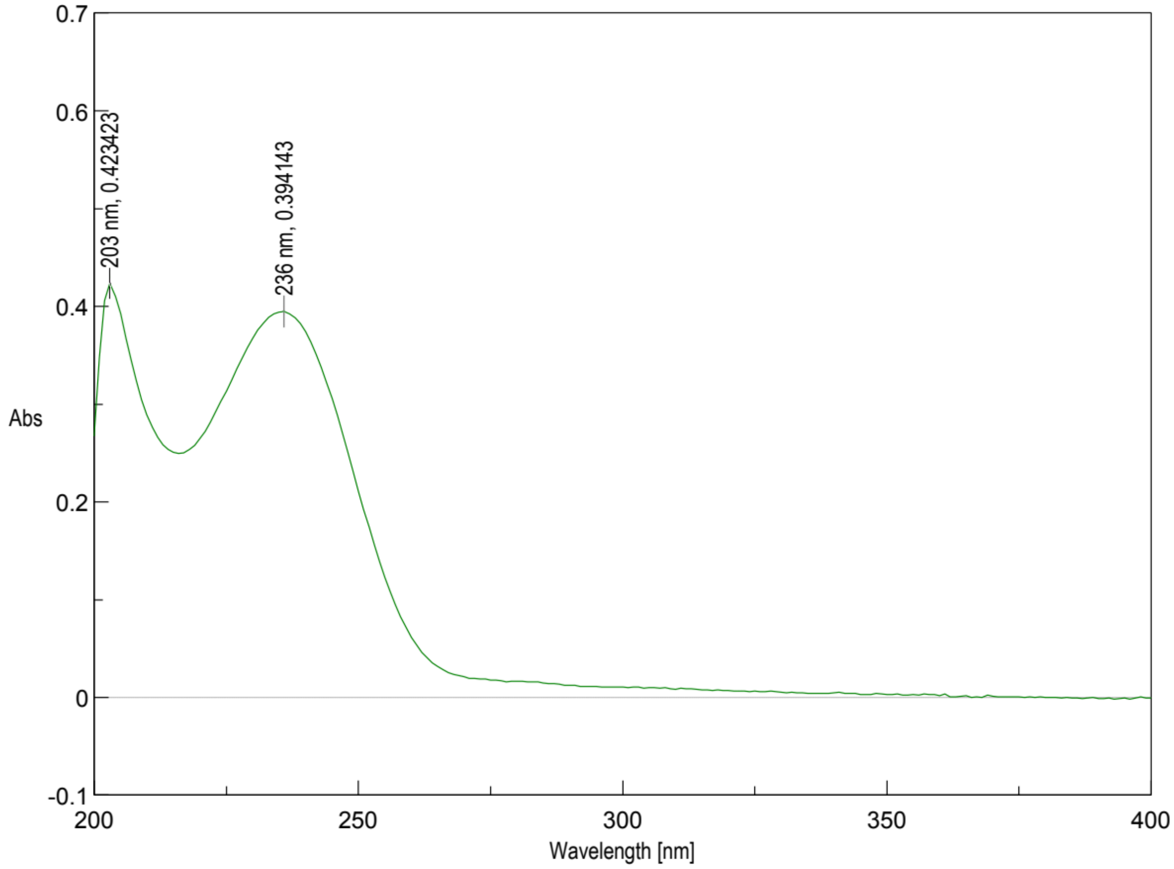

Figure S54. IR spectrum of **6**

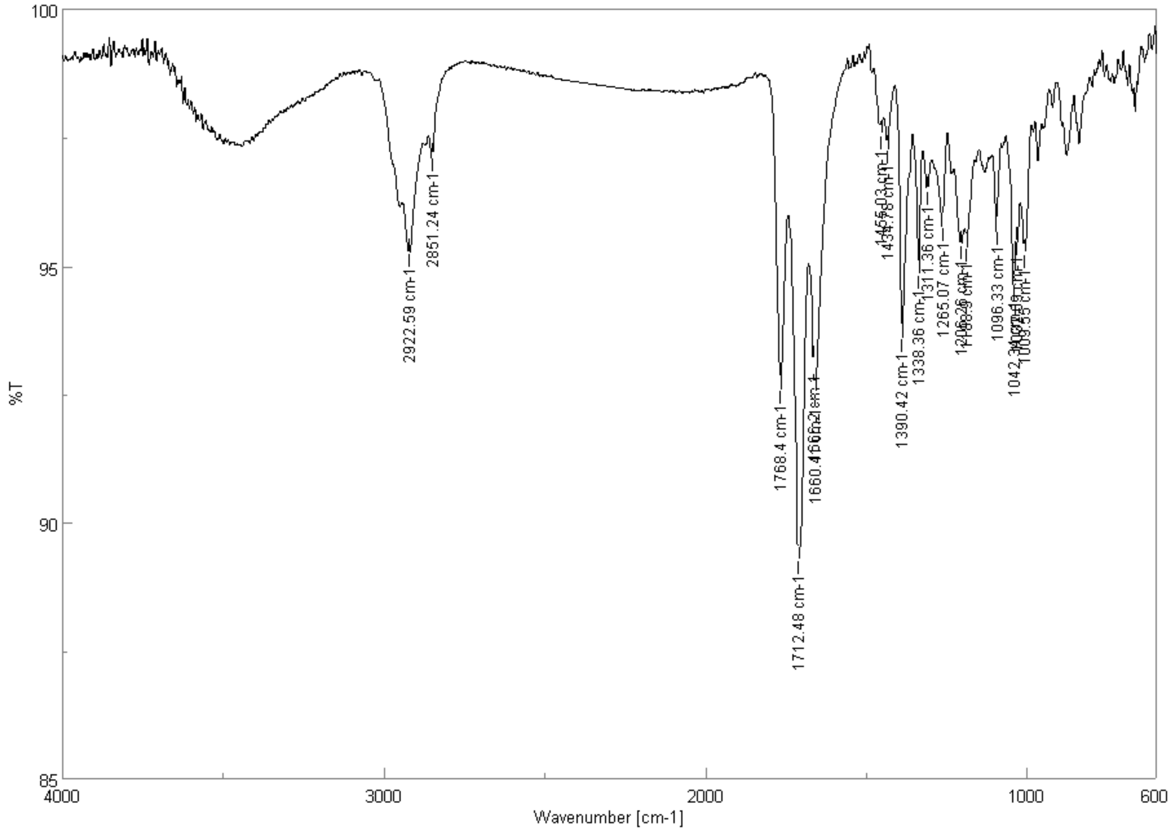

Figure S55. ^{1}H NMR spectrum of **7** (C_5D_5N, 600 MHz)

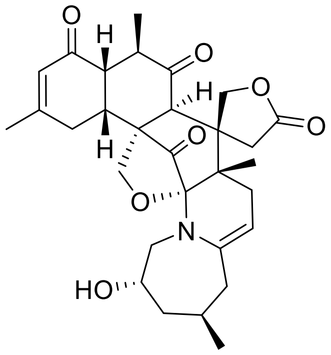

7

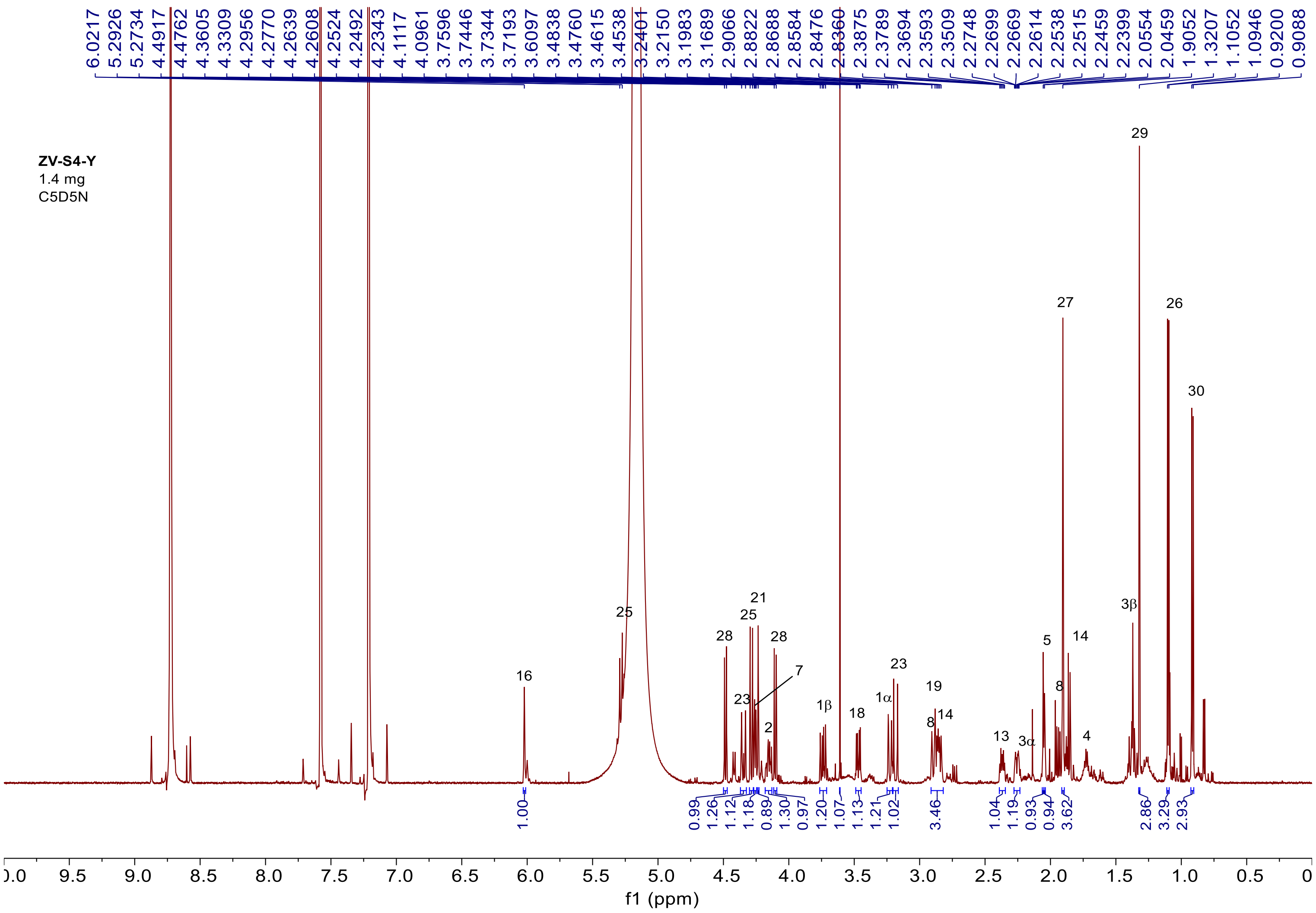

Figure S56. $^{13}C\{^{1}H\}$ NMR and DEPT spectra of **7** (C_5D_5N, 150 MHz)

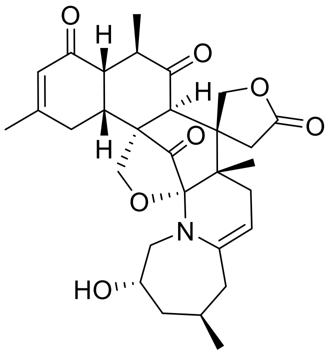

7

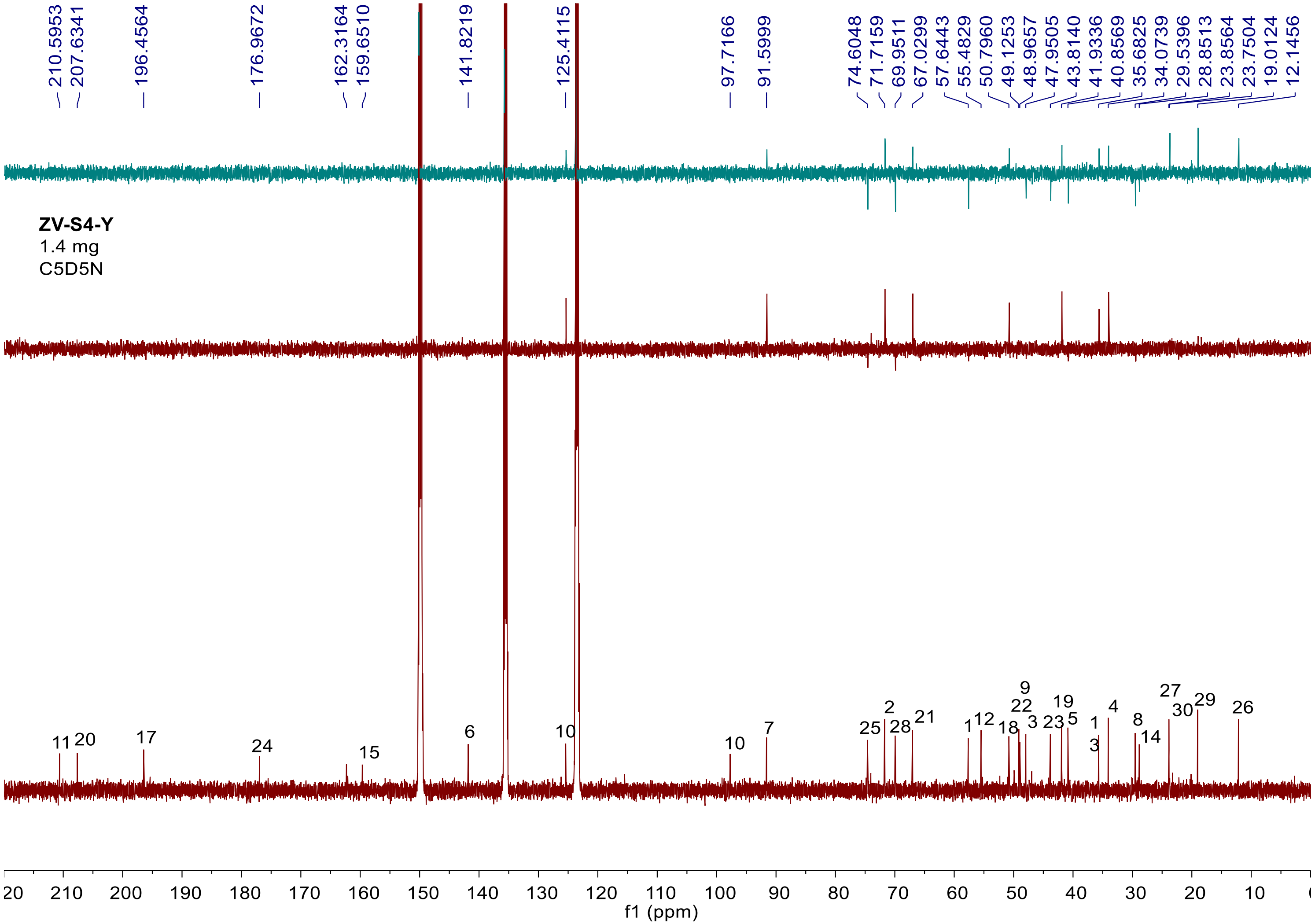

Figure S57. COSY spectrum of **7**

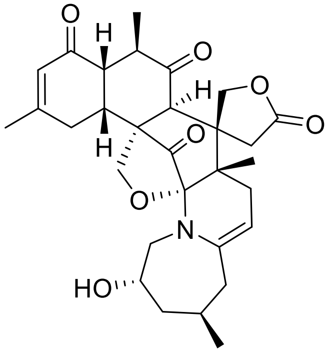

7

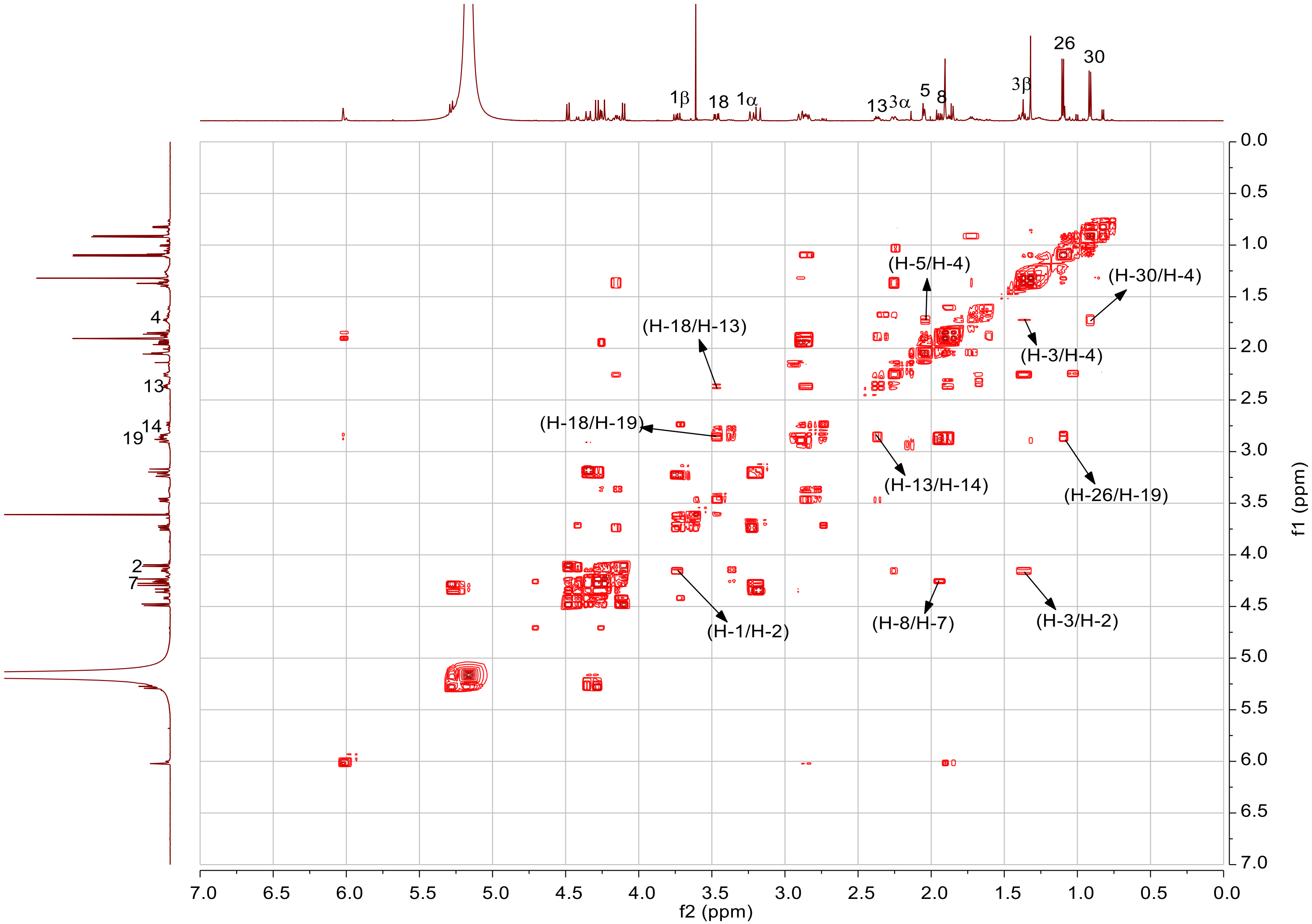

Figure S58. HSQC spectrum of **7**

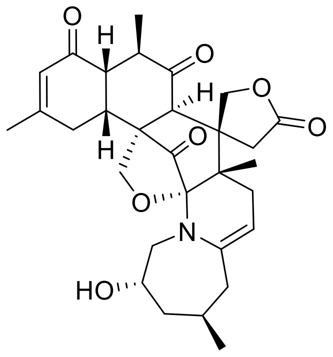

7

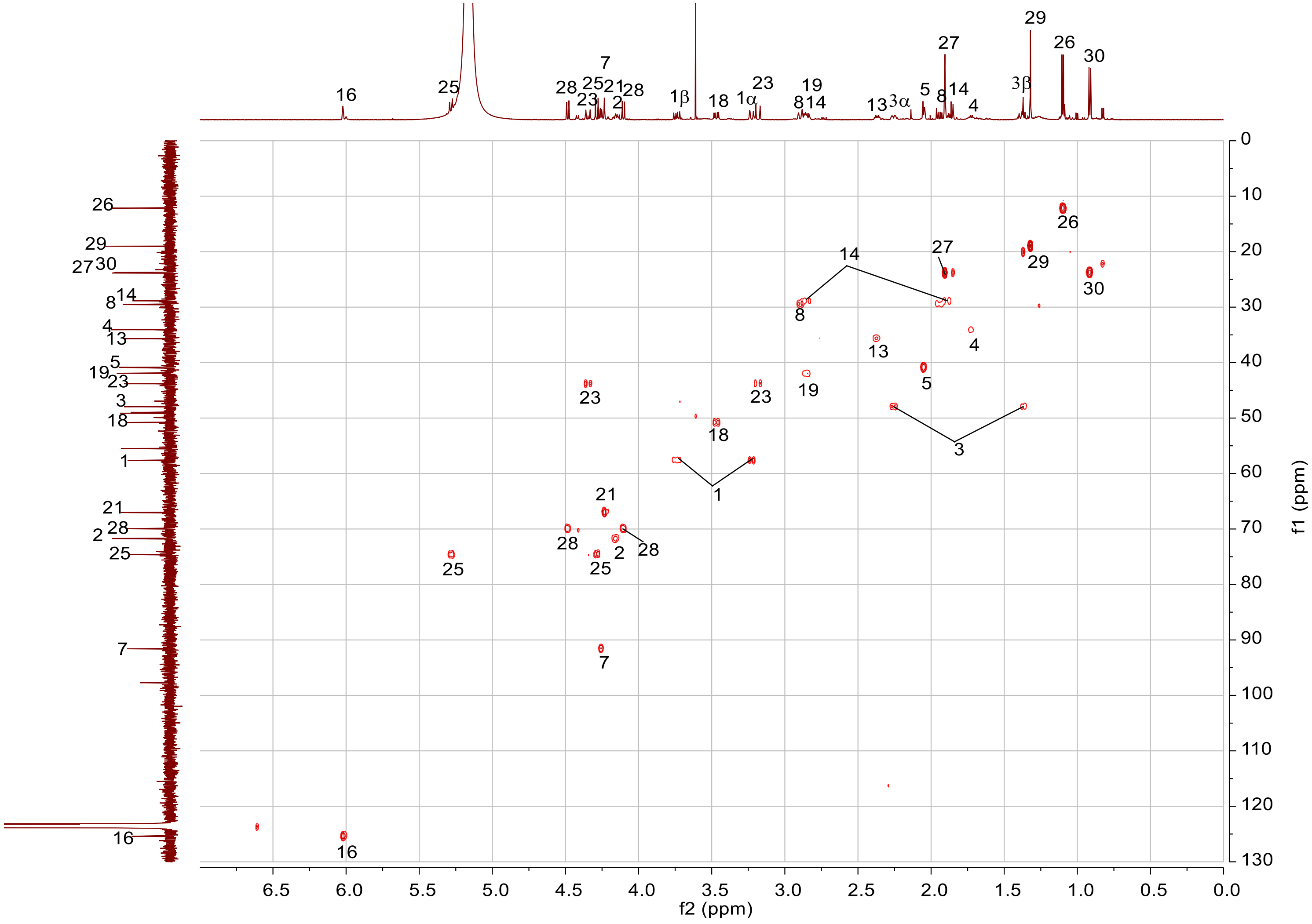

Figure S59. HMBC spectrum of **7**

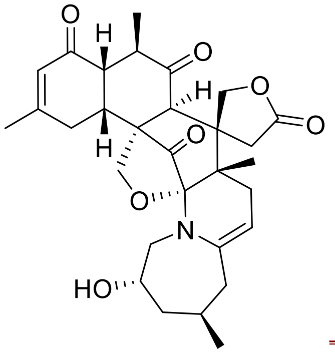

7

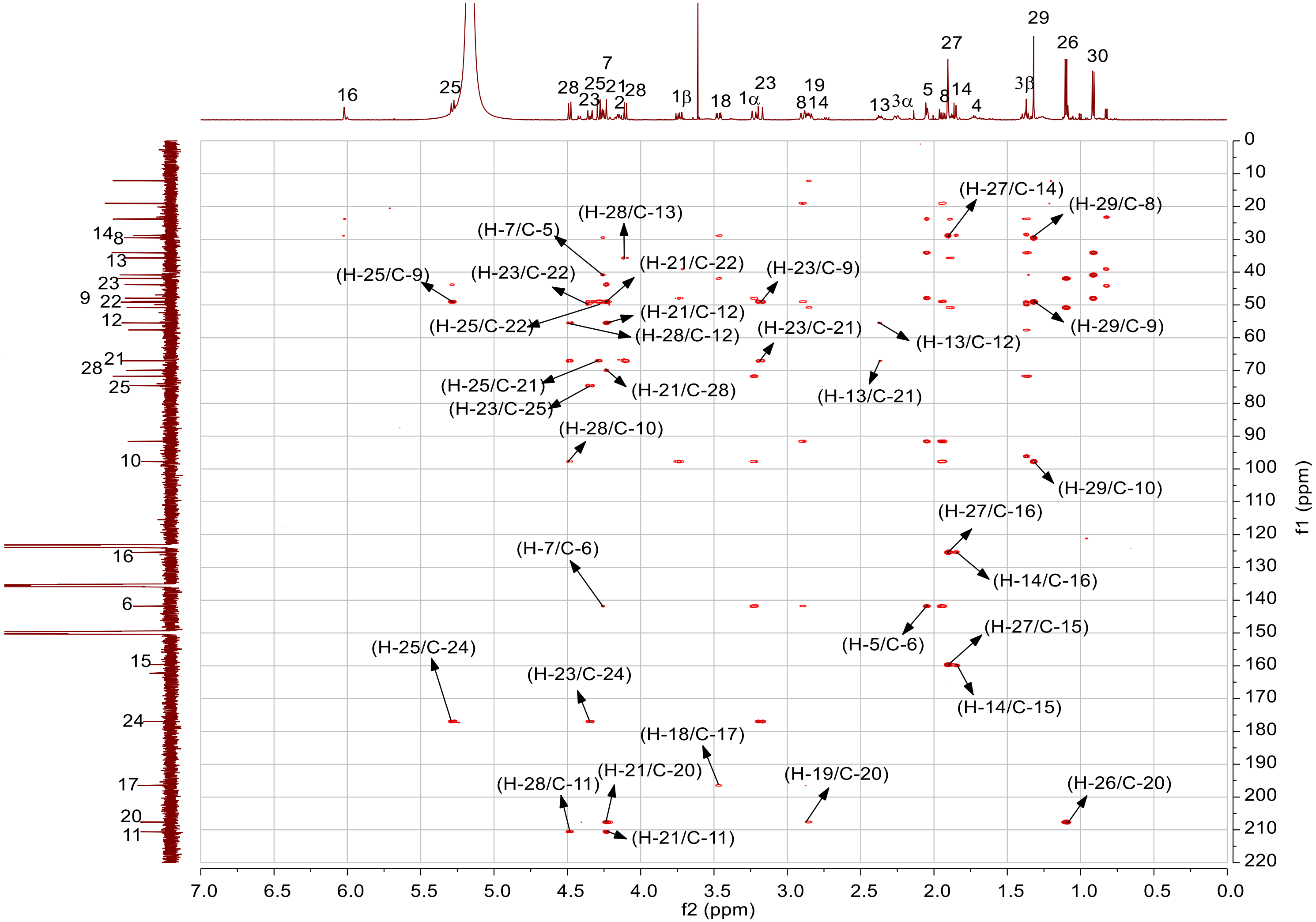

Figure S60. NOESY spectrum of **7**

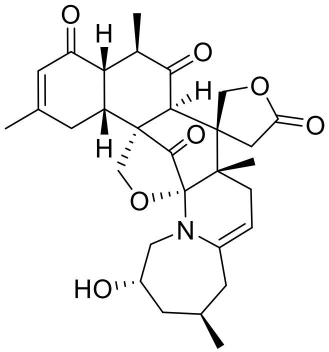

7

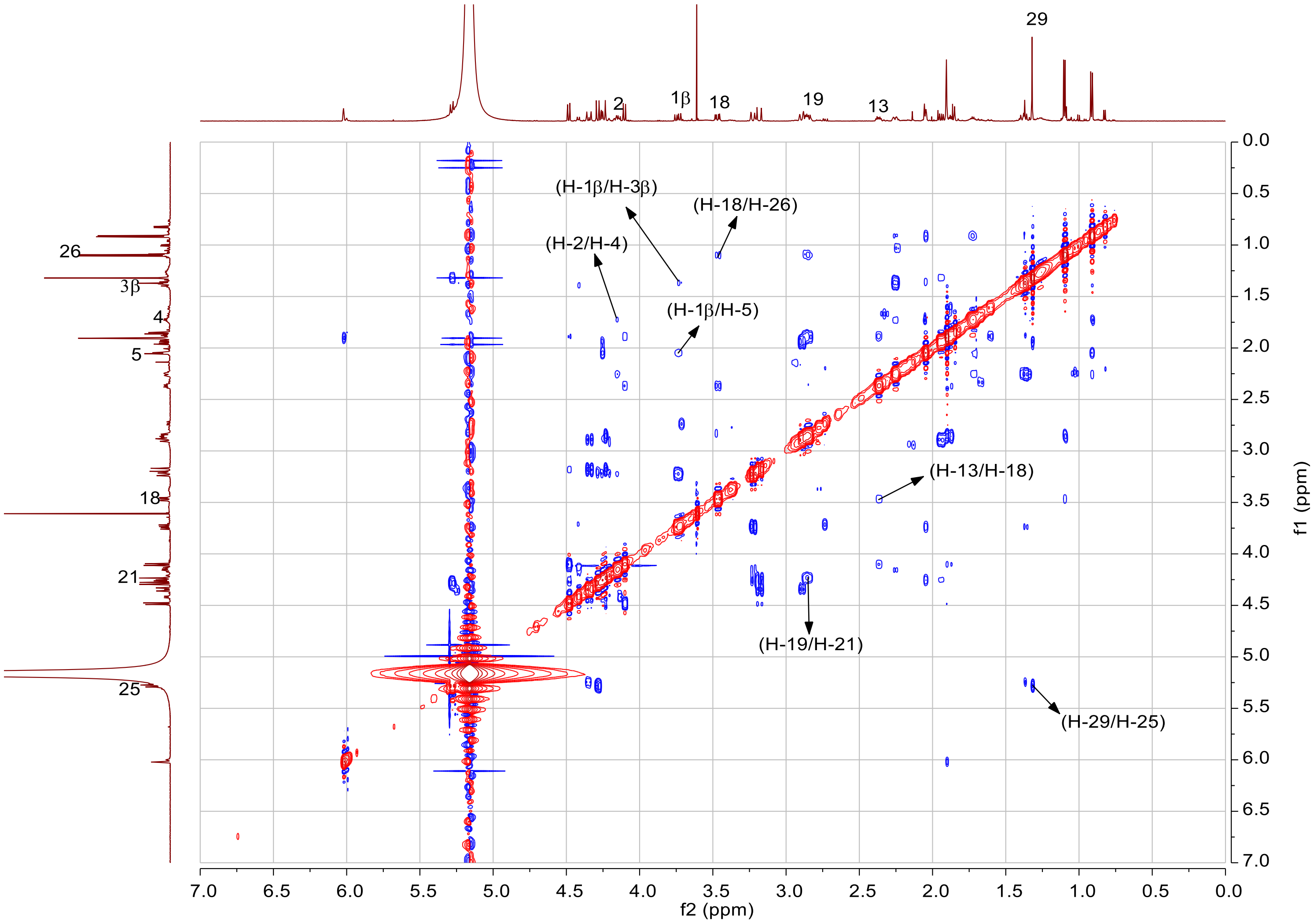

Figure S61. HRESIMS spectrum of **7**

Mass Spectrum SmartFormula Report

Analysis Info

Analysis Name	D:\QTOF\ZVS4Y.d	6/27/2022 11:35:54 AM
Method	tune_wide_pos_20220422.m	Operator: YU HSIAO-CHING
Sample Name	ZVS4-Y	Instrument: BRUKER micrOTOF-Q
Comment	ESI Positive	

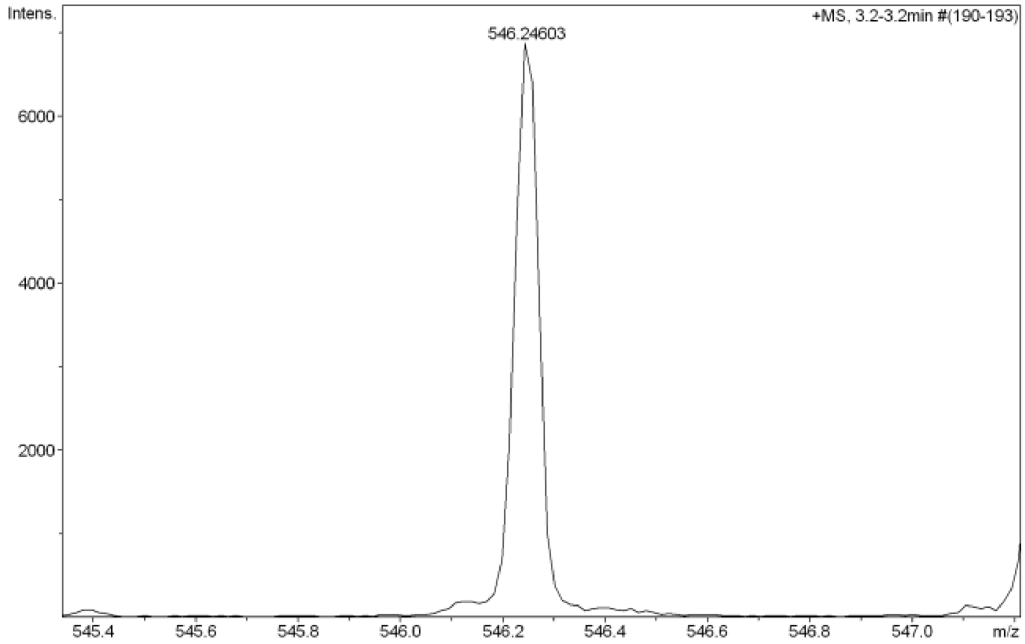

Meas. m/z	#	Formula	Score	m/z	err [mDa]	err [ppm]	mSigma	rdb	e⁻ Conf	N-Rule
546.24603	1	C 30 H 37 N Na O 7	100.00	546.24622	0.19	0.35	46.6	12.5	even	ok

Figure S62. UV spectrum of **7**

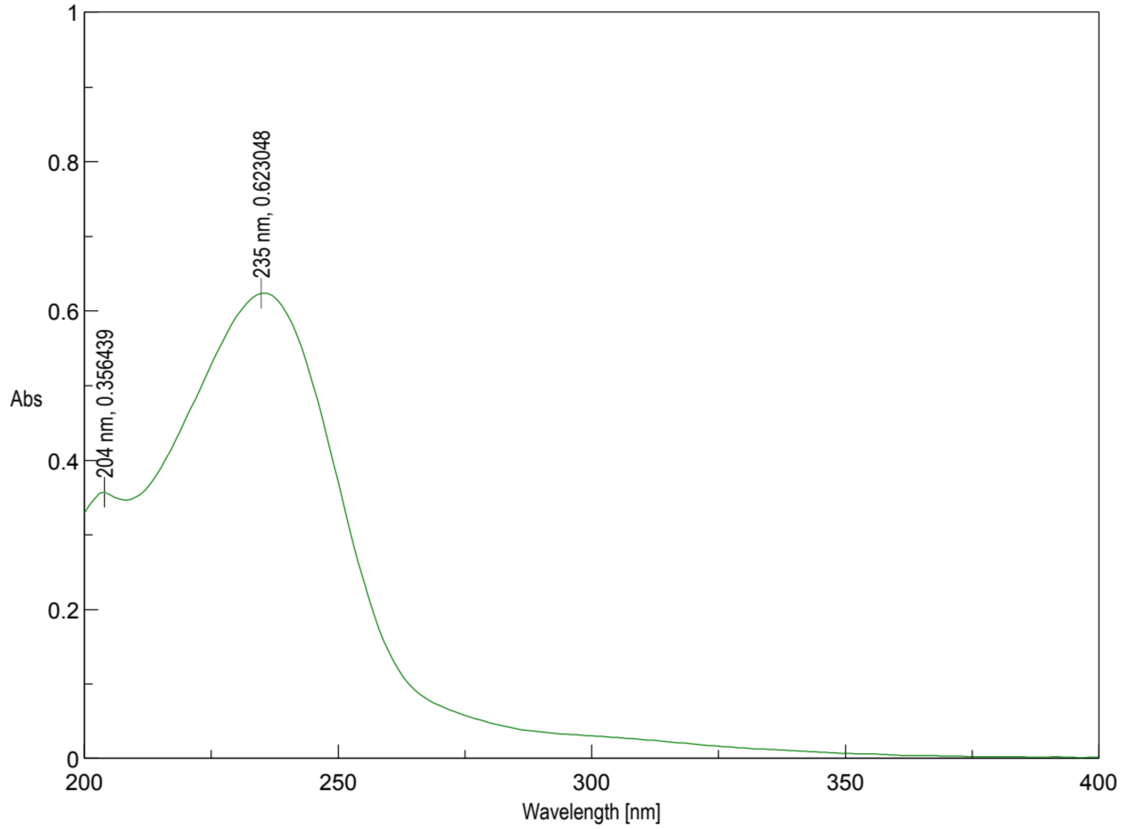

Figure S63. IR spectrum of **7**

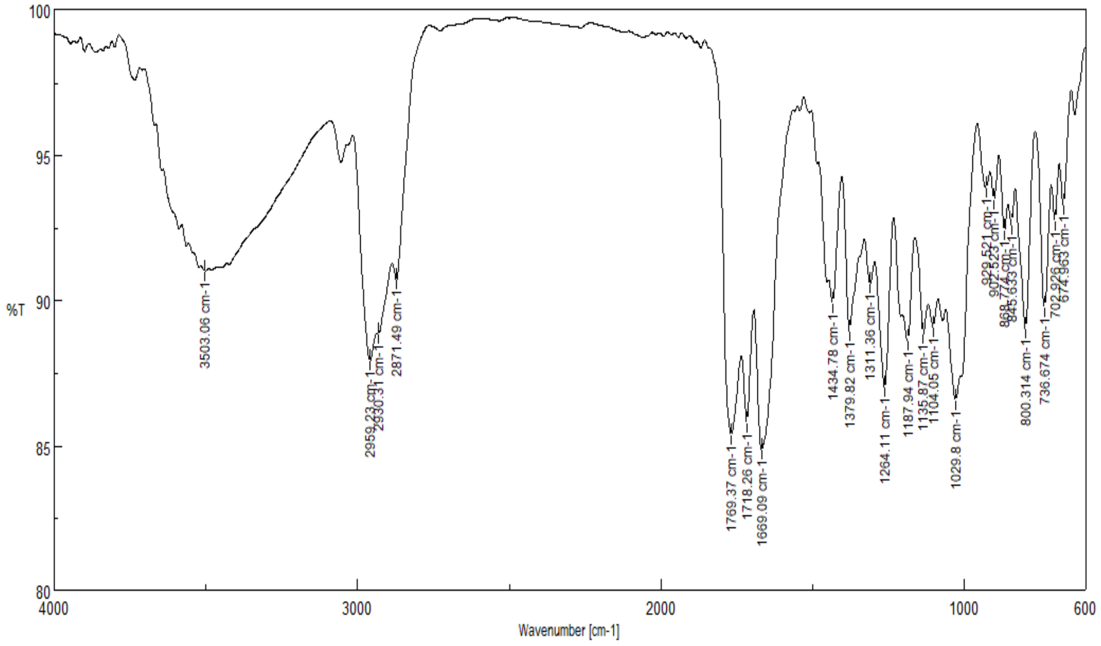

Figure S64. ^{1}H NMR spectrum of **8** (C_5D_5N, 400 MHz)

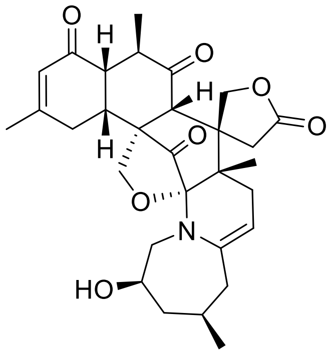

8

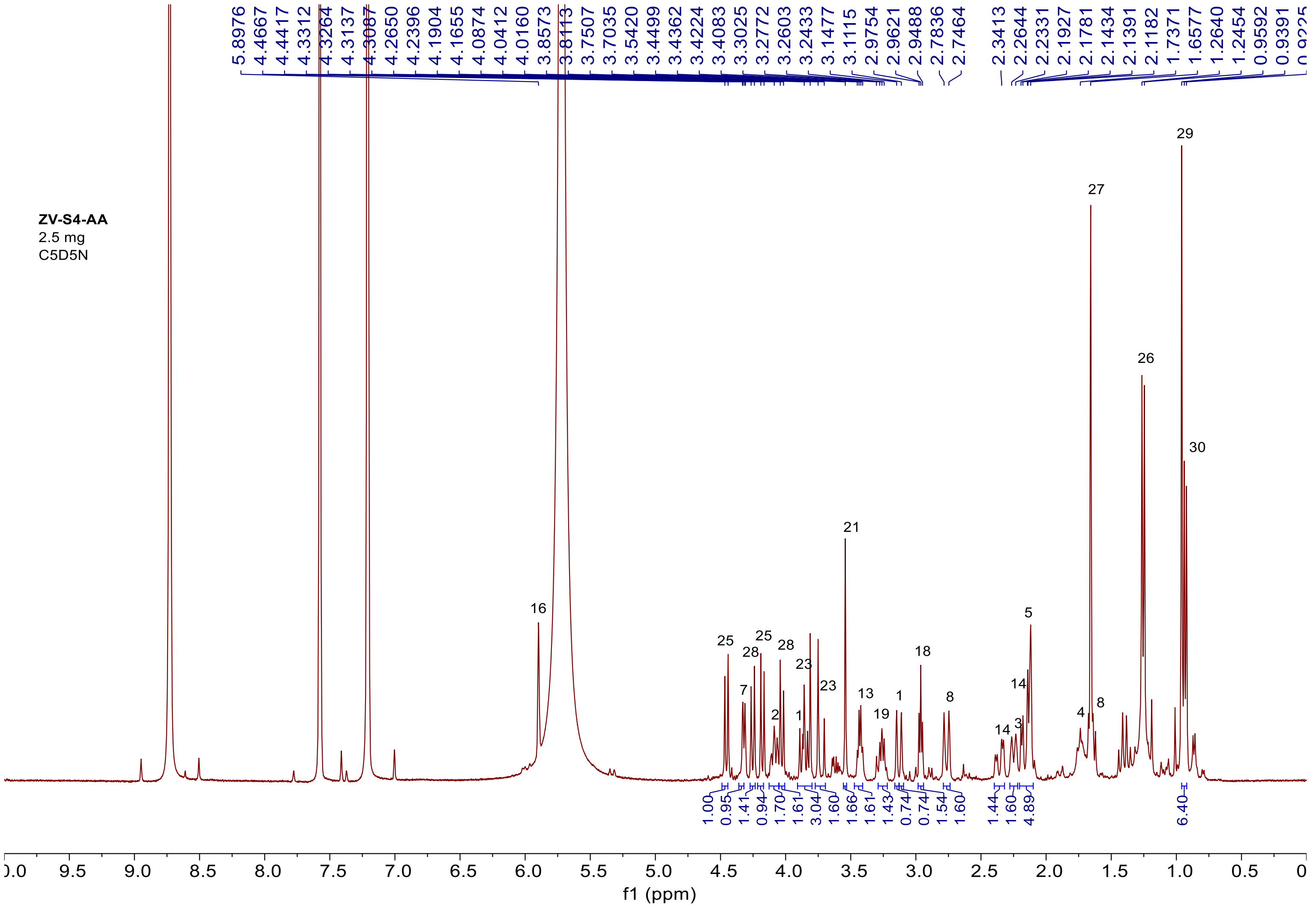

Figure S65. $^{13}C\{^{1}H\}$ NMR spectrum of **8** (C_5D_5N, 100 MHz)

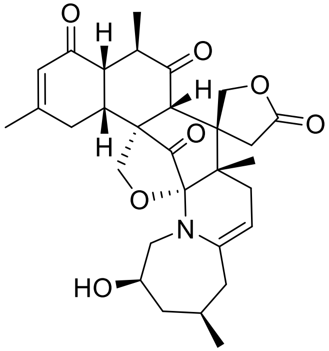

8

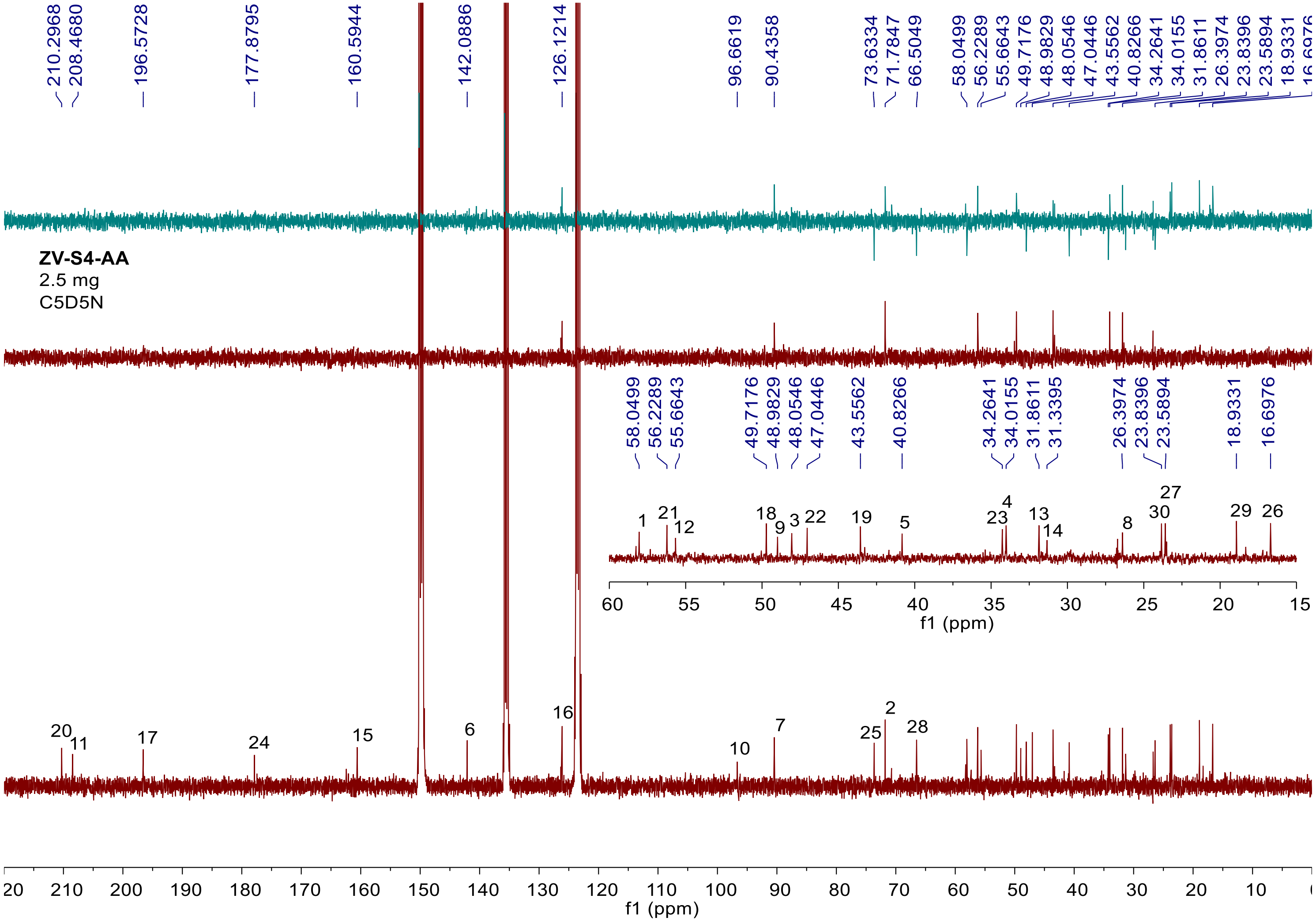

Figure S66. COSY spectrum of **8**

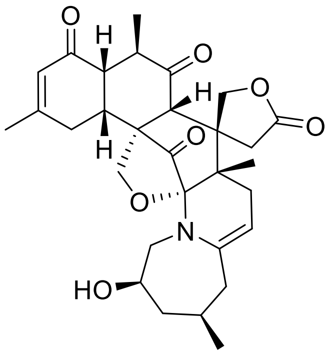

8

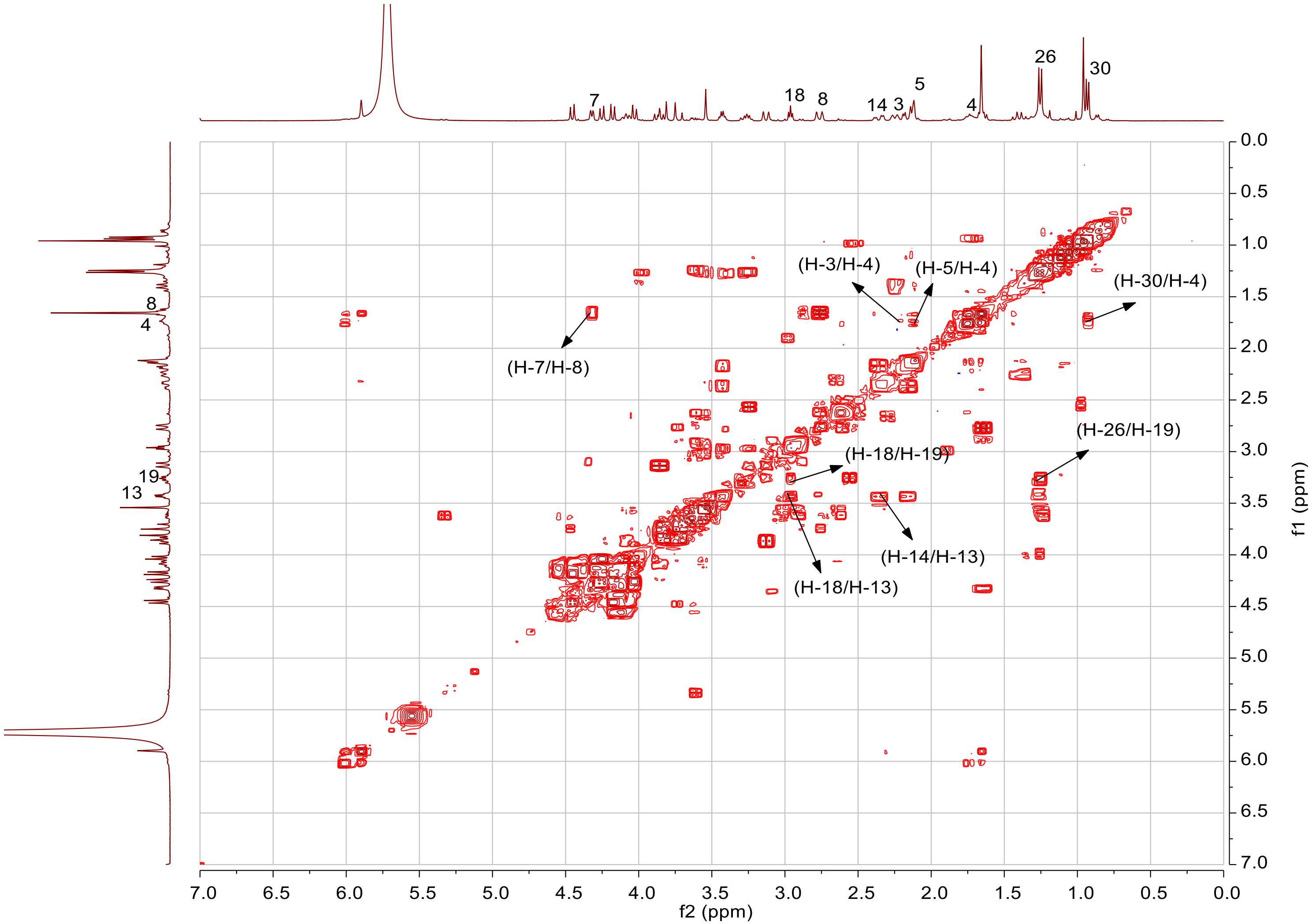

Figure S67. HSQC spectrum of **8**

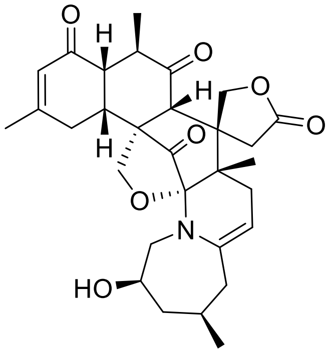

8

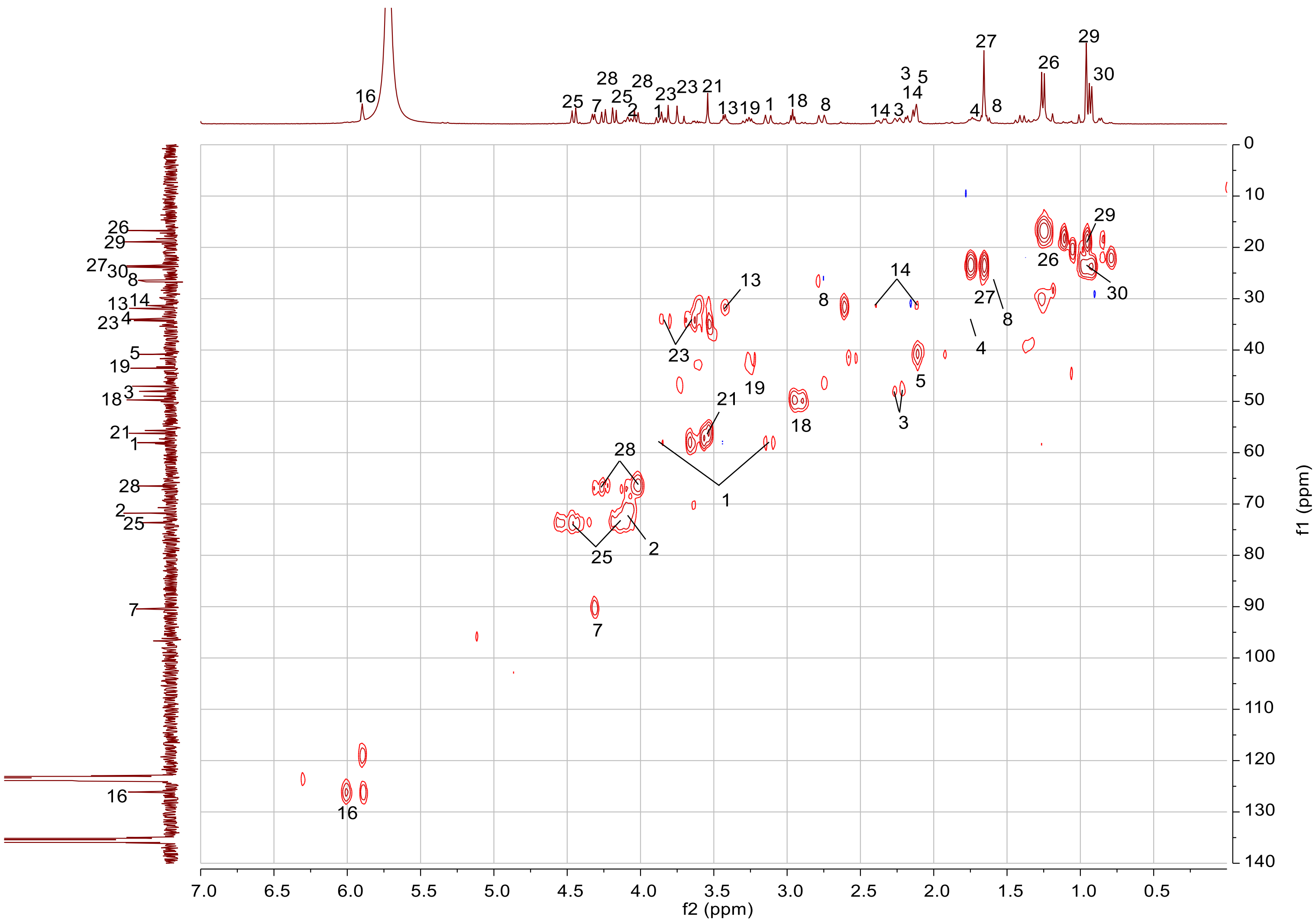

Figure S68. HMBC spectrum of **8**

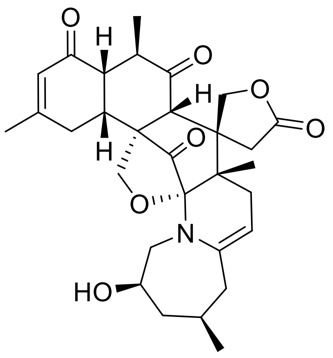

8

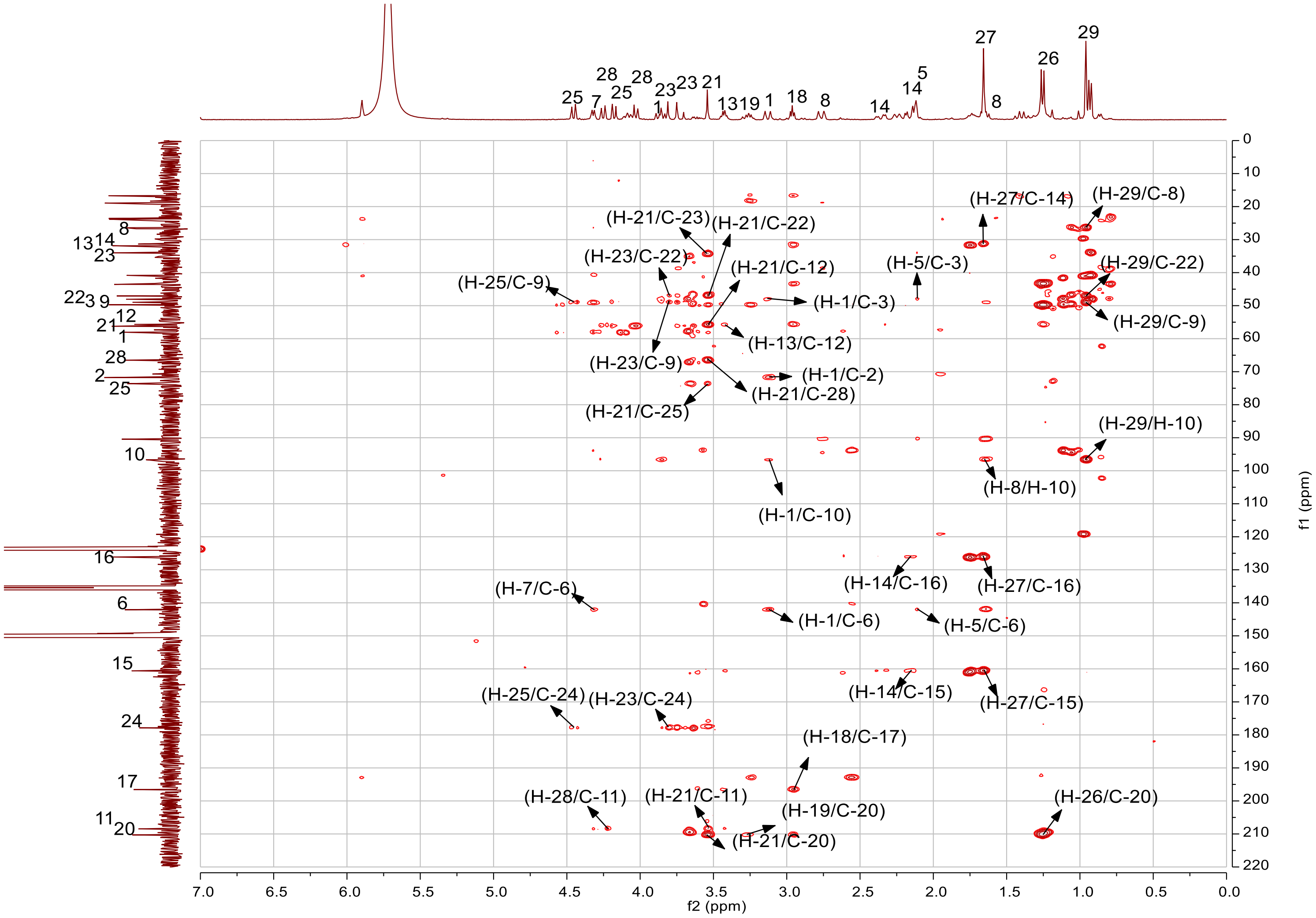

Figure S69. NOESY spectrum of **8**

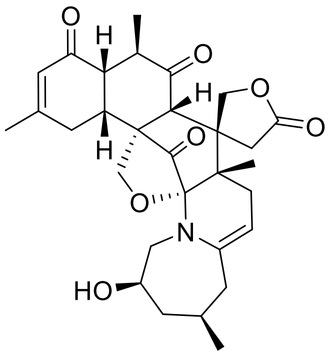

8

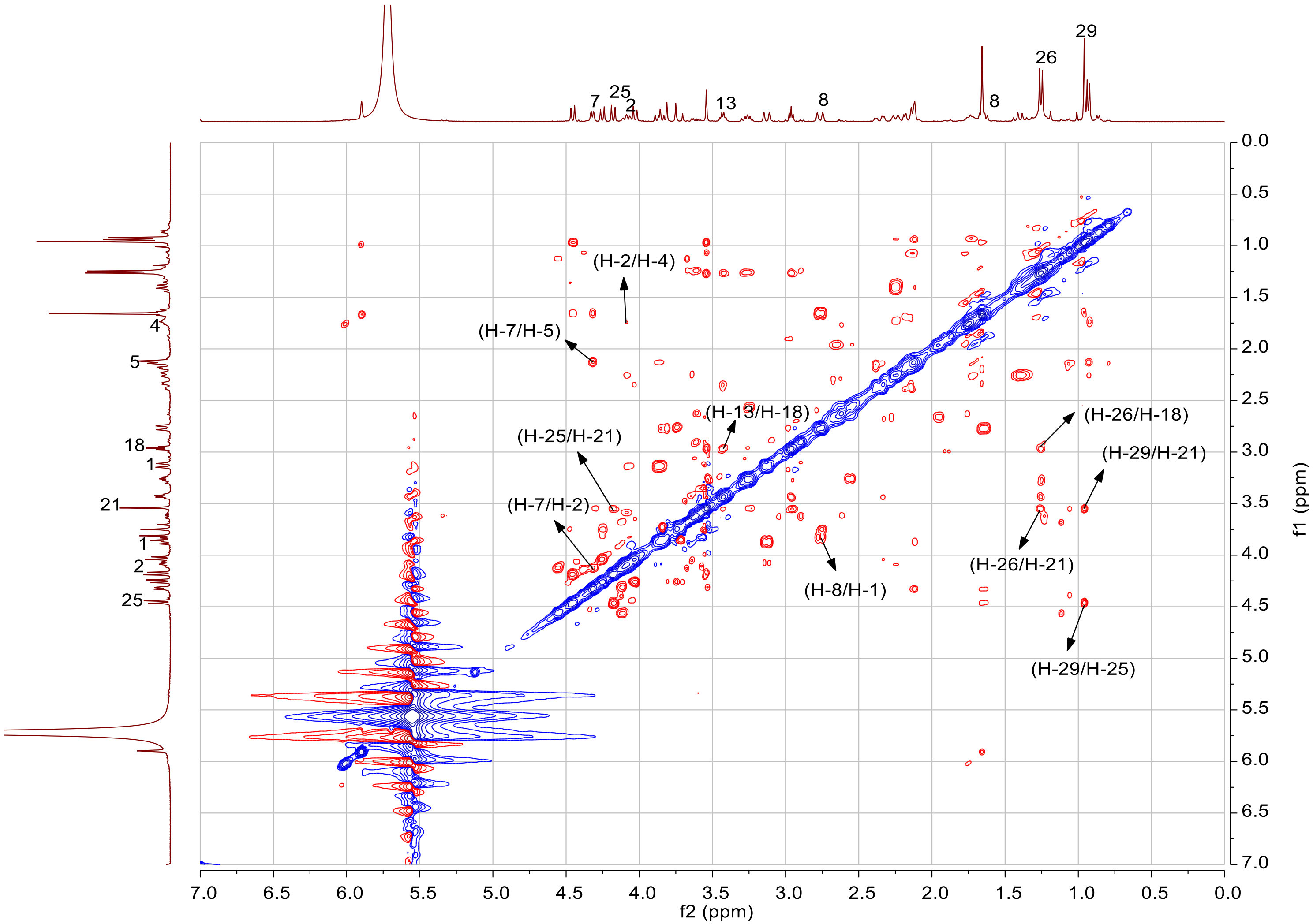

Figure S70. HRESIMS spectrum of **8**

Mass Spectrum SmartFormula Report

Analysis Info

Analysis Name	D:\0727\ZV-S4-AAR.d	7/28/2022 11:21:00 AM
Method	tune_wide_pos_20220422.m	Operator: YU HSIAO-CHING
Sample Name	ZV-S4-AA	Instrument: BRUKER micrOTOF-Q
Comment	ESI Positive	

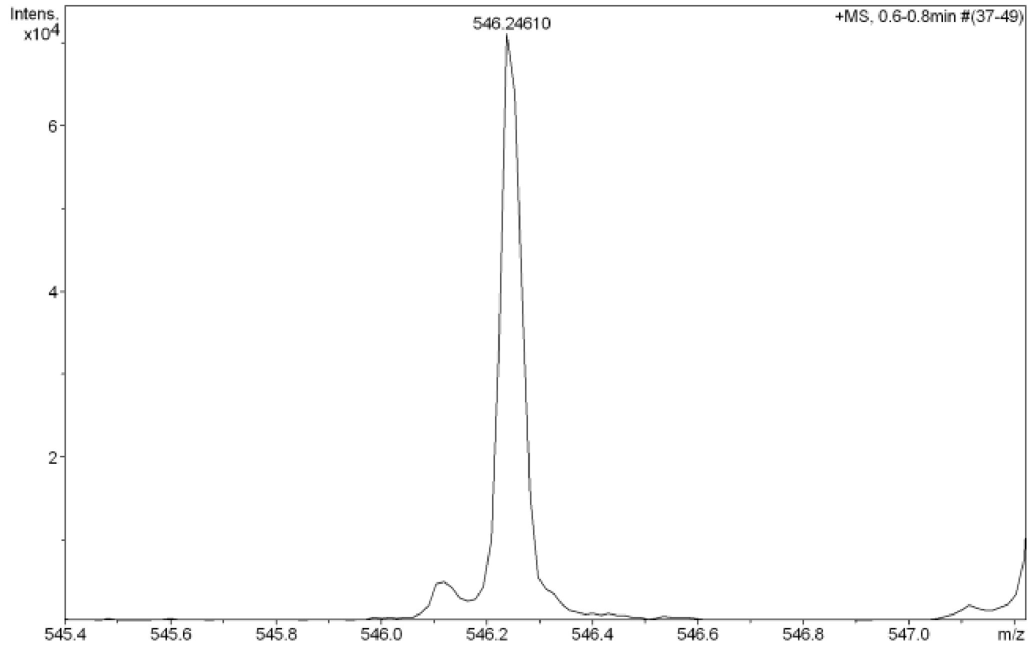

Meas. m/z	#	Formula	Score	m/z	err [mDa]	err [ppm]	mSigma	rdb	e¯ Conf	N-Rule
546.24610	1	C 30 H 37 N Na O 7	100.00	546.24622	0.13	0.23	30.6	12.5	even	ok

Figure S71. UV spectrum of **8**

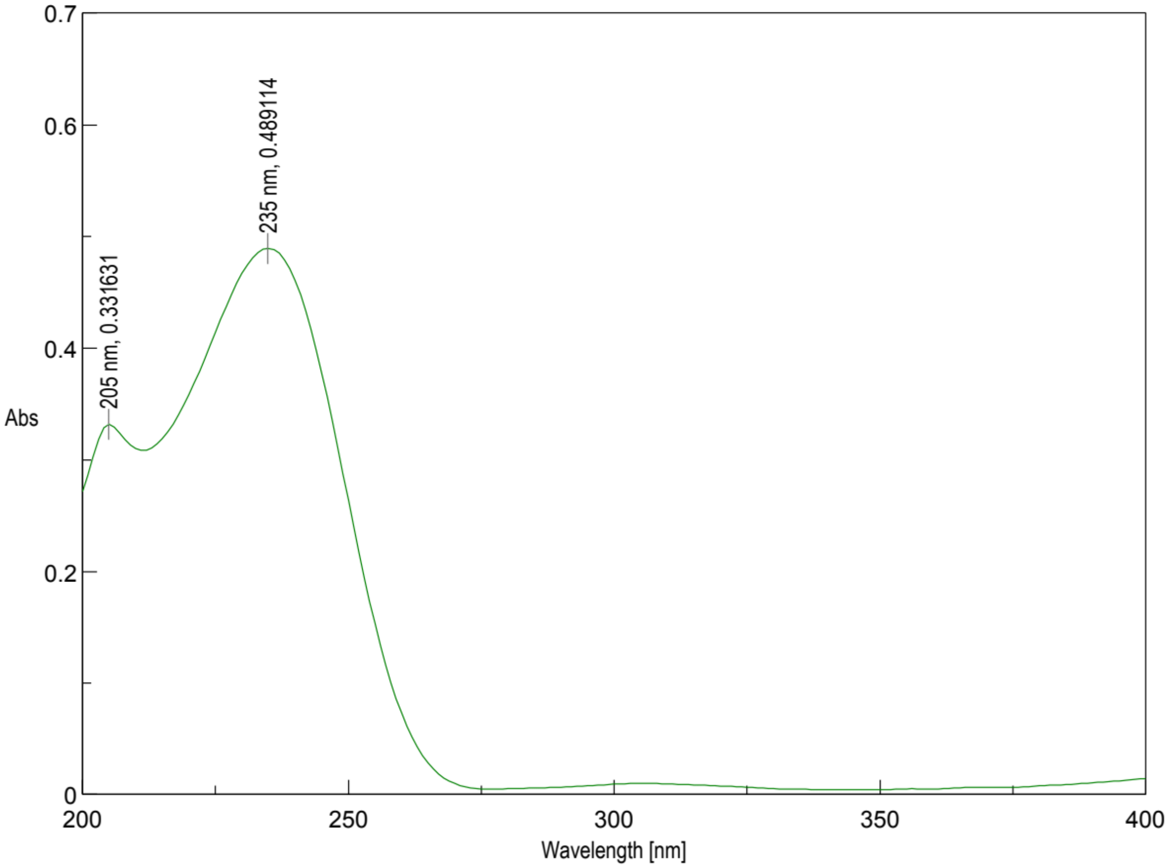

Figure S72. IR spectrum of **8**

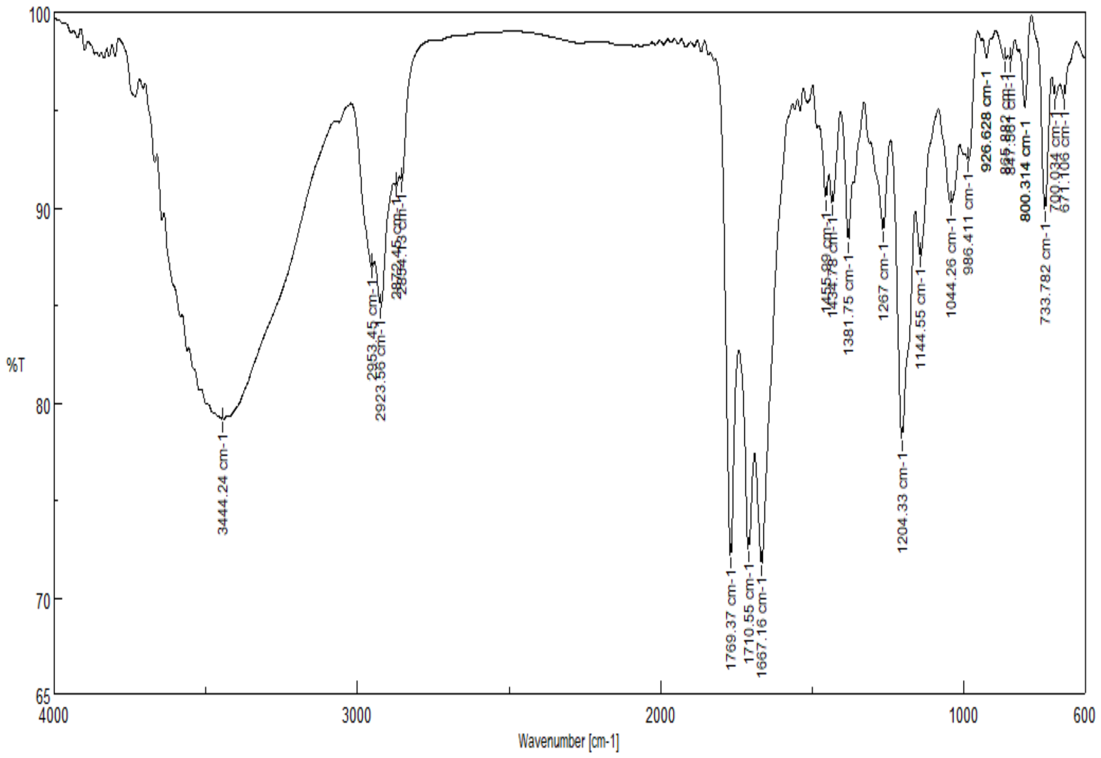

Figure S73. ^{1}H NMR spectrum of **9** (C_5D_5N, 600 MHz)

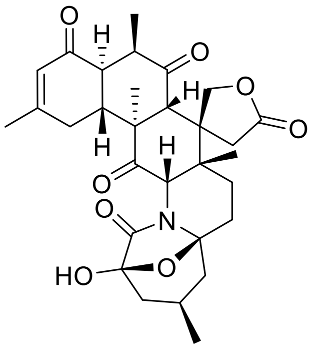

9

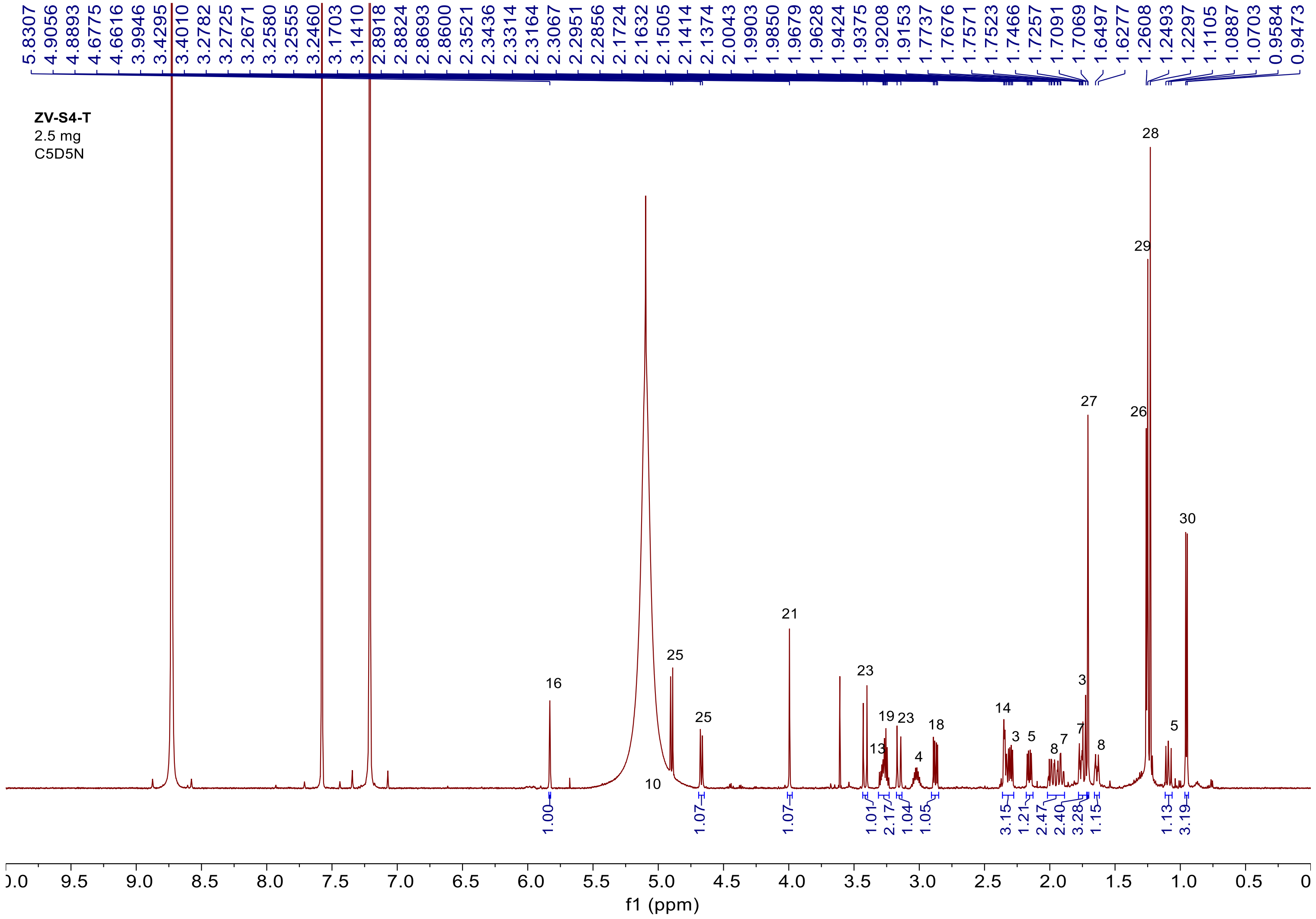

Figure S74. $^{13}C\{^{1}H\}$ NMR and DEPT spectra of **9** (C_5D_5N, 150 MHz)

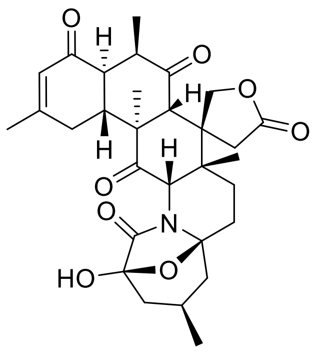

9

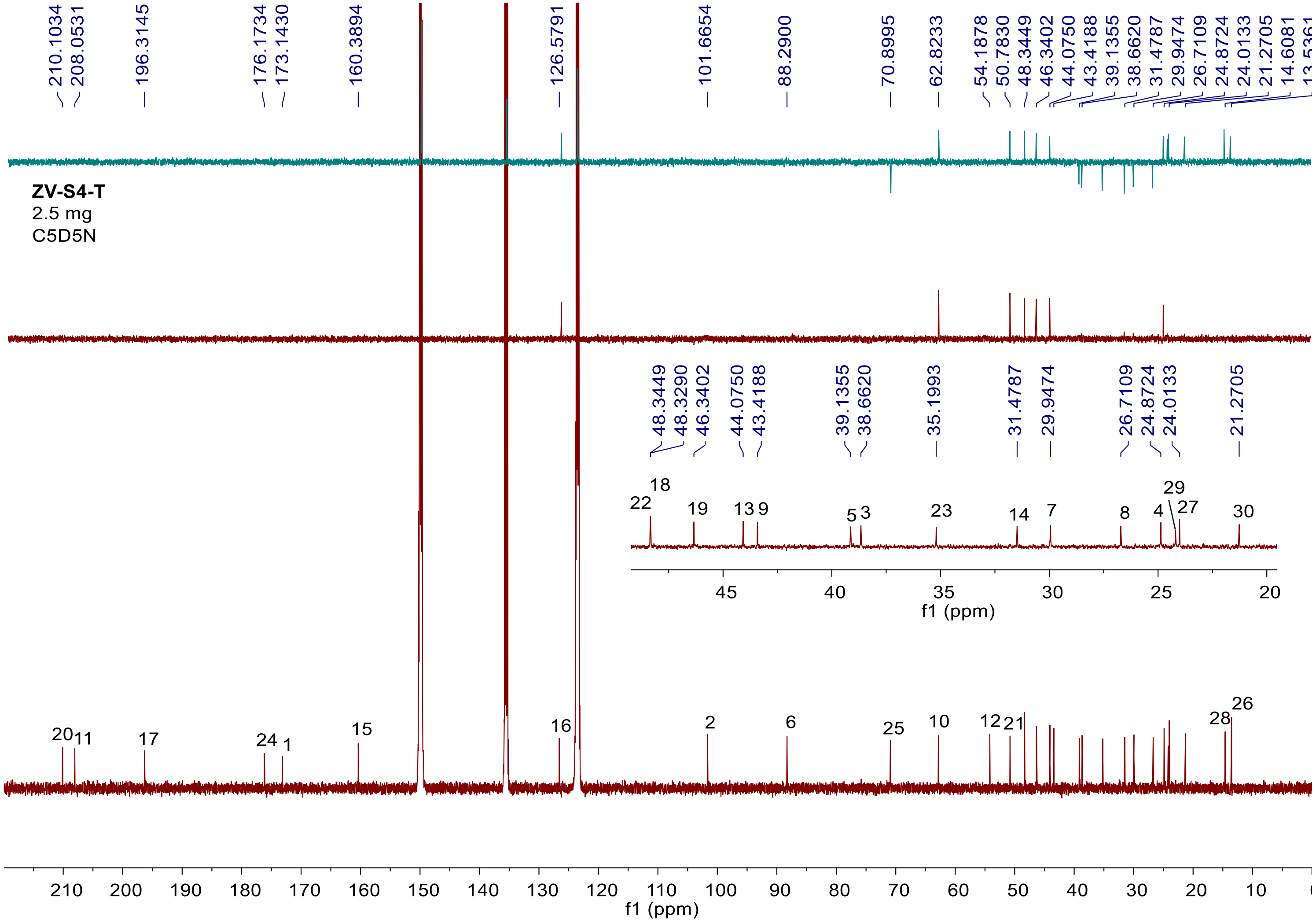

Figure S75. COSY spectrum of **9**

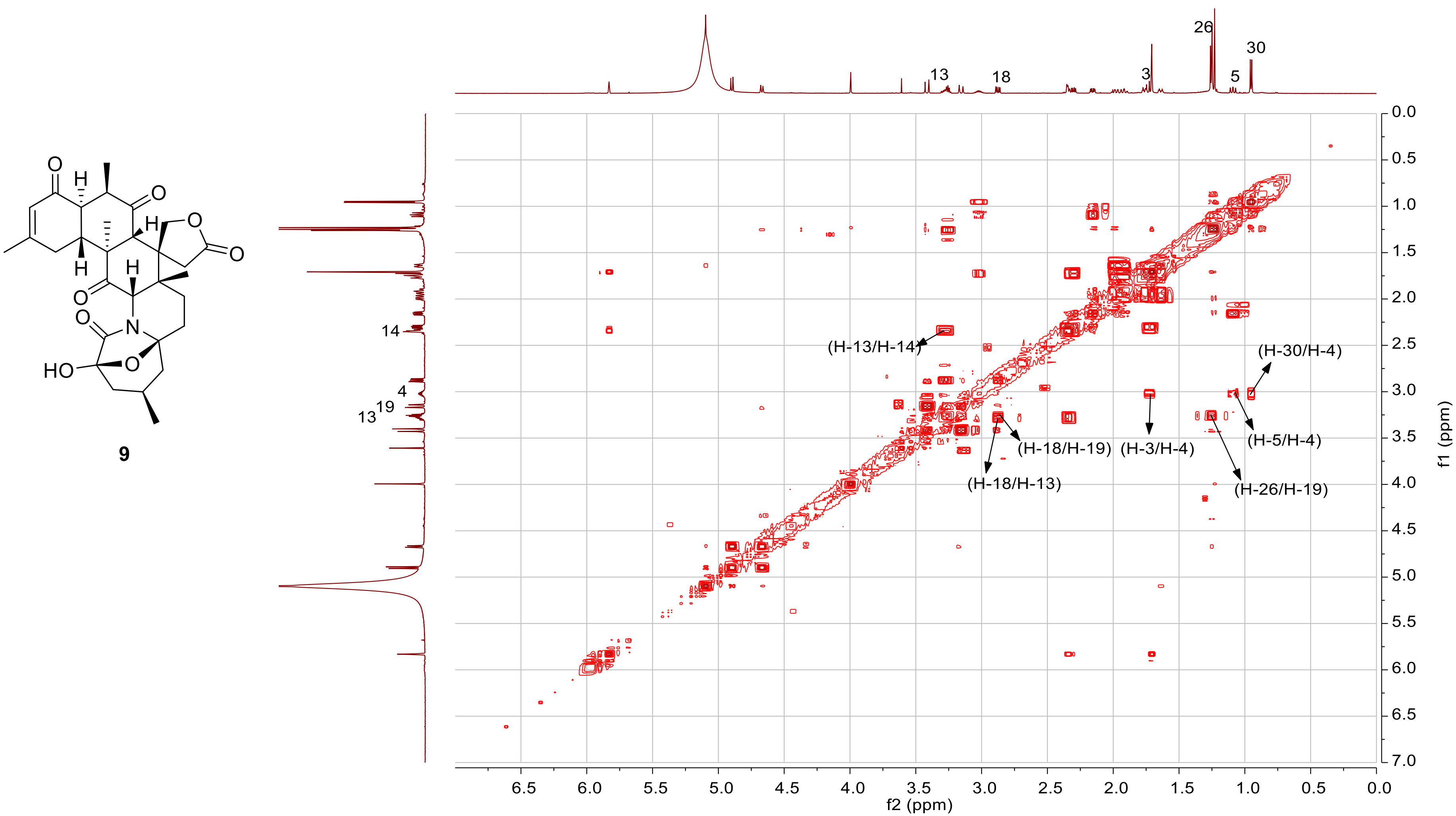

Figure S76. HSQC spectrum of **9**

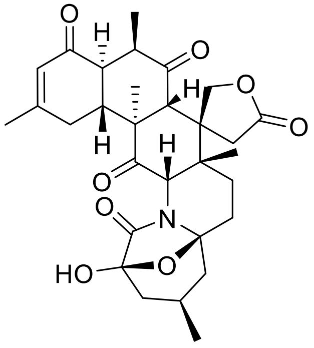

9

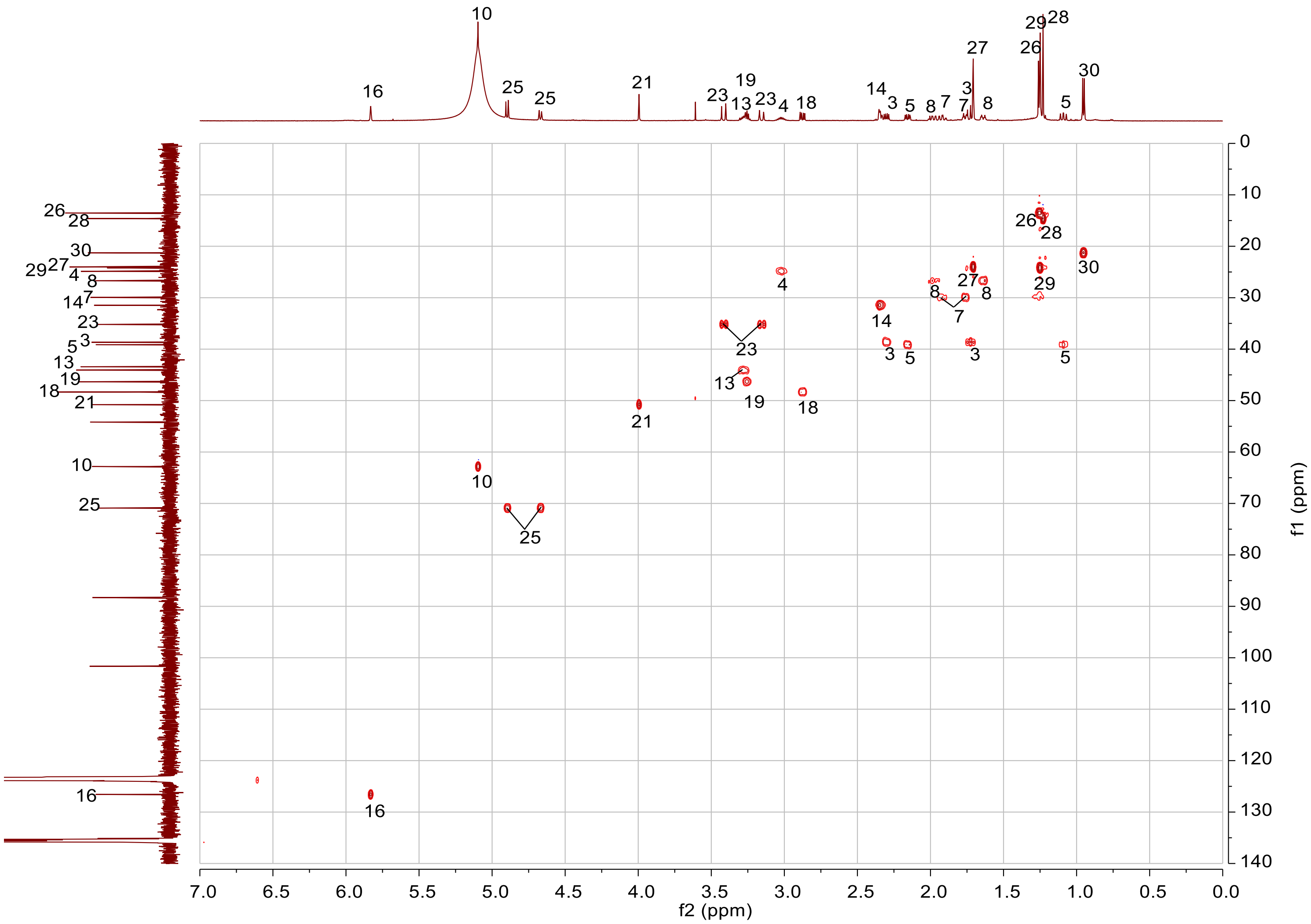

Figure S77. HMBC spectrum of **9**

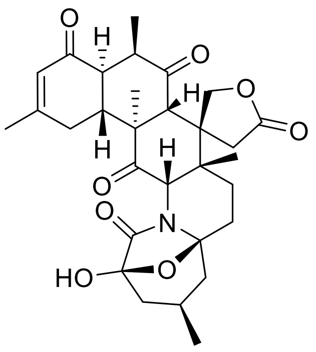

9

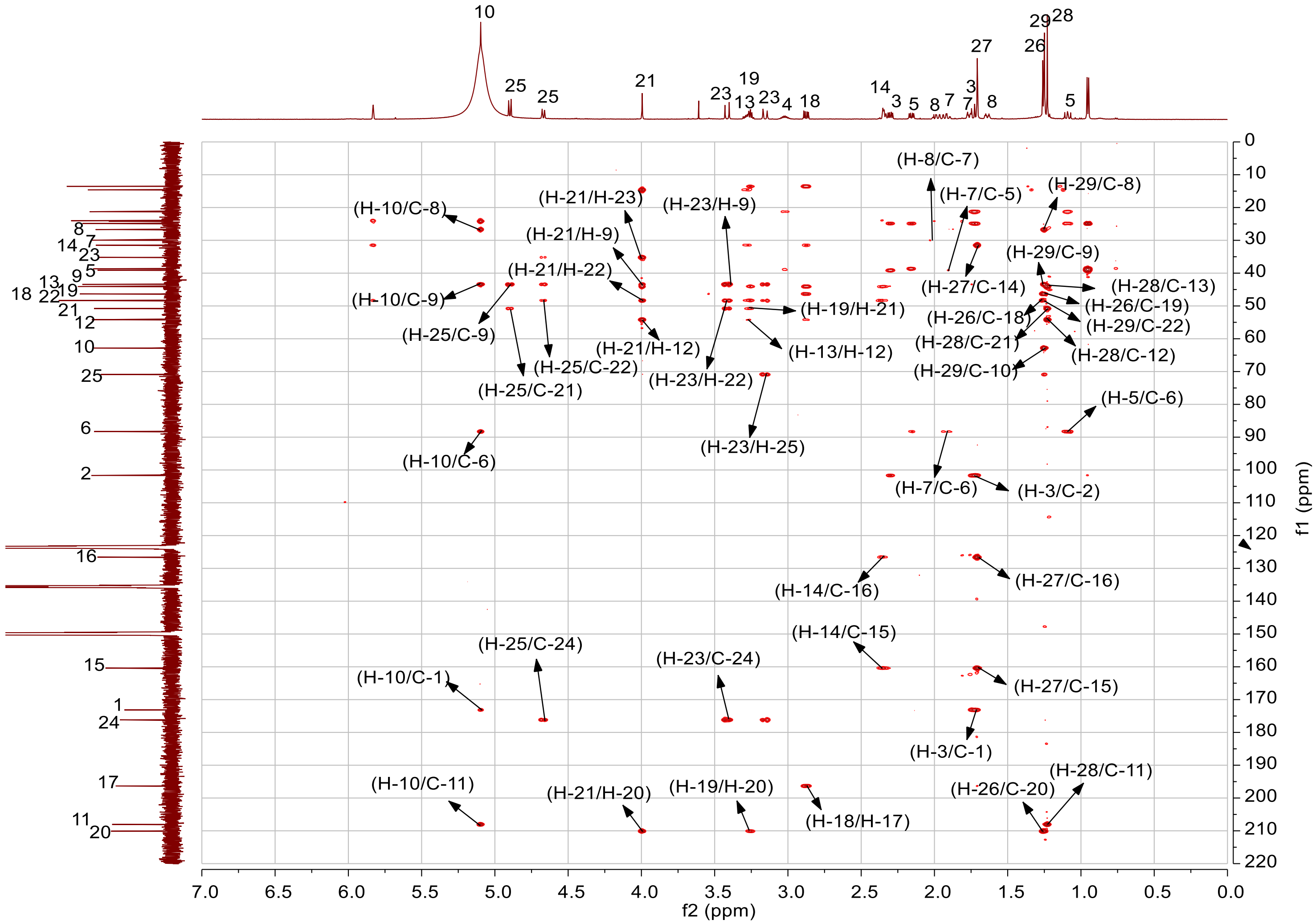

Figure S78. NOESY spectrum of **9**

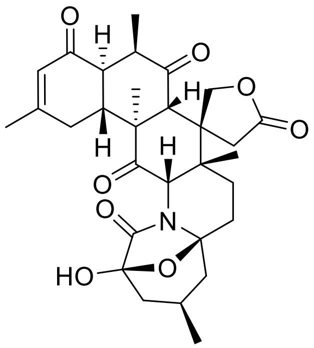

9

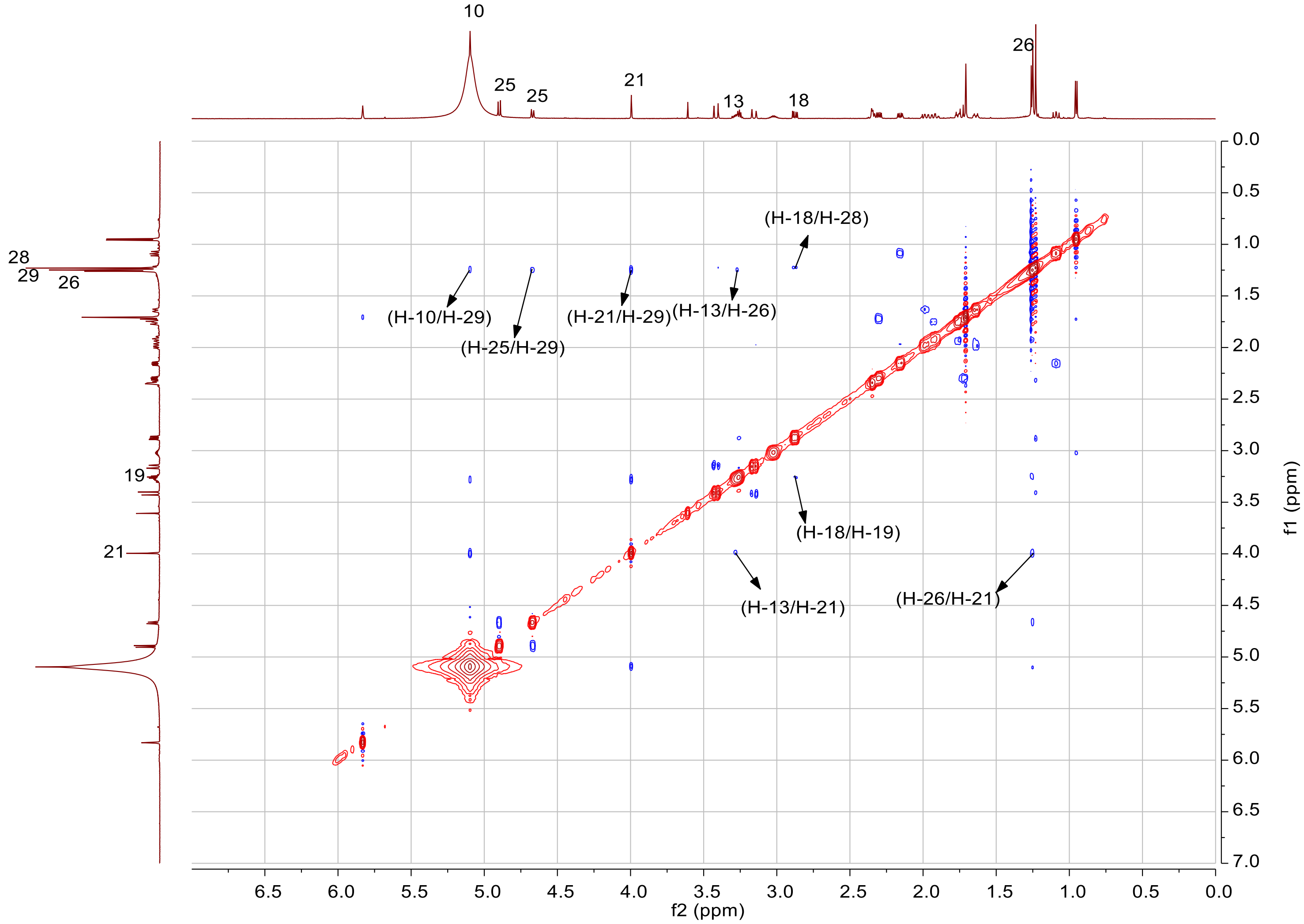

Figure S79. HRESIMS spectrum of **9**

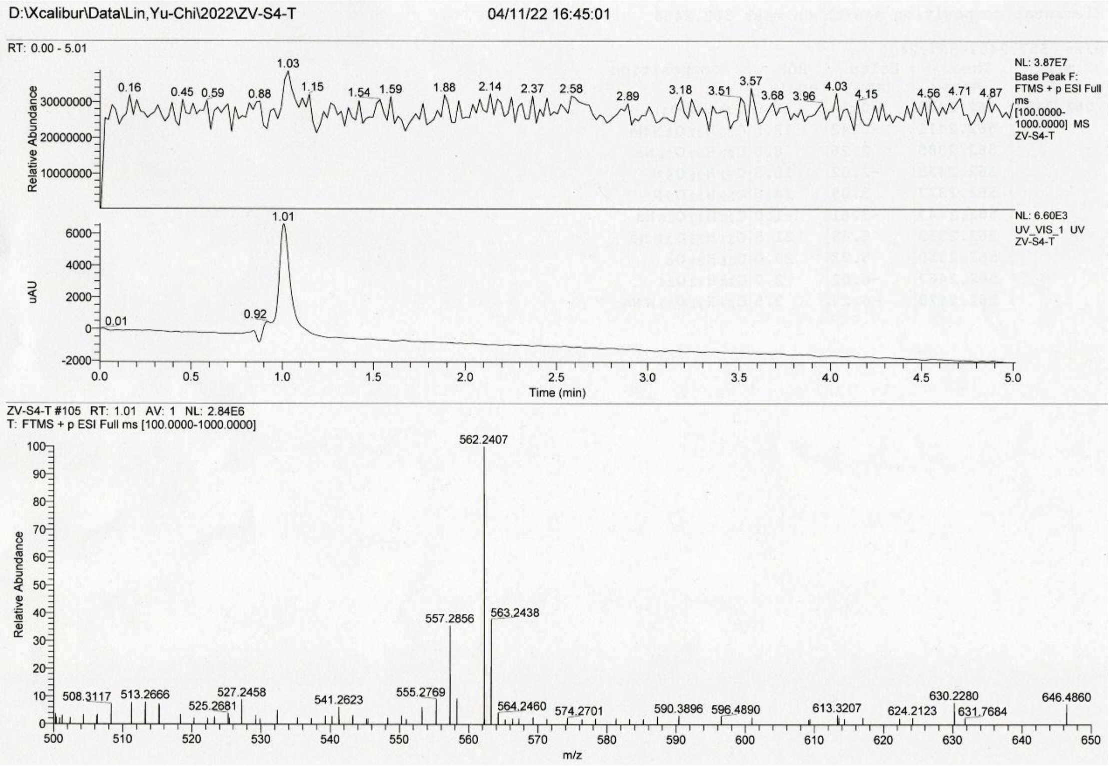

Elemental composition search on mass 562.2404

m/z= 557.2407-567.2400

m/z	Theo. Mass	Delta (mmu)	RDB equiv.	Composition
562.2407	562.2409	-0.14	11.0	$C_{29}H_{38}O_{11}$
	562.2411	-0.42	12.5	$C_{30}H_{37}O_{8}N\,Na$
	562.2385	2.26	8.0	$C_{27}H_{39}O_{11}Na$
	562.2435	-2.82	15.5	$C_{32}H_{36}O_{8}N$
	562.2377	3.05	24.5	$C_{39}H_{32}O_{3}N$
	562.2443	-3.61	-1.0	$C_{20}H_{43}O_{16}Na$
	562.2353	5.45	21.5	$C_{37}H_{33}O_{3}N\,Na$
	562.2350	5.73	20.0	$C_{36}H_{34}O_{6}$
	562.2467	-6.02	2.0	$C_{22}H_{42}O_{16}$
	562.2470	-6.29	3.5	$C_{23}H_{41}O_{13}N\,Na$

Figure S80. UV spectrum of **9**

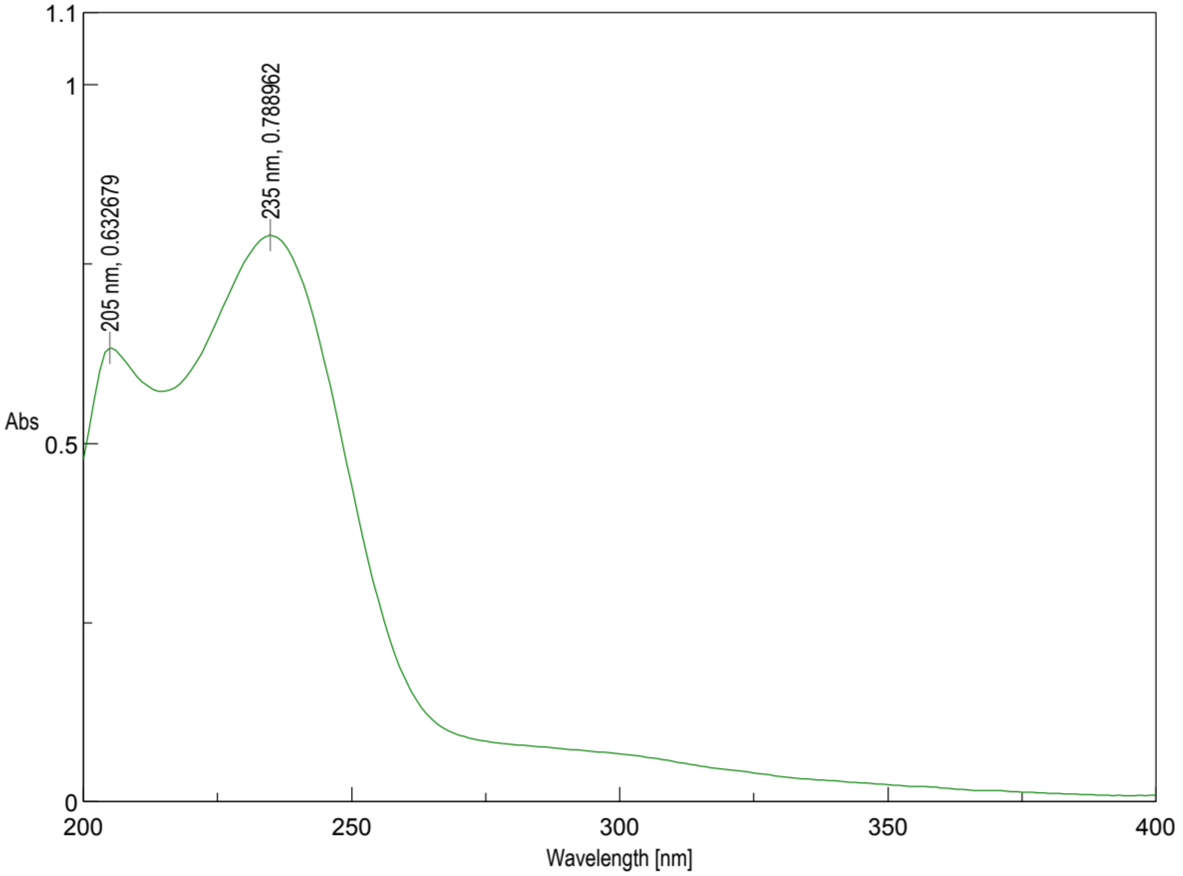

Figure S81. IR spectrum of **9**

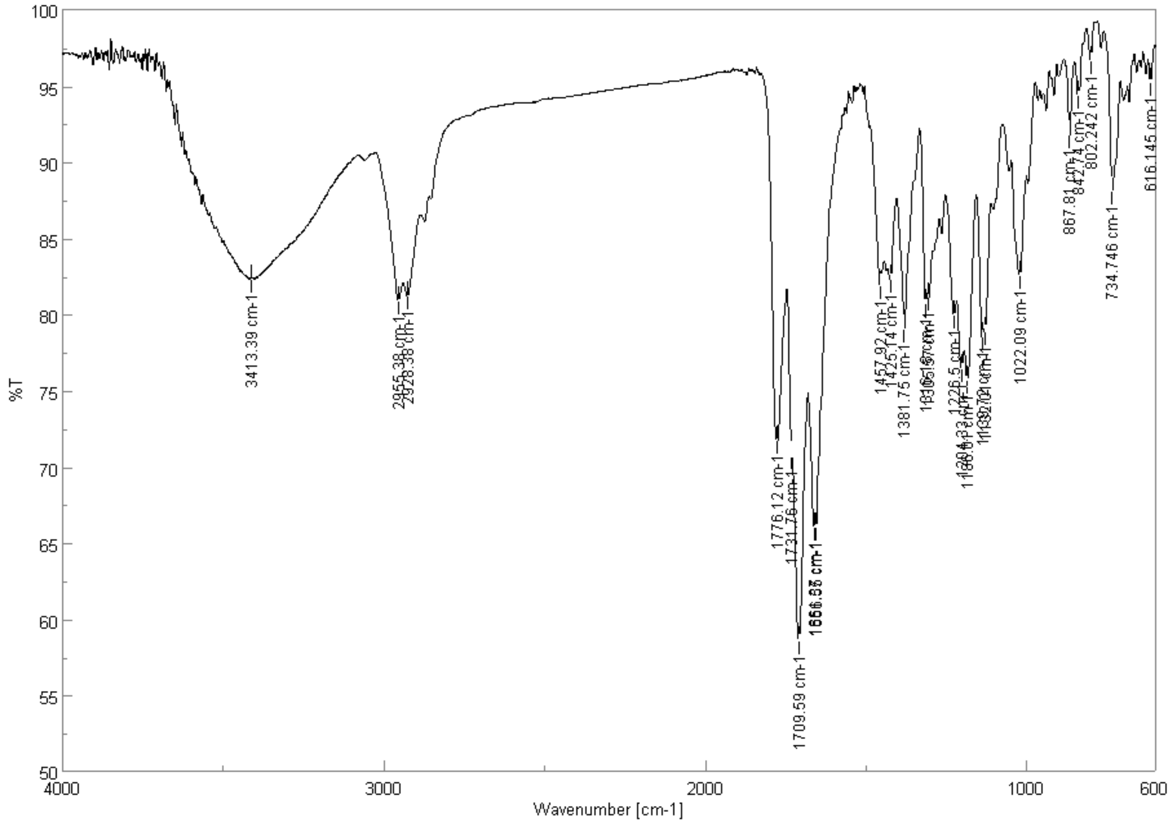

Figure S82. ^{1}H NMR spectrum of **10** (C_5D_5N, 600 MHz)

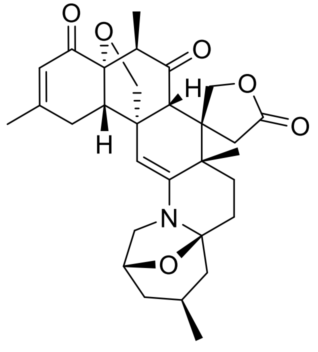

10

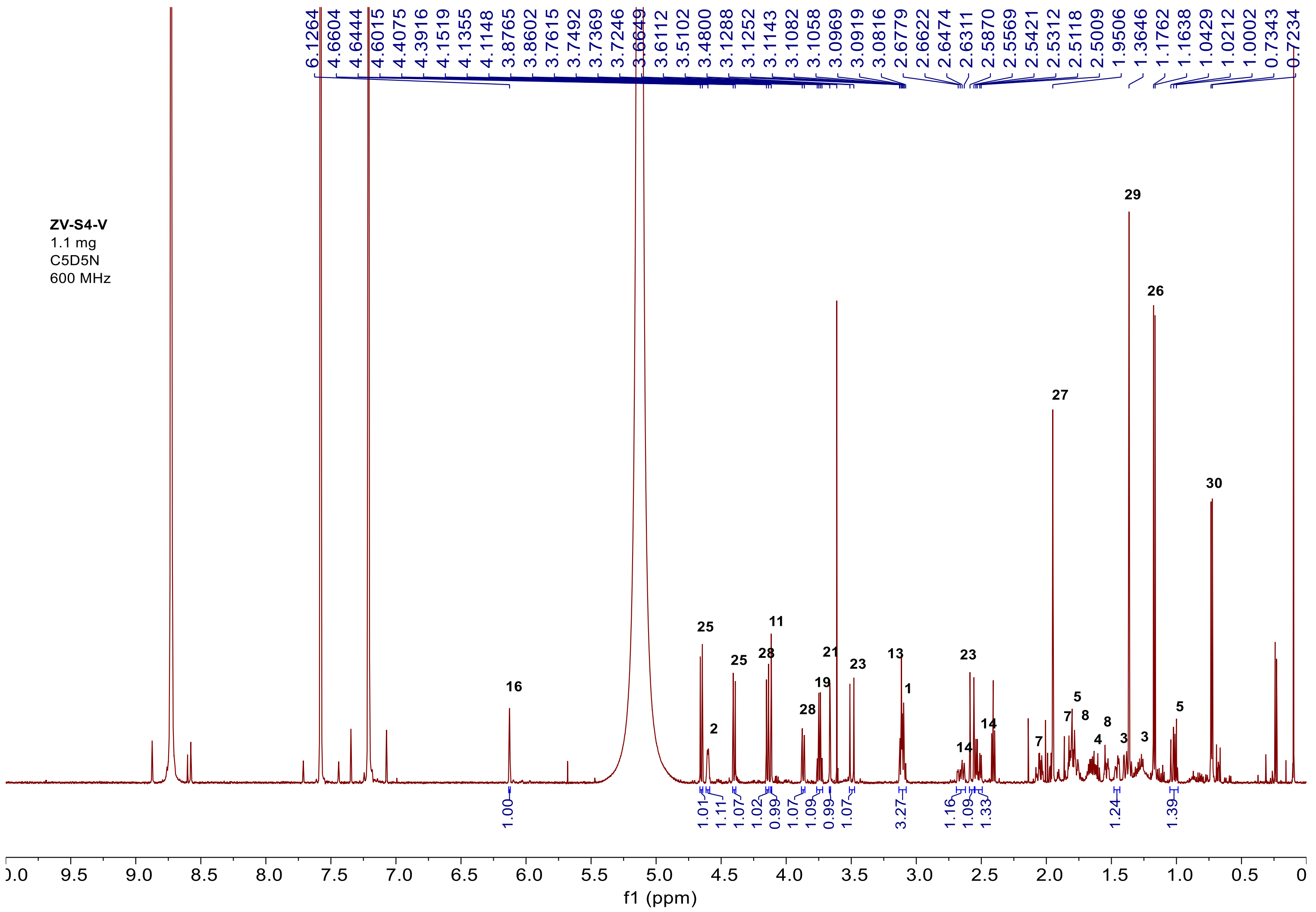

Figure S83. $^{13}C\{^{1}H\}$ NMR and DEPT spectra of **10** (C_5D_5N, 150 MHz)

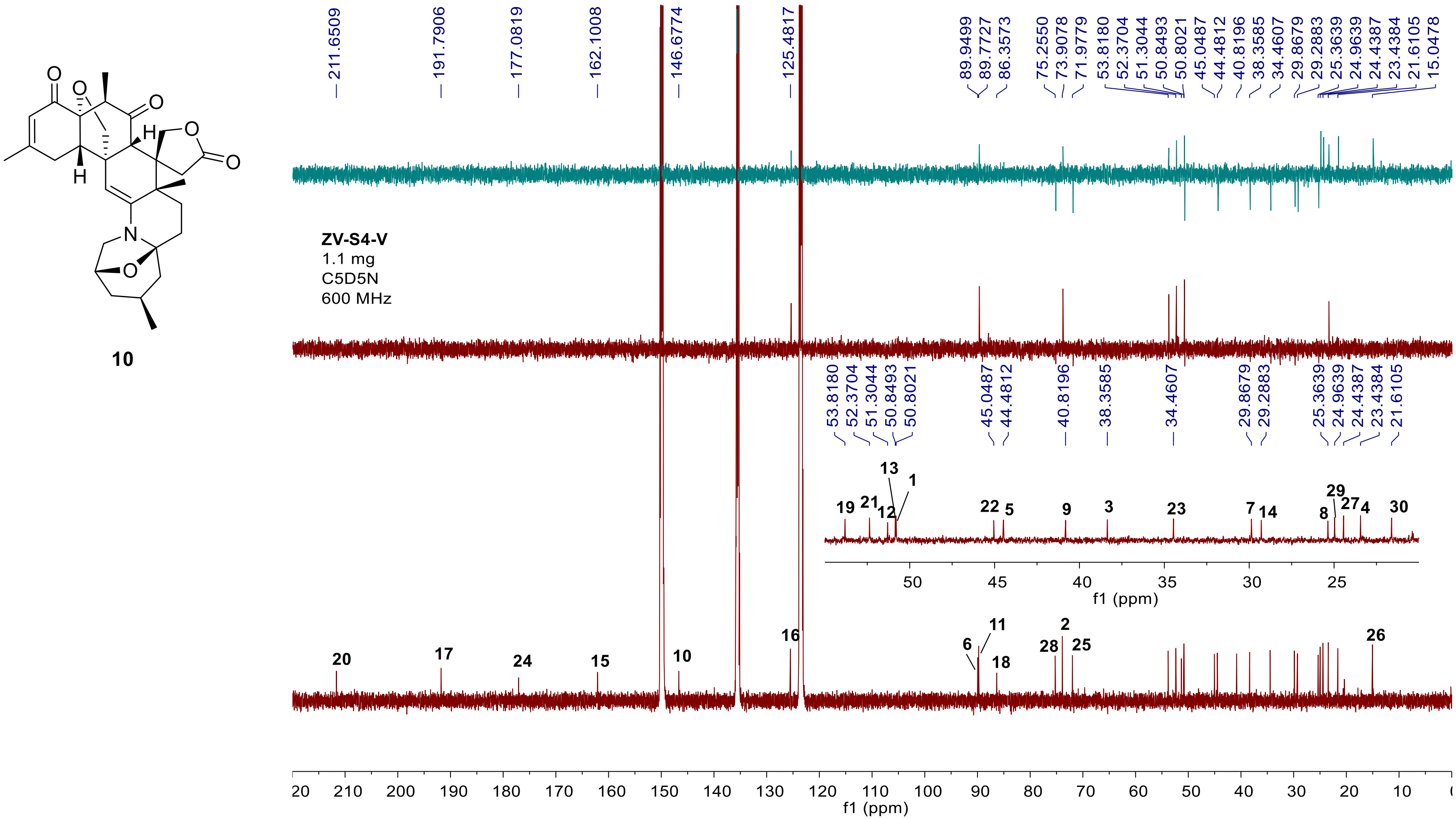

Figure S84. COSY spectrum of **10**

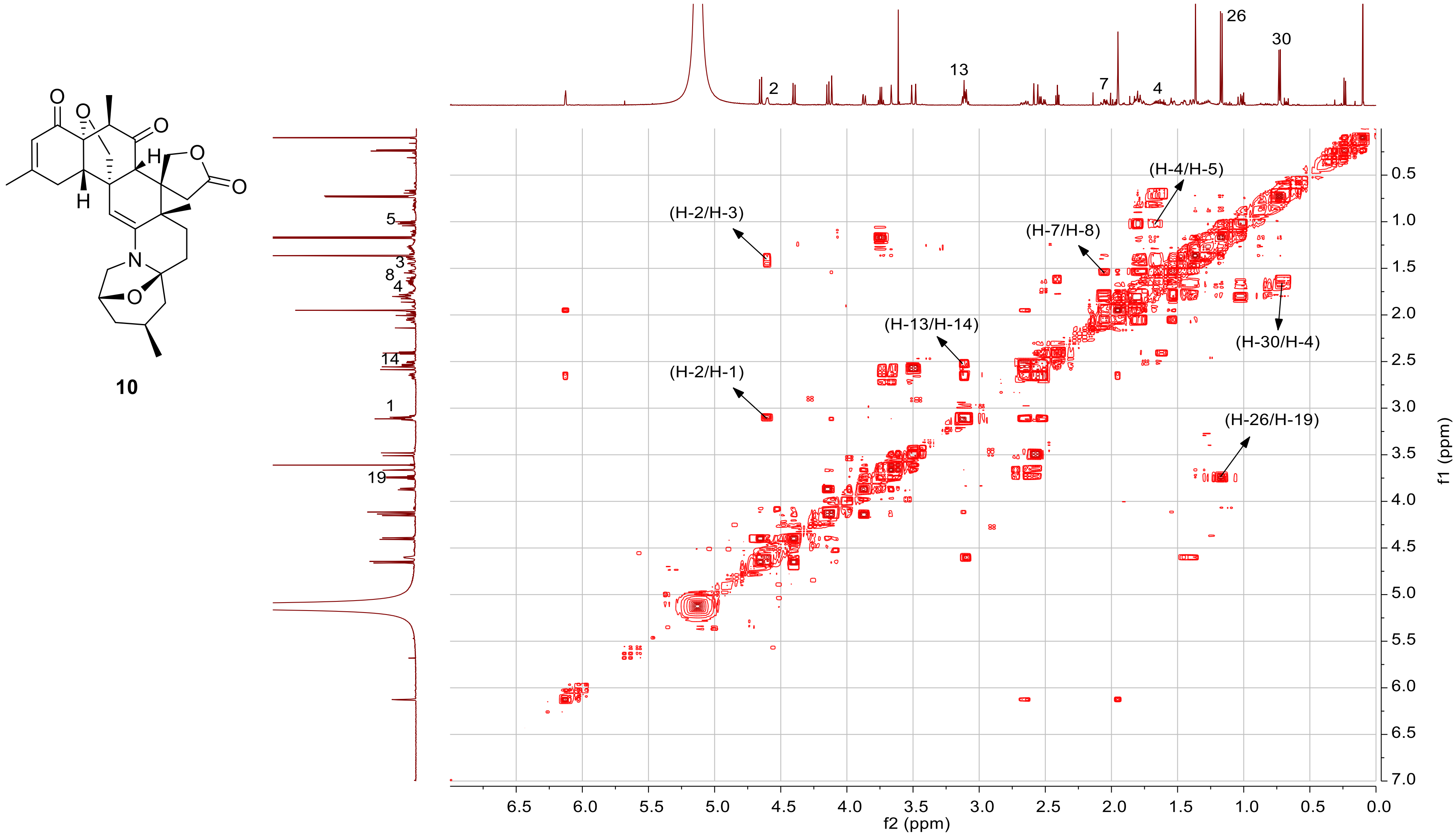

Figure S85. HSQC spectrum of **10**

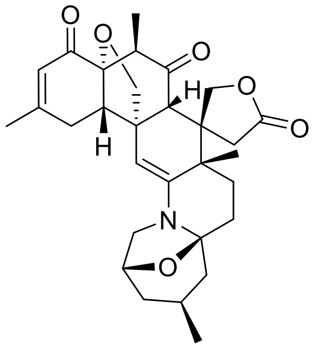

10

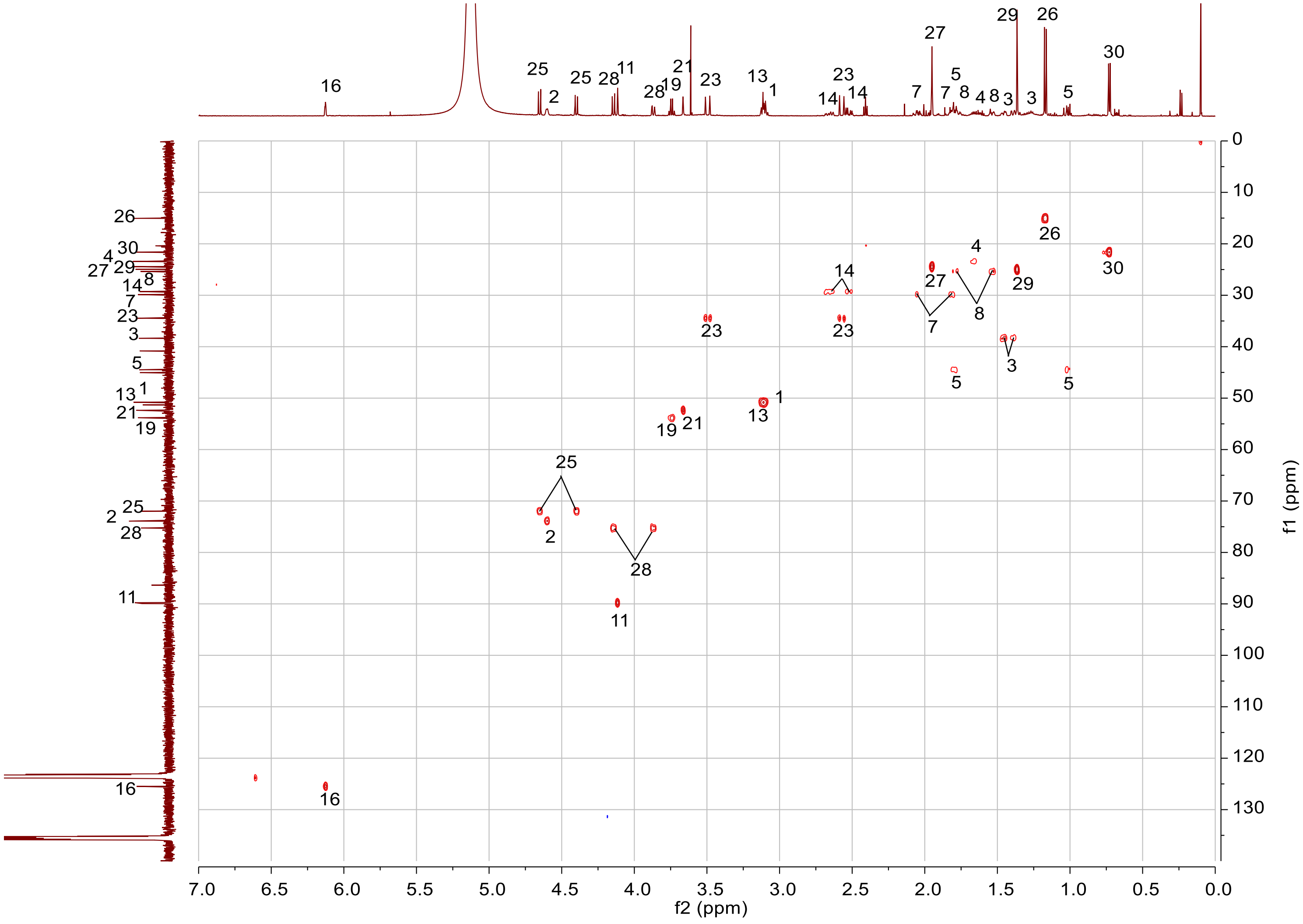

Figure S86. HMBC spectrum of **10**

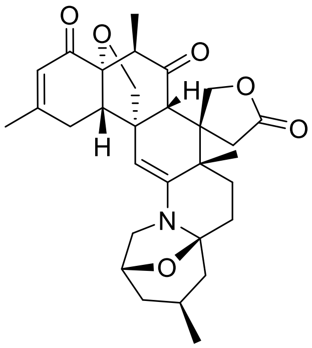

10

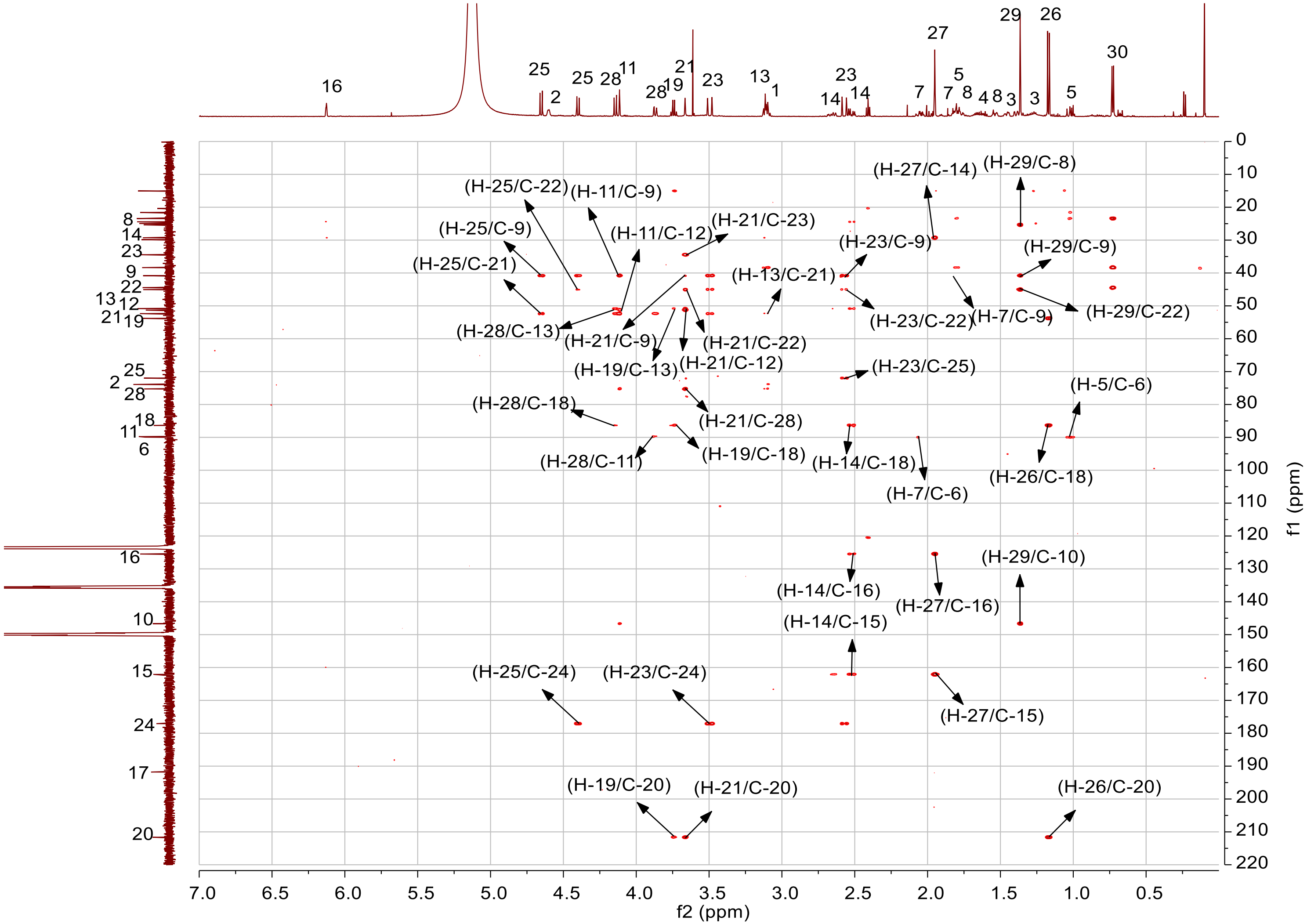

Figure S87. NOESY spectrum of **10**

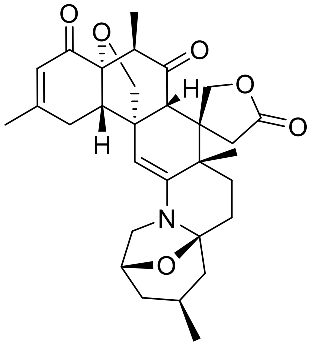

10

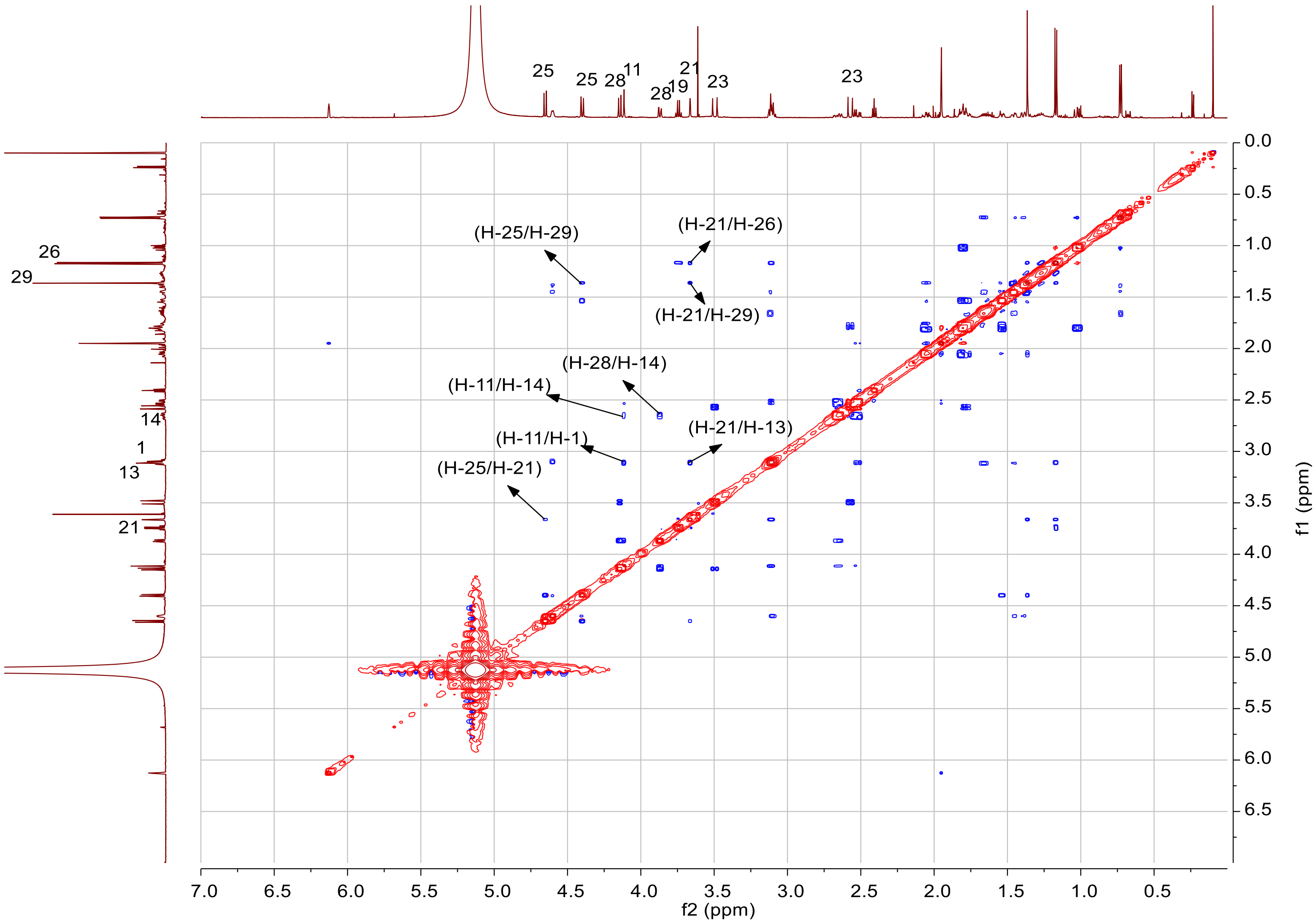

Figure S88. HRESIMS spectrum of **10**

Mass Spectrum SmartFormula Report

Analysis Info

Analysis Name	D:\QTOF\ZVS4V.d	6/27/2022 11:29:27 AM
Method	tune_wide_pos_20220422.m	Operator: YU HSIAO-CHING
Sample Name	ZVS4-V	Instrument: BRUKER micrOTOF-Q
Comment	ESI Positive	

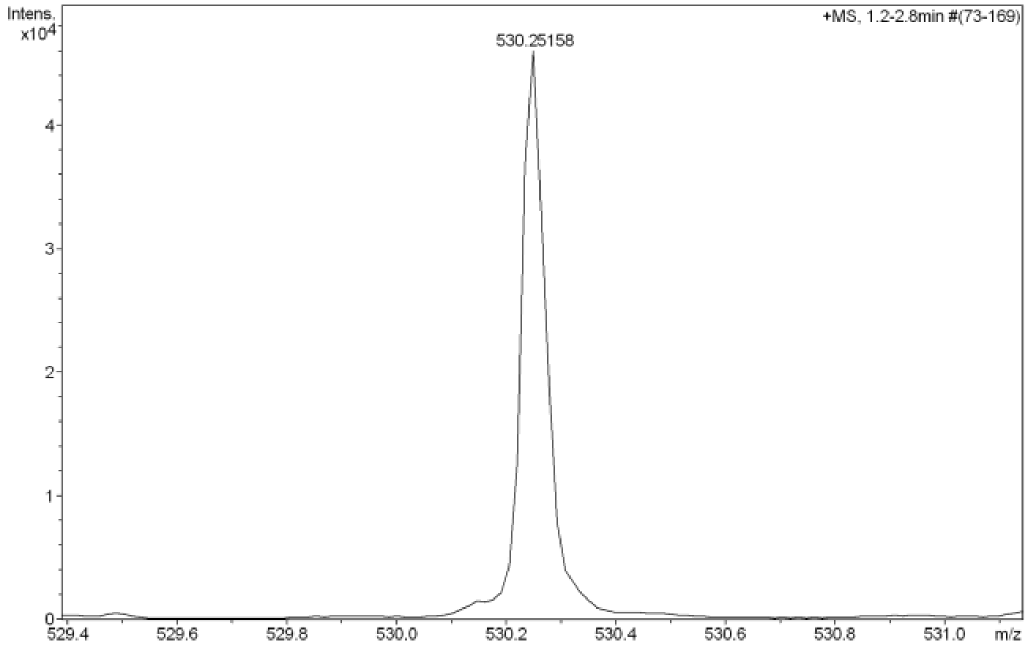

Meas. m/z	#	Formula	Score	m/z	err [mDa]	err [ppm]	mSigma	rdb	e⁻ Conf	N-Rule
530.25158	1	C 30 H 37 N Na O 6	100.00	530.25131	-0.27	-0.50	23.0	12.5	even	ok

Figure S89. UV spectrum of **10**

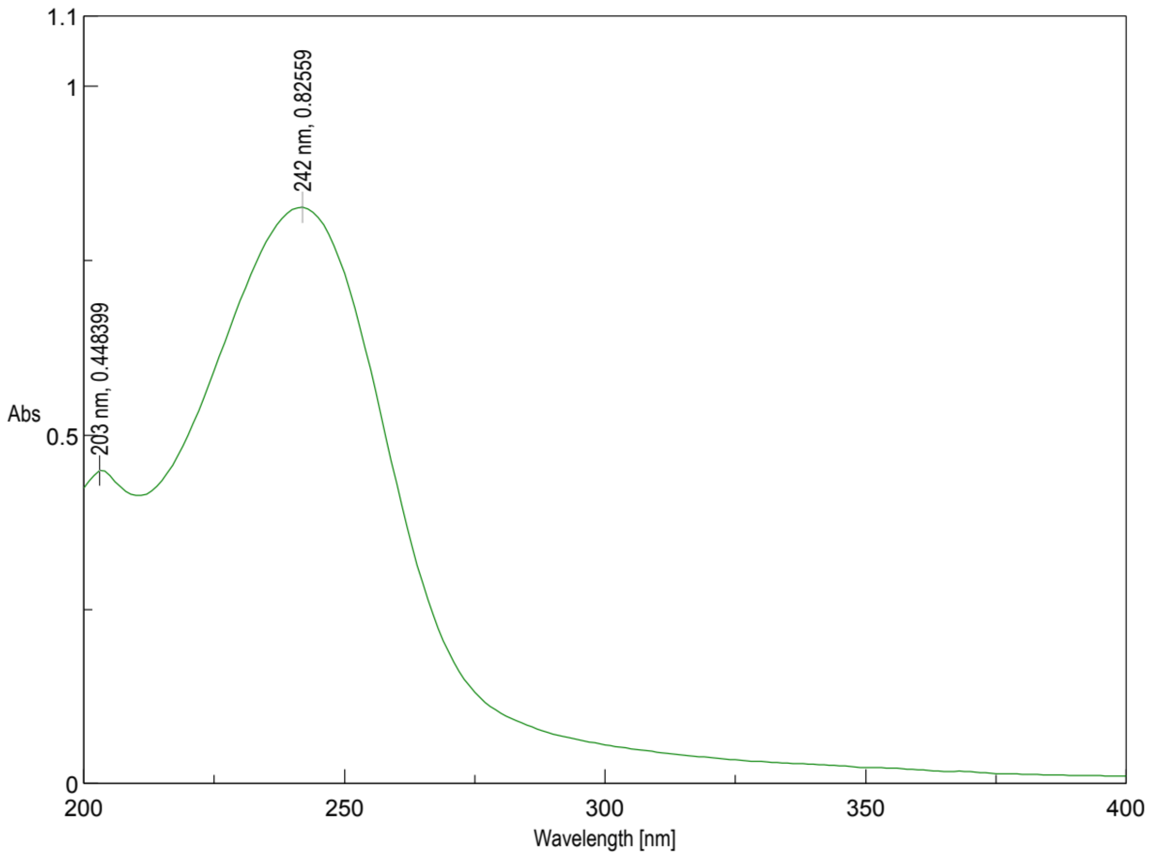

Figure S90. IR spectrum of **10**

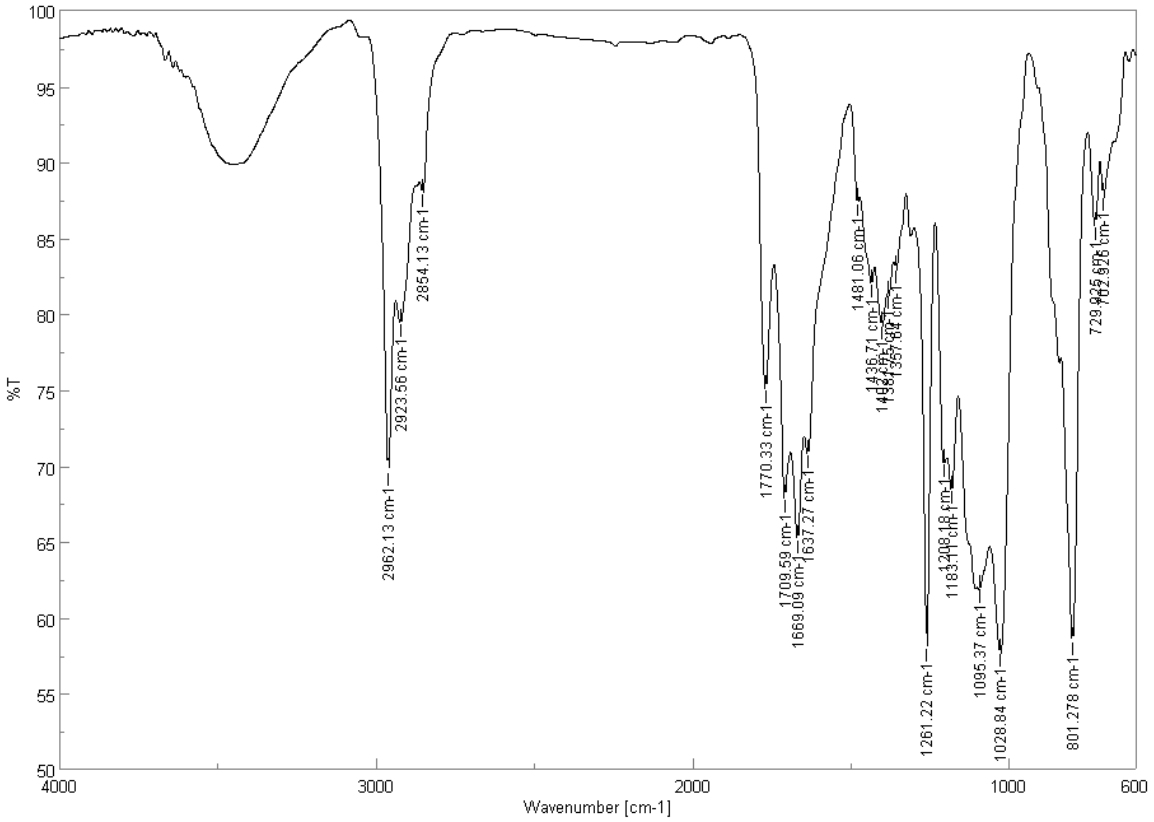

Figure S91. ^{1}H NMR spectrum of **11** (C_5D_5N, 600 MHz)

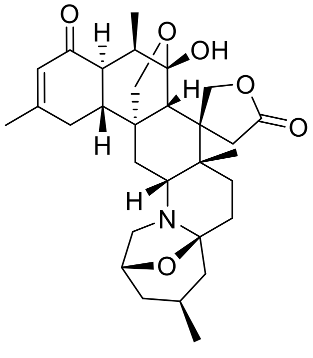

11

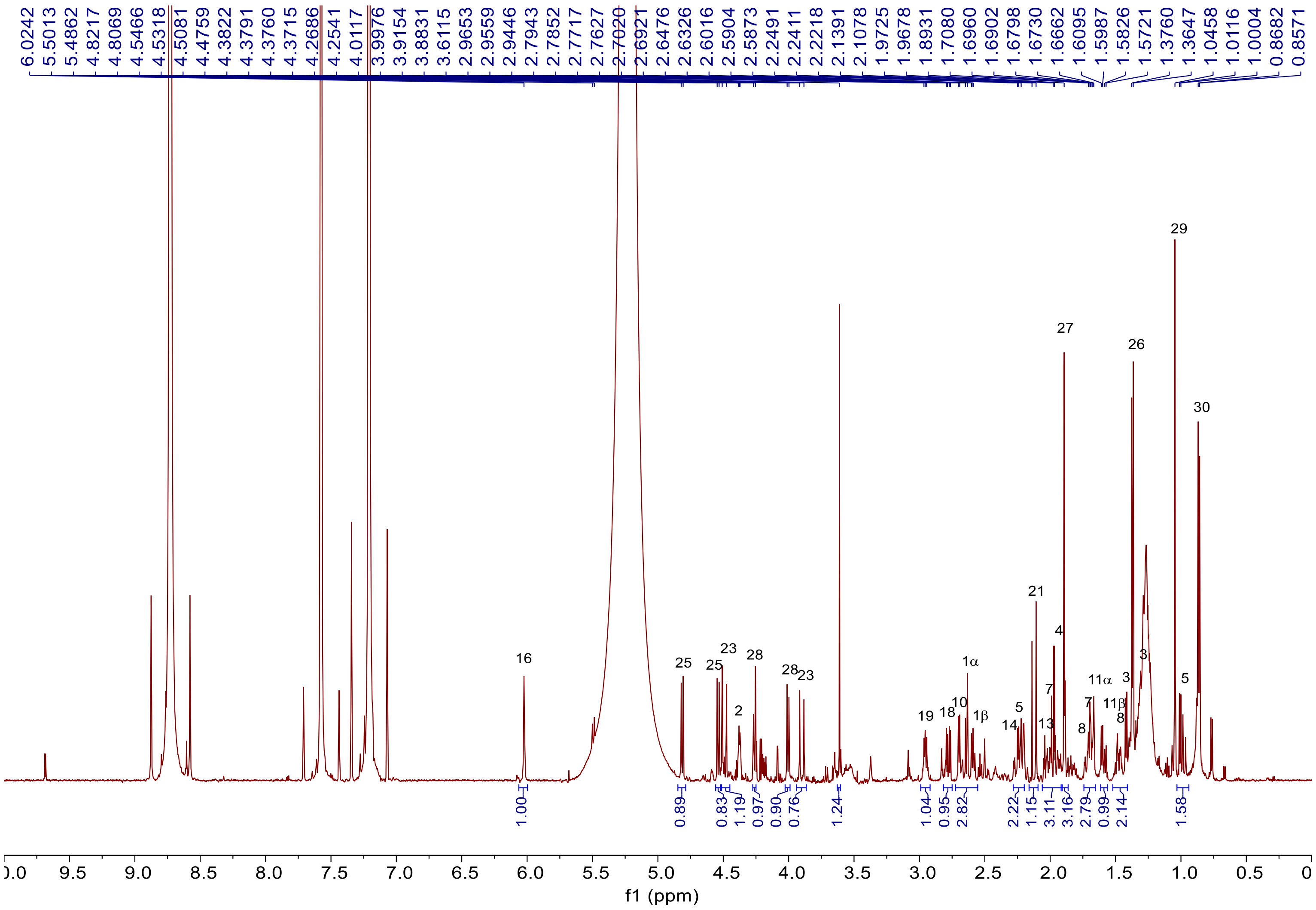

Figure S92. $^{13}C\{^1H\}$ NMR and DEPT spectra of **11** (C_5D_5N, 150 MHz)

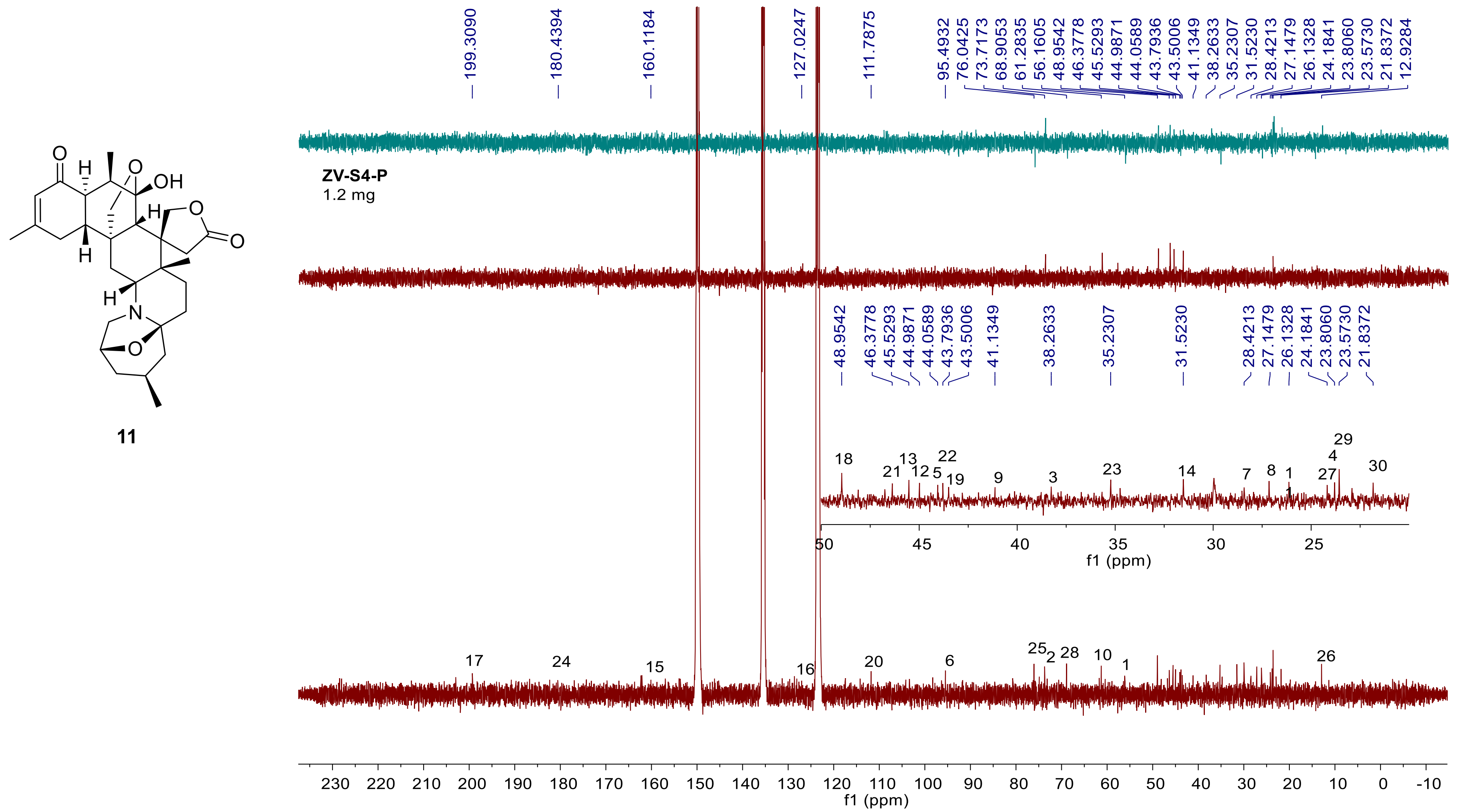

Figure S93. COSY spectrum of **11**

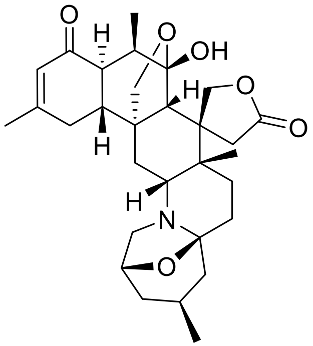

11

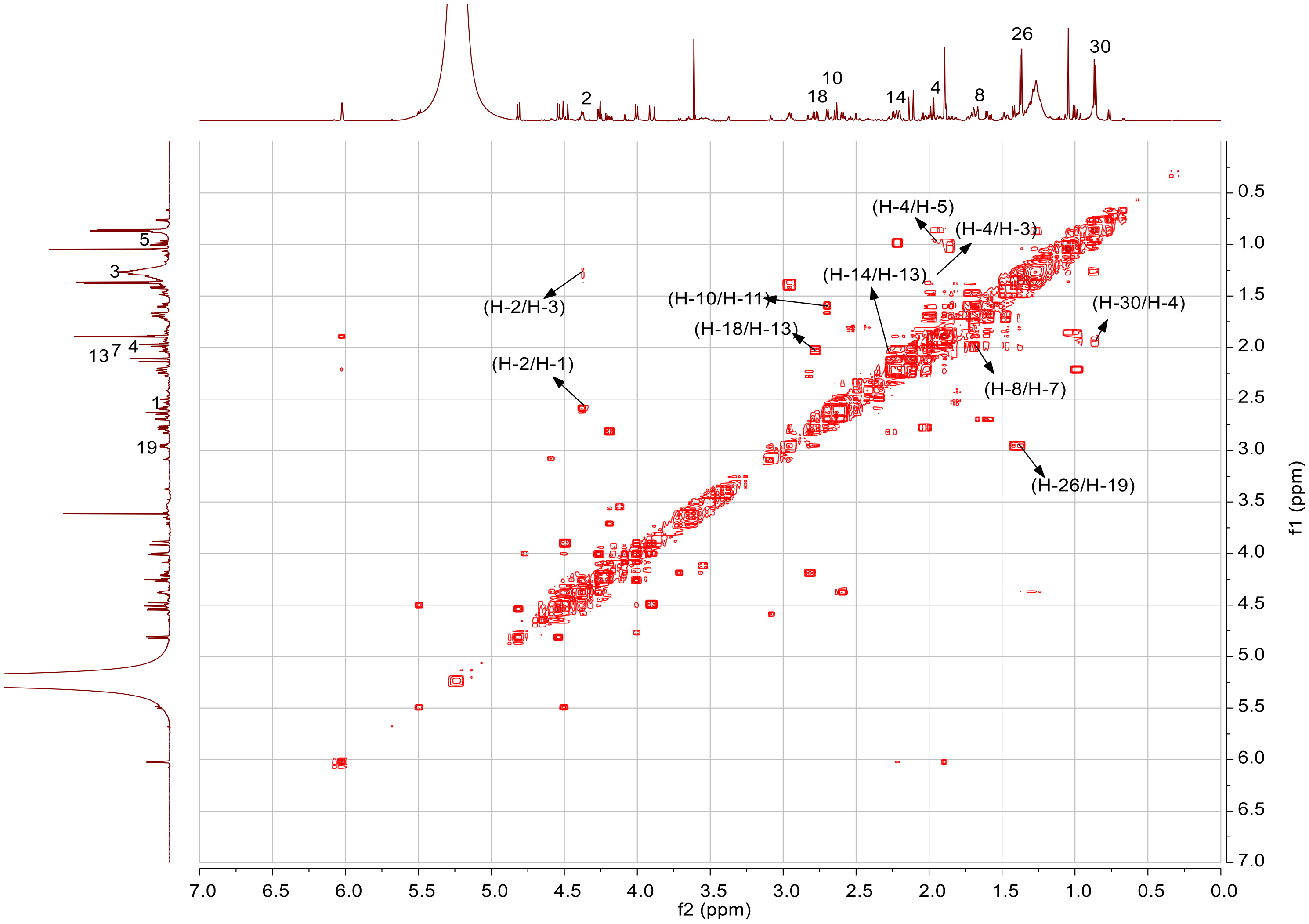

Figure S94. HSQC spectrum of **11**

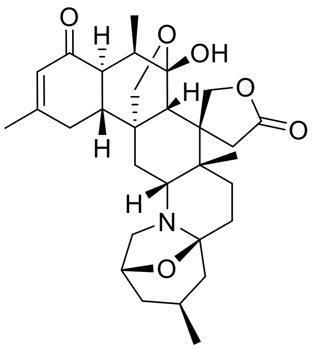

11

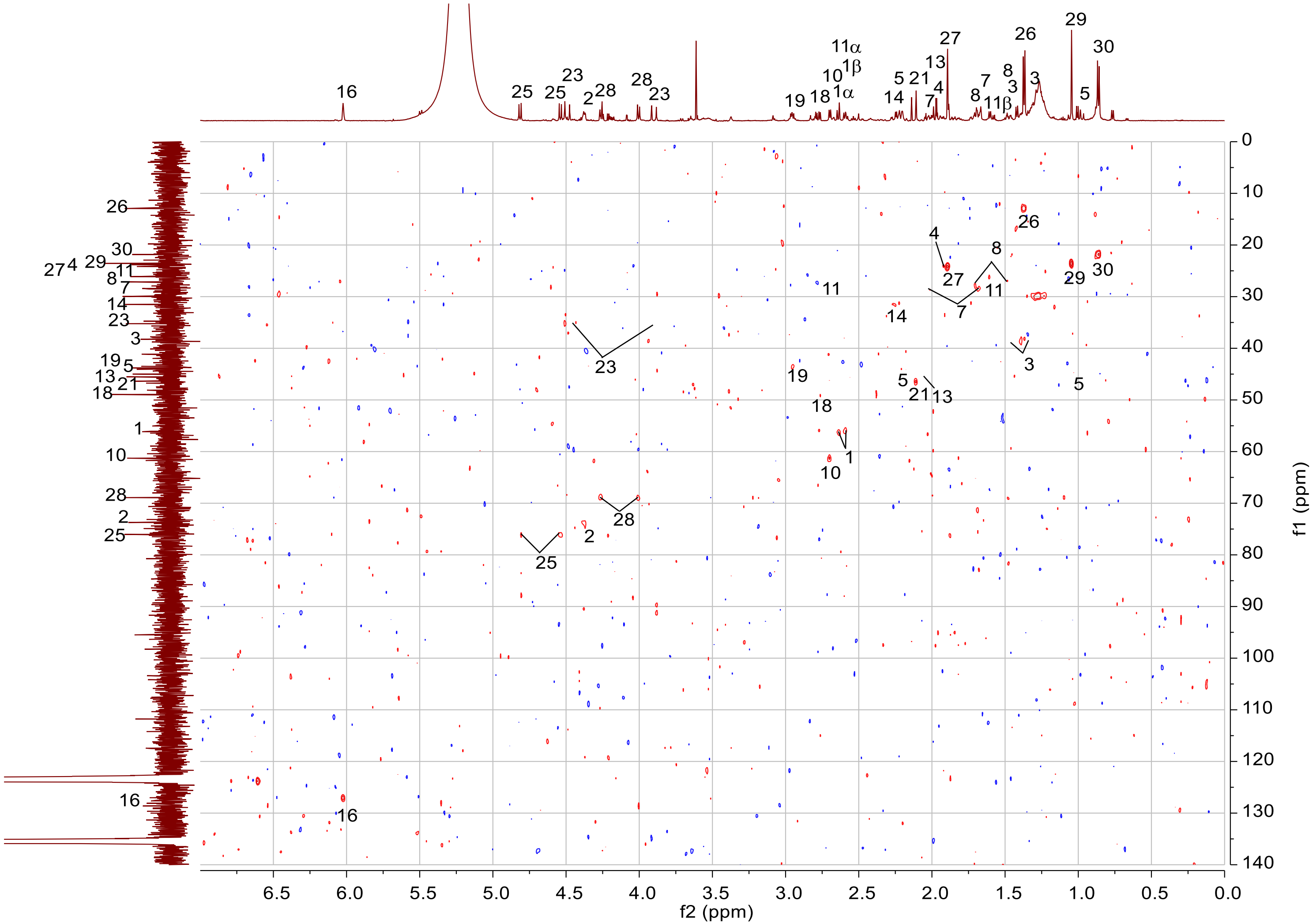

Figure S95. HMBC spectrum of **11**

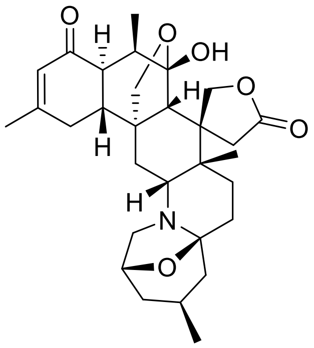

11

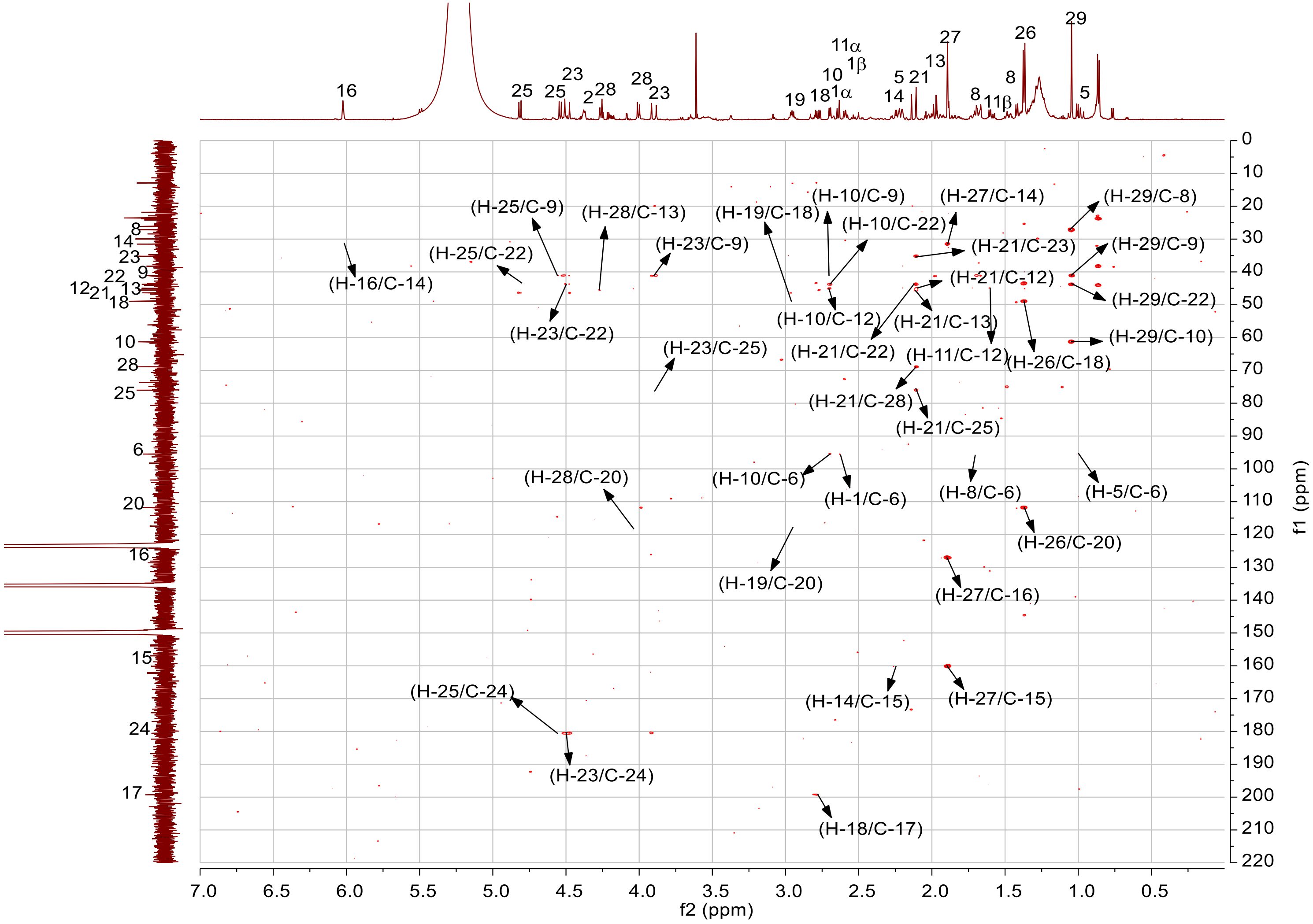

Figure S96. NOESY spectrum of **11**

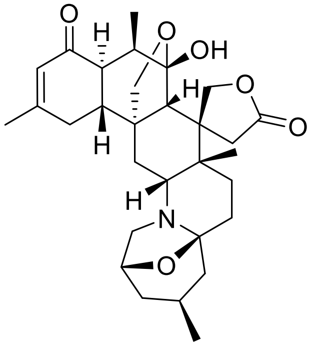

11

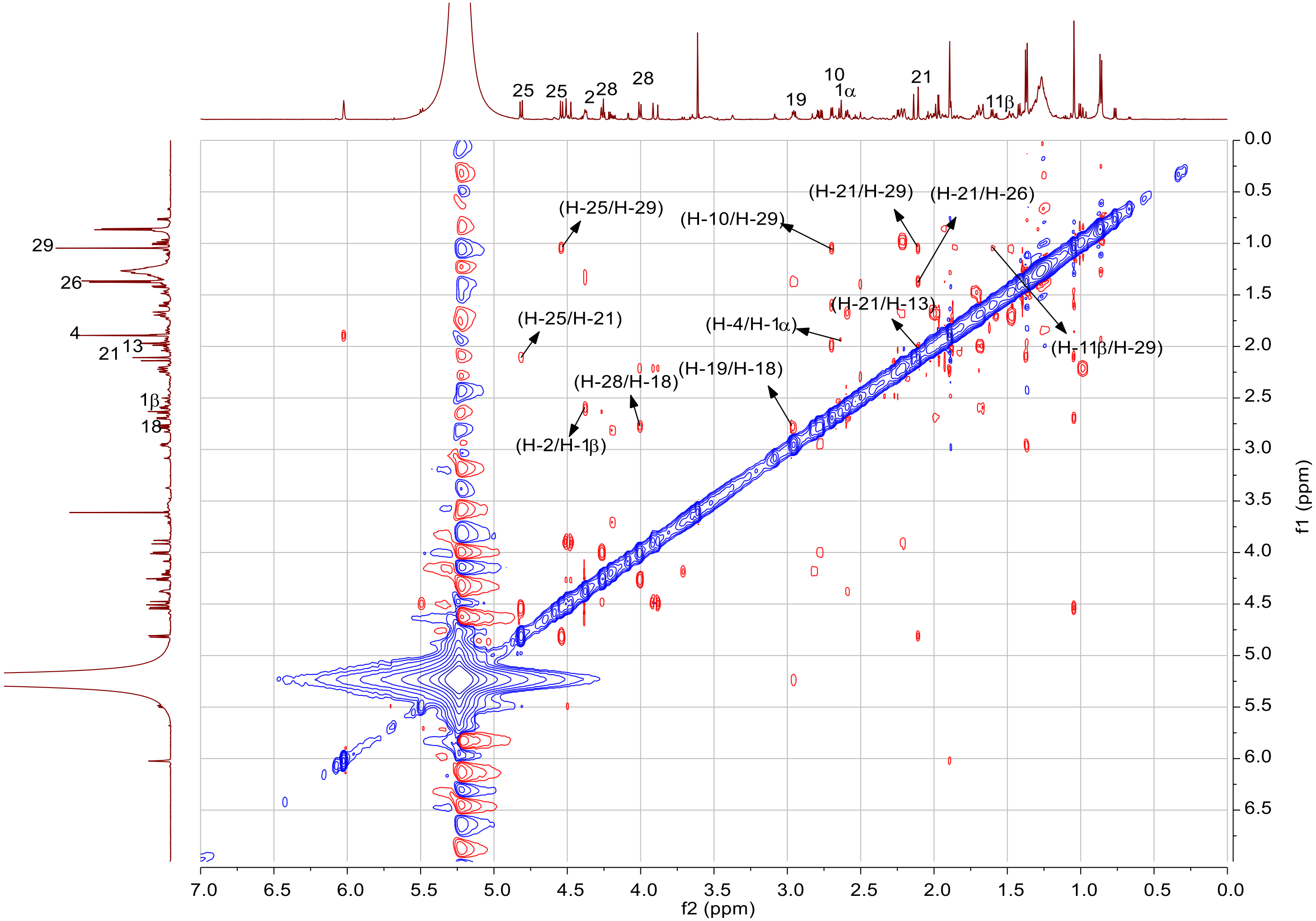

Figure S97. HRESIMS spectrum of **11**

Mass Spectrum SmartFormula Report

Analysis Info

Analysis Name	D:\QTOF\ZVS4PR1.d	2/24/2022 2:42:49 PM
Method	_tune_low_pos(50-1000)_20170502_Liao lab.m	Operator: YU HSIAO-CHING
Sample Name	ZV-S4-P	Instrument: BRUKER micrOTOF-Q
Comment	ESI Positive	

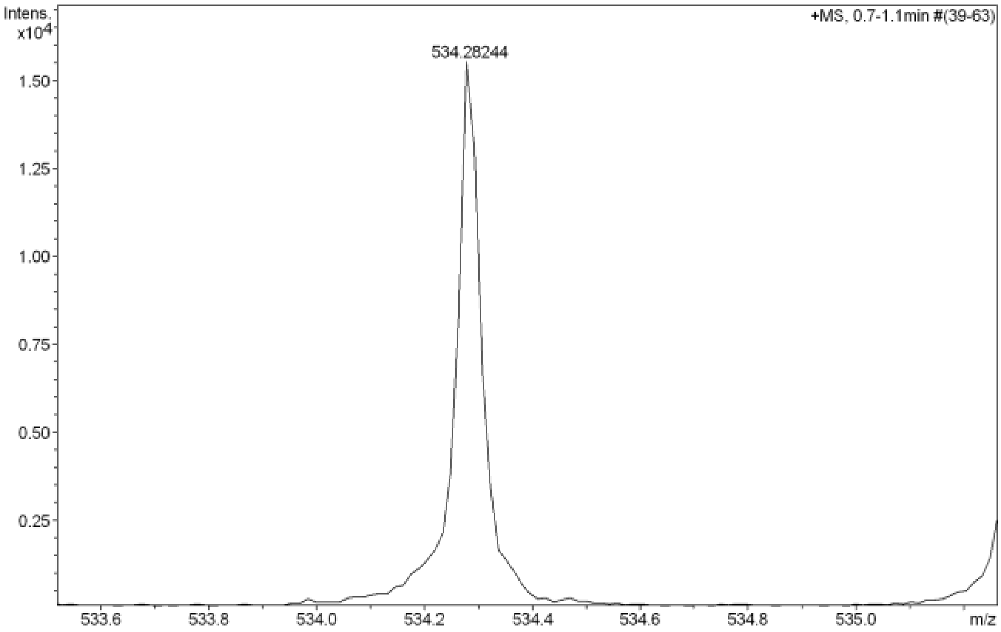

Meas. m/z	#	Formula	Score	m/z	err [mDa]	err [ppm]	mSigma	rdb	e⁻ Conf	N-Rule
534.28244	1	C 30 H 41 N Na O 6	100.00	534.28261	0.17	0.31	11.2	10.5	even	ok

Figure S98. UV spectrum of **11**

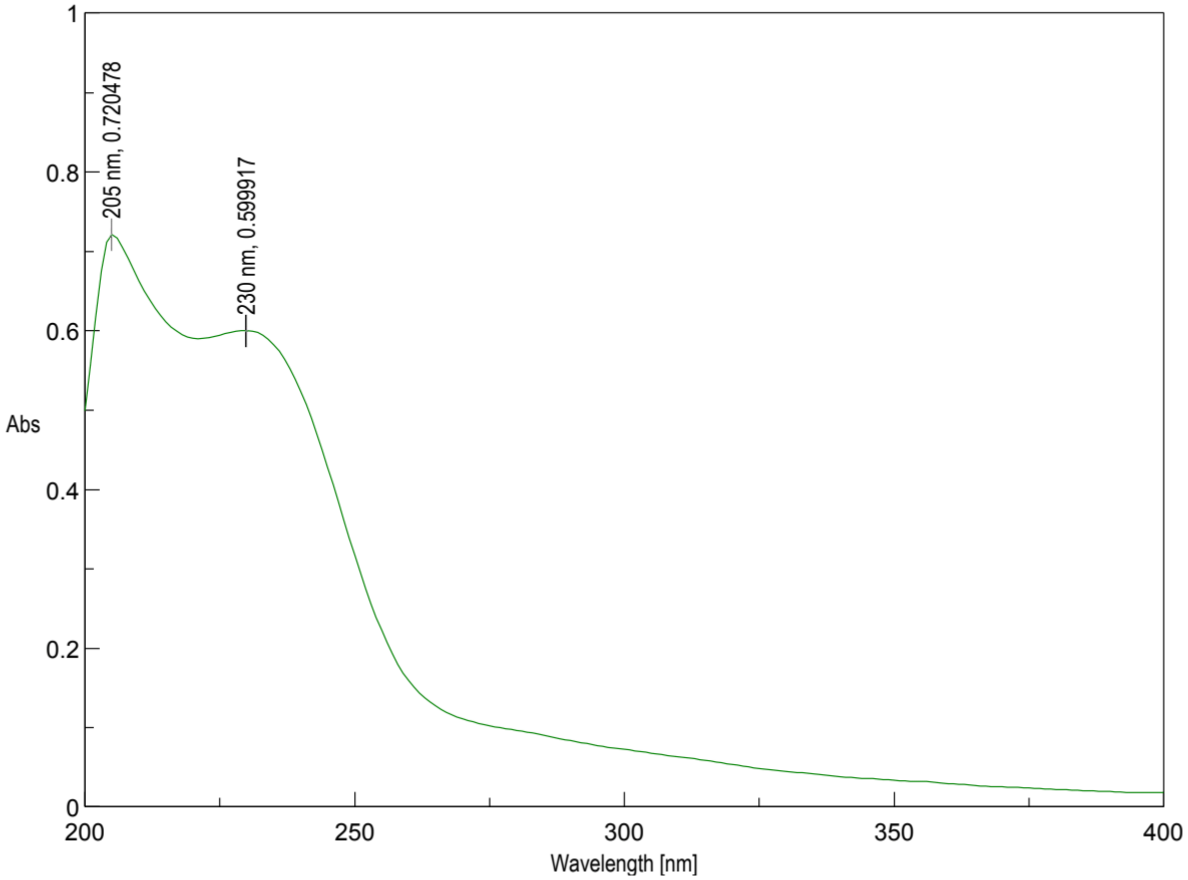

Figure S99. IR spectrum of **11**

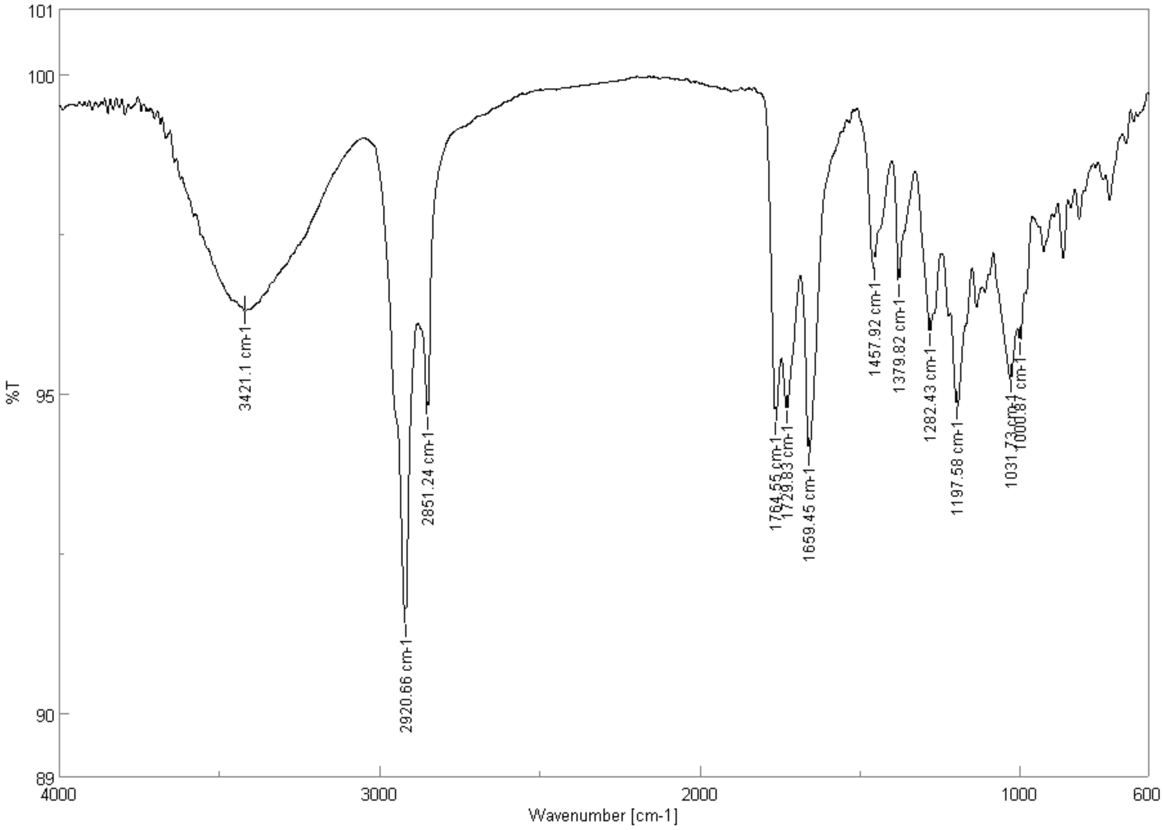

Figure S100. ^{1}H NMR spectrum of **12** (C_5D_5N, 600 MHz)

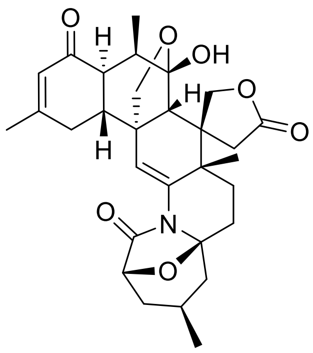

12

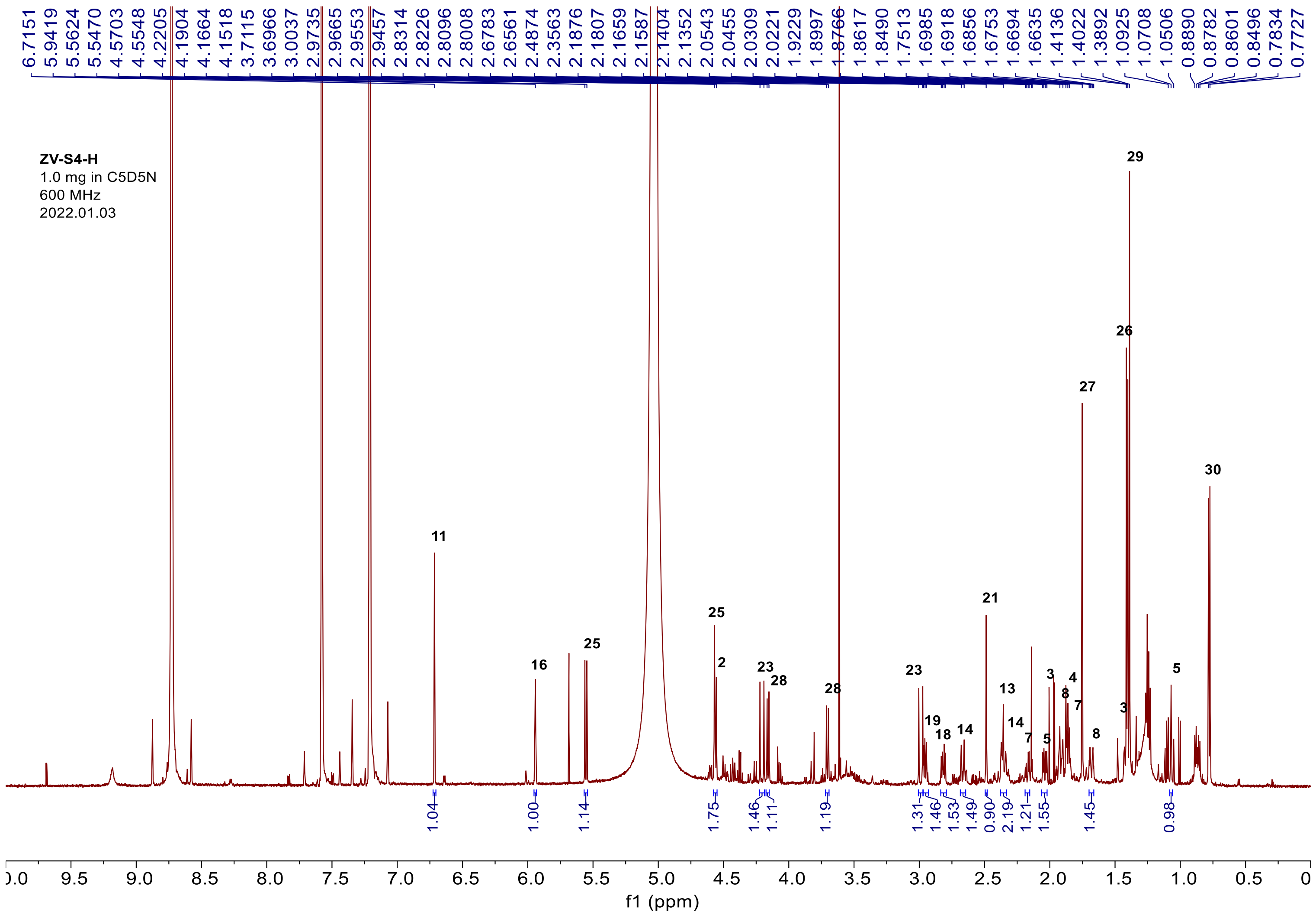

Figure S101. $^{13}C\{^{1}H\}$ NMR and DEPT spectra of **12** (C_5D_5N, 150 MHz)

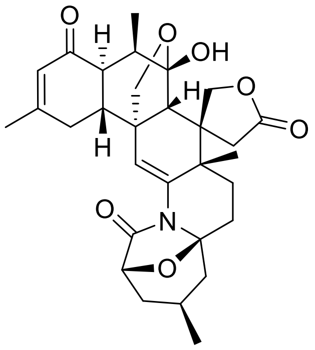

12

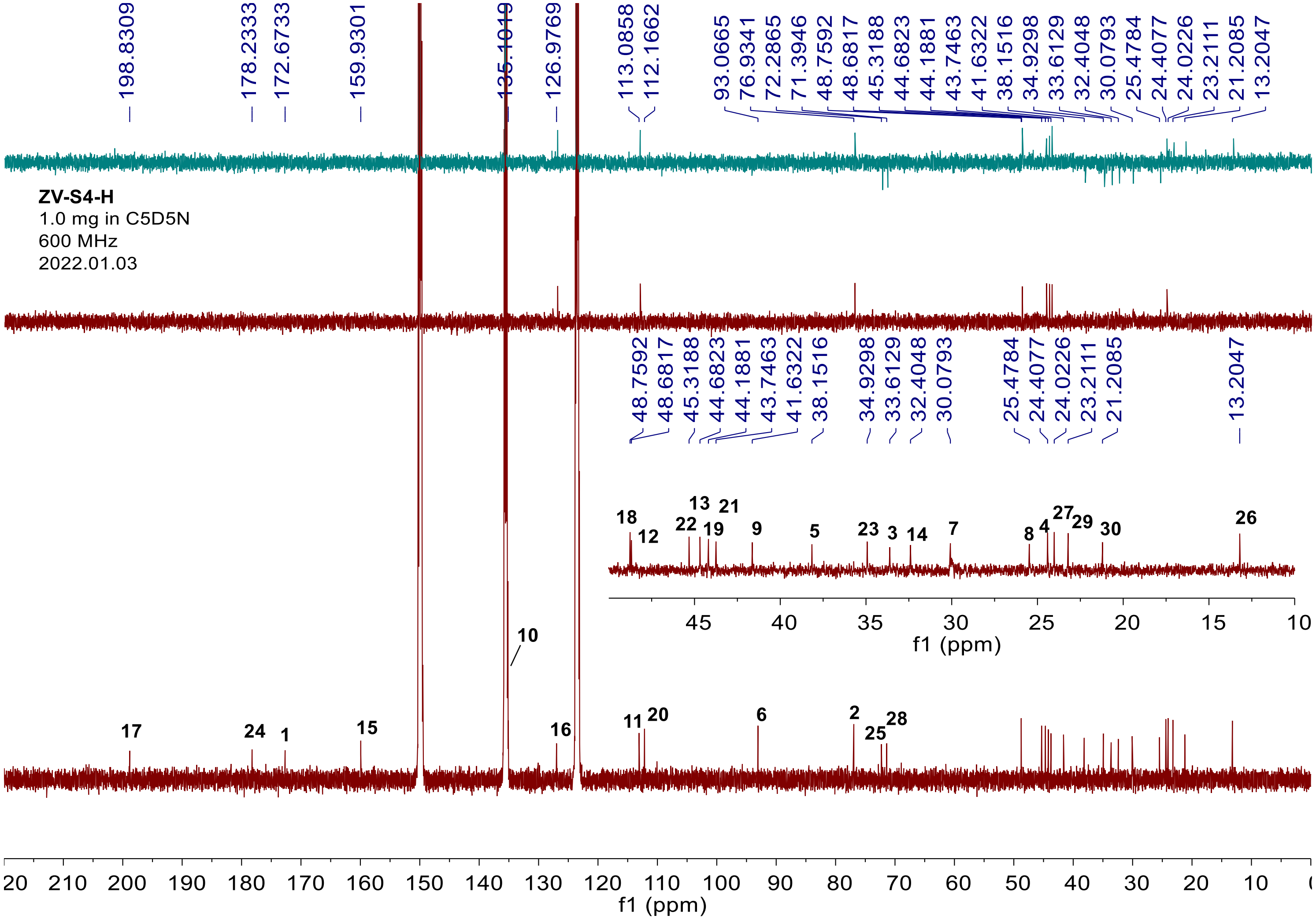

Figure S102. COSY spectrum of **12**

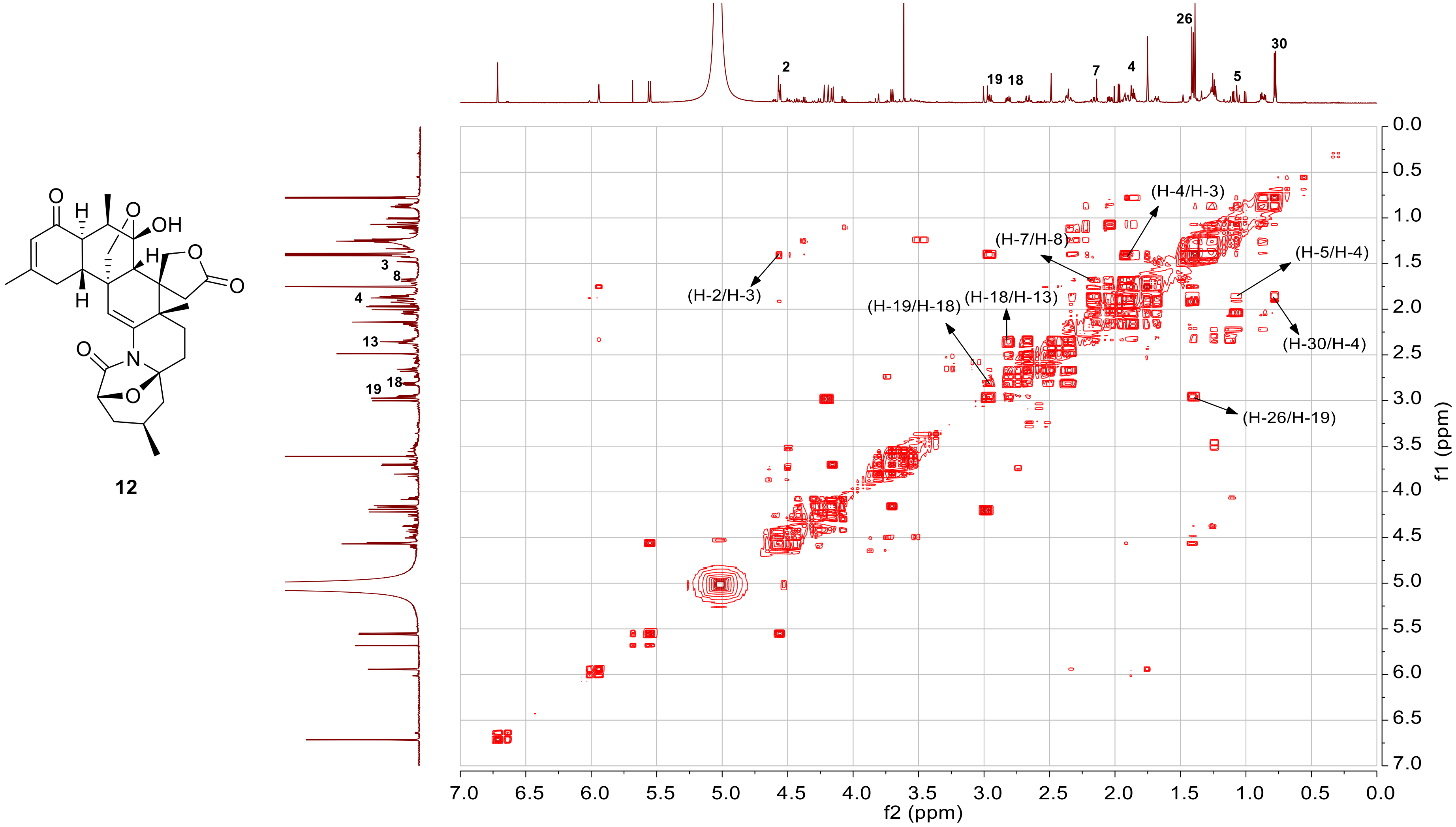

Figure S103. HSQC spectrum of **12**

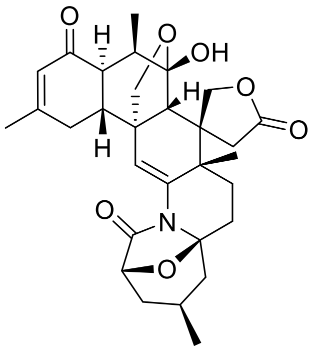

12

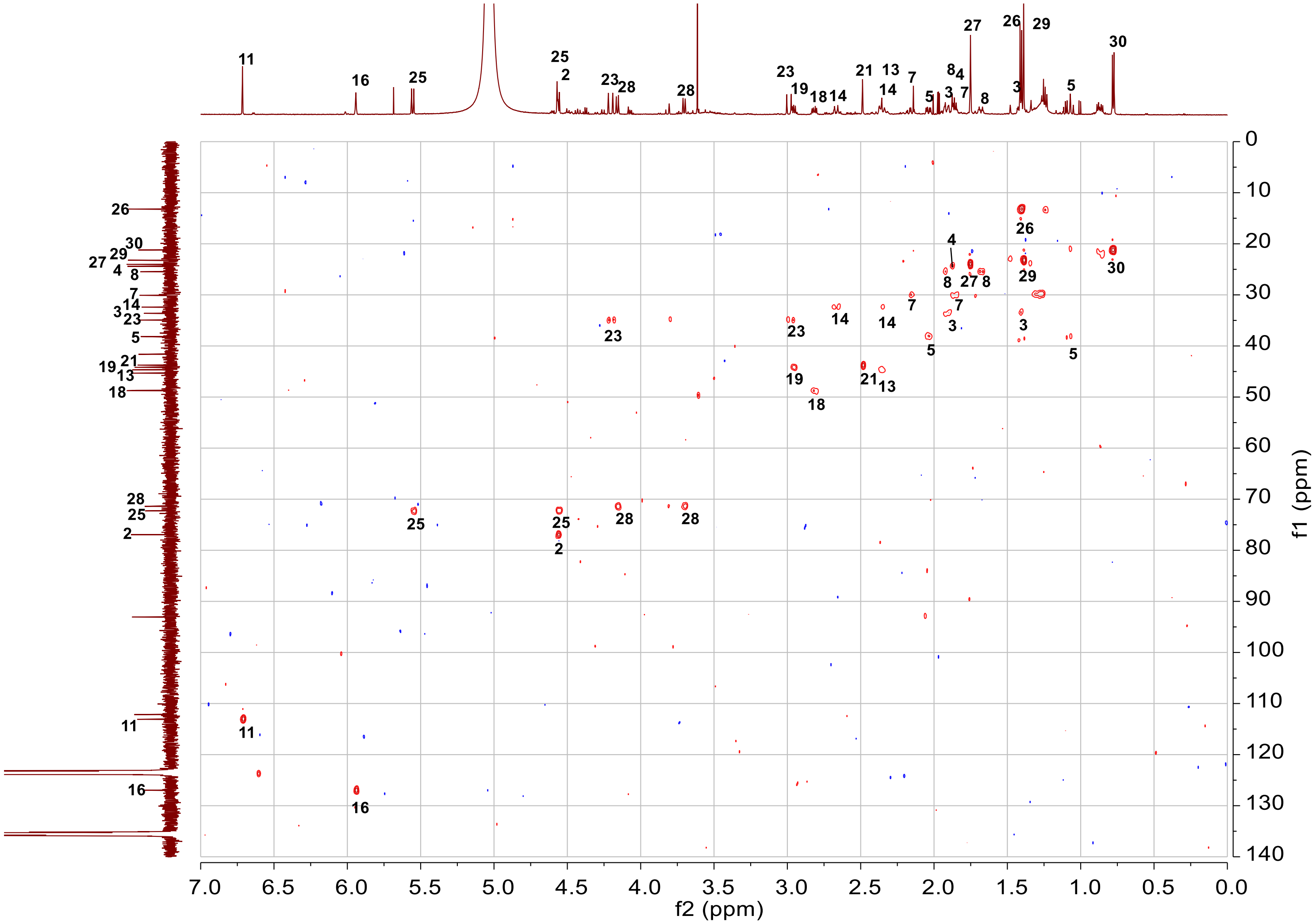

Figure S104. HMBC spectrum of **12**

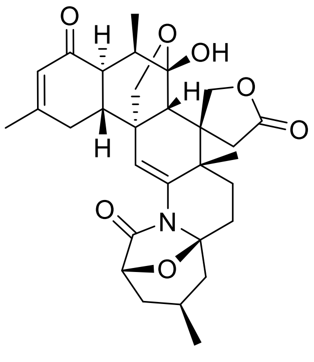

12

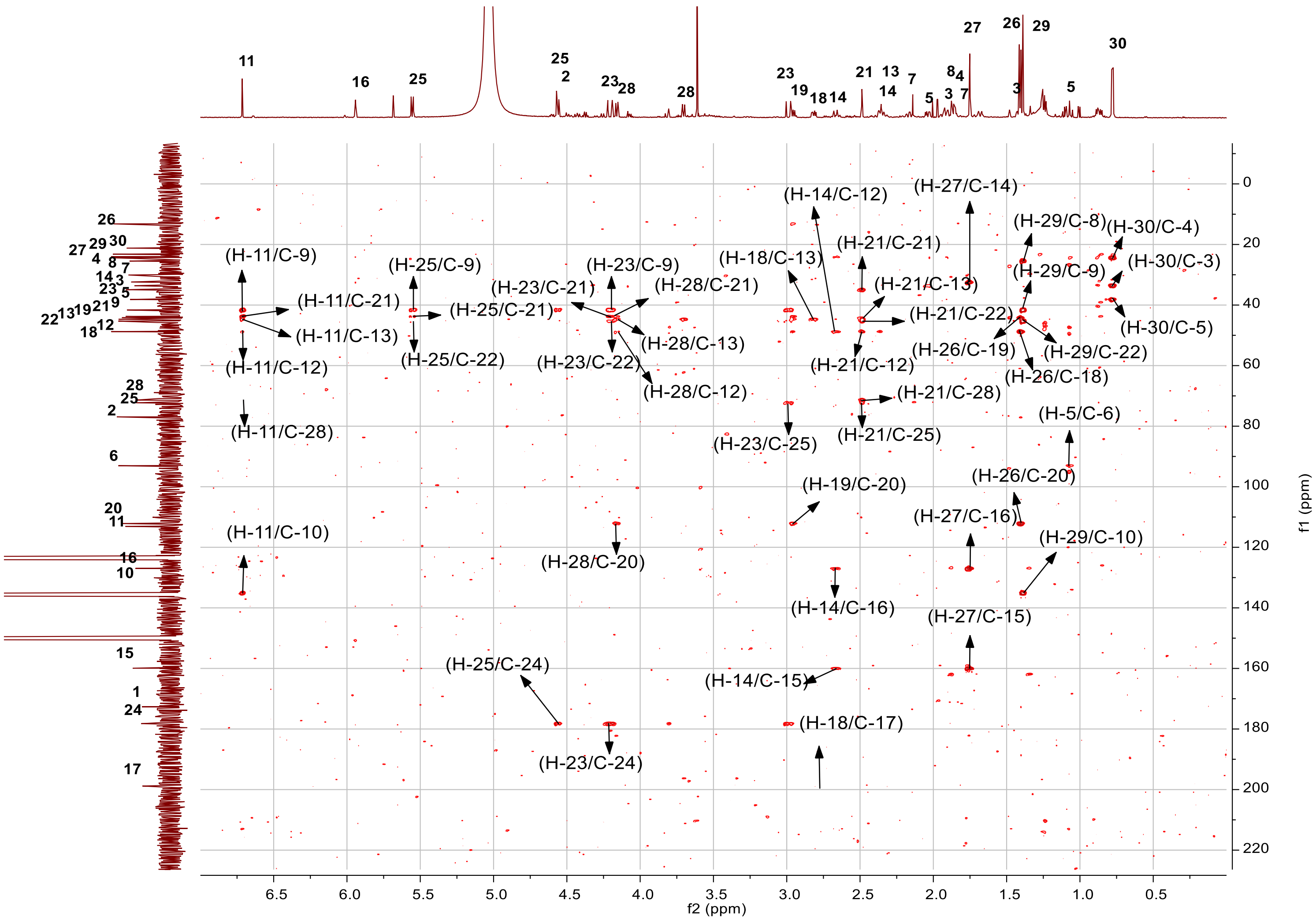

Figure S105. NOESY spectrum of **12**

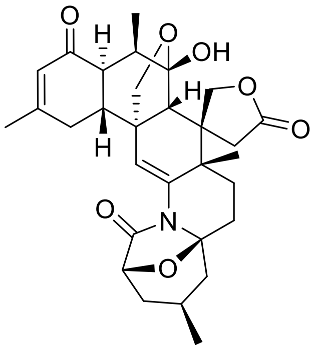

12

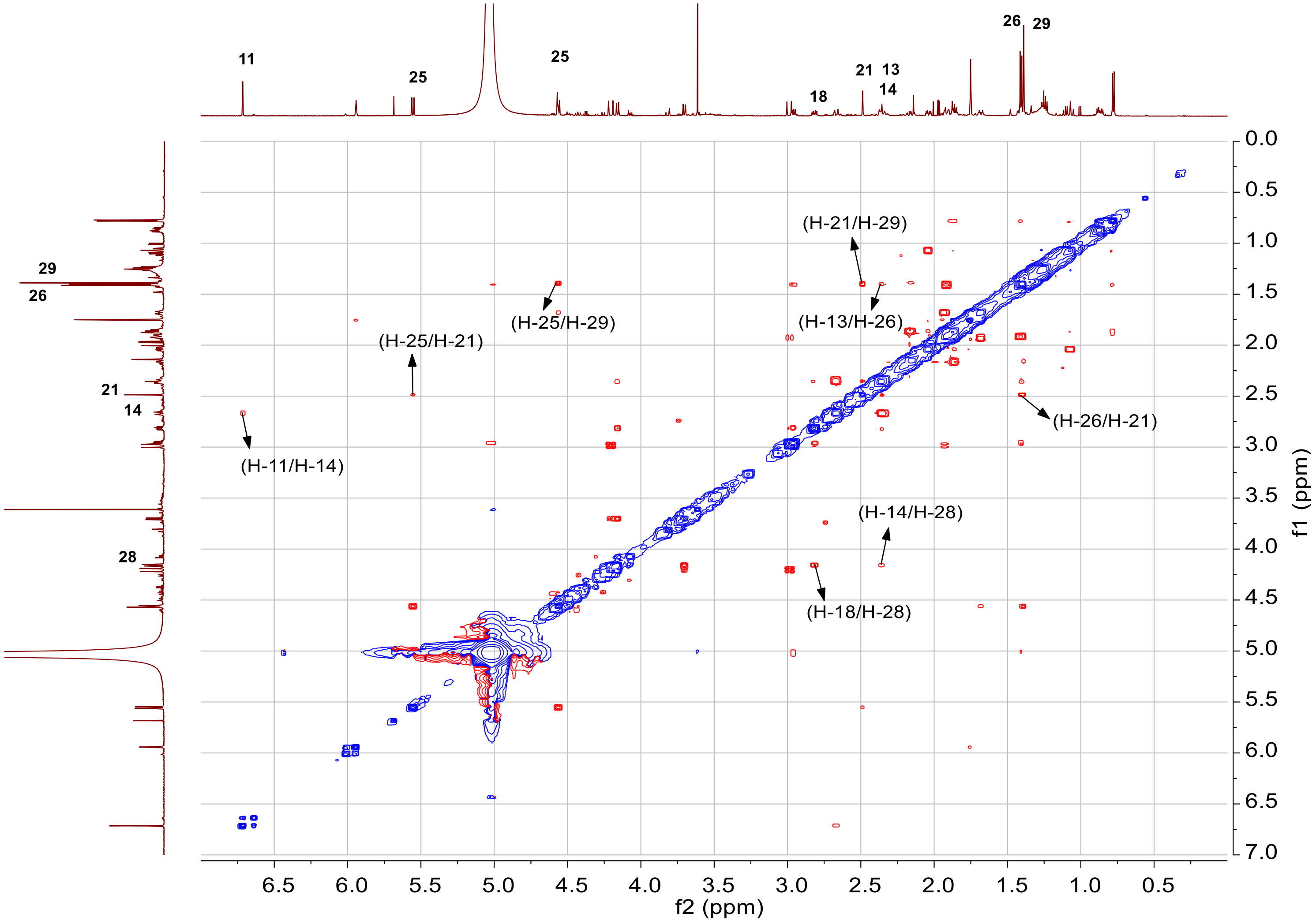

Figure S106. HRESIMS spectrum of **12**

Mass Spectrum SmartFormula Report

Analysis Info

Analysis Name	D:\QTOF\ZVS4HR.d	2/24/2022 2:58:45 PM
Method	_tune_low_pos(50-1000)_20170502_Liao lab.m	Operator: YU HSIAO-CHING
Sample Name	ZV-S4-H	Instrument: BRUKER micrOTOF-Q
Comment		

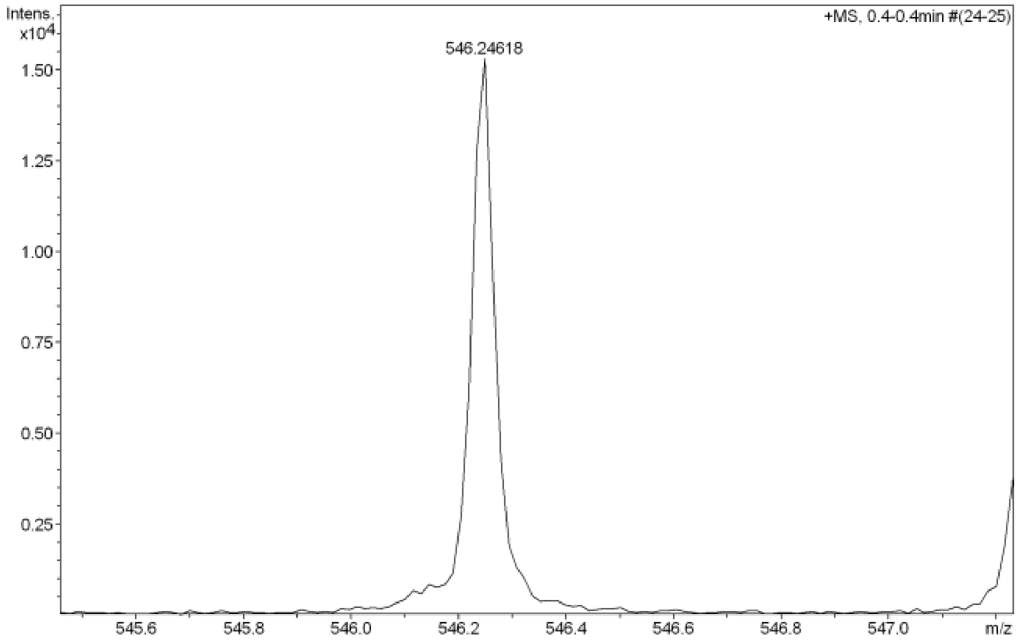

Meas. m/z	#	Formula	Score	m/z	err [mDa]	err [ppm]	mSigma	rdb	e⁻ Conf	N-Rule
546.24618	1	C 30 H 37 N Na O 7	100.00	546.24622	0.04	0.07	19.4	12.5	even	ok

Figure S107. UV spectrum of **12**

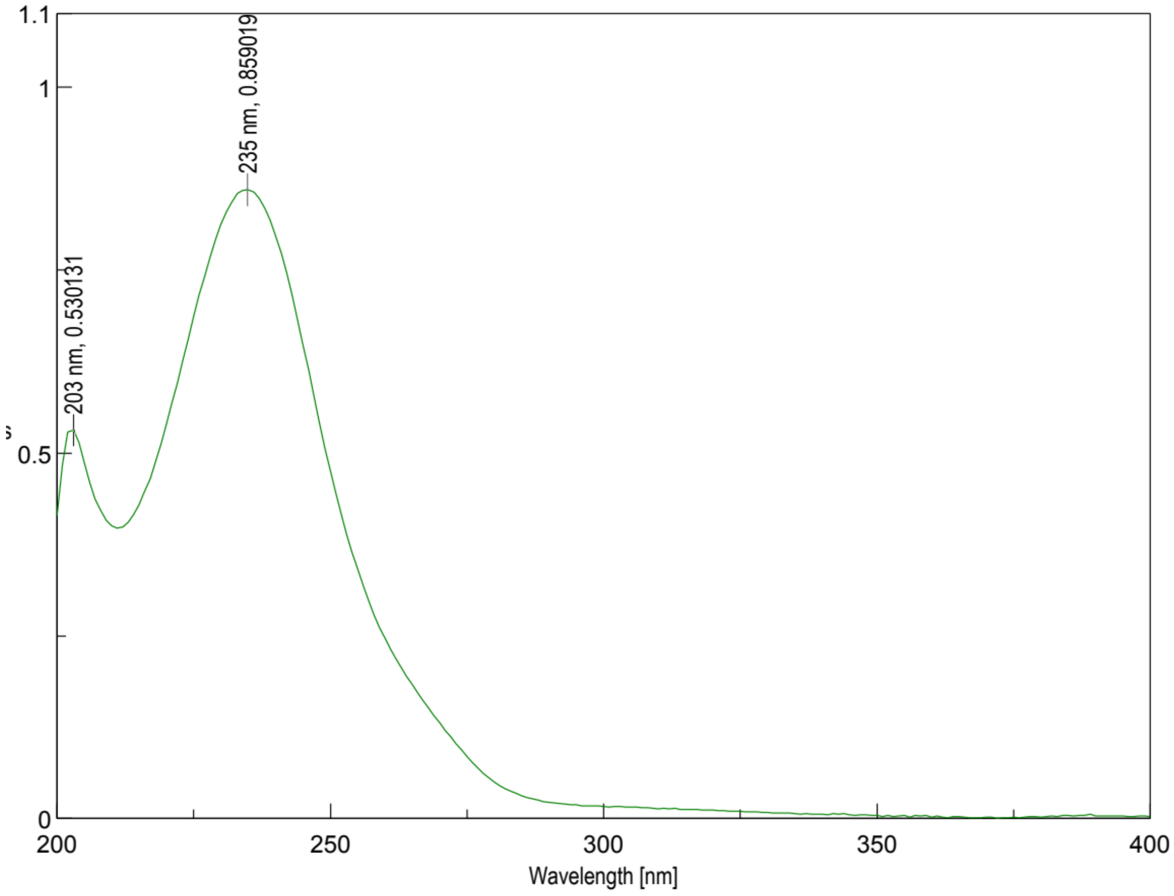

Figure S108. IR spectrum of **12**

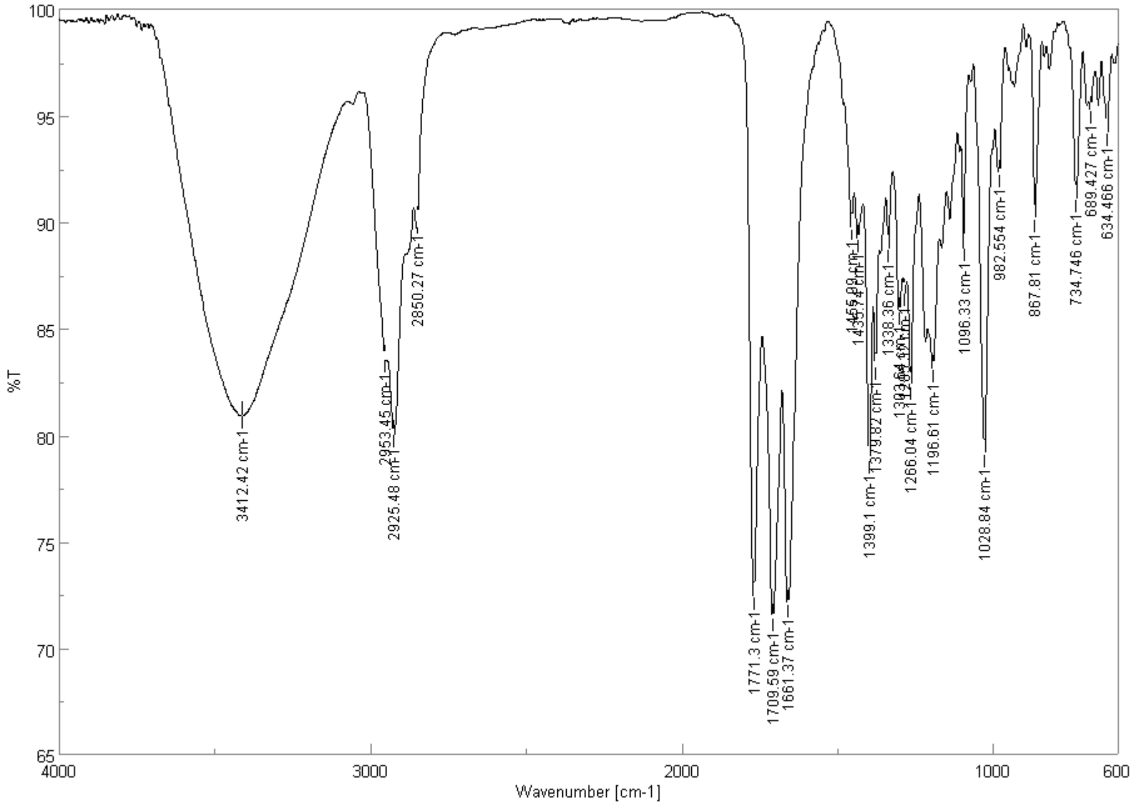

Figure S109. ^{1}H NMR spectrum of **13** (C_5D_5N, 600 MHz)

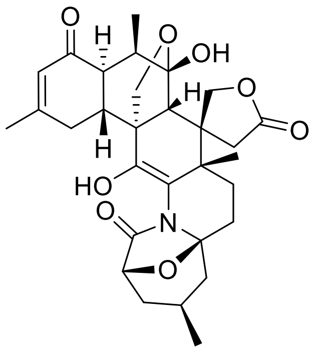

13

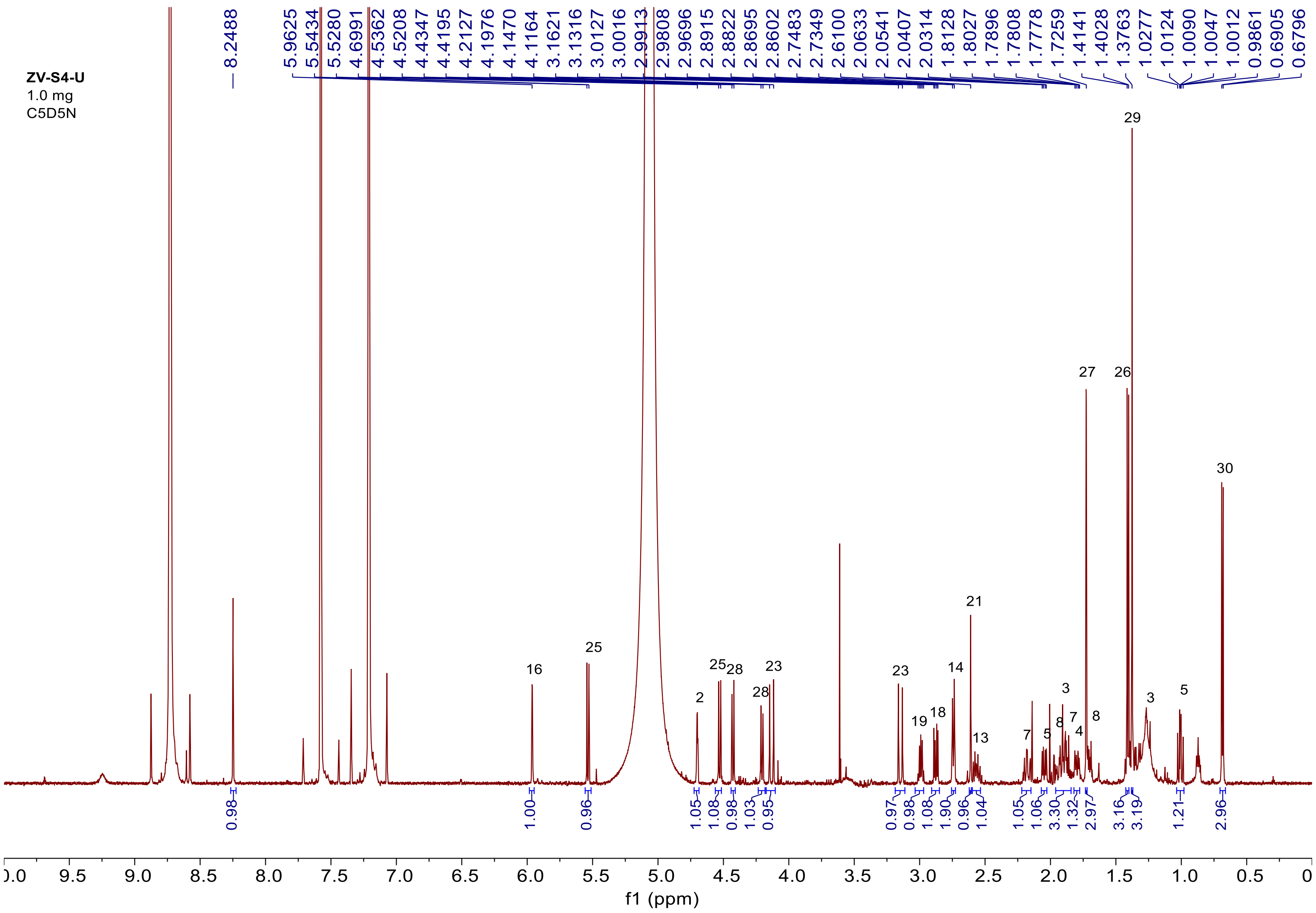

Figure S110. $^{13}C\{^{1}H\}$ NMR and DEPT spectra of **13** (C_5D_5N, 150 MHz)

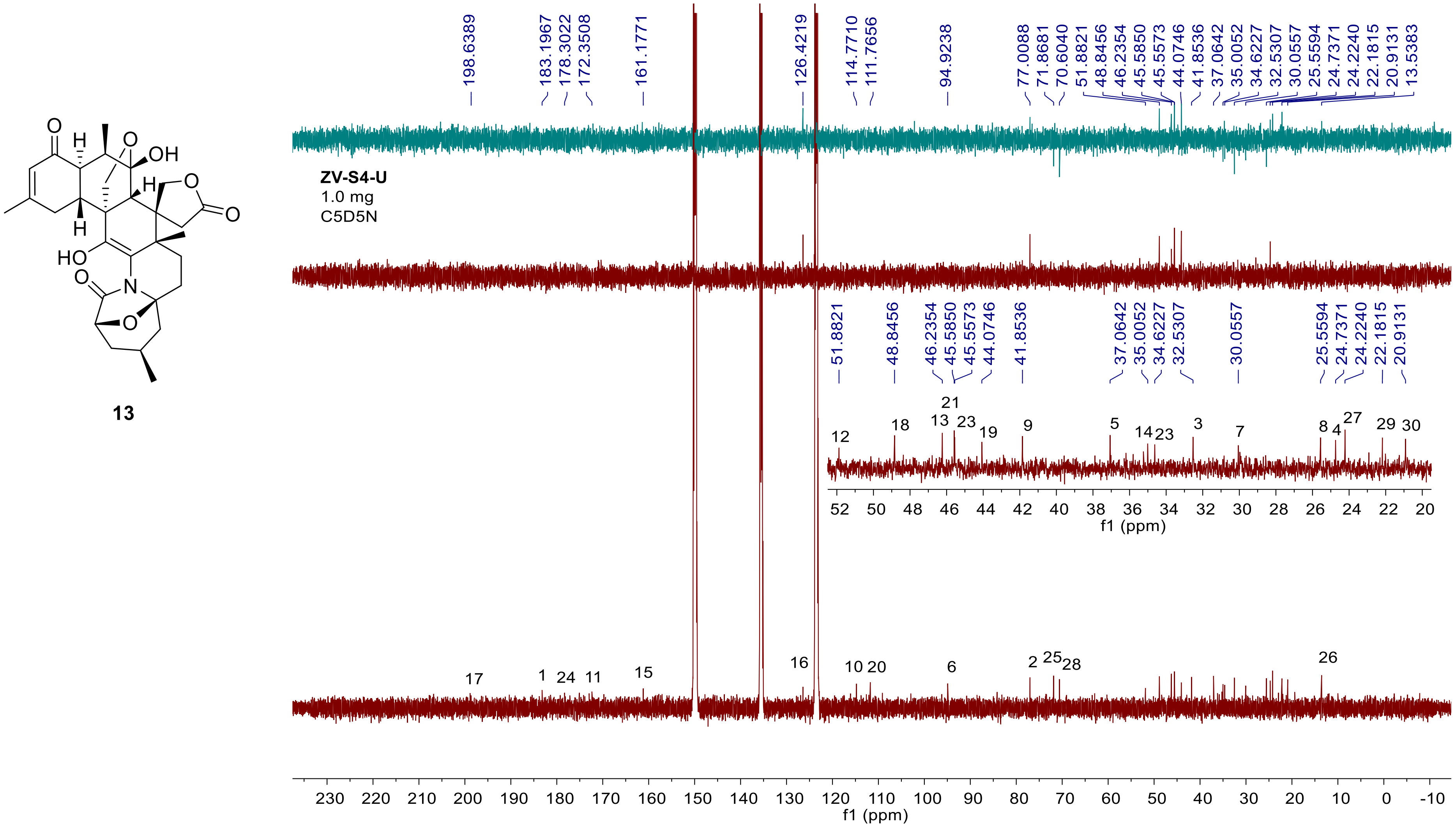

Figure S111. COSY spectrum of **13**

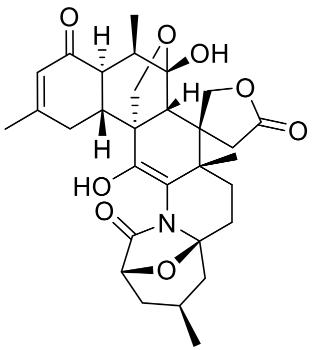

13

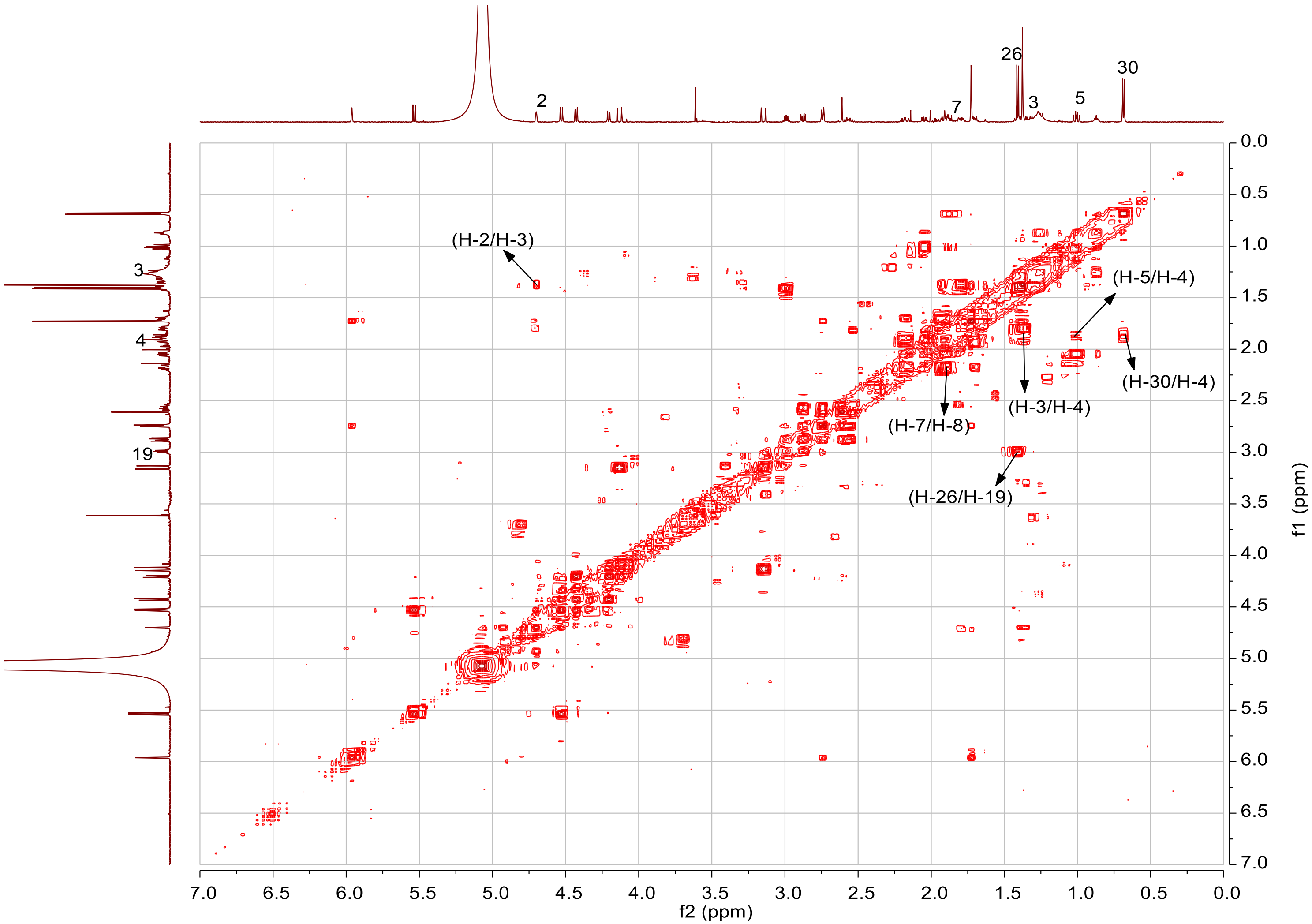

Figure S112. HSQC spectrum of **13**

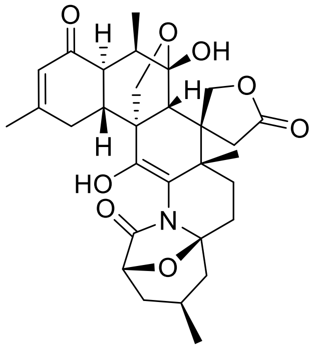

13

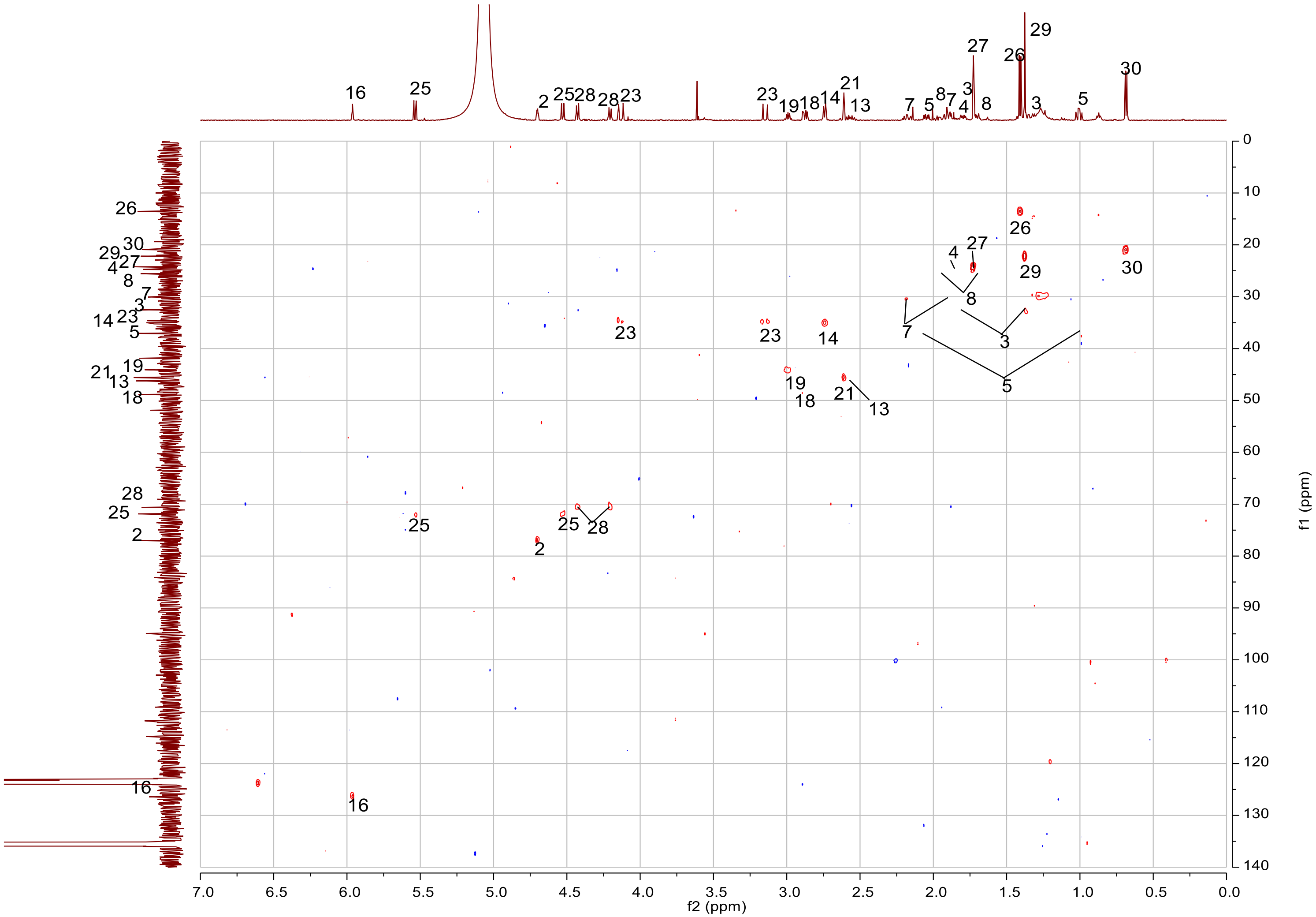

Figure S113. HMBC spectrum of **13**

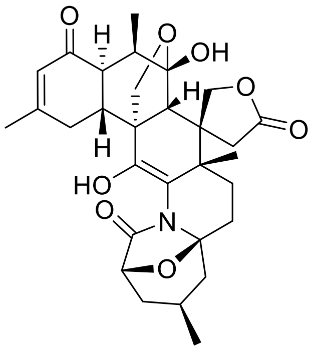

13

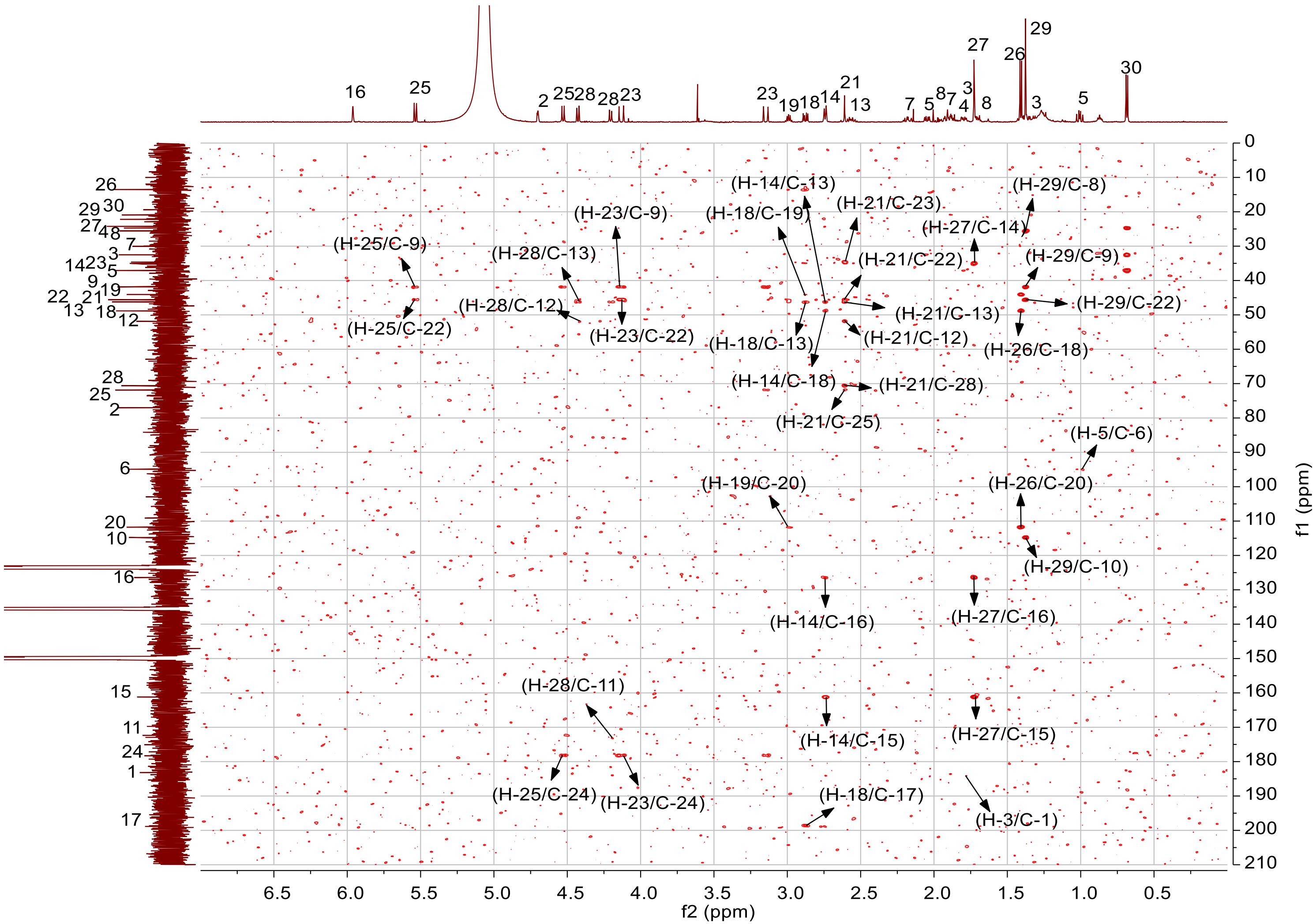

Figure S114. NOESY spectrum of **13**

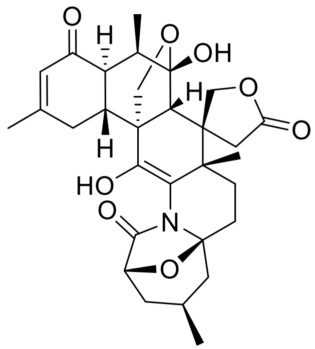

13

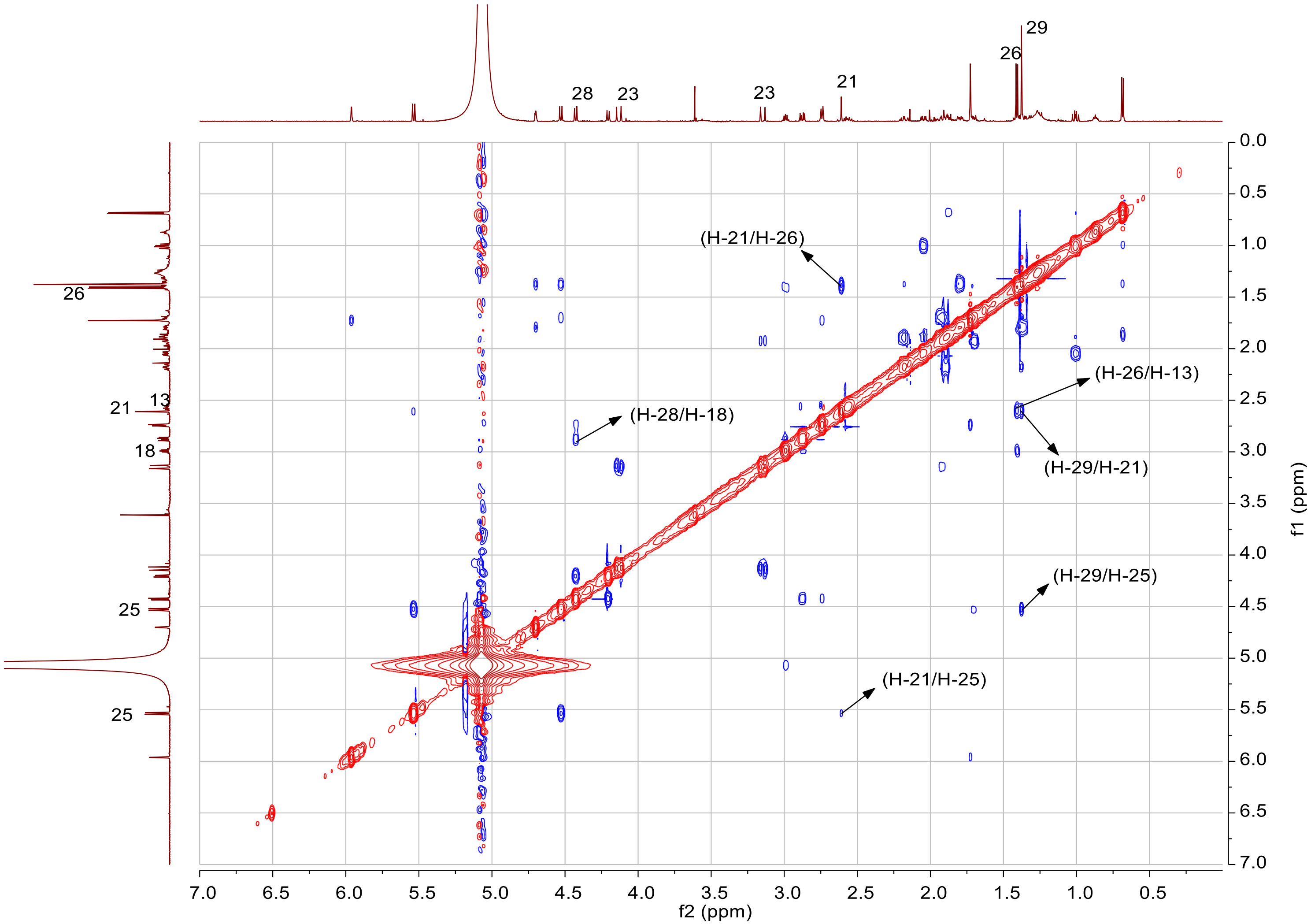

Figure S115. HRESIMS spectrum of **13**

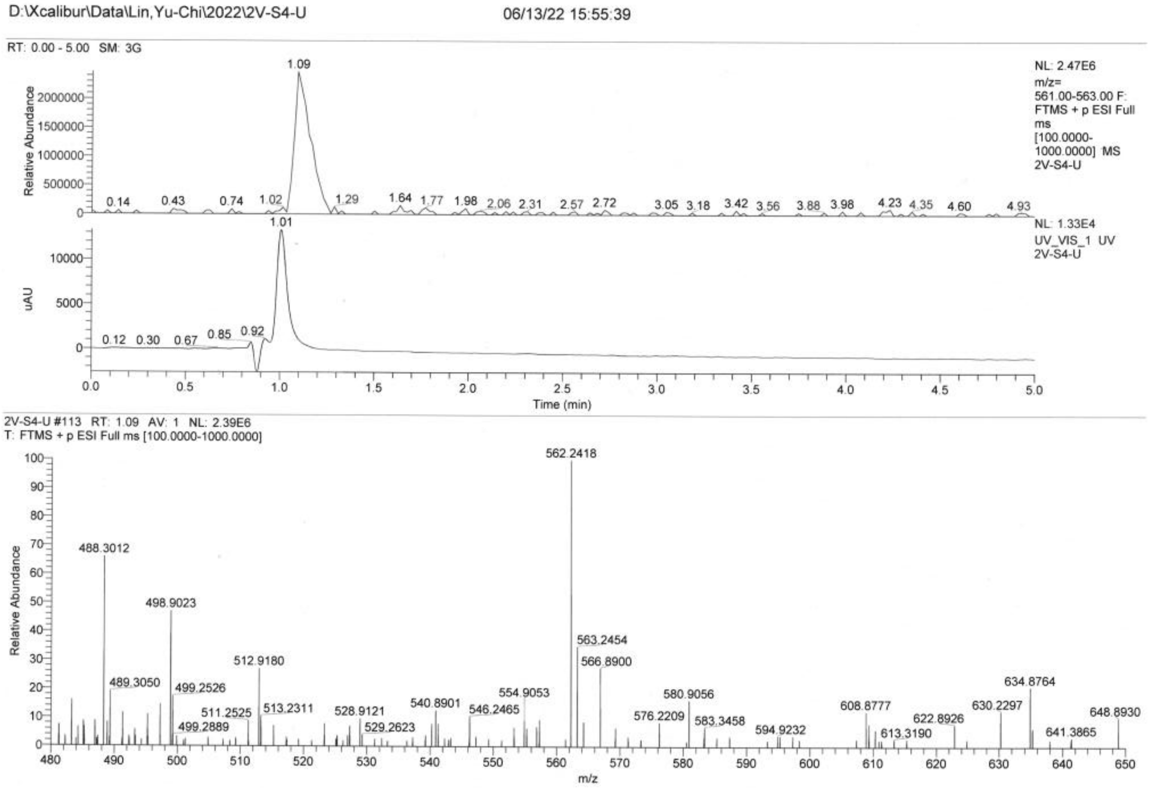

Elemental composition search on mass 562.2409

m/z= 557.2418-567.2400

m/z	Theo. Mass	Delta (mmu)	RDB equiv.	Composition
562.2418	562.2411	0.68	12.5	$C_{30}H_{37}O_8NNa$
	562.2409	0.96	11.0	$C_{29}H_{38}O_{11}$
	562.2435	-1.72	15.5	$C_{32}H_{36}O_8N$
	562.2443	-2.51	-1.0	$C_{20}H_{43}O_{16}Na$
	562.2385	3.36	8.0	$C_{27}H_{39}O_{11}Na$
	562.2377	4.15	24.5	$C_{39}H_{32}O_3N$
	562.2467	-4.92	2.0	$C_{22}H_{42}O_{16}$
	562.2470	-5.19	3.5	$C_{23}H_{41}O_{13}NNa$
	562.2478	-6.02	21.0	$C_{38}H_{35}O_3Na$
	562.2353	6.55	21.5	$C_{37}H_{33}O_3NNa$

Figure S116. UV spectrum of **13**

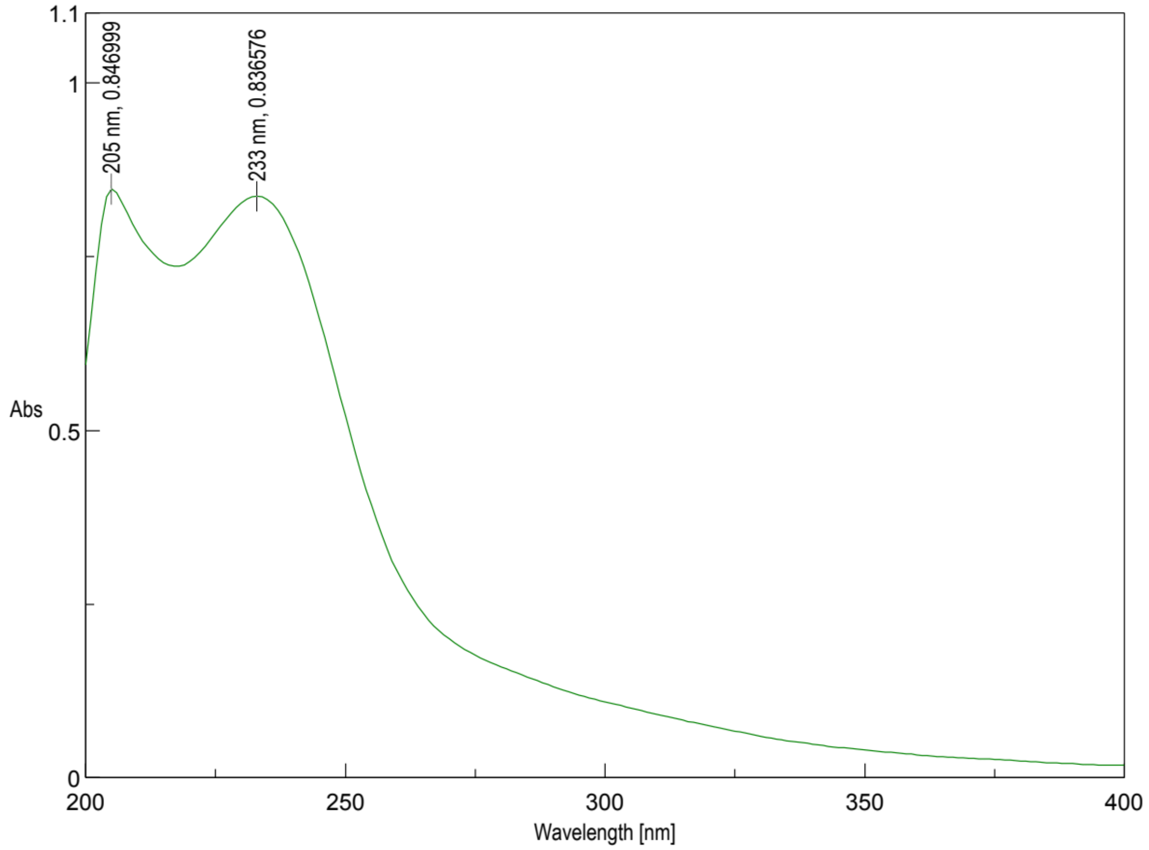

Figure S117. IR spectrum of **13**

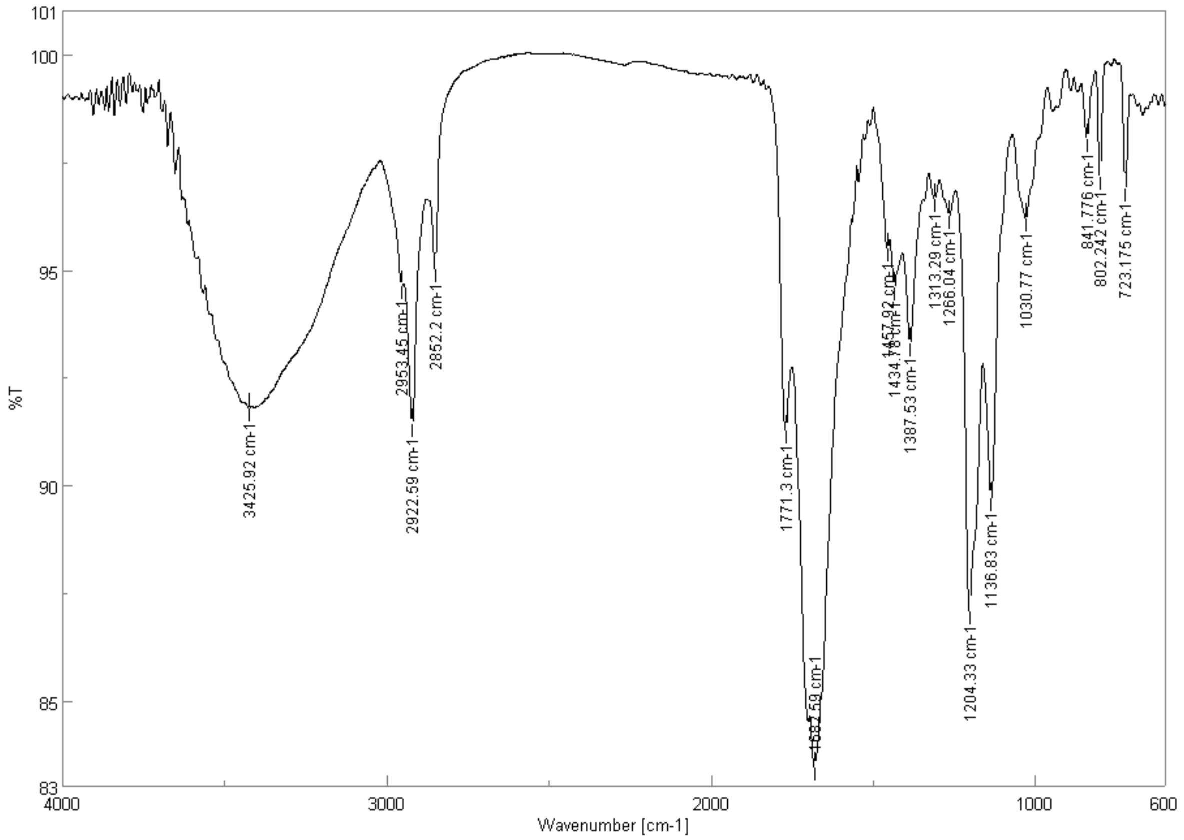

Figure S118. ^{1}H NMR spectrum of **14** (C_5D_5N, 400 MHz)

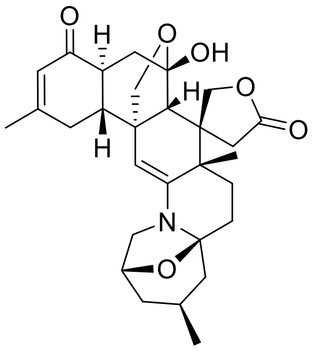

14

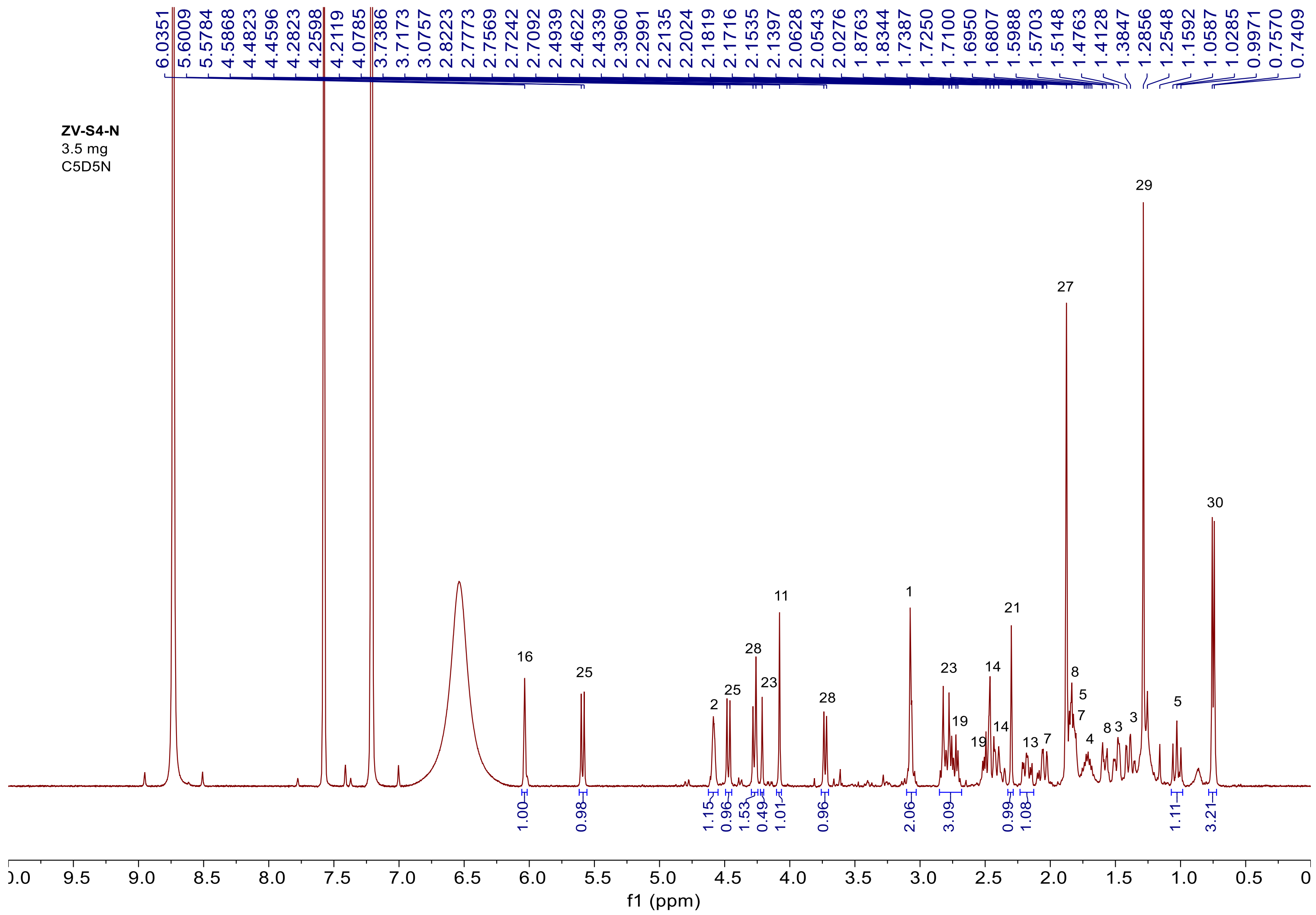

Figure S119. $^{13}C\{^1H\}$ NMR and DEPT spectra of **14** (C_5D_5N, 100 MHz)

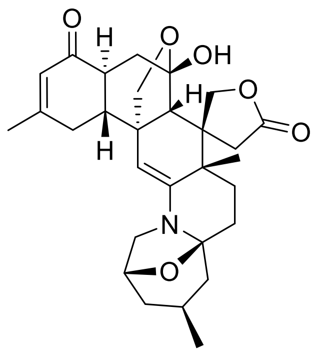

14

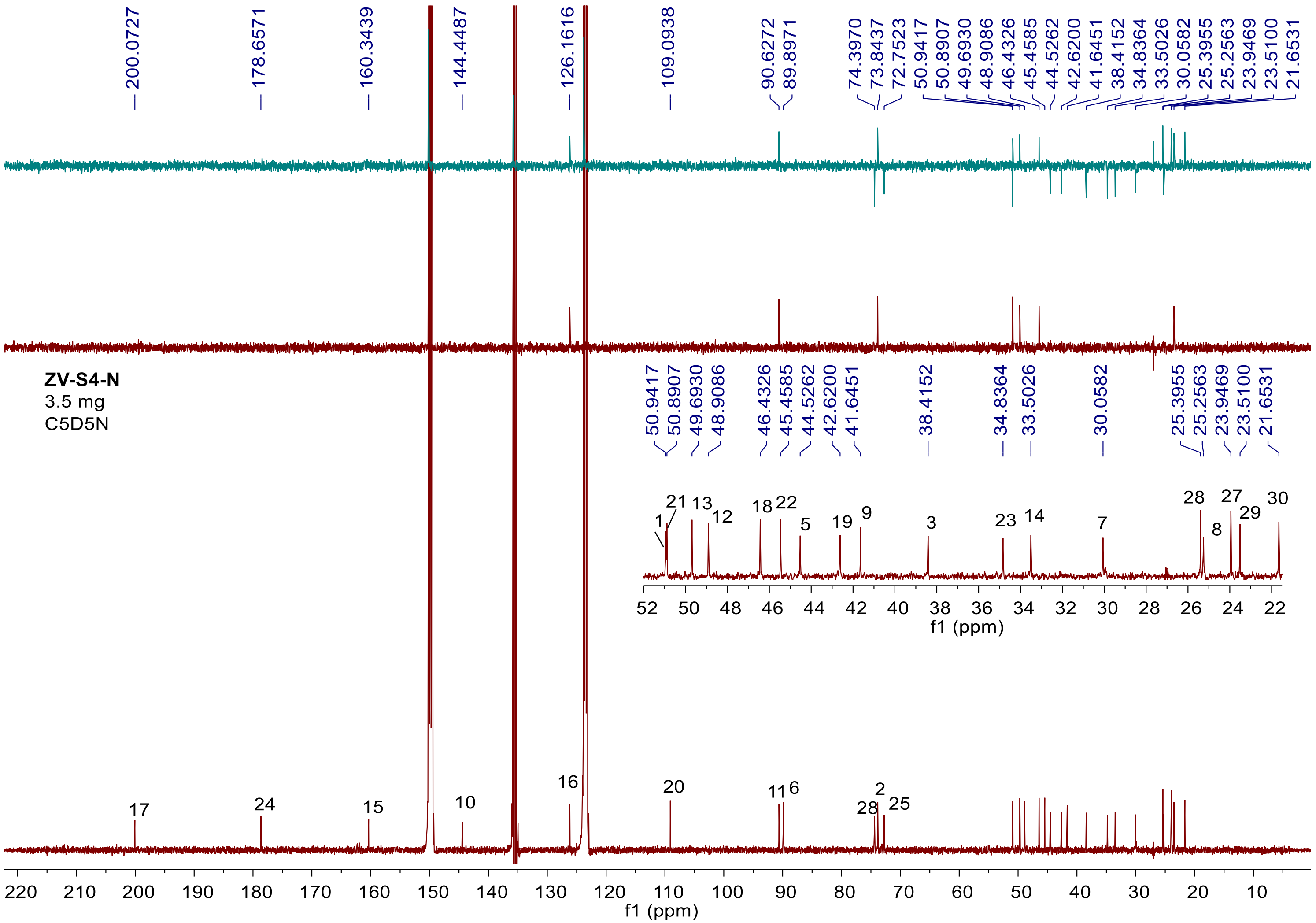

Figure S120. COSY spectrum of **14**

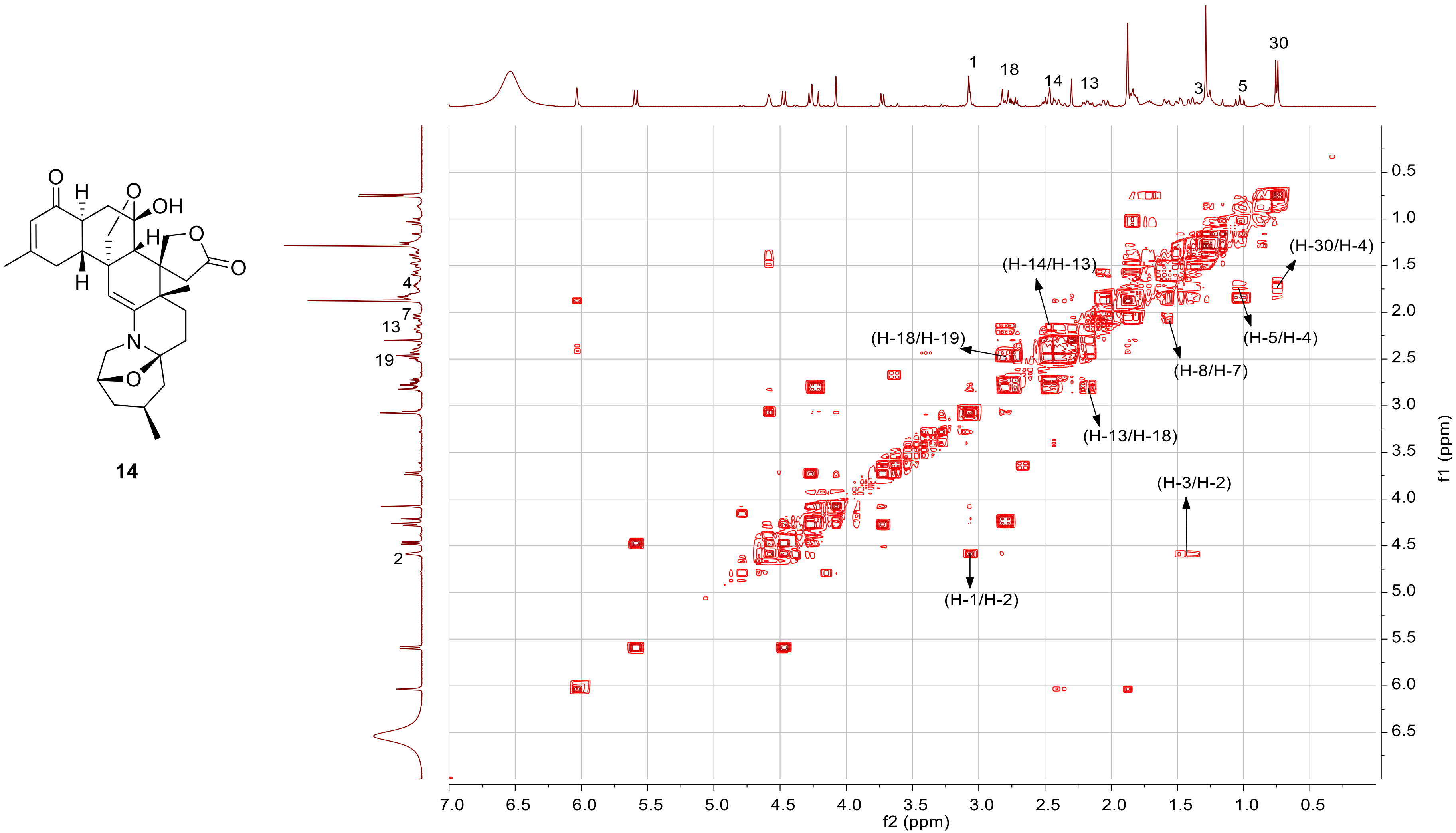

Figure S121. HSQC spectrum of **14**

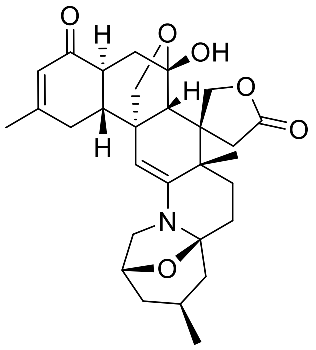

14

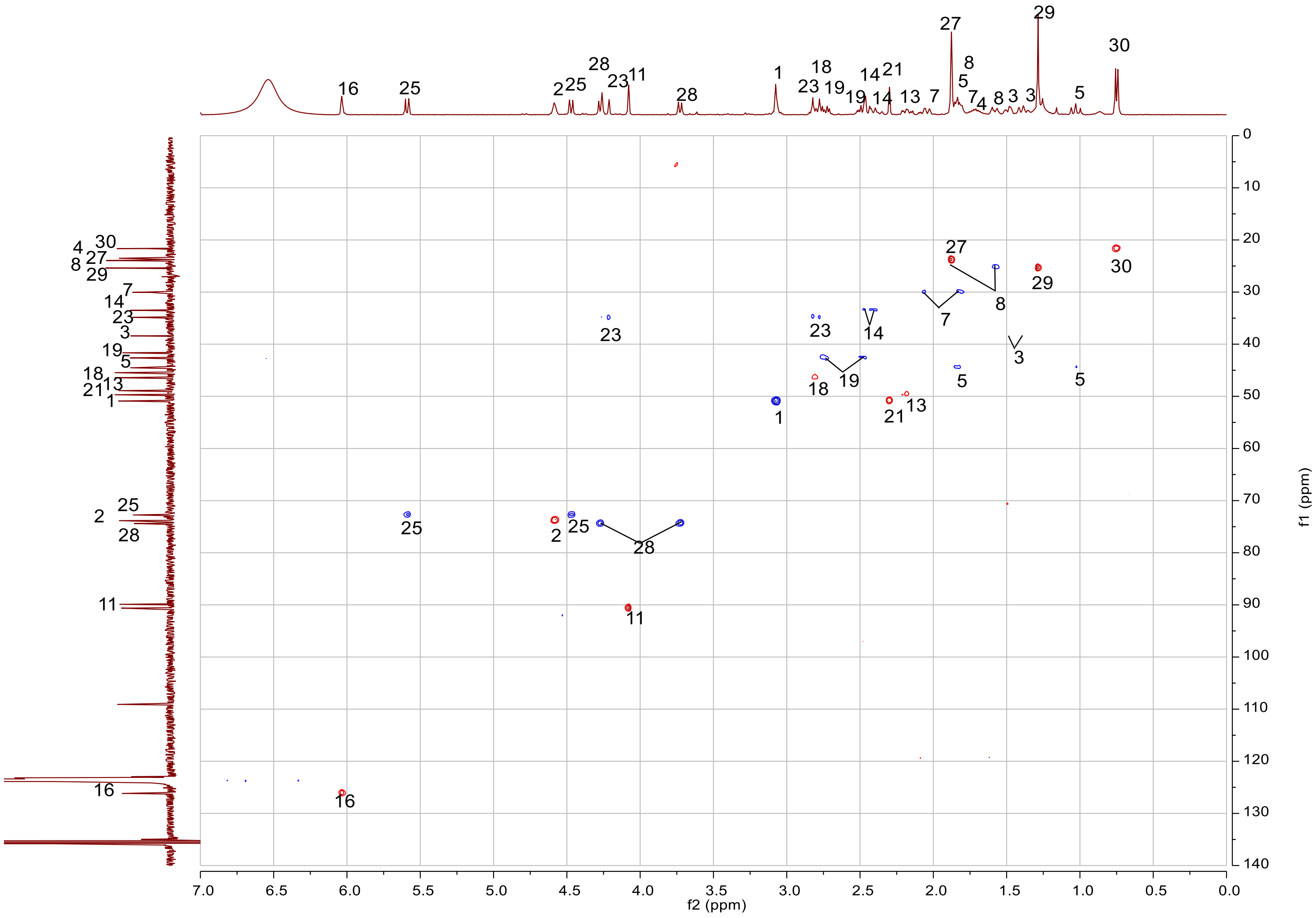

Figure S122. HMBC spectrum of **14**

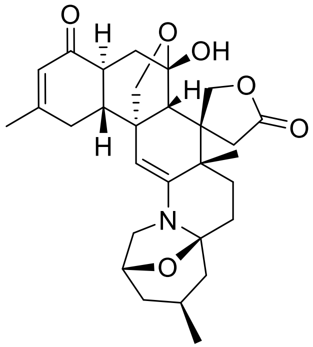

14

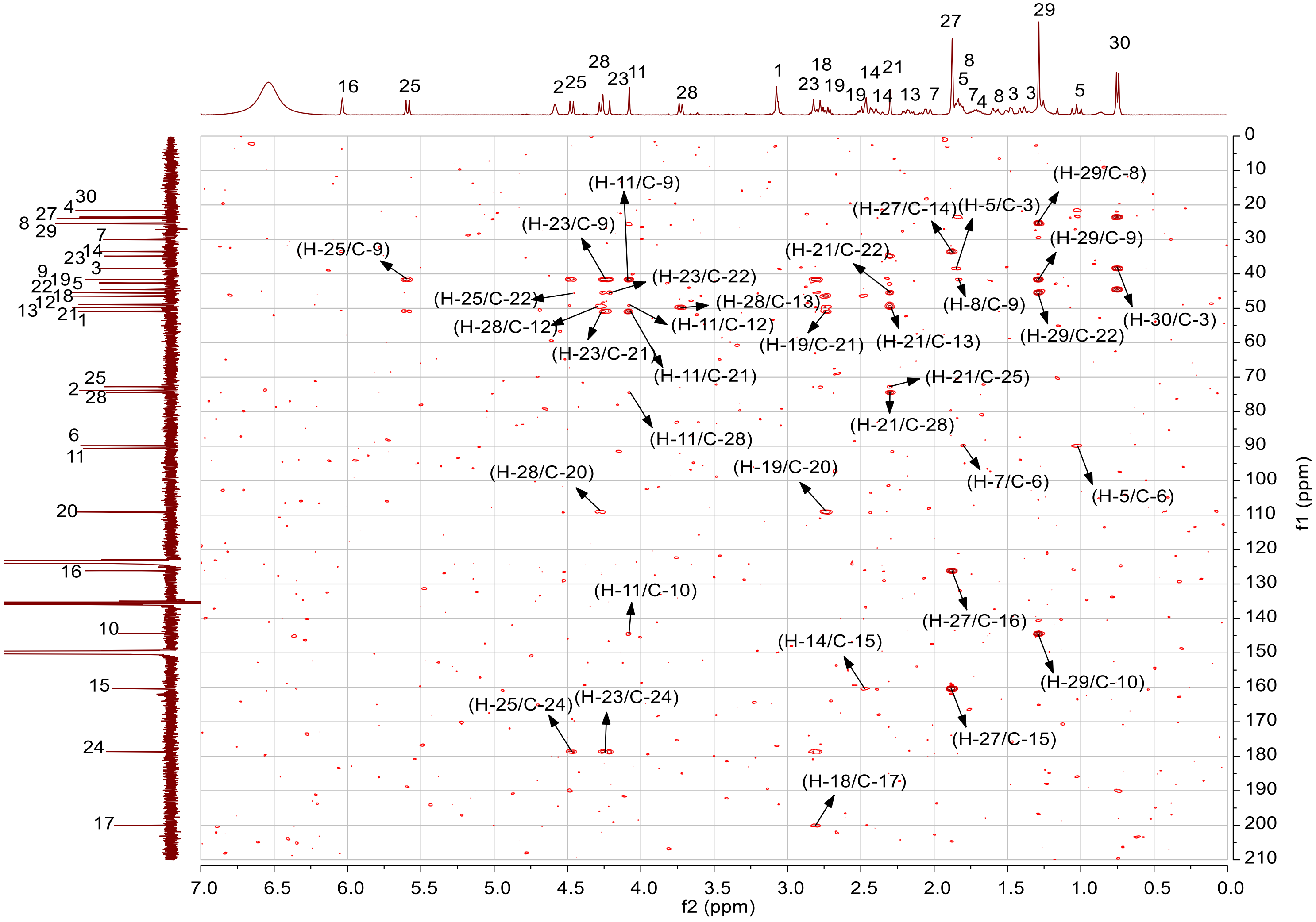

Figure S123. NOESY spectrum of **14**

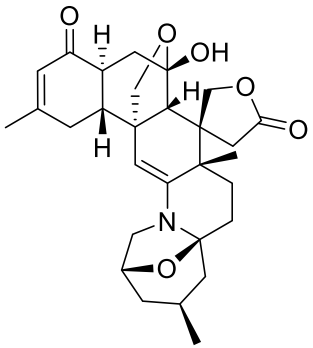

14

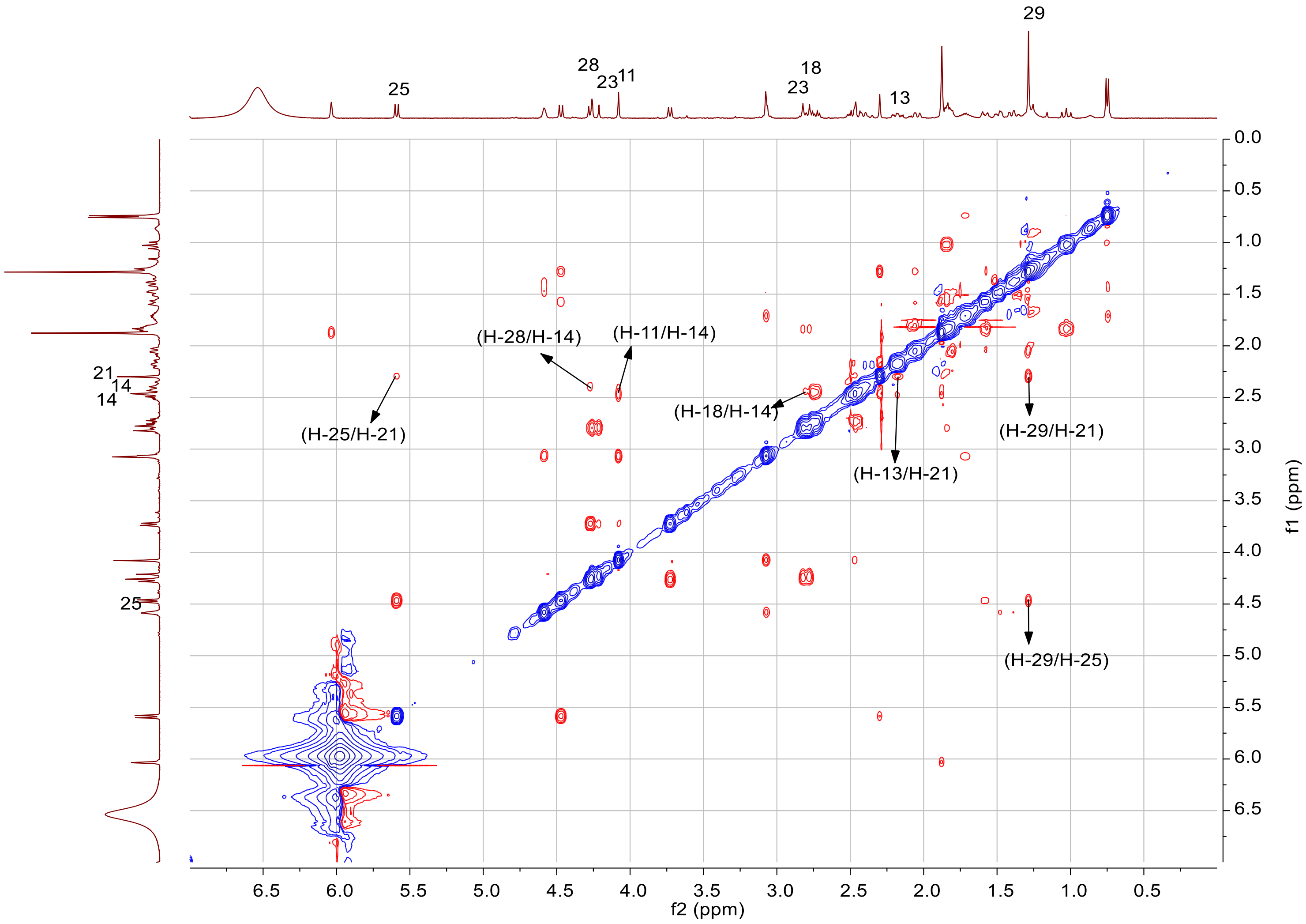

Figure S124. HRESIMS spectrum of **14**

Mass Spectrum SmartFormula Report

Analysis Info

Analysis Name	D:\QTOF\ZVS4NR.d	1/25/2022 1:25:20 PM
Method	_tune_low_pos(50-1000)_20170502_Liao lab.m	Operator: YU HSIAO-CHING
Sample Name	ZV-S4-N	Instrument: BRUKER micrOTOF-Q
Comment	ESI Positive	

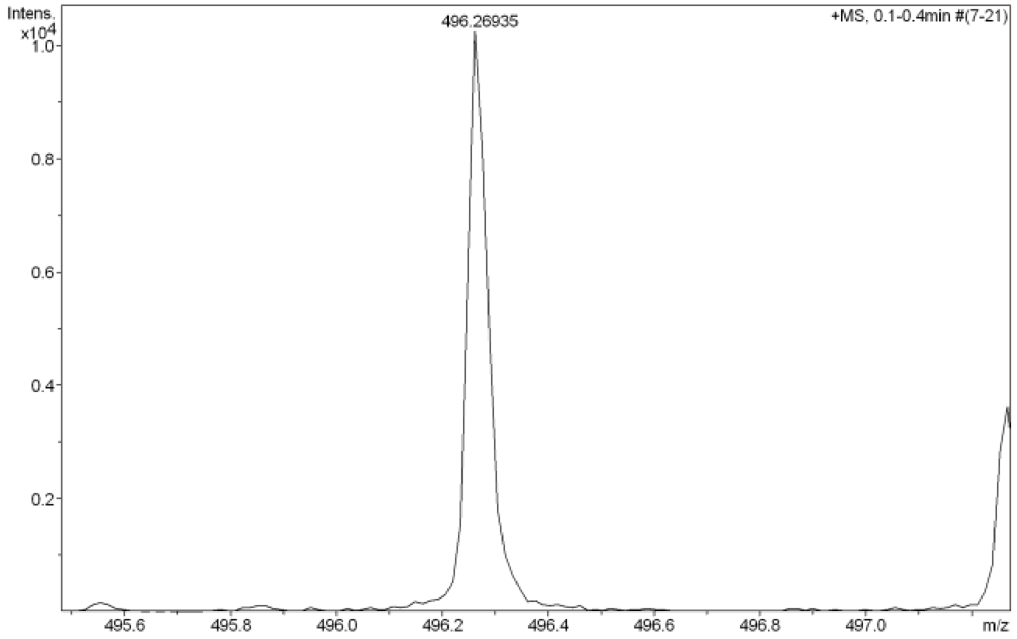

Meas. m/z	#	Formula	Score	m/z	err [mDa]	err [ppm]	mSigma	rdb	e⁻ Conf	N-Rule
496.26935	1	C 29 H 38 N O 6	100.00	496.26936	0.01	0.03	16.4	11.5	even	ok

Figure S125. UV spectrum of **14**

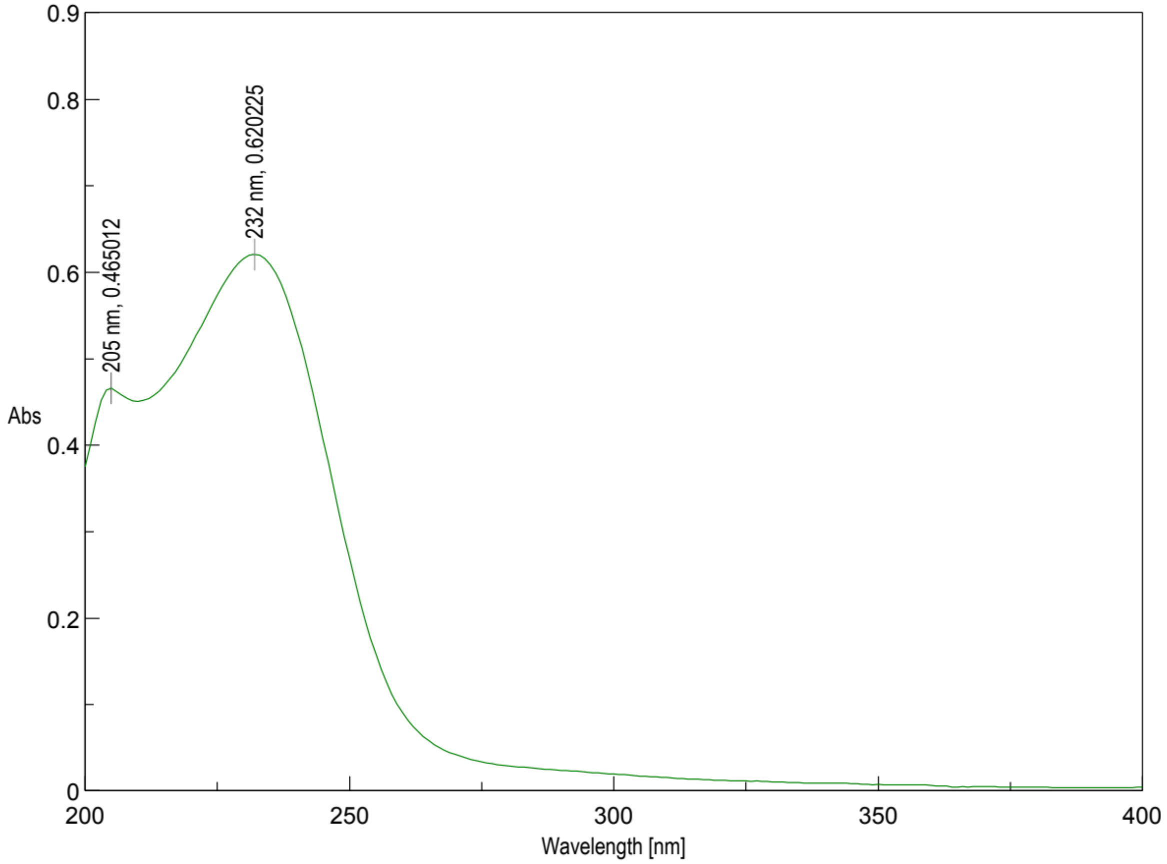

Figure S126. IR spectrum of **14**

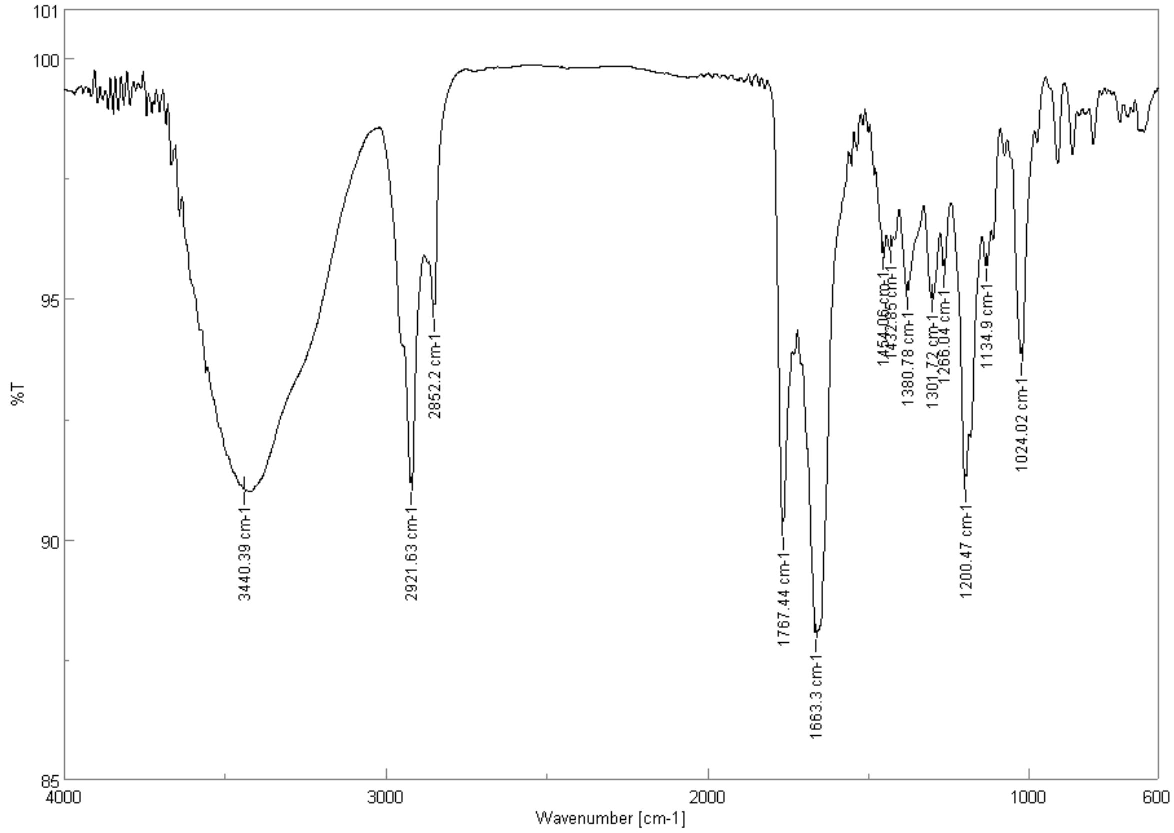

Figure S127. X-ray ORTEP drawings of 18-*epi*-kuroshine E (**16**) (displacement ellipsoids are drawn at the 50% probability level).

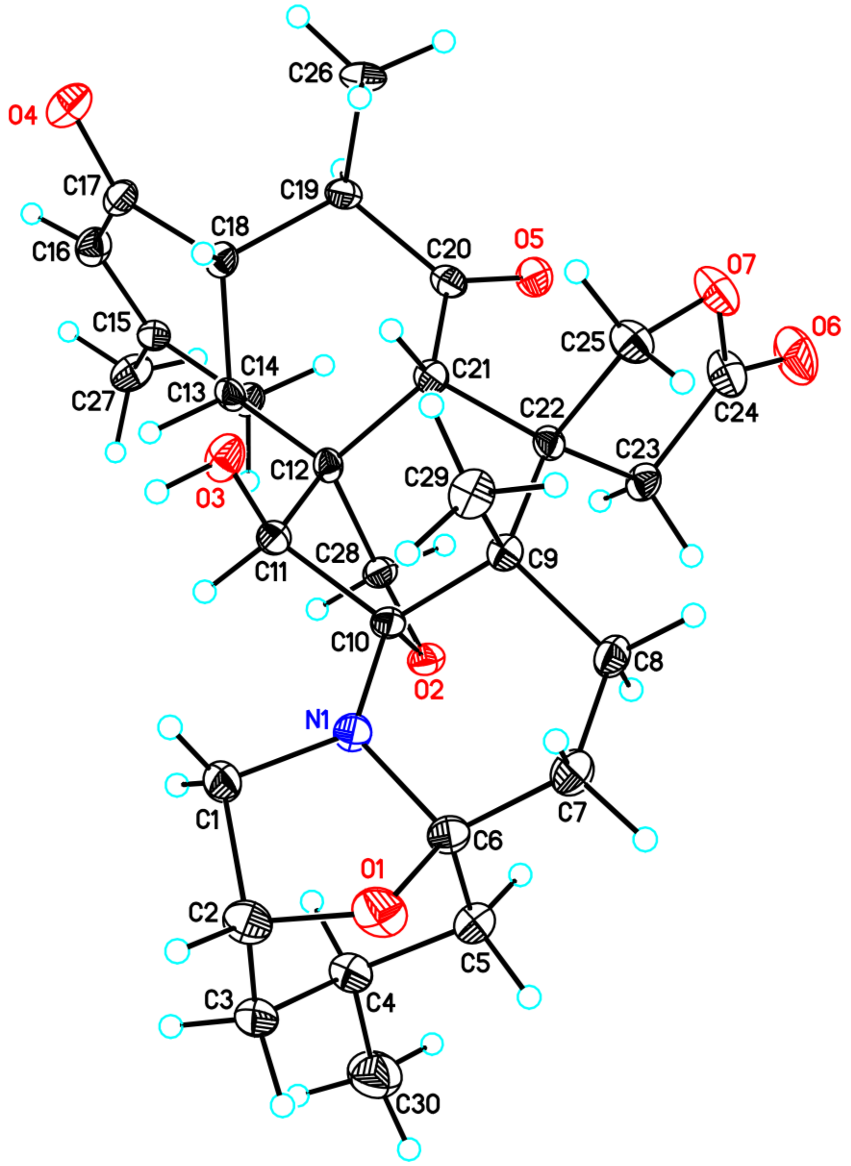

Figure S128. X-ray ORTEP drawings of kuroshine E (displacement ellipsoids are drawn at the 50% probability level).

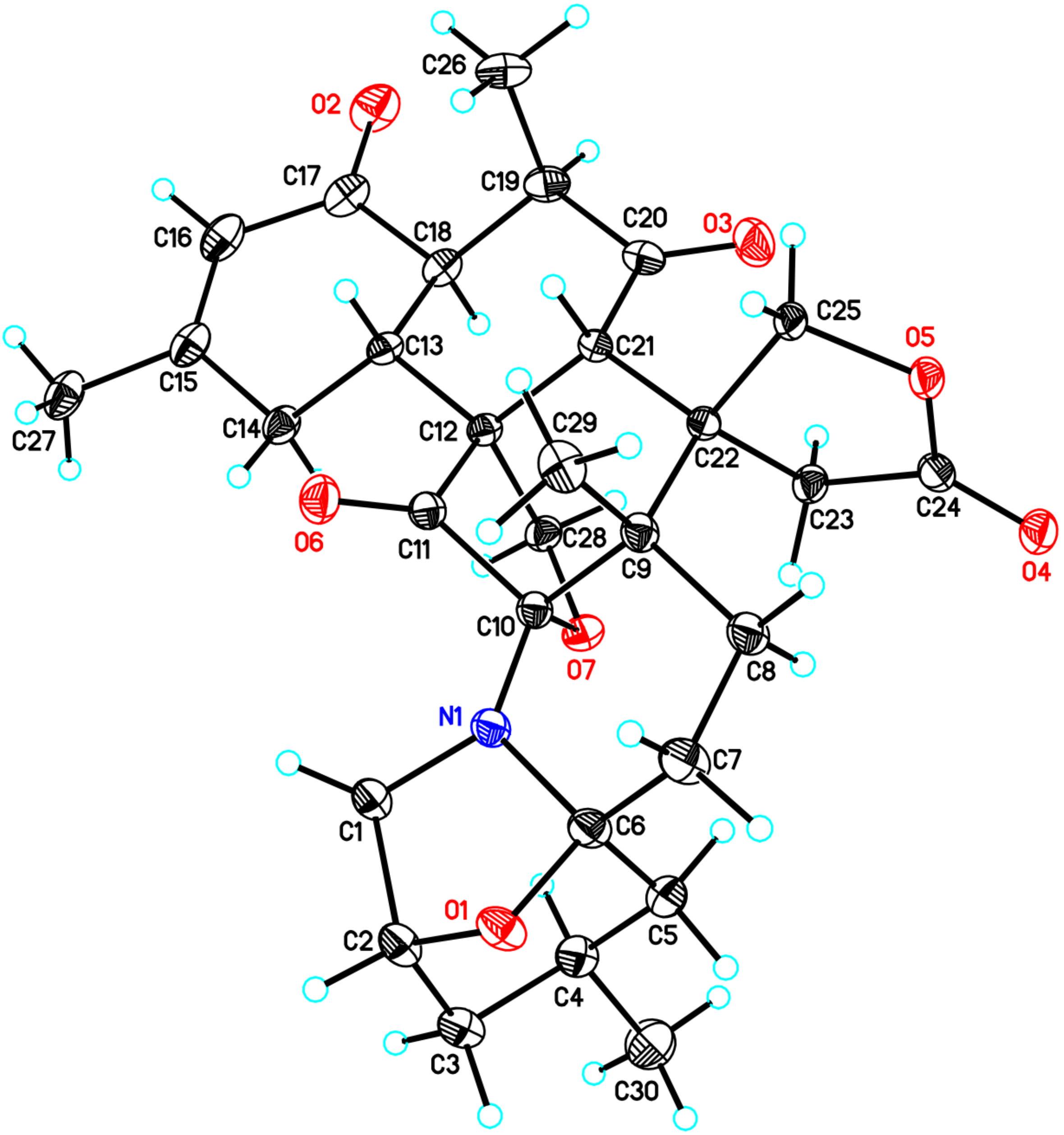

Figure S129. X-ray ORTEP drawings of 28-deoxyzoanthenamine (displacement ellipsoids are drawn at the 50% probability level).

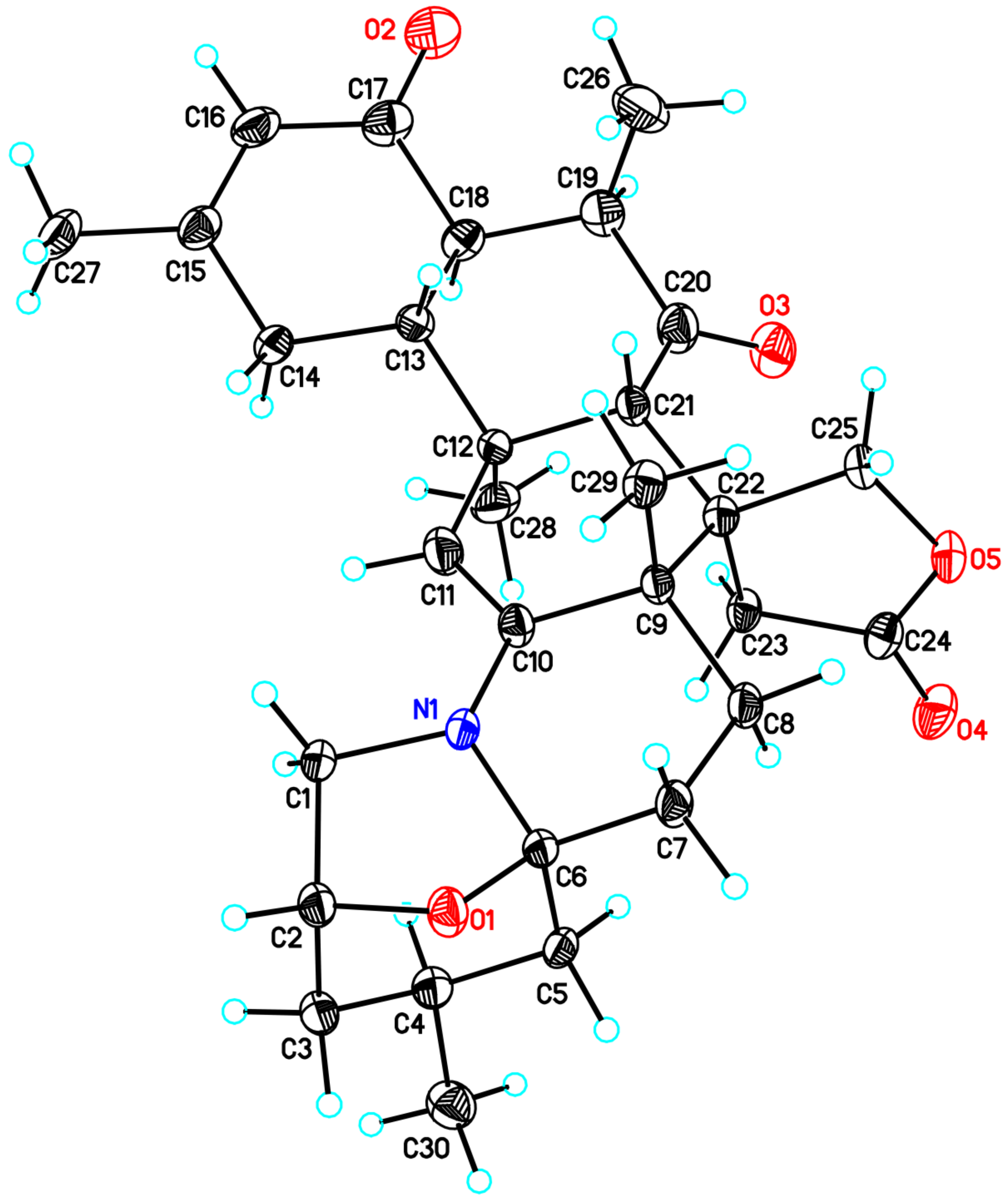

Supplement: Supplementary file 1 — jo5c00280_si_001.pdf [file jo5c00280_si_001.pdf]
